# The Octadecanoids: Synthesis and Bioactivity of 18-Carbon Oxygenated Fatty Acids in Mammals, Bacteria, and Fungi

**DOI:** 10.1021/acs.chemrev.3c00520

**Published:** 2024-12-16

**Authors:** Johanna Revol-Cavalier, Alessandro Quaranta, John W. Newman, Alan R. Brash, Mats Hamberg, Craig E. Wheelock

**Affiliations:** † Unit of Integrative Metabolomics, Institute of Environmental Medicine, Karolinska Institutet, Stockholm SE-171 77, Sweden; ‡ Larodan Research Laboratory, Karolinska Institutet, Stockholm SE-171 77, Sweden; § Western Human Nutrition Research Center, Agricultural Research Service, USDA, Davis, California 95616, United States; ∥ Department of Pharmacology, Vanderbilt University, Nashville, Tennessee 37232, United States; ⊥ Department of Respiratory Medicine and Allergy, Karolinska University Hospital, Stockholm SE-141-86, Sweden; # Department of Nutrition, University of California, Davis, Davis, California 95616, United States; ∇ West Coast Metabolomics Center, Genome Center, University of California, Davis, Davis, California 95616, United States

## Abstract

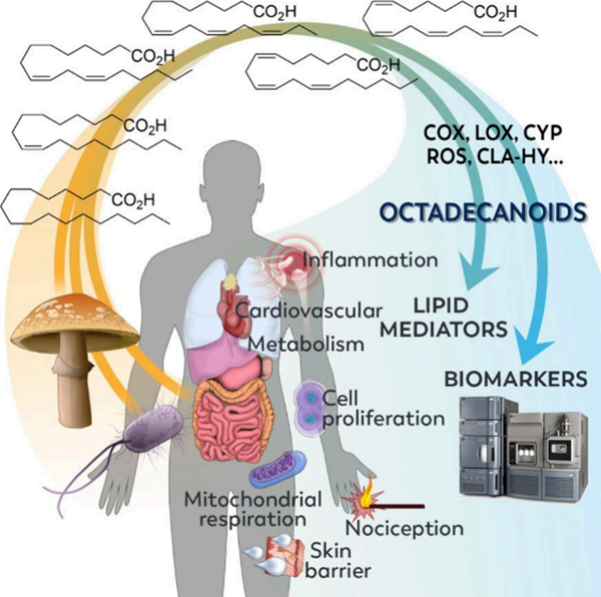

The octadecanoids are a broad class of lipids consisting of the oxygenated products of 18-carbon fatty acids. Originally referring to production of the phytohormone jasmonic acid, the octadecanoid pathway has been expanded to include products of all 18-carbon fatty acids. Octadecanoids are formed biosynthetically in mammals via cyclooxygenase (COX), lipoxygenase (LOX), and cytochrome P450 (CYP) activity, as well as nonenzymatically by photo- and autoxidation mechanisms. While octadecanoids are well-known mediators in plants, their role in the regulation of mammalian biological processes has been generally neglected. However, there have been significant advancements in recognizing the importance of these compounds in mammals and their involvement in the mediation of inflammation, nociception, and cell proliferation, as well as in immuno- and tissue modulation, coagulation processes, hormone regulation, and skin barrier formation. More recently, the gut microbiome has been shown to be a significant source of octadecanoid biosynthesis, providing additional biosynthetic routes including hydratase activity (e.g., CLA-HY, FA-HY1, FA-HY2). In this review, we summarize the current field of octadecanoids, propose standardized nomenclature, provide details of octadecanoid preparation and measurement, summarize the phase-I metabolic pathway of octadecanoid formation in mammals, bacteria, and fungi, and describe their biological activity in relation to mammalian pathophysiology as well as their potential use as biomarkers of health and disease.

## 1 Introduction

### 1.1 Fatty Acid Biosynthesis

**Scheme 1:**
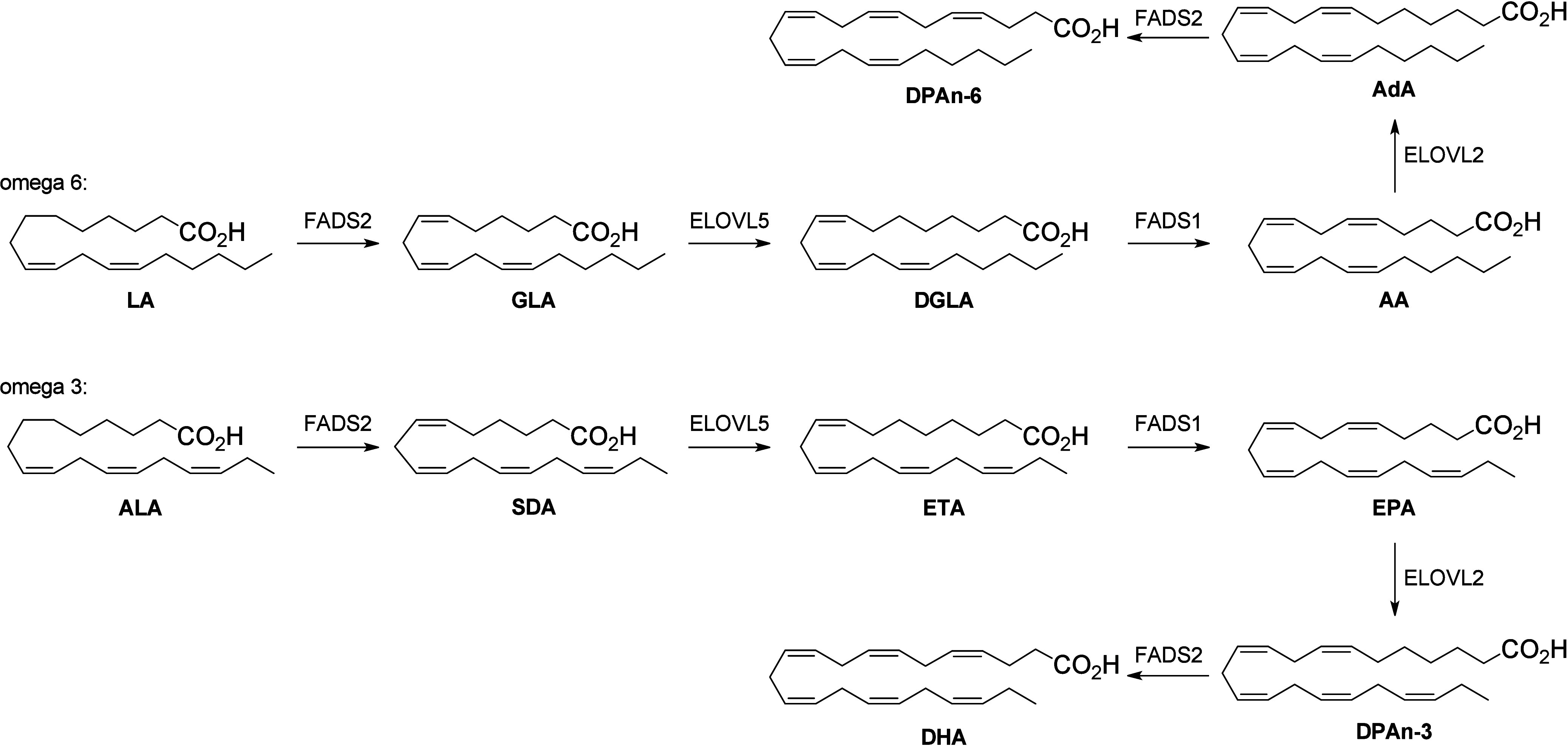
Formation of PUFAs from the Essential Fatty Acids Linoleic Acid (LA) and α-Linolenic Acid (ALA) via the Action of Desaturases and Elongases FADS = fatty acid desaturase. ELOVL = elongase. PUFA nomenclature is as in the text.

Fatty acids were described by Michel Eugène Chevreul in 1813.^1^ They have since been demonstrated to be important sources of energy and membrane constituents as well as act as signaling molecules that are involved in multiple biological activities including intracellular signaling, regulation of transcription, protein modification and other cellular processes.^2−4^ The early work of George and Mildred Burr established that the C18 polyunsaturated long-chain fatty acids (PUFAs) linoleic acid (LA, 18:2 ω6) and α-linolenic acid (ALA, 18:3 ω3) are dietary essential fatty acids (EFAs)^5^ because humans and most mammals lack the desaturase enzymes that can introduce an ω6 (n-6) or ω3 (n-3) double bond beyond carbons 9 and 10.^6^ Ralph Holman then formally established the ω6 and ω3 families of PUFAs in 1963.^7^ PUFA biochemistry has now been extensively mapped and we have a solid understanding of the enzymatic processes that form the full complement of these compounds *in vivo* (Scheme 1). Fatty acid biosynthesis involves a series of CH_2_ extensions and desaturations on the α-side of the fatty acid chain to convert EFAs to longer chain PUFAs.^8^ Both LA and ALA are converted by desaturases (fatty acid desaturases, FADS) and elongases (elongation of very long chain fatty acids, ELOVL) into downstream PUFAs. γ-linolenic acid (GLA, 18:3 ω6) and stearidonic acid (SDA, 18:4 ω3) are formed from LA and ALA, respectively, via the action of FADS2, and further converted into C20-PUFAs (dihomo-γ-linolenic acid (DGLA) and eicosatetraenoic acid (ETA), respectively) by ELOVL5. The enzyme FADS1 then desaturates these species to form arachidonic acid (AA) and eicosapentaenoic acid (EPA), which can then be converted into C22-PUFAs (adrenic acid (AdA) and docosapentaenoic acid (DPAn-3)) by ELOVL2. Finally, FADS2 activity forms DPAn-6 and docosahexaenoic acid (DHA), respectively.^9^ As our knowledge of the fatty acid biosynthesis pathways has increased, so has our understanding of their importance in biochemistry, physiology and health, with dysregulation in fatty acid biosynthesis associated with multiple pathologies.^10^

### 1.2 Oxylipins

The term oxylipin was defined by Hamberg and colleagues in 1991 to constitute a large group of oxygenated lipids formed from fatty acids by reaction(s) involving at least one step of mono- or dioxygenase-catalyzed oxygenation.^11^ The term was introduced to address the need to describe oxygenated lipids produced from fatty acids besides arachidonic acid (i.e., noneicosanoids). This definition has since been expanded to generally encompass all fatty acid-derived oxygenated lipids including products of enzymatic and nonenzymatic free radical-catalyzed oxidation and nonradical photosensitized oxidation of monounsaturated fatty acids (MUFAs) and PUFAs.^12^ Selected oxylipins exert potent mediator functions in a multitude of biological processes, including inflammation, immune activation, cellular development, ion transport, airway smooth muscle contraction, and thrombosis.^13−16^ These lipid mediators can also play a pivotal role in numerous diseases, including diabetes,^17^ obesity,^18^ cancer,^19,20^ pulmonary,^21^ psychiatric,^22^ metabolic,^23,24^ cardiovascular,^25^ and autoimmune diseases.^26^

Oxylipins can be classified according to the number of carbon atoms in the backbone of the fatty acid from which they are produced. In mammalian systems, the most well studied oxylipins are the eicosanoids produced from C20-PUFAs including the ω6 fatty acids AA and DGLA, the ω3 fatty acid EPA, and the ω9 fatty acid Mead acid (MA).^27,28^ More recently, the docosanoids produced from C22-PUFAs, including the ω6 fatty acid AdA and the ω3 fatty acids DPAn-3 and DHA, have been investigated.^29,30^ The oxygenation of C20-fatty acids from both ω6 and ω3 PUFAs produces a wide array of eicosanoid lipid mediators, including the well-studied prostaglandins (and their autoxidation analogues, the isoprostanes), thromboxanes, hydroxy-eicosatetraenoic acids (HETEs) and leukotrienes.^31^ The 2-series prostaglandins are produced from AA (e.g., PGD_2_, TxB_2_); however, analogous series can be produced from other C20-PUFAs including DGLA (e.g., PGD_1_, TxB_1_) and EPA (e.g., PGD_3_, TxB_3_). The critical role of eicosanoids in the mediation of a plethora of biological processes has been thoroughly studied and reviewed in the literature^31−34^ and will not be further discussed. Analogously, the oxygenation of long chain ω3 PUFAs produces the docosanoids including the resolvins, maresins and protectins, collectively referred to as specialized pro-resolving mediators (SPMs).^35^ The growing interest in SPMs has resulted in several recent reviews of their synthesis and biological activity, and interested readers are referred to those publications.^12,36−39^ There is increasing interest in this important class of compounds and the biosynthesis of nonmammalian oxylipins has been reviewed in detail.^40^ Collectively, oxylipins constitute a broad class of lipids that include multiple potent mediators of fundamental biological processes, demonstrating the importance of investigating these molecules.^41^

### 1.3 Octadecanoids

**Scheme 2:**
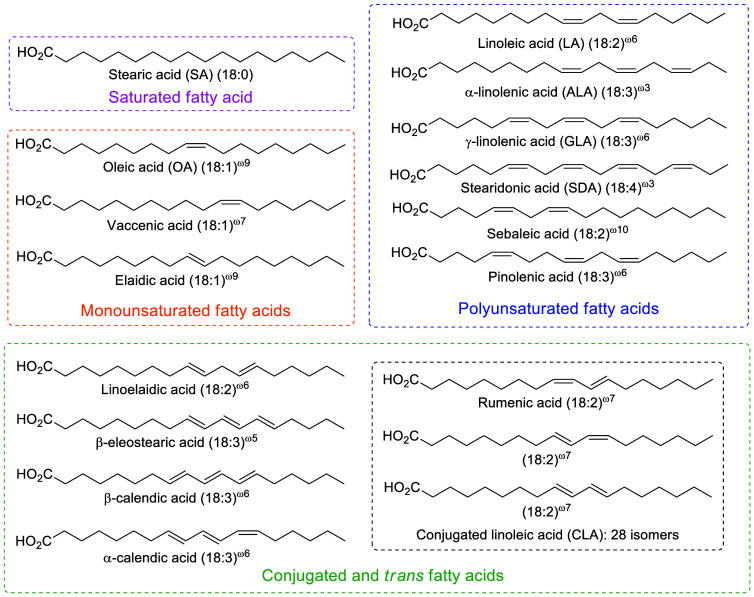
Examples of 18-Carbon Fatty Acids

The term octadecanoid refers to the oxygenated products of C18-FAs (including saturated FAs, MUFAs and PUFAs, Scheme 2).^32^ While the canonical use of the term octadecanoid has referred to the products of the jasmonate pathway,^42−44^ the definition can be broadened to be employed in the same fashion as for the eicosanoids and docosanoids. Many C18-FAs are present in the diet, resulting in high endogenous concentrations. The C18-PUFAs are found in high concentrations in triglyceride pools, cholesteryl esters, and membrane phospholipids^36^ to which they impart important physicochemical properties.^45,46^ A significant amount of C18-PUFAs are released from the membranes by phospholipases^47−49^ and are oxidized by the same enzymatic systems responsible for the formation of eicosanoids and docosanoids to produce the octadecanoids. It should be highlighted that phospholipase activity releases fatty acids from the nuclear and mitochondrial membranes.^50^ Given the high abundance of C18-FAs in the human diet,^51^ constitutive levels of many octadecanoids are generally higher than the eicosanoids and docosanoids, with LA- and ALA-derived octadecanoids constituting over 50% of the oxylipins present in tissues.^32^ However, the role of octadecanoids in human pathophysiology has not been closely investigated. The vast majority of studies related to octadecanoid oxylipins have focused on their role as phytohormones in plant systems, where they occur ubiquitously and regulate biotic and abiotic stress signaling, as well as plant growth and developmental processes, especially *via* the jasmonate pathway.^52^ The formation and function of octadecanoids in plants have been extensively studied, with a number of thorough reviews and will not be further discussed here.^52−54^

Only a few canonical octadecanoids have been investigated in-depth in humans, primarily the monohydroxy products, epoxides and diols produced from LA. In the 1980s, the pioneering work of Ozawa and colleagues identified potent toxic effects exerted by LA-derived epoxides and diols, linking them to the progression and severity of acute respiratory distress syndrome (ARDS).^55^ For these reasons, and because the epoxides are synthesized by leukocytes, the 9(10)- and 12(13)-epoxide regioisomers derived from LA were named leukotoxin and isoleukotoxin, respectively. These efforts were continued by Hammock and colleagues, who have extensively investigated the biology of the LA-derived epoxides and corresponding vicinal diols.^56−58^ The discovery of the potent bioactivity of these compounds sourced growing interest in LA-derived octadecanoids. In recent years, an increasing number of studies have started to expand our knowledge of how octadecanoids impact human pathophysiology, highlighting their ability to interact with various receptors and their involvement in the correct formation of the skin-water barrier,^59^ pain mediation,^60−62^ thermogenesis and heat regulation, cell proliferation,^65,66^ as well as in inflammation^67−69^ and immunomodulation.^70,71^

### 1.4 Scope

Given the lack of systematic reviews dedicated to octadecanoids outside of plant systems, the purpose of this review is to provide a detailed compilation of the primary octadecanoids formed from C18-FAs and their biosynthetic mechanisms, and to present a general overview of their biological functions as well as their suitability as biomarkers of diseases. In addition, an overview of the standard synthetic methods for preparation of octadecanoids is provided as well as a summary of the analytical methods used to measure octadecanoids. There is divergent terminology in the literature used for octadecanoids (e.g., terms like OXLAMs and oxiOMEs have been used to describe subclasses of LA metabolites)^61^ and a portion of this review addresses the need to standardize the nomenclature with a unifying principle applied to both oxygenated and nitrated fatty acids. The primary focus is on octadecanoids produced from oleic acid (OA), LA, ALA, GLA, and SDA. While octadecapentaenoic acid (ODPA) can be observed in some alga (e.g., dinoflagellates),^72^ it is generally not observed in mammals and ODPA-derived octadecanoids will not be discussed here. In addition, we discuss the conjugated linoleic acid (CLA) and conjugated linolenic (CLnA)-derived nitro-FAs. The less studied octadecanoids produced from C18-FAs possessing conjugated and/or *trans*-double bonds (e.g., conjugated linoleic acid) as well as minor C18-FAs (e.g., sebaleic acid, the major PUFA in human sebum and skin surface lipids) are discussed if the literature is available (Scheme 2). This review focuses on phase-I mammalian metabolism and includes bacterial and fungal metabolites produced by the symbiotic human microbiome. Nonenzymatic production of octadecanoids is considered independently from the organisms in which it takes place. Nonenzymatic oxidation by reactive oxygen species (ROS) and reactive nitrogen species (RNS) are discussed here; however, they are also broadly reviewed elsewhere.^73−76^

## 2 Proposed Octadecanoid Nomenclature

**Scheme 3:**
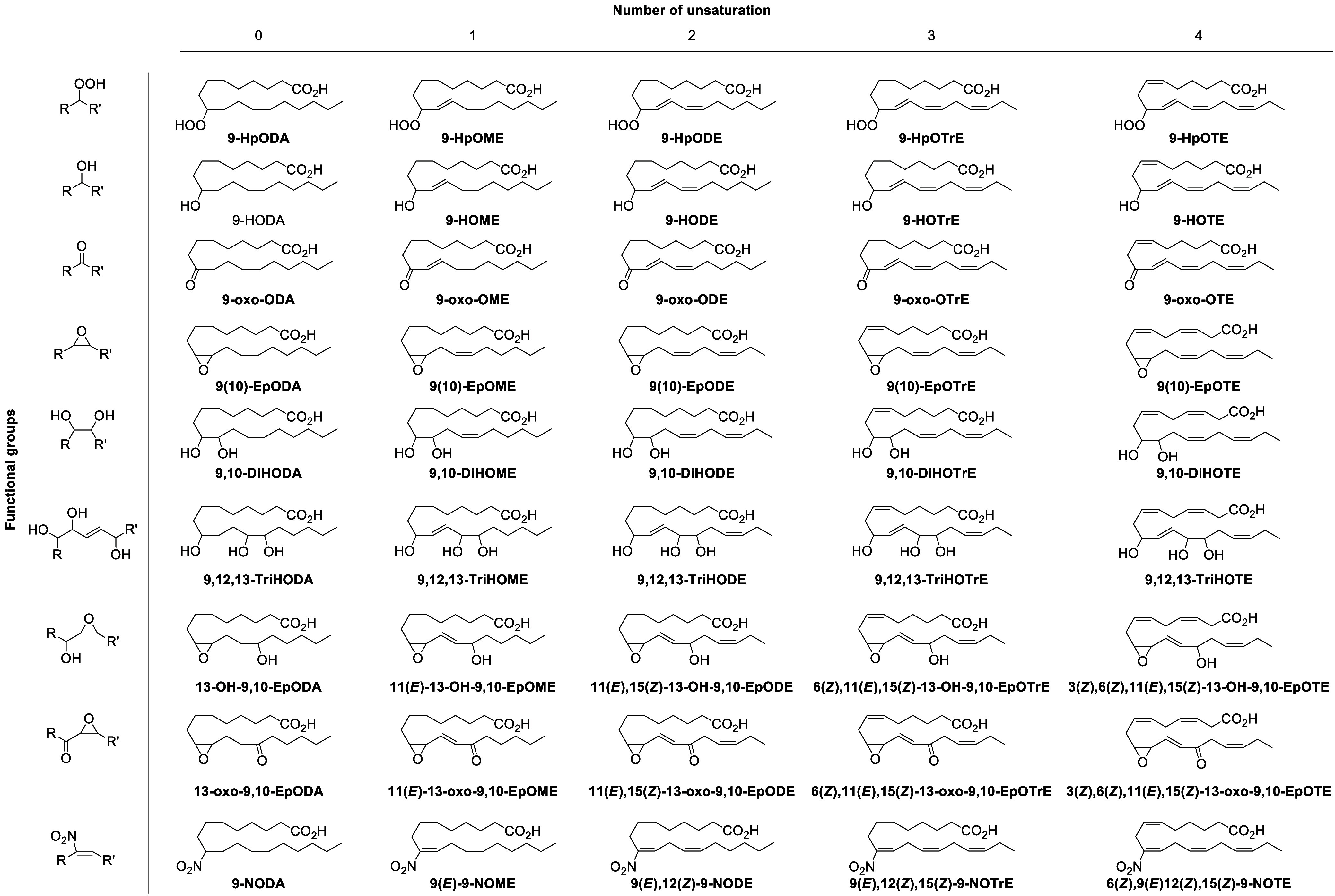
Proposed Octadecanoid Nomenclature System

There is currently no agreed common nomenclature for octadecanoids, with nonstandardized abbreviations often used to describe the same compound (e.g., 12(*Z*)-10-HOME,^77^ 10-OHODA,^78^ or HYA^79^ are used to name 10-hydroxy-12(*Z*)-octadecenoic acid, and 9-oxo-OTrE,^80^ 9-oxo-OTA,^81^ and 9-oxo-OTE^82^ are used to designate 9-oxo-10(*E*),12(*Z*),15(*Z*)-octadecatrienoic acid). This unclarity complicates the literature as well as proposes challenges for systematic lipid naming and curation approaches (e.g., LIPID MAPS,^83,84^ HMDB^85^). We therefore propose a system of nomenclature that is based upon the IUPAC system used for linear eicosanoids, which is well established and broadly used in the literature.^86^ This nomenclature was first proposed by our group in a previous paper,^77^ and it will be expanded and further developed here. The name of each compound starts by numbering the position of the oxygenated function carried by the carbon chain. If relevant (i.e., in the case of enantiopure compounds), the chirality of the carbon carrying the oxygenated function is indicated just after the position number of the function. The number, followed by chirality indication if appropriate, is separated by a dash from the abbreviation of the oxygenated function: “Hp”, hydroperoxy; “H”, monohydroxy; “DiH”, dihydroxy; “TriH”, trihydroxy; “oxo”, ketone; “Ep”, epoxide; “DiEp”, diepoxide, “N”, nitro. This prefix is followed by a capital “O”, indicating that these compounds have an 18-carbon backbone (i.e., octadecanoid), akin to the use of “E” for the 20-carbon backbone (i.e., eicosanoid). Finally, the number of unsaturations (i.e., “enes”) in the carbon chain is indicated: “ME”, monoene; “DE”, dienes; “TrE”, trienes; “TE”, tetraenes. For saturated metabolites, the suffix “DA” is used in the abbreviation to indicate that the compound is saturated (e.g., 9-HODA, 9-**H**ydroxy**O**cta**D**ec**A**noic). In this system, **9-HOTrE** will be **9-H**ydroxy-**O**ctadeca**Tr**i**E**noic acid, and **9,10-DiHODA** will be **9,10-DiH**ydroxy **O**cta**D**ec**A**noic acid (Scheme 3). Of note, the prefix “oxo” is recommended for ketone functionality, which is in accordance with IUPAC nomenclature. While the letter “K” has been used extensively (e.g., 9-KODE, 9-KOTrE), “oxo” is the correct terminology (e.g., 9-oxo-ODE, 9-oxo-OTrE).

**Scheme 4:**
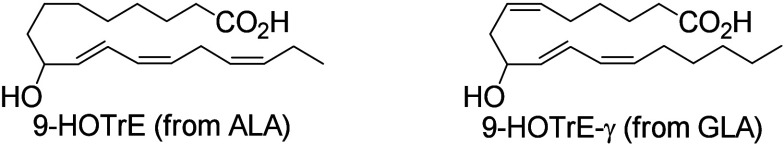
Nomenclature for α-Linolenic Acid (ALA)- and γ-Linolenic Acid (GLA)-Derived Octadecanoids

Isomeric octadecanoids derived from ALA and GLA, which only differ in the position of the double bonds (9(*Z*),12(*Z*),15(*Z*) and 6(*Z*),12(*Z*),15(*Z*) (for ALA and GLA, respectively), are discriminated by the addition of a suffix after their name. Given the lower abundance and minor number of reported octadecanoids produced by GLA, the suffix γ will be expressed only for GLA-octadecanoids. Scheme 4 shows an example for the metabolites produced by hydroxylation of C-9 in ALA and GLA.

**Scheme 5:**
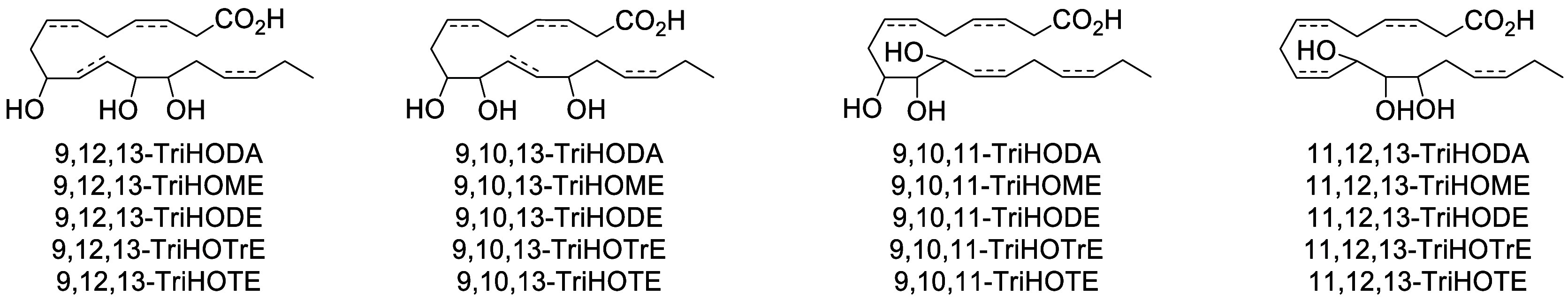
Nomenclature for Triol-Based Octadecanoids The dashed lines indicate potential position of unsaturations that would need to be explicitly indicated in the naming scheme.

This nomenclature does not indicate the position of the double bonds, and several isomers possessing different double bond configuration or position would be named in the same fashion. To address this issue, we propose to use this system of nomenclature for the products generated by canonical *bis*-allylic attack of the 1,4-pentadiene (i.e., giving products at the first or fifth positions), and to explicitly indicate the position of double bonds for any other metabolites (e.g., 10(*E*),12(*Z*)-9-HODE would be named simply 9-HODE, because it results from the *bis*-allylic attack of the 1,4-pentadiene of LA), whereas the position of the unsaturations is indicated for any other isomer. For example, 10-hydroxy-9(*Z*),12(*Z*)-octadecadienoic acid would be expressed as 9(*Z*),12(*Z*)-10-HODE. For *cis*-epoxides and diols, the positions of the double bonds can be neglected for the products of epoxidation of a *cis*-double bond of the parent fatty acid (and for the diol resulting from the hydrolysis of these epoxides). Thus, 9(10)-EpOME and 9,10-DiHOME are used only for 12(*Z*)-9(10)-EpOME and 12(*Z*)-9,10-DiHOME respectively, while the position of the double bonds is indicated for any other isomers. For the triols, this system of nomenclature is used for the products generated by *bis*-allylic attack followed by a rearrangement described in Scheme 12. Since the mechanisms of formation of triols identified so far only produce compounds with unsaturations as described in Scheme 5, the nomenclature will encompass most triols identified to date. For other regioisomers, the position of the double bonds is indicated (e.g., 9(*Z*)-12,13,17-TriHOME). If new metabolites with a different system of unsaturations are discovered, the positions will have to be specified in the name.

**Scheme 6:**
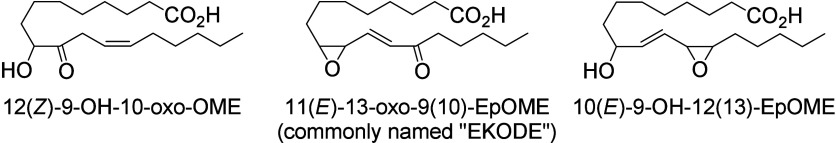
Nomenclature for Octadecanoids Possessing Two Functional Groups Nomenclature is as described in Scheme 3.

For metabolites possessing multiple oxygenated moieties, the functions are defined in the following order: hydroperoxys > hydroxys > ketones > epoxides > nitro. The position of the first function is indicated, followed by a dash and then indication of the nature of the function: “Hp” for hydroperoxides “OH” for hydroxys, “DiOH” for diols, “TriOH” for triols, “oxo” for ketones, “Ep” for epoxides and “N” for nitro. Then a dash is added before indicating the position of the next function (e.g., 12(*Z*)-9-OH-10-oxo-OME, 11(*E*)-13-oxo-9(10)-EpOME, 10(*E*)-9-OH-12(13)-EpOME: see Scheme 6). The position of the double bonds is always indicated for metabolites presenting multiple functions.

This system of nomenclature is not novel, is currently used by multiple research groups in the oxylipin field and is based upon the accepted IUPAC system. The necessity to define it unequivocally arises from the common use of ambiguous abbreviations that can result in unclear identification of compounds. A relevant example is the well-known octadecanoid EKODE, which is a nonenzymatic keto-epoxy metabolite of LA. EKODE is abbreviated as a compound possessing two unsaturations when it is in reality a monounsaturated metabolite. In our proposed system, this compound would be named oxo-EpOME, as illustrated in Scheme 6. As interest in octadecanoids continues to increase, it is important for the field that a common nomenclature system is used when reporting structures. This approach will greatly enhance the ability of lipid curation databases such as LIPID MAPS to include these compounds. The nomenclature adopted in this review for the phytoprostane^87,88^ and phytofuran^88,89^ species is well established and follows the rules determined by Taber and colleagues for isoprostane species.

## 3 Sources of Octadecanoids

### 3.1 Metabolism Overview

**Scheme 7:**
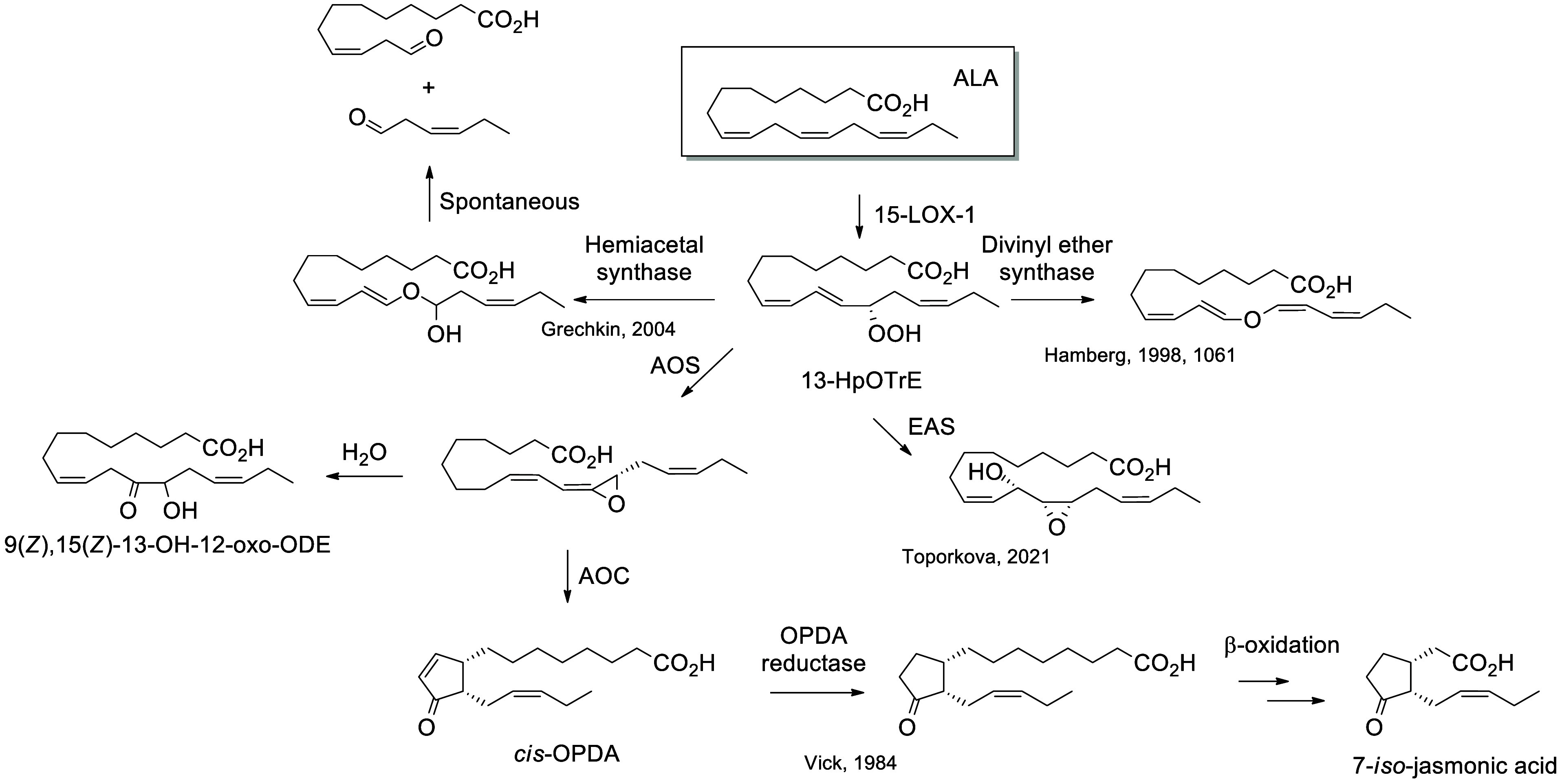
Biosynthetic Pathways for the Oxidation of α-Linolenic Acid (ALA) in Plants This pathway includes the canonical octadecanoid pathway, which leads to the production of the phytohormone jasmonic acid. The synthetic route is referenced if known.

In plants, there are four main biosynthetic pathways for PUFA oxidation: the CYP74 enzymes, including allene oxide synthases (AOS),^90^ hydroperoxide lyases (hemiacetal synthases),^91^ divinyl ether synthases,^92^ and epoxy-alcohol synthases (EAS).^93^ First, ALA is oxidized by 15-LOX-1 to form a hydroperoxide on the 13-position (13-HpOTrE), which can be rearranged by divinyl ether synthase to form an ether^92^ or by hemiacetal synthase to form a hemiacetal that is spontaneously cleaved into 9(*Z*)-12-oxo-9-dodecenoic acid and 3(*Z*)-hexenal.^91^ EAS transforms 13-HpOTrE into 11-OH-12(13)-EpODE.^93^ Finally, AOS transforms the hydroperoxide into 9(*Z*),11(*E*),15(*Z*)-12(13)-EpOTrE. This transformation is the first step of the jasmonate pathway, originally described as the “octadecanoid pathway”, which produces the phytohormone jasmonic acid. The epoxide is then cyclized by allene oxide cyclase (AOC) into *cis*-OPDA. The intracyclic double bond is reduced by OPDA reductase and several steps of β-oxidation to give 7-*iso*-jasmonic acid.^90^ Finally, 9(*Z*),11(*Z*),15(*Z*)-12(13)-EpOTrE can spontaneously rearrange into 9(*Z*),15(*Z*)-13-OH-12-oxo-ODE (Scheme 7).

Plants, like animals, produce monooxygenated products (hydroxy and epoxy acids) as well as desaturated products and products possessing conjugated double bonds. Well-studied conversions here are those catalyzed by various nonheme di-iron enzymes.^94^ Action by such enzymes results in the formation of ricinoleic acid, crepenynic acid, and vernolic acid as well as calendic acid and other conjugated fatty acids present in certain seed oils. These products occur in plants only, and to the best of our knowledge there are no parallel pathways in animal tissue. Dimorphecolic acid, a plant-specific product related to 9-HODE, is also produced by a member of this group of enzymes. However, again there is no overlap between plant and animal products since in dimorphecolic acid the double bonds have the 10(*E*),12(*E*) configuration whereas they are 10(*E*),12(*Z*) in the animal lipoxygenase product 9-HODE.

In mammals, the main enzymes responsible for the formation of eicosanoids and docosanoids, namely lipoxygenases (LOX), cyclooxygenases (COX), and cytochrome P450s (CYP), also utilize C18-FAs as substrates.^95−98^ The affinity of these enzymes for C18-FAs is generally lower compared to their longer chain analogues; however, lower octadecanoid production can be compensated by the higher substrate abundance.^99^ Enzymatic oxidation is performed with determined stereospecificity, where the produced stereoisomers depend on the specific enzyme and substrate. The octadecanoids produced by enzymatic biosynthesis include alcohols, diols, triols, ketones, and epoxides, or combinations of these functional groups (e.g., hydroxy-epoxides and keto-epoxides). The introduction of multiple functional groups in C18-FA metabolism is generally the result of sequential metabolism. For example, the ALOXE3 gene in human skin encodes epidermal lipoxygenase 3 (eLOX3), which possesses hydroperoxide isomerase activity that is able to transform 12(*R*)-LOX-dependent LA metabolites to the corresponding hydroxy-epoxides.^100^ Similarly, the 12-LOX-derived ALA C-9 monohydroxy products can be further targeted by 15-LOX to produce a nonvicinal diol.^101^ Nonenzymatic conversion is effected by radical or small reactive molecules such as singlet oxygen. As opposed to enzymatic oxidation, the stereochemistry of these processes is not controlled and nonenzymatic octadecanoids are produced as racemic mixtures. In addition to the same linear structures described for the enzymatic oxidation, nonenzymatic oxidation can produce cyclic compounds like endoperoxides and, for PUFAs possessing at least three double bonds, compounds with 5-atom rings (e.g., phytoprostanes and phytofurans^102,103^). Finally, the interaction of conjugated octadecanoids with nitric oxide can yield nitro fatty acids (NO_2_-FAs) in both plant and animal systems.^104,105^ To our knowledge, fungal NO_2_-FA formation has not been reported. The catabolism of octadecanoids is similar to other oxylipins and fatty acids in general, with a combination of initial β-oxidation in conjunction with further rounds of β- and/or ω-oxidation.^41,106^ Mitochondrial β-oxidation has been proposed to be a major metabolic regulatory checkpoint for oxylipins during inflammation.^107^ However, these pathways have yet to be explicitly explored for the octadecanoids.

### 3.2 Dietary Octadecanoids

#### 3.2.1 Dietary Intake of C18-PUFAs

LA constitutes ∼7% of the total energy uptake in the United States,^45^ and is now the most highly consumed PUFA in the Western Diet.^108^ LA is readily available from vegetable oils (e.g., sunflower, safflower, soybean, corn, and canola) as well as nuts and seeds.^108^ Worldwide, there has been a large and rapid increase in the amount of LA consumed in response to the work of Ancel Keys, who recommended a dietary shift from saturated to unsaturated fat for cardiovascular health.^109^ In particular, global diets have become high in soybean and corn oils. The health effects associated with consumption of LA are unclear and context dependent,^110−113^ and can be associated with genotype.^114^ The effects of LA supplementation upon octadecanoid levels are also ambiguous. In some studies, dietary levels of LA have been reported to associate with observed octadecanoid levels, for example, increased dietary LA resulted in higher observed LA-derived octadecanoid levels in the brain,^115^ while lowering dietary LA resulted in lower plasma levels of 9- and 13-HODE (as well as 9- and 13-oxo-ODE).^116^ However, this relationship is not always clear, for example feeding mice a diet high in LA-derived octadecanoids did not affect the levels observed in liver.^117,118^ The literature suggests that while dietary levels of parent PUFAs can affect observed octadecanoid levels, supplementation with octadecanoids directly does not. There are a number of reviews on this relationship for further reading.^119,120^

**Scheme 8:**
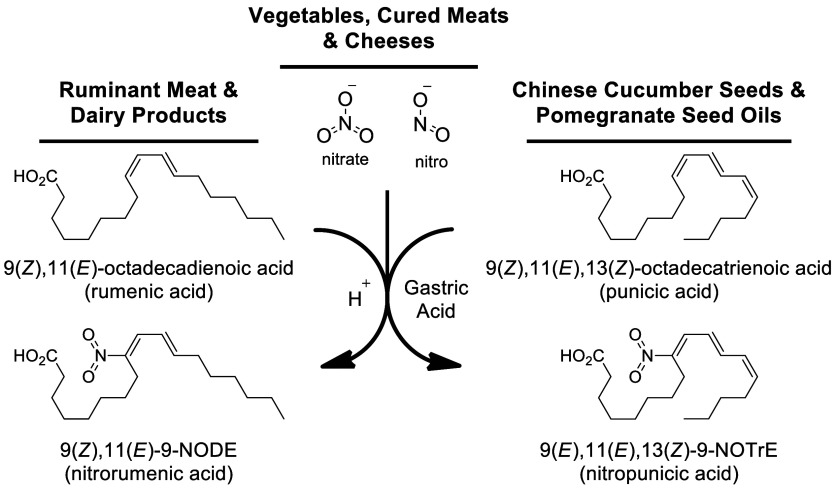
Dietary Nitrate/Nitrite Can Combine with Conjugated Fatty Acids to Form Nitrated Fatty Acids (NO_2_-FAs) in the Acidic Environments of the Stomach

While numerous studies cite the conversion of LA into AA (the precursor of “pro-inflammatory” eicosanoids) to be a pro-inflammatory process, the biochemical reality is more nuanced.^108,121^ LA exerts biochemical effects in its own right,^122,123^ and the bioactivity of eicosanoids is species and context dependent, including both pro-inflammatory and pro-resolving (e.g., lipoxins) actions.^121^ ALA is readily available in vegetable oils (e.g., soybean, canola, flaxseed, and perilla) as well as seeds and nuts (particularly walnuts) and other plant food sources.^124^ Dietary ALA is generally associated with beneficial health effects,^125^ including that of the Mediterranean diet,^126^ and is associated with a reduced risk in all causes mortality.^112,127^ However, concerns have also been expressed about associations with cancer.^127^ In terms of other C18-PUFAs, the dietary levels are generally low and they are primarily consumed as supplements including GLA (e.g., borage seed oil, evening primrose oil, black currant, ahiflower, spirulina) and SDA (e.g., black currant, ahiflower, spirulina). Recent work has shown that SDA supplementation with ahiflower oil increases the circulating levels of EPA and EPA-derived oxylipins, but does not affect DHA levels.^128^ CLAs are the most abundant conjugated PUFAs in the food supply, primarily present in foods derived from ruminants.^129^ The less common CLnAs are found in various plant seed oils.^130,131^ CLnAs are also present in some foods, with 9(*Z*),11(*E*),13(*Z*)-octadecatrienoic acid (i.e., punicic acid) being found at up to 70% in pomegranate seed oil.^130^ Moreover, the edible seeds of the Chinese cucumber, *Trichosanthes kirilowii*, can also provide a dietary source of CLnAs (Scheme 8).^132^ This rare oil is currently under investigation as a nutraceutical.^133^ The health effects associated with dietary PUFA consumption have been extensively studied and consist of an ongoing topic of investigation and will not be further discussed here. Interested readers are directed to several in-depth reviews.^134−138^

#### 3.2.2 Dietary Sources of Octadecanoids

Multiple octadecanoids are present in the diet from both plant and animal sources,^32,139−144^ which constitutes a significant exogenous source of octadecanoid exposure. For example, in 2017 Mubiru et al. documented the C18-epoxy-FA levels in triglycerides present in 390 foods available in the Belgian market.^145^ They reported levels between 12 and 687 mg/kg of total C18-epoxy-FAs with animal source foods, being substantially lower than those from plants. More recently, Koch et al. measured the oxylipin content of food oils and processed foods and reported high concentrations (up to 80 mg per serving) in fried falafel and processed foods such as vegetarian sausage/fish fingers.^146^ They proposed that the epoxy-to-diol ratio could be a potential marker for refined oils (e.g., the ratio of 15(16)-EpODE to 15,16-DiHODE), with diols increased in refined oils. In the same year, a study quantified the levels of parent PUFAs and of the main metabolites of LA and ALA in different plant and algae edible oils.^142^ The results highlighted a relationship between the levels of parent PUFA and the related oxylipins for all oils except olive and flaxseed oil, which had higher levels of oxylipins in relation to the fatty acids. The levels of oxylipins detected in the different oils were very heterogeneous and spanned several orders of magnitude. Flaxseed oil had the highest absolute levels of ketones and hydroxys from both LA and ALA, whereas canola oil had the lowest levels for HODEs and oxo-ODEs and olive oil for HOTrEs and oxo-OTrEs. Soybean oil was found to be rich in DiHOMEs and DiHODEs, while corn and flaxseed oil had the highest levels of EpOMEs and EpODEs, respectively. The median concentrations determined in this work are summarized in Table 1, expressed in μM as the median value obtained in the various oil samples.^142^

**Table 1:** Median Concentration Values of Octadecanoids Quantified in Commercial Edible Plant Oils (μM)^142^

octadecanoid^a^	parent PUFA	soybean oil	corn oil	canola oil	olive oil	flaxseed oil
13-HODE	LA	1.57	8.40	0.15	14.3	24.1
9-HODE	LA	0.33	5.45	0.22	14.0	68.1
13-oxo-ODE	LA	0.28	0.45	0.12	4.42	25.8
9-oxo-ODE	LA	0.89	2.32	0.68	10.3	2.88
12(13)-EpOME	LA	5.10	13.0	2.88	1.21	13.0
9(10)-EpOME	LA	4.43	17.3	2.55	0.25	9.20
12,13-DiHOME	LA	18.8	9.03	0.98	0.57	0.35
9,10-DiHOME	LA	23.9	15.4	0.36	0.76	0.44
9-HOTrE	ALA	0.99	0.23	0.42	2.09	50.8
13-HOTrE	ALA	0.16	0.42	0.95	0.91	19.3
15(16)-EpODE	ALA	3.59	2.43	3.84	0.22	249
12(13)-EpODE	ALA	0.57	0.21	0.85	0.85	15.1
9(10)-EpODE	ALA	0.74	1.01	1.03	0.18	20.7
15,16-DiHODE	ALA	28.0	1.53	11.2	0.15	7.99
12,13-DiHODE	ALA	0.72	0.19	0.10	0.14	0.381
9,10-DiHODE	ALA	9.16	1.72	0.15	0.70	0.31

a Compound nomenclature is as described in Scheme 3.

Our own preliminary investigations of octadecanoid levels in walnuts have revealed an array of octadecanoids with TriHOMEs > HODEs > oxo-OMEs > HOTEs > EpOMEs > EpODAs > EpODEs > DiHOMEs > DiHODAs > DiHODEs.^147^ In general, the most well studied aspect of oxygenated lipids in food is the impact of oxidation on edible oils^148,149^ and meat.^150^ While the incubation of cooked meat in gastric fluids can promote lipid peroxidation, coincubation with plant-based antioxidants suppressing this activity.^151^ Moreover using oxygenated stearates in simulated gastric conditions, Márques-Ruiz et al. demonstrated that ∼20–60% of 9(10)-EpODA was converted to 9,10-DiHODA, and ∼50% of 12-HpODA was degraded, while 12-HODA and 12-oxo-ODA were unaltered.^152^ Using deuterium labeled compounds, 13-HODE was shown to be absorbed after oral gavage, with an incorporation half-life of 71 min in the rat.^153^ The tracer was incorporated into multiple tissues, but not the brain. In rats, consumption of trilinoleoylglycerol hydroperoxide led to the formation of 13-HODE, oxo-EpOMEs, and aldehydes in the gut, with enterocyte absorption.^154^ Animal studies considering the impact of oxidized oil consumption reported decreases in liver and plasma triglycerides and cholesterol.^155^ Moreover, gastric cells exposed to 13-HODE increased the production of branched chain amino acids, suggesting a possible beneficial impact of oxidized oil consumption.^156^

In contrast to the distribution of oxygenated lipids in foods, for nitro lipids, only (*E*,*Z*)-NODEs have been reported as endogenous components of extra virgin olive oil.^157^ More typically, the acidic pH of gastric juices can promote conjugated linoleic and conjugated linolenic acid reactions with dietary nitrite (NO_2_^–^) to form bioactive nitro lipids *in vivo*.^158^ Nitrite is present in many vegetables, including spinach, lettuce and beets, and foods cured with nitrate salts, including many cured meats.^159^ Concurrent ingestion of conjugated fatty acids and nitrate increases conjugated NODE levels in plasma, urine and tissues,^160^ with demonstrated anti-inflammatory and anti-hypertensive effects.^161,162^ The putative health effects associated with dietary nitrate should also be considered within the context of the negative effects on nitrosative stress.^159^

Therefore, while some ingested octadecanoids in foods can be substantially altered in the gut, others are unaltered, and some can be formed during the digestion process, with substantial proportions available for host absorption. The potential impact of these exogenous sources of octadecanoids on host metabolism deserves further attention.

## 4 Enzymatic Biosynthesis of Octadecanoids

Octadecanoids can be produced from MUFAs and PUFAs by the action of different enzymes present in plants and mammals. Analogous to the formation of eicosanoids, the main enzymes known to oxidize C18-FAs are the cyclooxygenases, the lipoxygenases and the cytochrome P450s. These enzymes produce a large panel of alcohols, diols, triols, ketones and epoxides, as well as metabolites possessing a combination of these moieties. C18-FAs are generally poorer substrates for these enzymes compared to their C20 analogues; however, due to their high abundance, the amount of produced octadecanoids is significant. In addition, bacteria and fungi possess their own set of enzymes that can create unique octadecanoids, influencing the octadecanoid profile of the host.

### 4.1 Cyclooxygenases

Cyclooxygenase (COX) enzymes, also called prostaglandin–endoperoxide synthases and prostaglandin G/H synthases, are heme-containing dioxygenase enzymes that possess oxygenase and peroxidase activity on PUFAs. COX enzymes are homodimers and possess two isoforms (COX-1 and COX-2), each consisting of three distinct domains. On both isoforms, the catalytic domain is located on the C-terminal domain and contains separate oxygenase and peroxidase active sites on opposite sides of the heme cofactor. The N-terminal domain facilitates dimerization and membrane binding, while the third domain is important for membrane binding.^31^ Both oxygenase and peroxidase evidence slight differences in their activities and expression between the two isoforms. The two COX isoforms possess similar *V*_max_ and *K*_m_(95) but exhibit differences in expression, tissue distribution, allosteric regulation and substrate specificity. COX-1 is ubiquitously expressed, and its expression sites include blood vessels, prostate, immune cells (monocytes, T-cells), platelets, stomach, resident inflammatory cells, smooth muscles, and mesothelium of many organs.^163^ On the other hand, COX-2 is generally synthesized in response to inflammatory stimuli in many tissues including prostate, immune cells (T-cells, B-cells, monocytes), and stomach, but is also constitutively expressed in the brain, lungs, gut, thymus, kidneys, and blood vessels.^163−165^ AA is the main substrate of both isoforms, but LA is a known competitive substrate and inhibitor of prostaglandin formation by COX-1 and COX-2.^166^ To a lesser extent, ALA is oxidized by COX-2 and while GLA is a poor substrate of both isoforms, it is a better substrate for COX-2.^167^

#### 4.1.1 Enzymatic Mechanism

**Scheme 9:**
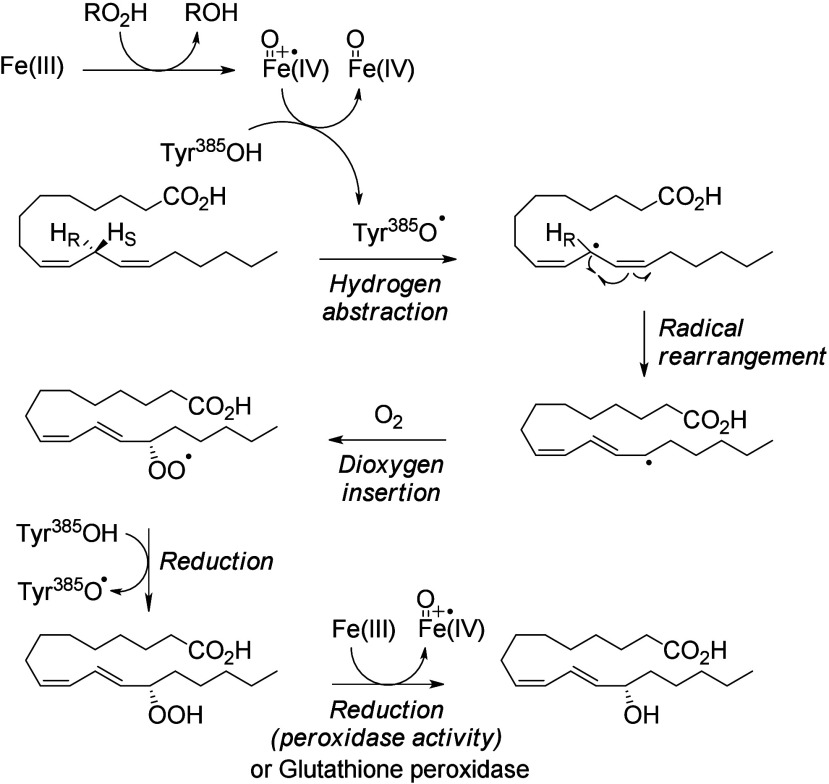
Cyclooxygenase (COX) Peroxidase Activity

The COX catalytic domain possesses a heme-contained iron. Fe(III) is converted into iron(IV)-oxoporphyrin-radical *via* the reduction of a fatty acid hydroperoxide or an organic hydroperoxide. The action of this oxoferryl porphyrin radical on tyrosine^385^ creates a tyrosyl radical that abstracts a *bis*-allylic hydrogen and forms a conjugated radical. A dioxygen molecule is trapped by this radical to form a peroxyl radical.^168^ In contrast to the formation of prostaglandins from C20-PUFAs, this step is not followed by cyclization and addition of a second dioxygen molecule on C18-PUFAs. Instead, the peroxyl is protonated to yield a hydroperoxide (lipoxygenase-type reaction) that is reduced into a hydroxy group by oxidation of Fe(III) included in the peroxidase active site or by other enzymes such as glutathione peroxidase (Scheme 9). Hydroxylated C18-PUFAs are generated *via* the same pathway as side products of prostaglandin biosynthesis from C20-PUFAs.^31^

#### 4.1.2 Cyclooxygenase-Derived Octadecanoids

**Scheme 10:**
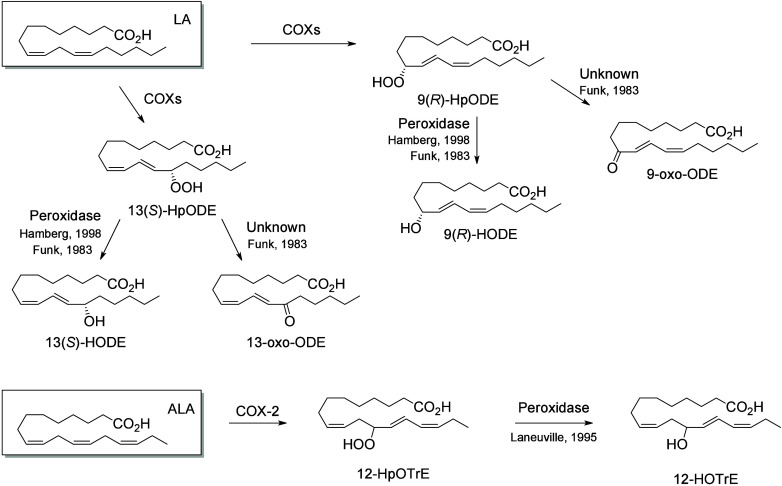
Linoleic Acid (LA) and α-Linolenic Acid (ALA)-Derived Octadecanoids Produced by Cyclooxygenases (COX) Nomenclature is as described in Scheme 3. The synthetic route is referenced if known.

The stereospecific removal of the LA 11-pro(*S*) hydrogen by COX enzymes forms two hydroperoxides, 13(*S*)-HpODE and 9(*R*)-HpODE, that are reduced into two main monohydroxylated products 13(*S*)-HODE and 9(*R*)-HODE (Scheme 10).^169^ The ratio of 9-HODE and 13-HODE vary depending on the enzyme source (tissue vs. recombinant) and the species. In ovine vesicular gland, COX-1 catalyzes preferentially the oxygenation of the 9-position of LA and the formation of 9- and 13-HODE in an 8:2 ratio. In contrast, recombinant human COX-1 expressed in COS-1 cells yielded 13-HODE and 9-HODE in a 5/1 ratio consuming ∼10% of the provided LA, while under the same conditions human COX-2 transformed ∼35% of LA with a 13-HODE/9-HODE ratio of 8/1.^95^ Both 9- and 13-HpODE can also undergo dehydration to produce the respective oxo-products 9-oxo-ODE and 13-oxo-ODE,^170^ or be converted nonenzymatically into hydroxy-epoxides or in the skin by a subsequent LOX reaction.^61^ These hydroxy-epoxides can subsequently be hydrolyzed to trihydroxy metabolites (TriHOMEs) *via* a nonenzymatic pathway or by epoxide hydrolases present in both particulate and cytosolic fractions.^171^ A TriHOME is a stable end product of the oxidation sequence. It has been shown that the formation of 9-HpODE, 13-HpODE, and TriHOMEs is suppressed by inhibition of COX with indomethacin or acetylsalicylic acid in calf aorta.^172^ Contrary to the other substrates that undergo a hydrogen abstraction on the ω8 position, the hydrogen abstraction occurs on the ω5 position of ALA, creating 12-HOTrE as the main product (Scheme 10).

### 4.2 Lipoxygenases

Lipoxygenases (LOX) are nonheme iron-containing dioxygenases that catalyze the conversion of PUFAs containing a 1,4-pentadiene system into conjugated hydroperoxy-fatty acids. The human genome contains 6 functional LOX genes (*ALOX5*, *ALOX12*, *ALOX12B*, *ALOX15*, *ALOX15B*, *ALOXE3*) encoding for 6 different LOX-isoforms: 5-LOX, 12(*S*)-LOX, 12(*R*)-LOX, 15-LOX-1, 15-LOX-2, and eLOX3. These isoforms differ by their expression in different mammalian tissues and by their activities (Table 2).

**Table 2:** Human Lipoxygenase (LOX) Tissue Expression and Substrate Preference^31,173^

human gene	name	major expression	substrates
ALOX15	15-LOX-1	eosinophils, bronchial epithelium, monocytes, macrophages, dendritic cells, reticulocytes	LA, GLA, ALA, AA, EPA, DHA
ALOX15B	15-LOX-2	hair roots, skin, prostate, macrophages, lung, cornea	GLA, ALA, AA, EPA, DHA (LA is a poor substrate)
ALOX12	12(*S*)-LOX	skin, platelets, umbilical vein endothelial cells, vascular smooth muscle cells	AA, DGLA, EPA, DHA
ALOX12B	12(*R*)-LOX	skin, hair roots, tonsil epithelial cells, bronchial epithelial cells	GLA, AA, DGLA (LA, EPA, and DHA are poor substrates)
ALOX5	5-LOX	leukocytes, macrophages, dendritic cell, mast cells, lung, placenta	ALA, AA, 5(*S*)-HpETE
ALOXE3	eLOX3	skin	9(*R*)-HpODE, 12(*R*)-HpETE

12(*S*)-LOX is primarily expressed in platelets, while 12(*R*)-LOX and eLOX3 are most frequently found in skin. 15-LOX-1 is highly expressed in leukocytes and airways endothelial cells whereas 15-LOX-2 is expressed in multiple tissues including skin, prostate, lung, cornea, liver, colon, kidney, brain, and monocytes-macrophages.^31,173,174^ Different LOX isoforms present varying affinities with octadecanoid PUFAs. For example, LA is the preferred octadecanoid substrate of 15-LOX-1, and an acceptable substrate for 15-LOX-2.^96^ The selectivity of LOX isoforms evidence species-specific variability. For example, the human 15-LOX-2 isoform produces 13(*S*)-HpODE while the murine gene *Alox15b* encodes a 8(*S*)-LOX enzyme that forms 9(*S*)-HpODE from LA.^173,175^

#### 4.2.1 Enzymatic Mechanism

**Scheme 11:**
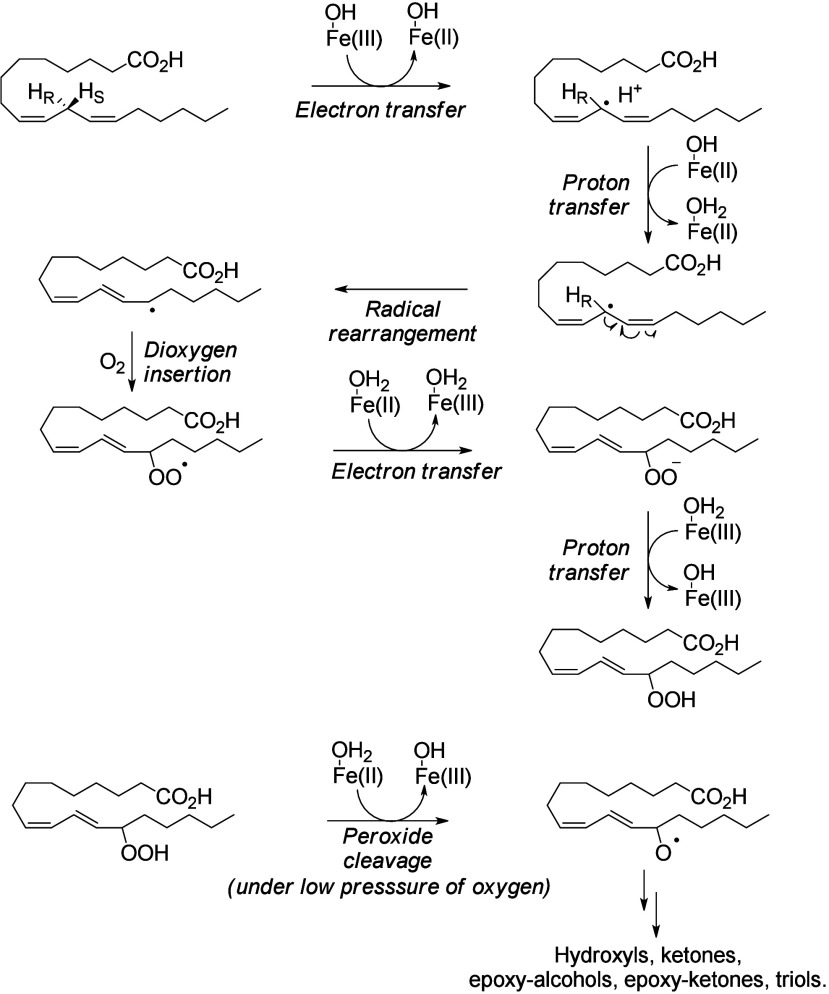
Lipoxygenase (LOX) Catalytic Mechanism

Nonheme ferric iron Fe(III) performs stereospecific hydrogen atom removal in a *bis*-allylic position via a mechanism involving proton-coupled electron transfer (PCET). First, an electron is transferred to Fe(III) to produce ferrous ion Fe(II), and the proton is simultaneously trapped by the iron’s hydroxide ligand. A radical rearrangement step creates a more stable conjugated diene. Then, the conjugated radical traps a dioxygen molecule to create a hydroperoxide after protonation via a second PCET mechanism that reforms Fe(III).^176^ Under low oxygen pressure conditions, the enzyme’s iron is in the ferrous state (Fe(II)) instead of staying in the ferric state (Fe(III)). Fe(II) can promote degradation of hydroperoxides and cause a Fenton reaction.^177−179^ In these conditions, the peroxyl can be cleaved by Fe(II) ions to create an alkoxyl radical. The formation of this alkoxyl radical is responsible for the production of various oxygenated metabolites (ketone, hydroxy-epoxide, keto-epoxide, triol, etc.) (Scheme 11).^31^

**Scheme 12:**
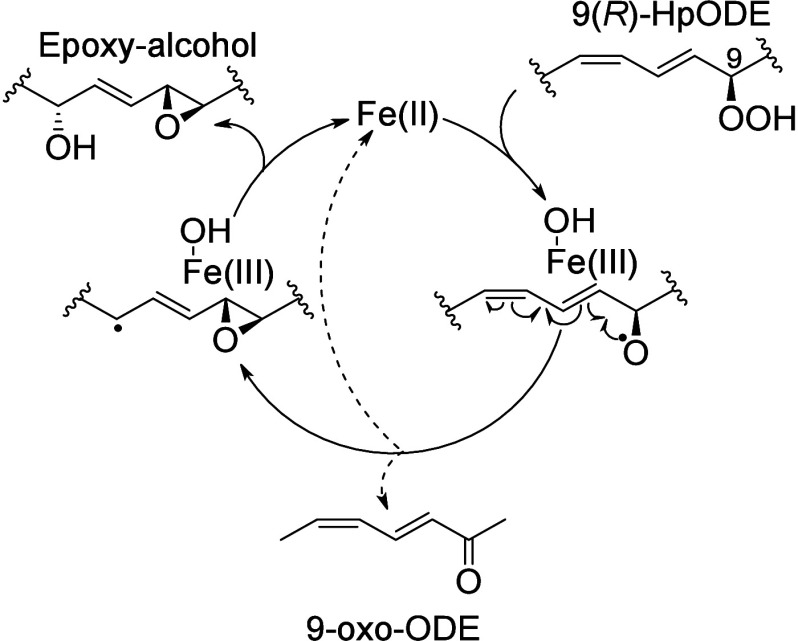
Epidermis-Type Lipoxygenase 3 (eLOX3) Catalytic Mechanism^100^

In the skin, eLOX3 exhibits a hydroperoxide isomerase activity that converts hydroperoxides to hydroxy-epoxides and ketones, but it does not have an oxygenase activity. In contrast to the classical Fe(II)/Fe(III) mechanism of LOX oxidation, where Fe(III) is the active form, the active species of eLOX3 oxidation is the ferrous ion. Fe(II) initiates a homolytic cleavage of the peroxide bond, creating an alkoxyl radical and a ferric ion possessing a hydroxide ligand. The alkoxyl radical cyclizes to form an epoxyallylic radical. Finally, a radical addition of the hydroxide included in the Fe(III)–OH complex forms a hydroxy-epoxide product and restores the active Fe(II) form. A ketone is formed as a side-product of the catalytic cycle (Scheme 12).^100^

#### 4.2.2 Lipoxygenase-Derived Octadecanoids

**Scheme 13:**
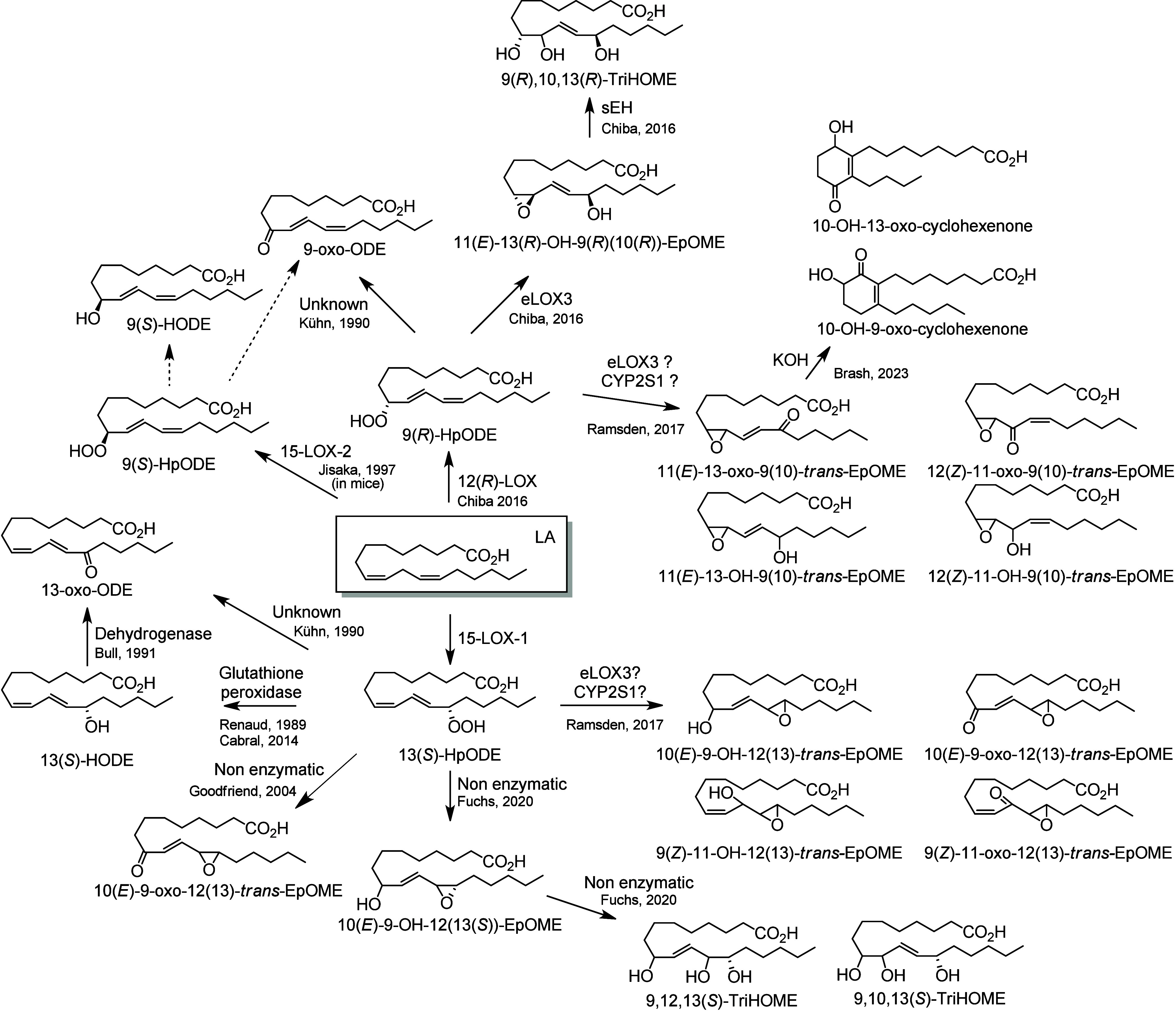
Linoleic Acid (LA)-Derived Octadecanoids Produced by Lipoxygenases (LOX) Nomenclature is as described in Scheme 3. Synthetic route is referenced if known or indicated with a question mark if unknown. The dotted arrows refer to the formation of compounds in mice for which a primary citation could not be identified in the literature.

In humans, 15-LOX-1 and 12(*R*)-LOX produce, respectively, 13(*S*)-HpODE^99^ and 9(*R*)-HpODE^180,181^ from LA. In mouse skin, 8(*S*)-LOX can convert LA into 9(*S*)-HpODE.^175^ These hydroperoxides can in turn be converted to hydroxys, ketones, hydroxy-epoxides, keto-epoxides, and triols (Scheme 13). Hydroperoxides can be reduced to hydroxy groups to produce 13(*S*)-HODE in human,^65,182^ and 9(*S*)-HODE in mice.^175^ The oxo-products 13-oxo-ODE and 9-oxo-ODE are produced from an alkoxyl radical derived from LOX-assisted hydroperoxide cleavage.^183^ 13-oxo-ODE can also be formed from 13-HODE by a NAD^+^ dependent dehydrogenase first identified in rat colon mucosa.^184^ The relative formation of the HODEs in mammals via LOX or COX activity is unclear and a topic that requires further investigation.

Hydroperoxide isomerization generates hydroxy-epoxides and keto-epoxides. In human skin, eight hydroxy-*trans*-epoxides and keto-*trans*-epoxides formed from 9(*R*)-HpODE and 13(*S*)-HpODE have been identified.^61^ The authors hypothesize that the enzymes eLOX3 and CYP2S1 are involved in the formation of these epoxy-metabolites. Isomerization of 9(*R*)-HpODE creates two hydroxy-epoxides possessing an epoxide at the 9(10)-position and a hydroxy at the 11- or 13-position, as well as the two corresponding ketones. From 13(*S*)-HpODE, two hydroxy-epoxides possessing an epoxide at the 12(13)-position and a hydroxy group at the 9- or 11-position are created, along with their corresponding ketones.^61^ This isomerization is performed nonenzymatically *via* the action of free radicals. Exposure of 13(*S*)-HpODE with FeCl_3_ and cysteine creates 10(*E*)-9-oxo-12(13)-*trans*-EpOME, which has been detected in human plasma.^185^ The hydroxy-epoxides can then be opened to generate triol metabolites, either by a nonenzymatic pathway as shown in eosinophils by Fuchs et al.^172^ or *via* the action of an epoxide hydrolase as suggested by Funk et al.^171^ It has been shown recently that alkaline treatment of the keto-epoxide 11(*E*)-13-oxo-9(*R*)(10(*R*))-*trans*-EpOME esterified to ceramides in skin form two cyclic metabolites, 10-OH-13-oxo-cyclohexenone and 10-OH-9-oxo-cyclohexenone.^186^

Trihydroxy derivatives are end products of C18-PUFA oxidation in mammals.^59^ 12(*R*)-LOX and eLOX3 are abundantly expressed in human skin. 9(*R*)-HpODE is isomerized by eLOX3 into 9(*R*),10,13(*R*)-TriHOME.^59^ The major product in pig and human skin possesses a 10(*S*)-configuration coming from an SN_2_ hydrolysis step that reverses the configuration at C-10 of the 9(*R*),10(*R*),13(*R*)-hydroxy-epoxide precursor.^59^ This high specificity supports the hypothesis that an epoxide hydrolase enzyme is involved in hydrolysis of hydroxy-epoxides in these tissues.^187^ In a cell line of human mast cells (LAD2 cells), TriHOME formation is LOX-independent and yields a 13(*R*) configuration, whereas in eosinophils this synthesis is initiated by 15-LOX, resulting in a 13(*S*)-configuration.^172^ Epidermal triols are almost exclusively esterified to ceramides and, together with epidermal keto-epoxides, possess an important role in maintaining the integrity of the water–skin barrier.^59^ Notably in plants, TriHOMEs contained in cuticle waxes have antifungal properties,^188^ and a parallel role in fungal defenses of the epithelial barrier can be postulated in animals.

**Scheme 14:**
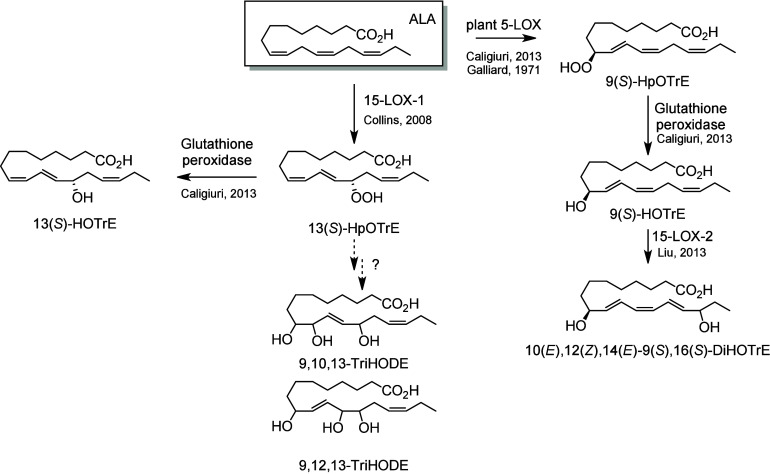
α-Linolenic Acid (ALA)-Derived Octadecanoids Produced by Lipoxygenases (LOX) Nomenclature is as described in Scheme 3. Synthetic route is referenced if known or indicated with a question mark if unknown. The dotted arrows refer to the formation of compounds for which a primary citation could not be identified in the literature.

LOX metabolites of ALA are of major importance in plants, although in animal systems ALA metabolism is less extensively studied, and only a small number of metabolites have been reported to date. The major product of ALA incubation with 15-LOX-1 is 13-HpOTrE, which is further reduced to 13-HOTrE.^189,190^ Finally, the diol 10(*E*),12(*Z*),14(*E*)-9(*S*),16(*S*)-DiHOTrE has been identified after further oxidation of 9(*S*)-HpOTrE (an ALA-metabolite of plant 5-LOX) by human 15-LOX-2.^101^ The biosynthesis of TriHODEs has not yet been reported (Scheme 14).

The metabolism of sebaleic acid by 5-LOX in neutrophils results in the production of 5-HODE, which can be further metabolized into 5-oxo-ODE by 5-hydroxyeicosanoid dehydrogenase in neutrophils and keratinocytes. The formation of 6(*E*),8(*Z*)-5(*S*)-18-DiHODE and 6(*E*),8(*Z*)-18-OH-5-oxo-ODE from sebaleic acid have also been reported, but the responsible enzymes remain unknown (Scheme 16).^191^

**Scheme 15:**
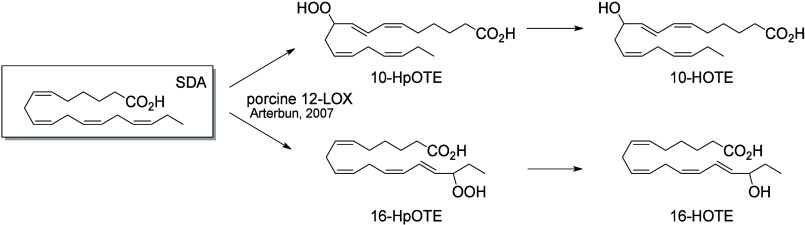
Stearidonic Acid (SDA)-Derived Octadecanoids Produced by Lipoxygenases (LOX) Nomenclature is as described in Scheme 3. Synthetic route is referenced if known.

Very few studies have been performed to identify mammalian SDA-derived octadecanoids. However, Arterburn et al. did report that the incubation of SDA with porcine 12-LOX followed by reduction with NaBH_4_ of the product led to the identification of two hydroxy-metabolites: 10-HOTE and 16-HOTE (Scheme 15).^192^

**Scheme 16:**
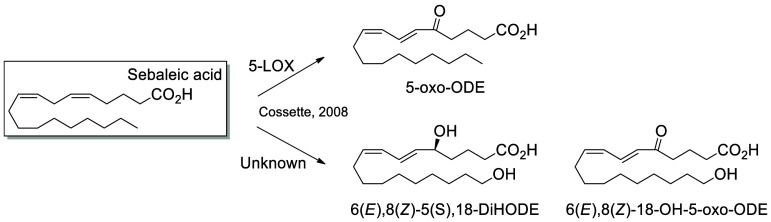
Sebaleic Acid-Derived Octadecanoids Produced by Lipoxygenases (LOX) Nomenclature is as described in Scheme 3. Synthetic route is referenced if known.

### 4.3 Cytochrome P450s

In mammals, CYPs are important enzymes that participate in oxidative, peroxidative, and reductive metabolism of a broad range of lipid substrates. CYPs are a superfamily of heme-containing monooxygenases that are ubiquitous across all domains of life.^193^ Humans possess 57 functional CYP-encoding genes, and their products can be divided into 18 families and 41 subfamilies. The CYP2, CYP3, and CYP4 families contain far more genes than the other 15 families. CYPs are widely distributed in mammalian tissues and are highly expressed in the lung, liver, brain, and kidney. Most CYPs are bound to either mitochondrial membranes or the endoplasmic reticulum, but they are also present in the plasma membrane and the nucleus. Their major functions include drug and lipid metabolism.^194^ CYPs catalyze four different reactions: ω-side chain hydroxylation, epoxidation, *bis*-allylic hydroxylation, and hydroxylation with double-bond migration (“allylic hydroxylation”).^98^

#### 4.3.1 Enzymatic Mechanism

**Scheme 17:**
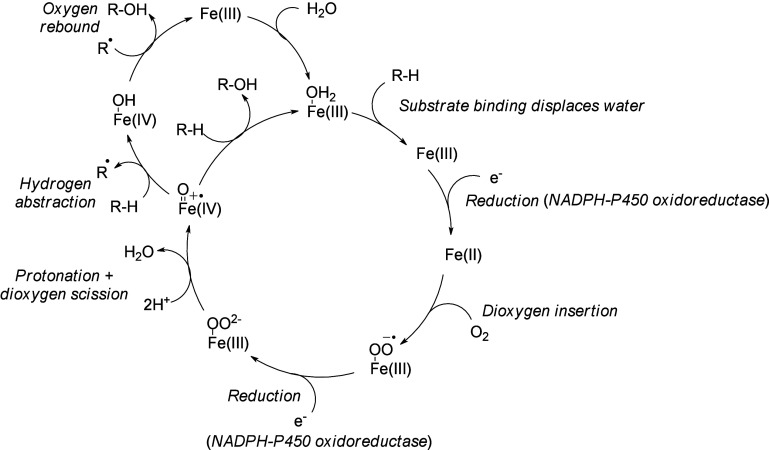
Cytochrome P450 (CYP) Catalytic Mechanism^31,196^

CYP-catalyzed oxygenations are NADPH-dependent and involve the scission of dioxygen with one oxygen atom reduced to water and the other incorporated into the substrate. In the prototypical reaction cycle, substrate recruitment displaces H_2_O from the axial position of the heme Fe(III). A NADPH-cytochrome P450 reductase undergoes a 1-electron reduction to form Fe(II), then O_2_ binding creates an oxygen–iron complex, and another reductase-assisted 1-electron reduction generates a negatively charged iron(III)-peroxo complex. Double protonation of this iron(III)-peroxo complex followed by the scission of a dioxygen molecule creates an iron(IV)-oxoporphyrin-radical cation intermediate, a direct oxidant in many CYP oxidation reactions. This oxoferryl porphyrin is responsible for hydroxylation reactions and undergoes a hydrogen abstraction followed by a radical insertion of an hydroxy group that recreates the starting Fe(III) ion (Scheme 17).^195,196^

**Scheme 18:**
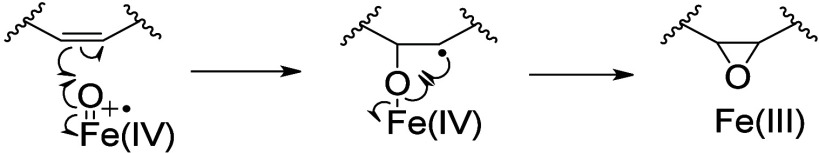
Cytochrome P450 (CYP) Epoxidation Mechanism^196^

The mechanism of epoxidation by CYPs includes formation of a π-complex between the iron(IV)-oxoporphyrin-radical cation intermediate and the alkene. The reaction proceeds with the insertion of the oxyl entity that initiates the formation of a radical intermediate. The radical scission of the oxygen–iron bond creates an epoxide and reforms Fe(III) ion (Scheme 18).

#### 4.3.2 Cytochrome P450-Derived Octadecanoids

**Scheme 19:**
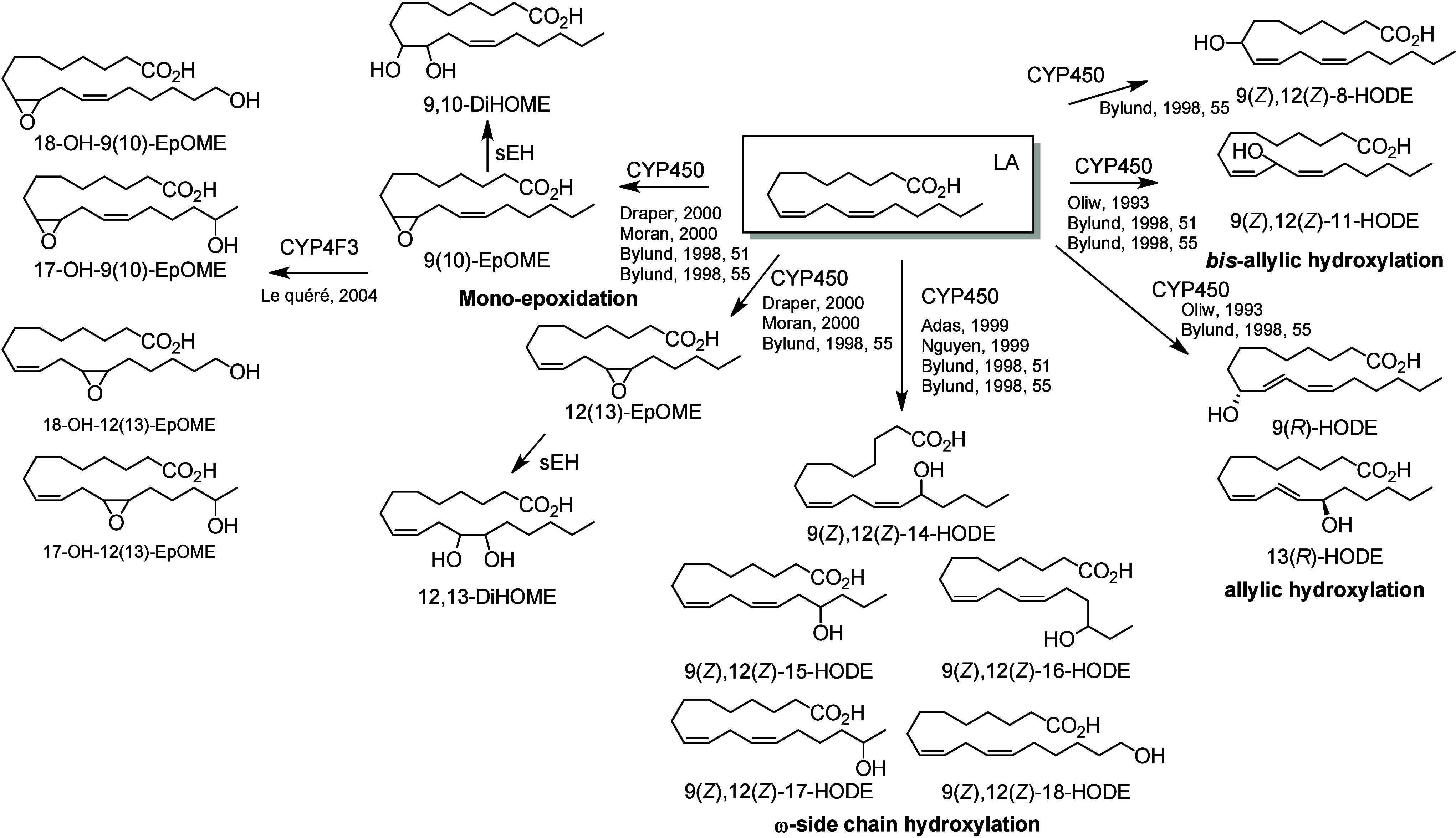
Linoleic Acid (LA)-Derived Octadecanoids Produced by Cytochrome P450 (CYP) Nomenclature is as described in Scheme 3. Synthetic route is referenced if known.

**Scheme 20:**
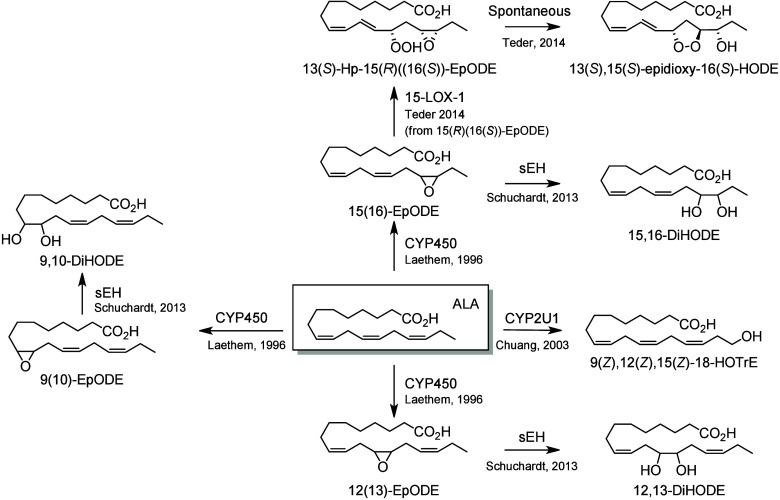
α-Linolenic Acid (ALA)-Derived Octadecanoids Produced by Cytochrome P450 (CYP) Nomenclature is as described in Scheme 3. Synthetic route is referenced if known.

**Scheme 21:**
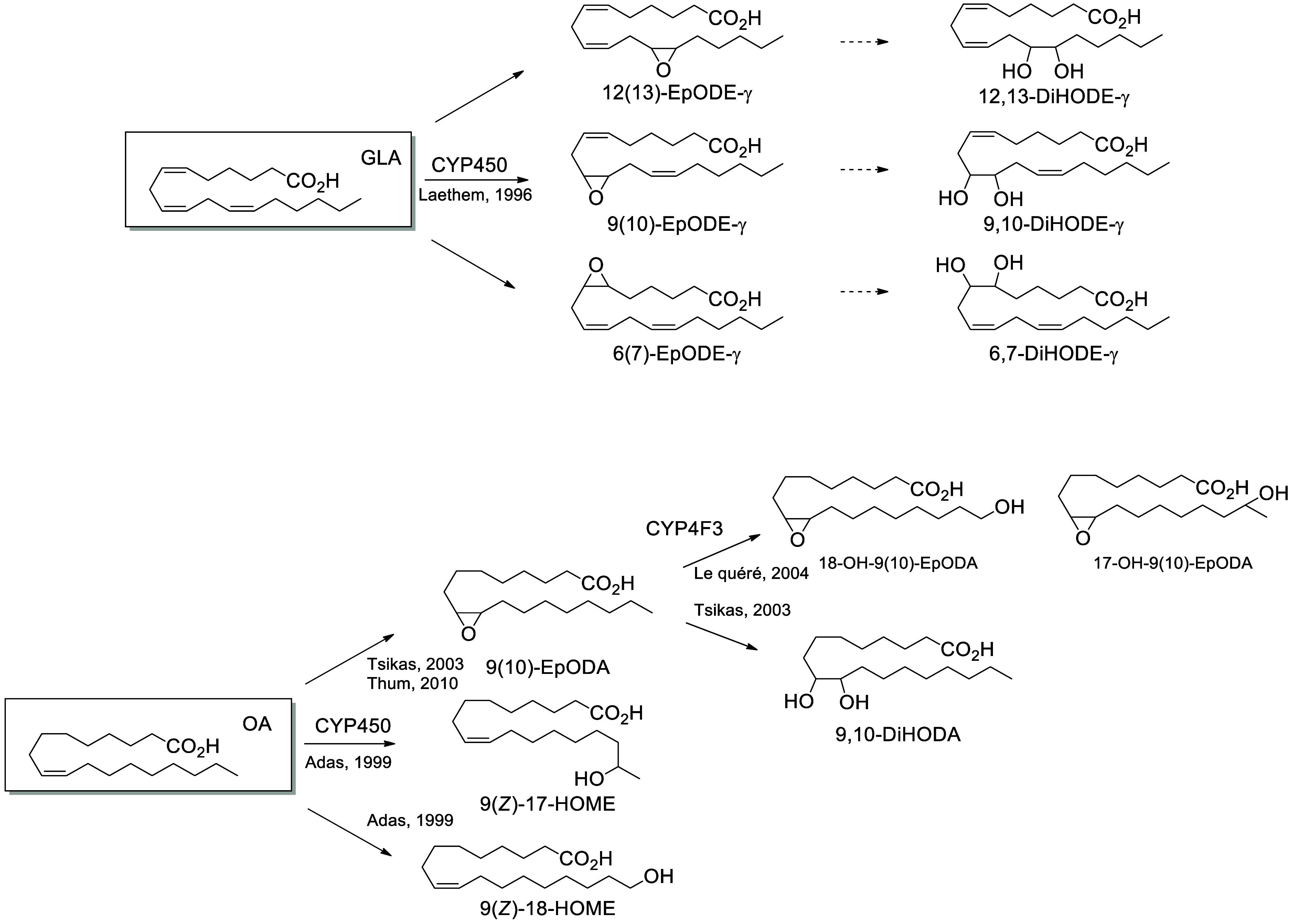
γ-Linolenic Acid (GLA)- and Oleic Acid (OA)-Derived Octadecanoids Produced by Cytochrome P450 (CYP) Nomenclature is as described in Scheme 3. Synthetic route is referenced if known. The dotted arrows refer to the formation of compounds for which a primary citation could not be identified in the literature.

*Epoxides/Diols.* CYPs oxidize LA (Scheme 19), ALA (Scheme 20), GLA, and OA (Scheme 21) into a wide range of alcohols and epoxides. LA possesses two double bonds, each of which can undergo a CYP-catalyzed epoxidation to create 9(10)-EpOME and 12(13)-EpOME. Epoxide hydrolase-catalyzed epoxide hydrolysis forms the corresponding vicinal diols 9,10-DiHOME and 12,13-DiHOME.^197^ In humans, CYP2J2 does not possess any hydroxylation function, resulting in the sole production of EpOMEs from LA.^198^ CYP2E1^198^ and CYP2C9^199^ are the major human LA epoxygenases but are also responsible for the formation of monohydroxylated species. The other CYPs responsible for epoxide and diol formation in humans are CYP1A2, CYP2E1, CYP2C8, CYP2C9, CYP2C19, CYP2J2, CYP2J3, CYP2J5, CYP2J9, and CYP3A4.^97,198,200^ These enzymes produce EpOMEs and DiHOMEs in mixture with the HODEs. In the same fashion as LA, ALA can be epoxidized by CYP enzymes on its three unsaturations to generate 9(10)-EpODE, 12(13)-EpODE, and 15(16)-EpODE.^201^ The monoepoxides can be hydrolyzed into 9,10-DiHODE, 12,13-DiHODE, and 15,16-DiHODE.^99^

It is known that the *cis*- or *trans*-configuration of an epoxide has a strong impact on the opening reaction catalyzed by sEH to form vicinal diols. For example, in the case of alkyl-phenyloxirane, the *cis*-epoxides have been found to be poor substrates for sEH. Among the two *trans*-epoxides (*R*,*R*) and (*S*,*S*), both plant and mammalian sEH have been shown to be enantioselective. Mouse and human sEH are both enantioselective for (*S*,*S*)-alkyl-phenyloxiranes, while cress sEH is enantioselective for (*R*,*R*)-alkyl-phenyloxiranes.^202^

Relatively recent studies demonstrated the lipoxygenase-catalyzed formation of hydroxy-endoperoxides from EpETrE-type fatty acid epoxides.^203^ One example is the transformation of 15(*R*),16(*S*)-EpODE by soybean LOX-1. In association with the expected formation of a 13(*S*)-hydroperoxide, intramolecular nucleophilic substitution (S_Ni_) between the hydroperoxy (nucleophile) and 15(16)-epoxy group (electrophile) forms a 13(*S*),(15(*S*))-epidioxy-16(*S*)-HODE (Scheme 20). The configuration of the endoperoxide (*cis* or *trans* side chains) was shown to depend on the steric relationship of the new hydroperoxy moiety to the enantiomeric configuration of the fatty acid epoxide.^203^ The results were proposed as a potential interaction of lipoxygenase and cytochrome P450 product formation.

GLA is also a CYP substrate and its three double bonds can be epoxidized by CYP2B1, giving 6(7)-EpODE-γ, 9(10)-EpODE-γ, and 12(13)-EpODE-γ.^201^ 12(13)-EpODE-γ as well as a smaller amount of 9(10)-EpODE-γ can also be generated by CYP2CAA and CYP2C2.^201^ CYPs (2C8, 2C9, 3A7, and 3A4 mainly) can also oxidize monounsaturated fatty acids to form the epoxide and diol metabolites. For example, 9(10)-epoxy-octadecanoic acid (i.e., 9(10)-EpODA) and 9,10-dihydroxy-octadecanoic acid (9,10-DiHODA) are produced from OA.^204,205^

*Alcohols.* Certain CYPs are also able to catalyze *bis*-allylic hydroxylation of LA to produce 11-HODE.^200,206^ This *bis*-allylic alcohol is unstable under acidic conditions and rearranges into racemic 9-HODE and 13-HODE. In human liver microsomes, the main CYPs responsible for the formation of 9(*Z*),12(*Z*)-11-HODE are CYP1A2 and CYP3A4.^200^ Allylic hydroxylation of LA also produces 9-HODE and 13-HODE possessing an (*R*)-configuration. The CYP responsible for the allylic hydroxylation of LA are CYP1A2, CYP2C9, and CYP3A4.^97^

Finally, LA can be hydroxylated in the 5 positions ω1 to ω5, creating 9(*Z*),12(*Z*)-14-HODE, 9(*Z*),12(*Z*)-15-HODE, 9(*Z*),12(*Z*)-16-HODE, 9(*Z*),12(*Z*)-17-HODE, and 9(*Z*),12(*Z*)-18-HODE.^97,200,207,208^ CYP2C9 can generate all of these metabolites, but other CYPs are specific for hydroxylation at single positions.^97^ For example, CYP2C19^97^ and CYP2E1^207^ form 9(*Z*),12(*Z*)-17-HODE as the major product, and CYP4A1 and CYP4A11 form primarily 9(*Z*),12(*Z*)-18-HODE.^207,208^ CYP1A2, CYP2C8, CYP2C9, CYP2C19, and CYP3A4 can catalyze hydroxylation on both the ω1 and ω2 positions.^200^ The ω1-hydroxylation of ALA to produce 9(*Z*),12(*Z*),15(*Z*)-18-HOTrE, has been reported by CYP2U1.^209^ Mammalian CYP2E1 can form the ω1 and ω-hydroxylated products 9(*Z*)-17-HOME and 9(*Z*)-18-HOME from OA,^207^ and 17-HODA and 18-HODA are produced from SA in rat microsomes in a NADPH-dependent reaction.^210^ The formation of 8-HODE from LA by CYP2C9 has been reported.^97^

*Hydroxy-epoxides/Nonvicinal Diols.* A few LA metabolites derived from a combination of these activities have also been observed. CYP4F3 enzymes are the main enzymes involved in the oxidation of epoxy-FAs, and are responsible for the ω1-hydroxylation, and to a lesser extent (ω2)-hydroxylation, of EpODA and EpOMEs. The ratios of ω/(ω1) hydroxylation were 8 and 7 for 9(10)-EpODA and 9(10)-EpOME, respectively, but decreased to 1.6 for 12(13)-EpOME.^211,212^ CYP4F11 produces 3,18-DiHODA by ω1-oxidation of 3-HODA in human liver.^171^

## 5 Microbial and Fungal Sources

### 5.1 Gut Microbiota Octadecanoids

Microbes and bacteria that constitute the gut microbiome are human symbionts that exert a vital function in the health status of their hosts.^213^ Among the various interactions, the gut microbiota exert profound effects on the host metabolism by transforming dietary fatty acids into bioactive molecules.^214,215^ Lipid metabolites produced by the gut microbiota can impact human health, interacting with different organ systems including gut–artery,^216,217^ gut–brain,^218^ and gut–lung^219,220^ axes. Gut dysbiosis can therefore induce multiple physiological consequences. For example, neonates at risk of childhood atopy, eczema, and asthma exhibit perturbation of the gut microbiome, including an increased number of bacterial epoxide hydrolase genes.^70,71^ This enzyme forms the 12,13-DiHOME, which has physiological functions associated with fatty acid catabolism and energetics^63,221^ but also exerts deleterious effects on lung function in patients with severe burns or COVID-19 infection.^68,71,222^ Moreover, specific lipid metabolites produced by the gut microbiota have been shown to confer resistance to inflammation caused by high fat diet (HFD)-induced obesity in mice.^79^ Accordingly, there are multiple health and physiological effects associated with microbiome-derived octadecanoids that are deserving of investigation.

The metabolism of C18-FAs by gastrointestinal anaerobic microbiota (e.g., lactic acid bacteria), generates multiple conjugated fatty acids and *trans*-fatty acids that can affect host lipid metabolism.^223^ Lactic acid bacteria transform growth-inhibiting PUFAs containing *cis*–*cis* pentadiene moieties into less toxic saturated fatty acids.^224^ In the process, CLAs, and *trans*-FAs are released into the host tissues. In ruminants, a multitude of these intermediate products are generated and may end up in consumed meat as well as milk.^225,226^ CLA defines a broad family of isomers possessing at least two conjugated double bonds, that exert a variety of effects on human health, the nature of which is still debated. However, abundant species such as rumenic acid and its isomer 10(*E*),12(*Z*)-octadecadienoic acid, were found to possess anticarcinogenic activity.^227^ The role of CLA^227,228^ and CLnA^229,230^ in cancer prevention have been reviewed. The group of 28 CLA isomers represents a significant source of substrate for downstream octadecanoids that have been little explored. The effects of *trans*-fatty acids on human health are clearer. For example, 10(*E*)-octadecenoic acid, the major isomer typically found in meat and milk, has been shown to have adverse effects on cardiovascular health.^231,232^ The bioactivity and health effects of ruminant meat lipids has been extensively reviewed by Vahmani et al.^233^

**Scheme 22:**
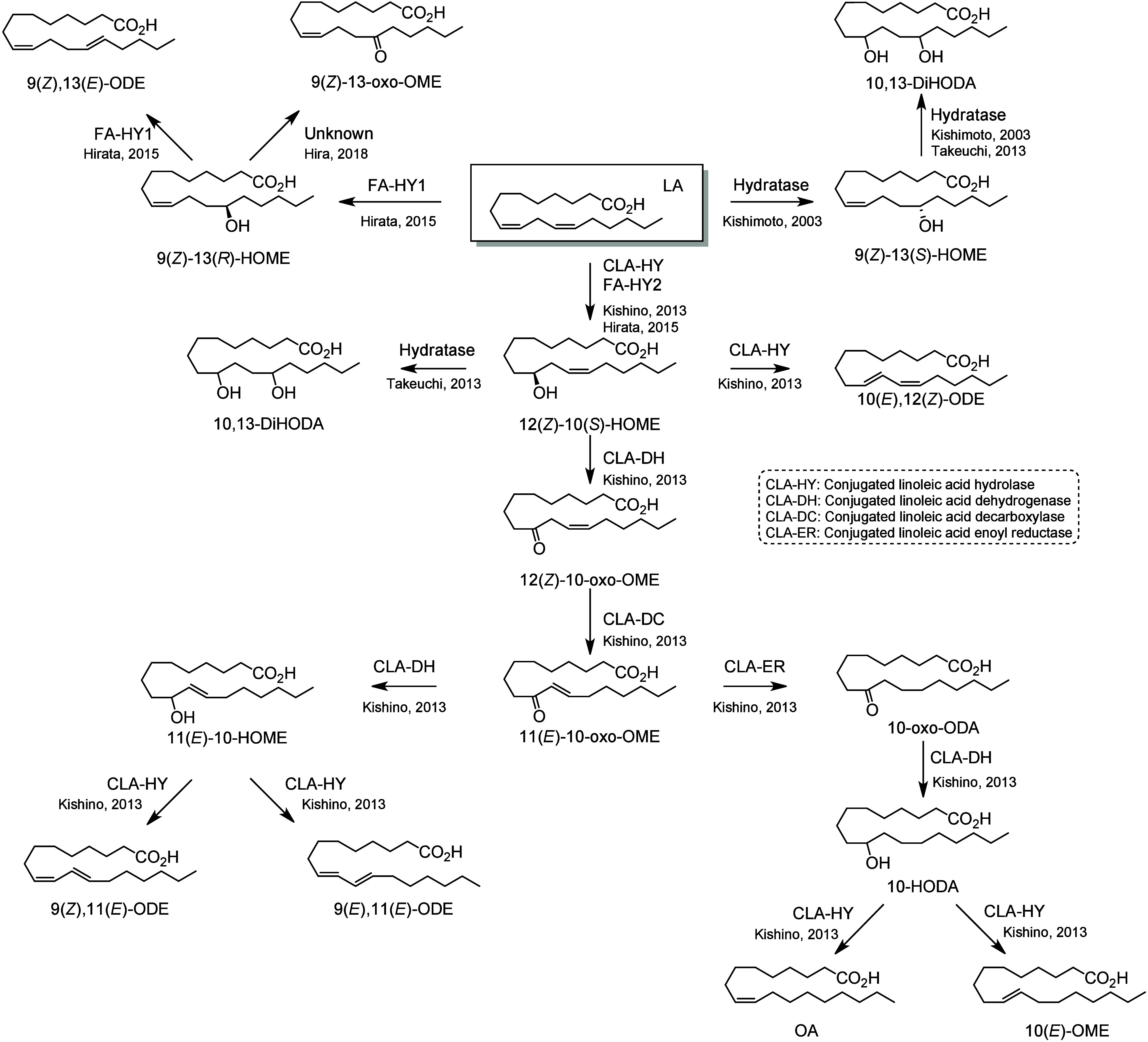
Linoleic Acid (LA)-Derived Octadecanoids Produced by the Gut Microbiota Nomenclature is as described in Scheme 3. Synthetic route is referenced if known.

**Scheme 23:**
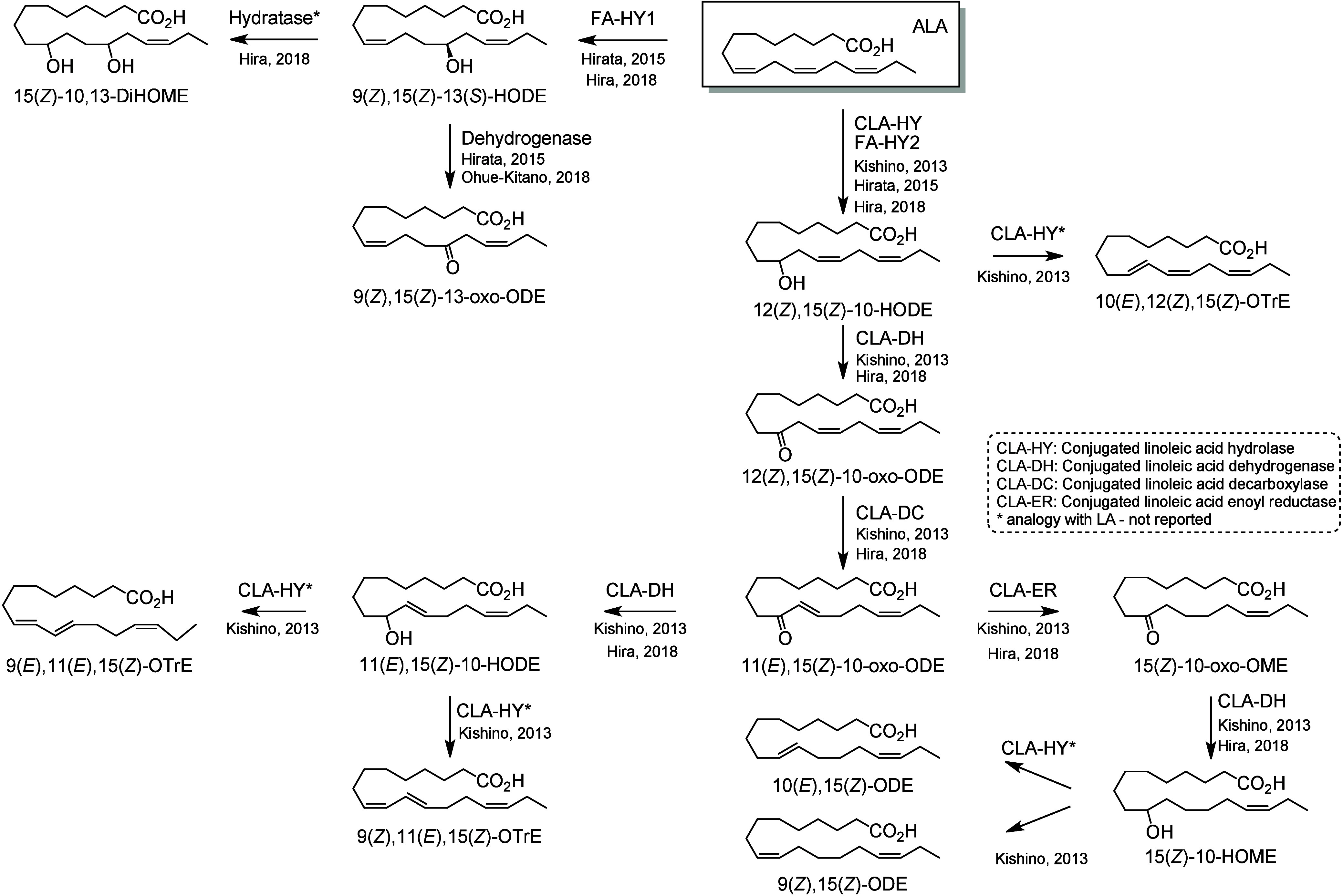
α-Linolenic Acid (ALA)-Derived Octadecanoids Produced by the Gut Microbiota Nomenclature is as described in Scheme 3. Synthetic route is referenced if known.

**Scheme 24:**
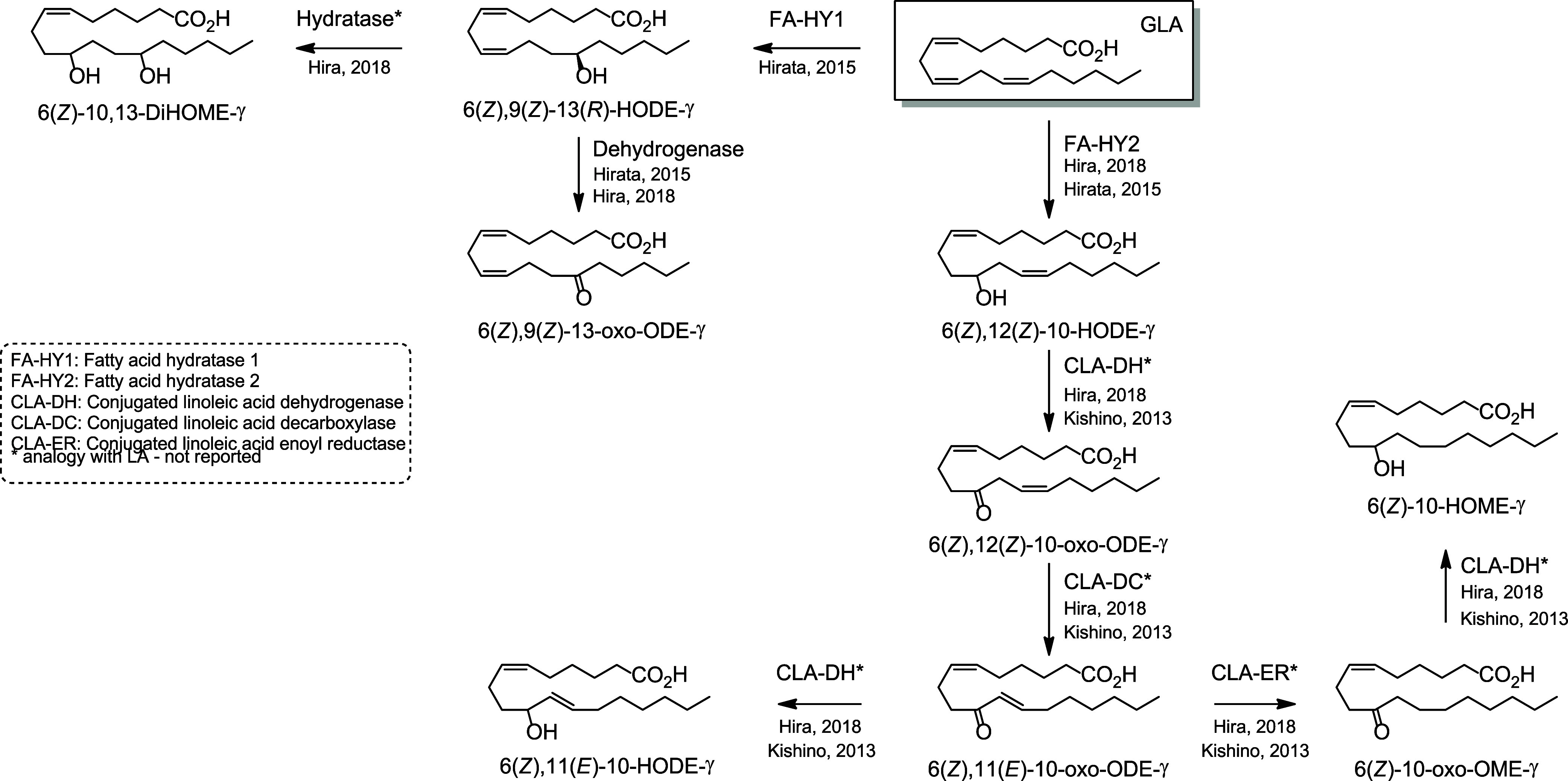
γ-Linolenic Acid (GLA)-Derived Octadecanoids Produced by the Gut Microbiota Nomenclature is as described in Scheme 3. Synthetic route is referenced if known.

Specific octadecanoids are also produced by most lactic acid bacteria and possess an alcohol or a ketone at the 10- or 13-position. The bacterial pathways leading to both series of metabolites were first described by the pioneering work of Jun Ogawa and colleagues.^223^ It is important to highlight that these enzymatic reactions occur in an anaerobic environment, and the introduction of the hydroxy group occurs via hydration and not oxidation. Hira et al. characterized the production of a large number of C18-FA-derived metabolites by gut lactic acid bacteria.^234^ In a representative gut bacterium *Lactobacillus plantarum* from a mouse model, four enzymes involved in the bacterial metabolism of PUFAs were identified: CLA-hydrolase (CLA-HY), CLA-dehydrogenase (CLA-DH), CLA decarboxylase (CLA-DC), and CLA-enoyl reductase (CLA-ER).^223^ The first reaction of LA metabolism is the hydration of the 9,10-alkene catalyzed by CLA-HY, which exhibits both hydratase and dehydratase activities and converts LA into 12(*Z*)-10(*S*)-HOME. In *Lactobacillus acidophilus*, this step can also be catalyzed by the enzyme fatty acid hydratase 2 (FA-HY2).^235^ The resulting monohydroxylated metabolite is subsequently dehydrated by CLA-HY to generate the conjugated-LA derivative 10(*E*),12(*Z*)-ODE. The next step is the oxidation of 12(*Z*)-10-HOME catalyzed by CLA-DH in the presence of NAD^+^. CLA-DH possesses both dehydrogenase and oxidoreductase activities and generates the oxo-product 12(*Z*)-10-oxo-OME. It can also catalyze the reverse reaction and reduce the ketone into the corresponding hydroxy in the presence of NADH. The Δ^12^-*cis* double bond is isomerized by acetoacetate decarboxylase CLA-DC, to form the more stable conjugated enone product 11(*E*)-10-oxo-OME. This enone is saturated by the enone reductase CLA-ER to generate 10-oxo-ODA in the presence of FAD/FMN and NADH. The 10-oxo-ODA is then reduced by CLA-DH into the corresponding alcohol 10-HODA. The final elimination of the hydroxy by CLA-HY creates OA and 10(*E*)-OME. CLA-DY can also catalyze the reduction of 11(*E*)-10-oxo-OME into 11(*E*)-10-HOME, which is dehydrated by CLA-HY into two conjugated-LA compounds 9(*Z*),11(*Z*)-ODE and 9(*E*),11(*Z*)-ODE (Scheme 22).^223^ Additional C18-PUFAs, ALA (Scheme 23) and GLA (Scheme 24), are also metabolized by these enzymes following the same pathway.^223^ For example, FA-HY2 efficiently converts ALA, GLA, and OA into the corresponding 10-hydroxy fatty acids.^235^

Most gut lactic acid bacteria generate the same metabolites as *Lactobacillus plantarum.* However, some lactic acid bacteria such as *Lactobacillus acidophilus* can also produce metabolites that are hydrated at the 13-position. The enzyme responsible for the hydration of C18-PUFAs on their 13-position has been identified in *Lactobacillus acidophilus* NTV001 as a hydratase termed FA-HY1.^235^ This enzyme can react with all C18-PUFAs possessing a Δ^12^*cis*-double bond including LA, ALA, GLA, and SDA. Incubation of PUFAs with FA-HY1 produces 9(*Z*)-13(*R*)-HOME from LA, 9(*Z*),15(*Z*)-13(*S*)-HODE from ALA and 6(*Z*),12(*Z*)-10-HODE from GLA. 9(*Z*)-13(*R*)-HOME is further metabolized by FA-HY1 into 9(*Z*),13(*E*)-ODE.^235^ The stereochemistry of 9(*Z*)-13(*R*)-HOME, produced from LA by FA-HY1 in the strain NTV001 of *L. acidophilus*, is distinct from that of the previously reported 9(*Z*)-13(*S*)-HOME produced by the strain 13951 of *L. acidophilus*.^236^ Through the screening of ∼300 strains of lactic acid bacteria, *Pediococcus sp.* AKU 1080 has also been identified as a strain with the ability to hydrate LA into three hydroxy-fatty acids, 12(*Z*)-10-HOME, 9(*Z*)-13-HOME, and 10,13-DiHODA. This last metabolite can be obtained from both 12(*Z*)-10-HOME and 9(*Z*)-13-HOME.^237^ The corresponding ketones have also been identified as gut bacteria metabolites; however, the enzymes responsible for their formation remain unknown.^234^

### 5.2 Other Bacterial and Microbial Metabolites

Multiple types of bacteria and microbes produce hydroxy fatty acids from different unsaturated fatty acids. Among them, *Streptococcus*,^238^*Nocardia*,^239^ and *Flavobacterium*(240) convert LA into 12(*Z*)-10-HOME. In addition to these well-known metabolites, some bacteria can produce unique metabolites of PUFAs, including diols, triols, tetrahydrofuranyl fatty acids (THFA), and bicyclic-fatty acids.

**Scheme 25:**
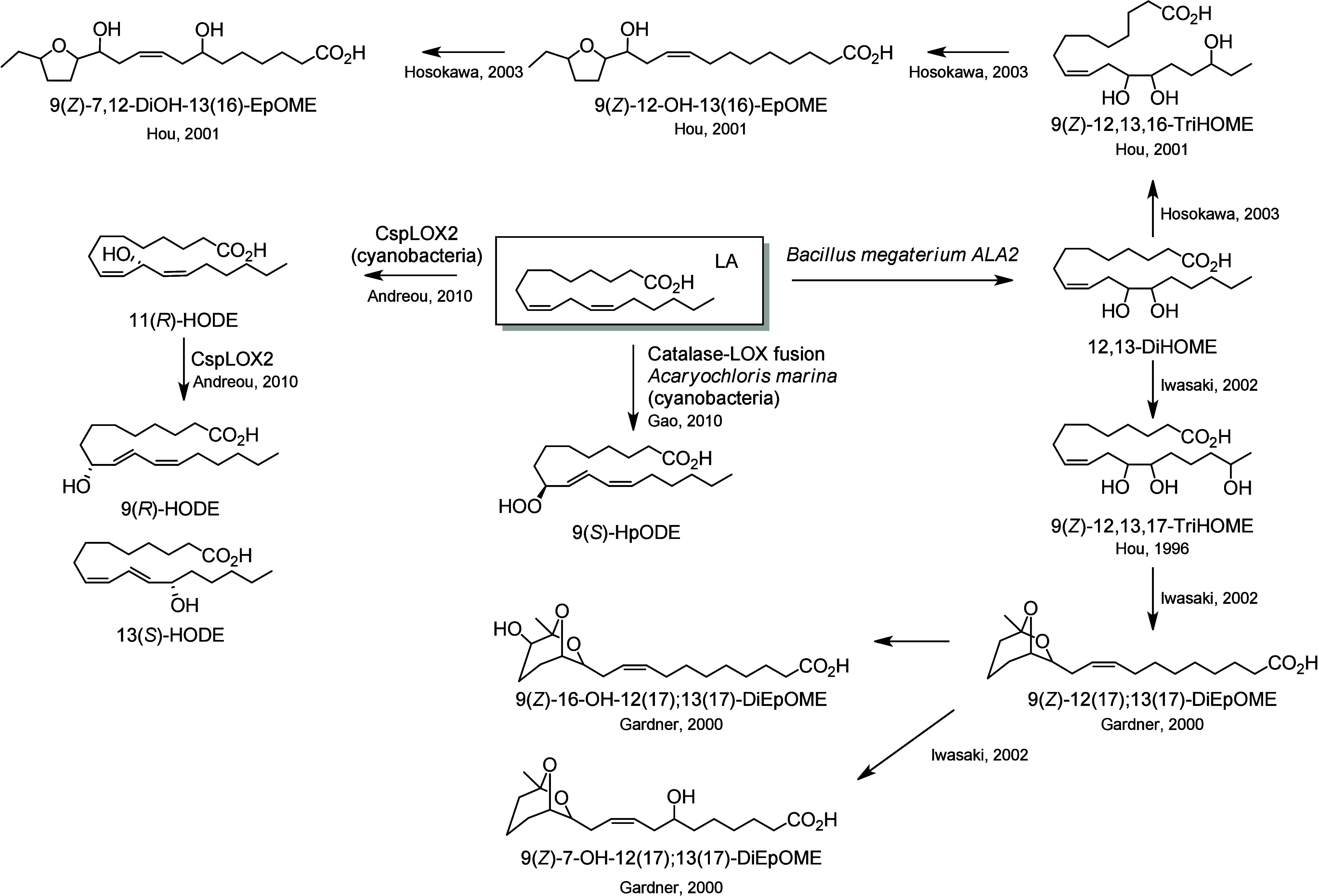
Linoleic Acid (LA)-Derived Octadecanoids Produced by Bacteria Nomenclature is as described in Scheme 3. Synthetic route is referenced if known.

A bacterial source of C18-PUFA metabolites has been extensively studied by Hou and colleagues.^241^ The bacterium responsible for the formation of a large range of unique PUFA metabolites is the strain *Bacillus megaterium* ALA2, which has been shown to effectively oxidize LA at the 7-, 12-, 13-, 16-, and/or 17-position, producing numerous unique triols and cyclic PUFA metabolites (Scheme 25). The production of an uncommon triol compound, 9(*Z*)-12,13,17-TriHOME, from LA by a microbial culture of *B. megaterium* ALA2 isolated from a dry soil sample, has been reported.^242^ This triol is the major product formed from LA by this bacterial strain, and is generated from 12,13-DiHOME.^243^ It is a precursor of three diepoxy bicyclic fatty acids, 9(*Z*)-12(17);13(17)-DiEpOME, 9(*Z*)-7-OH-12(17);13(17)-DiEpOME, and 9(*Z*)-16-OH-12(17);13(17)-DiEpOME that have been identified from LA.^244,245^ The generation of a second original triol (9(*Z*)-12,13,16-TriHOME), has also been reported by the same group. This second TriHOME can be epoxidized to a THFA (9(*Z*)-12-OH-13(16)-EpOME), which can be further metabolized into another THFA (9(*Z*)-7,12-DiOH-13(16)-EpOME).^246^

**Scheme 26:**
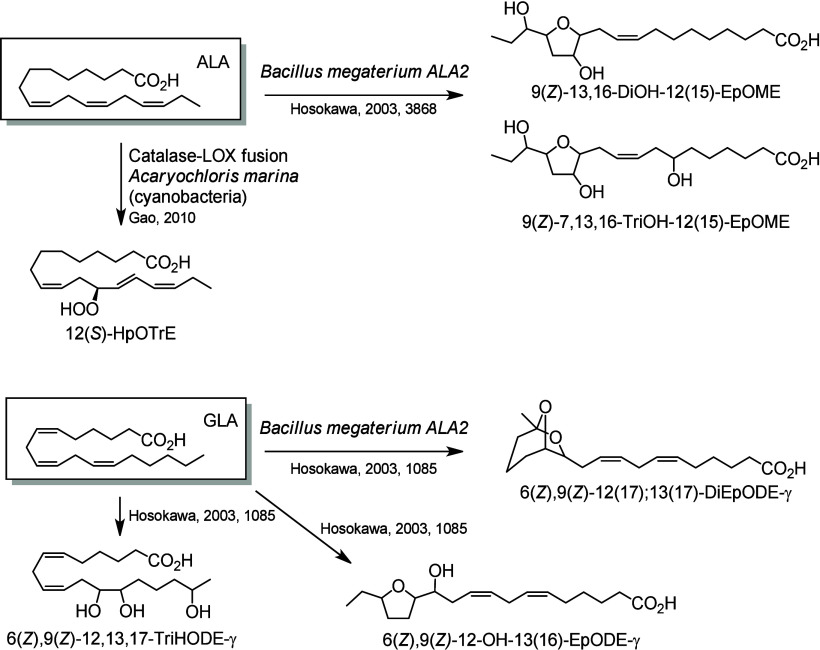
α-Linolenic Acid (ALA)- and γ-Linolenic Acid (GLA)-Derived Octadecanoids Produced by Bacteria Nomenclature is as described in Scheme 3. Synthetic route is referenced if known.

*B. megaterium* ALA2 also produces THFA from ALA and GLA (Scheme 26). In 2003, Hosokawa et al. identified two new ALA metabolites, 9(*Z*)-13,16-DiOH-12(15)-EpOME and 9(*Z*)-7,13,16-TriOH-12(15)-EpOME,^247^ and three new GLA metabolites, 6(*Z*),9(*Z*)-12(17);13(17)-DiEpODE, 6(*Z*),9(*Z*)-12,13,17-TriHODE, and 6(*Z*),9(*Z*)-12-OH-13(16)-EpODE.^248^

Cyanobacteria possess mini-LOX enzymes, which show only the LOX’s C-terminal catalytic domain that contains iron. The mini-LOX of *Cyanothece sp*. (CspLOX_2_) creates *bis*-allylic hydroperoxides, 11(*R*)-HODE from LA, as well as 9(*R*)-HODE and 13(*S*)-HODE.^249^ The lipoxygenase domain of a catalase-LOX fusion protein from the cyanobacterium *Acaryochloris marina* converts LA to 9(*R*)-HpODE and oxygenates omega-3 fatty acids at the ω7 carbon, forming 12(*R*)-HpOTrE from ALA.^250^

**Scheme 27:**
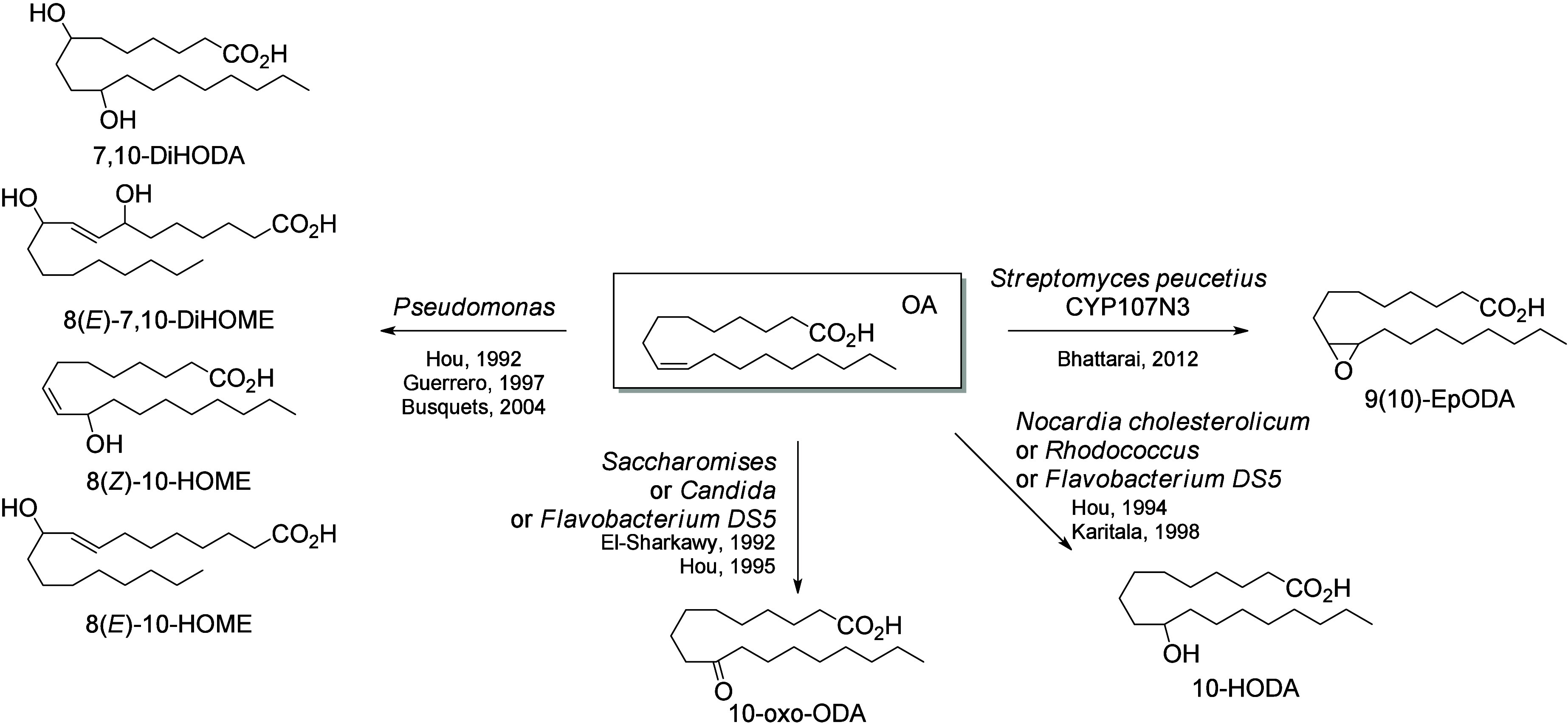
Oleic Acid (OA)-Derived Octadecanoids Produced by Bacteria and Microbes Nomenclature is as described in Scheme 3. Synthetic route is referenced if known.

Microbial and bacterial transformations of OA have been extensively studied. OA can be metabolized by a large range of microbes and bacteria, most of which oxidize the 10-position, but oxidation of the 7-position has also been reported.^241,251,252^*Nocardia cholesterolicum*,^239,241^*Rhodococcus sp*.,^239^ and *Flavobacterium DS5*(253) can hydroxylate OA into 10-HODA. The corresponding ketone, 10-oxo-ODA, can be produced by *Flavobacterium DS5*,^252^ as well as by fungal strains such as *Saccharomyces sp.* and *Candida sp.*.^251^ In their effort to convert agricultural oils to value-added industrial chemicals, Hou et al. extensively studied the bioconversion of OA by the bacterial stain *Pseudomonas* PR3. They reported the formation of a new dihydroxylated compound, 8(*E*)-7,10-DiHOME.^254^ In addition, they identified a monohydroxylated metabolite produced by the same strain, 8(*Z*)-10-HODE, and hypothesized it to be a potential intermediate in the bacterial biosynthesis of 8(*E*)-7,10-DiHOME.^255^ Guerrero et al. continued this study and showed that 8(*E*)-10(*S*)-HOME is generated by *Pseudomonas* 42A2 from OA and can be transformed into 8(*E*)-7,10-DiHOME.^256^ The enzyme responsible for this transformation was reported to be a LOX-like enzyme.^257^ Finally, OA can be epoxidized into 9(10)-EpODA by the enzyme CYP107N3 of *Streptomyces peucetius* (Scheme 27).^258^

### 5.3 Fungal Metabolites

Fungi can penetrate an organism *via* alimentation (mushrooms) or by the airways due to their presence in inhaled air.^259^ Fungi can generate biologically active PUFA metabolites that are also produced in humans (e.g., 13-HODE, EpOMEs), which may interfere with the host metabolism as shown for eicosanoids.^260^ It is known that fungal octadecanoids can interfere in plant signaling pathways by mimicking endogenous oxylipins;^261−263^ however, the influence of fungal-generated endogenous octadecanoids in animals is mostly unexplored. Fungi also possess diverse enzymes that exhibit unusual catalytic activities and can generate a high diversity of specific metabolites, whose effects on animals remain to be investigated.

The transformation of LA in mushrooms has a long history aimed at unravelling the mechanistic basis for formation of the characteristic mushroom aroma of the 8-carbon alcohol, 3(*R*)-1-octen-3-ol. In the 1980s, Wurzenberger and Grosch uncovered the LA metabolism leading to this 1-octen-3-ol, identifying the two steps as the oxygenation of LA to 10(*S*)-HpODE followed by its cleavage to the 10-carbon acid-aldehyde 8(*E*)-10-oxo-decanoate and the 8-carbon alcohol.^264−266^ They also showed that ALA is converted via the equivalent transformations, generating 1,5(*Z*)-octadien-3-ol and 2(*Z*),5(*Z*)-octadien-1-ol.^267^ It is apparent that a LOX enzyme would be incapable of oxygenating LA to the 10-hydroperoxide and therefore the participation of a heme oxygenase-peroxidase has been long suspected. In 2012, a cyanobacterial catalase-heme dioxygenase fusion protein was show to transform LA to the two products.^268^ The proof of the conjecture with mushroom proteins came quite recently with the characterization of a heme dioxygenase in *Coprinopsis cinerea*;^269^ the expressed cDNAs of two heme dioxygenase-P450 fusion proteins converted LA to the 10(*S*)-HpODE. At least in this mushroom species, the second step of hydroperoxide lyase cleavage was not catalyzed by the expressed cDNAs and the hydroperoxide lyase enzyme remains uncharacterized.

The understanding of fungal enzymatic systems involved in the production of octadecanoids has its roots in the seminal work of Ernst Oliw who investigated these mechanisms in various fungal species. Dioxygenase–cytochrome P450 fusion enzymes (DOX-CYP) are common fungal enzymes that are divided into seven subfamilies (5,8-LDS, 7,8-LDS, 8,11-LDS, 10(*R*)-DOX, 10(*R*)-DOX-EAS, 9(*R*)-DOX-AOS, and 9(*S*)-DOX-AOS).^270^ Linoleate diol synthases (LDS) contain a heme and exhibit two related enzyme activities. They catalyze the dioxygenation of the 8-position of LA and the isomerization of a hydroperoxide group into dihydroxy-LA.^271^ LDS oxidize LA to 9(*Z*),12(*Z*)-8(*R*)-HpODE (8(*R*)-DOX activity), which is further metabolized into 9(*Z*),12(*Z*)-5,8-DiHODE by 5,8-LDS (in *Aspergillus sp*.),^272,273^ 9(*Z*),12(*Z*)-7,8-DiHODE by 7,8-LDS (in *Gaeumannomyces graminis*(274,275) and *Magnaporthe grisea*(276)), and 9(*Z*),12(*Z*)-8(*R*),11(*S*)-DiHODE by 8,11-LDS (in *Aspergillus clavatus*,^277^*Aspergillus fumigatus*,^273^ or *Penicillium chrysogenum*(278)). 10(*R*)-DOX-EAS oxidizes LA into 10(*R*)-HpODE, which is further metabolized into 8(*E*)-10-OH-12(13)-EpOME. This enzyme also produces smaller amounts of monohydroxylated metabolites 9(*Z*),12(*Z*)-8-HODE and 10-HODE, and it has also been shown to oxidize the 8- and 10-position of ALA and OA, but not of GLA, which is a poor substrate.^279^ 9(*R*)-DOX-AOS and 9(*S*)-DOX-AOS oxidize LA to 10(*E*),12(*Z*)-9(*S*)(10)-EpODE and 10(*E*),12(*Z*)-9(*R*)(10)-EpODE.^279^ The production of 9(*Z*),12(*Z*)-17-HODE, 9(*Z*),12(*Z*)-8,17-DiHODE, and 9(*Z*),12(*Z*)-8,16-DiHODE *in vitro* from LA by an of as yet undetermined CYP has also been reported in the rice blast fungus *M. grisea*.^276^

**Scheme 28:**
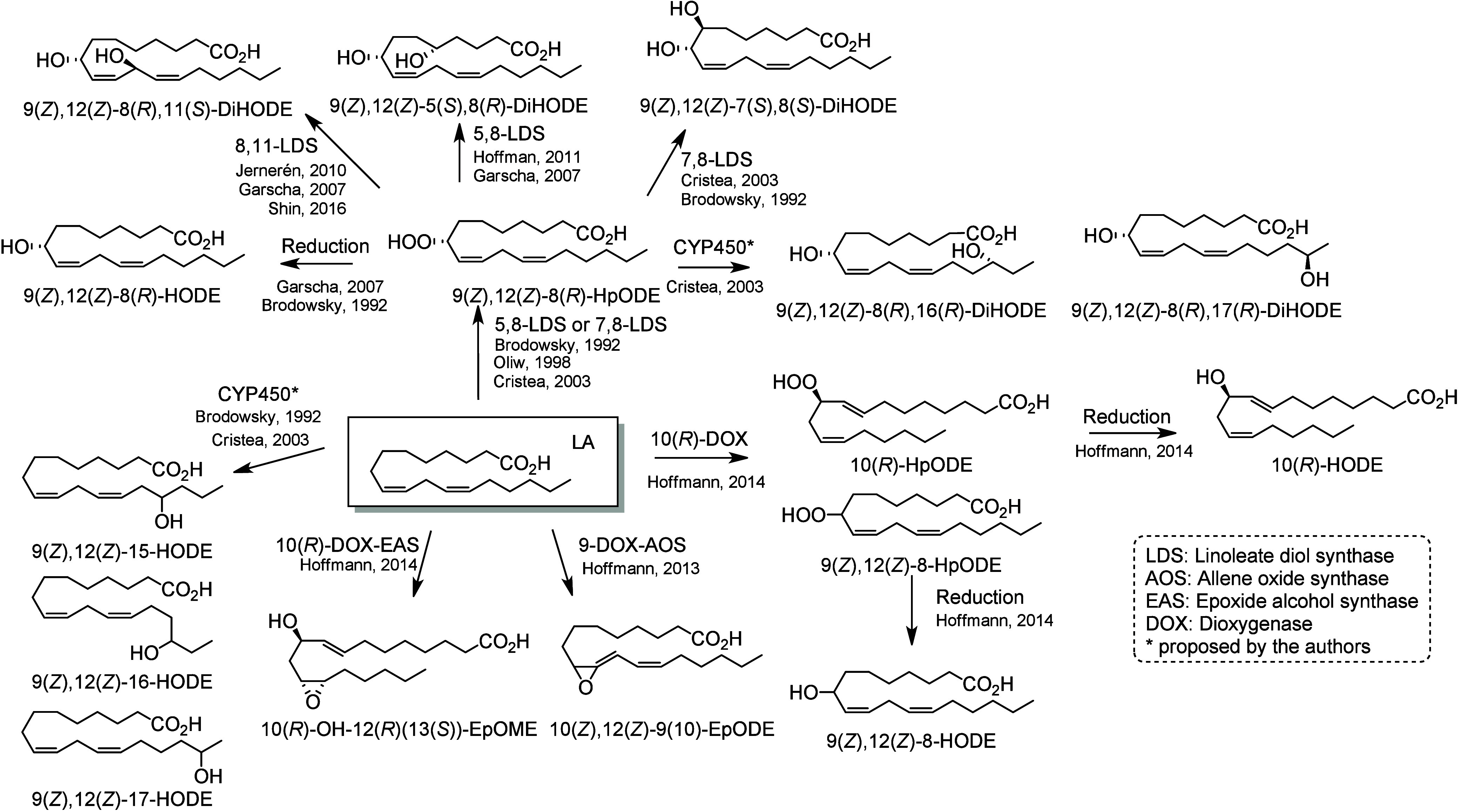
Linoleic Acid (LA)-Derived Octadecanoids Produced by Fungi Enzymes Nomenclature is as described in Scheme 3. Synthetic route is referenced if known. The scheme is split into two due to size limitations (see Scheme 29).

In the cytosolic fraction of *G. graminis*, the products of LA hydroxylation to ω2 or ω3 alcohols and of ALA’s ω3 unsaturation epoxidation and hydrolysis have been observed, but the responsible CYP(s) remain unknown (Scheme 28).^275^

Fungi also possess LOX enzymes containing a manganese ion instead of the more common ferric ion in their active site.^280^ The *G. graminis* 13(*R*)-MnLOX, also called Mn-LO, forms 13(*R*)-HpODE and 13(*R*)-HpOTrE from LA and ALA, respectively. 9(*Z*),12(*Z*)-11(*S*)-HpODE is first produced and then rearranges into 13(*R*)-HpODE *via* a linoleoyl radical.^281^ GLA is a poor substrate for Mn-LO, but minor amounts of 11-HOTrE-γ and 13-HOTrE-γ have been observed.^280^

**Scheme 29:**
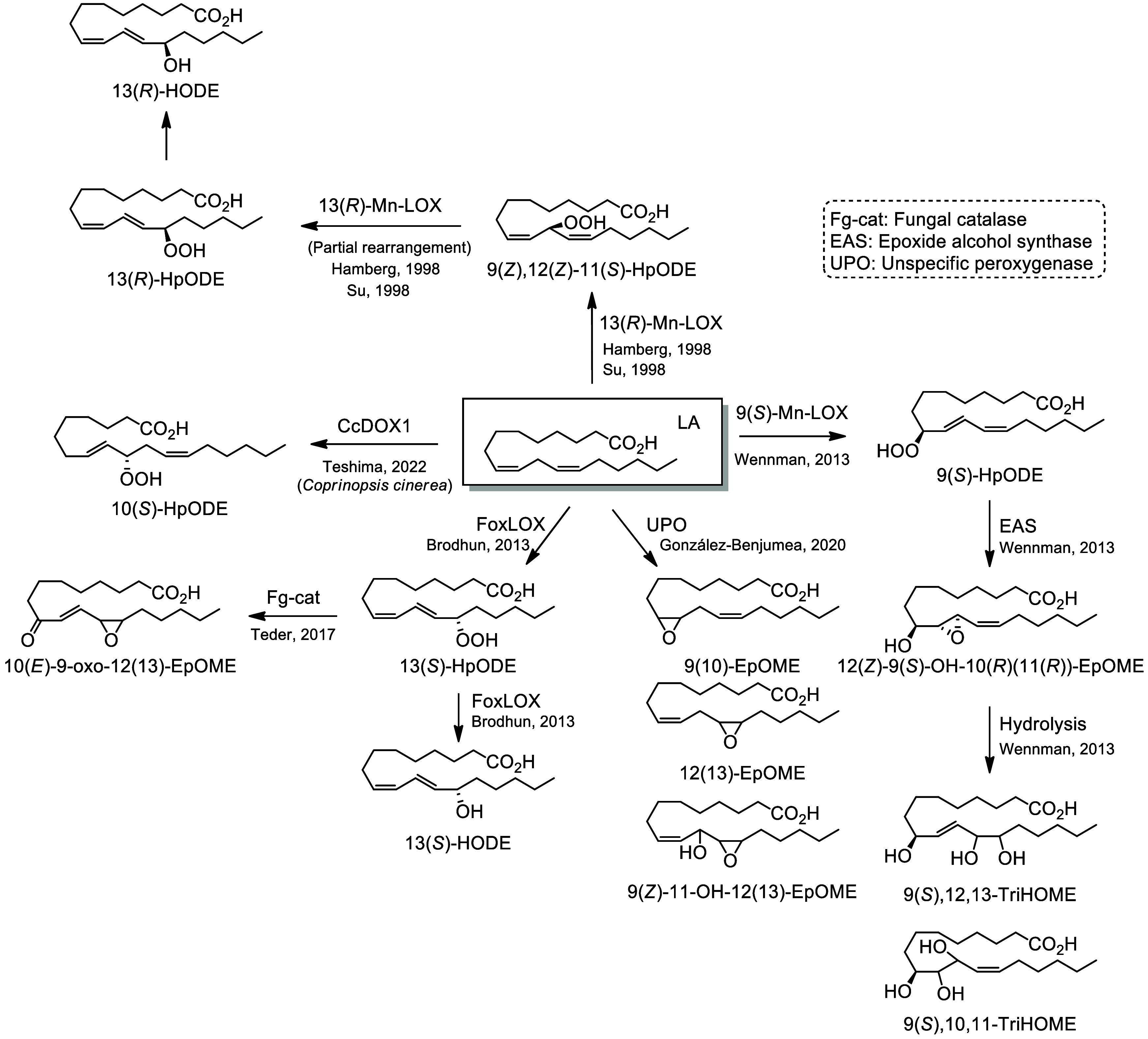
Linoleic Acid (LA)-Derived Octadecanoids Produced by Fungi Enzymes Nomenclature is as described in Scheme 3. Synthetic route is referenced if known. The scheme is split into two due to size limitations (see Scheme 28).

A fungal 9(*S*)-Mn-LOX and an epoxy-alcohol synthase have been found in the mycelium of the rice stem pathogen, *Magnaporthe salvinii*. This new Mn-LOX generates 9(*S*)-HpODE from LA, which is further metabolized by EAS into 12(*Z*)-9(*S*)-OH-10(*R*)(11(*R*))-*trans*-EpOME. The hydrolysis of the epoxide generates two triols, 9(*S*),10,11-TriHOME and 9(*S*),12,13-TriHOME. ALA is metabolized with little positional specificity at the 9-, 11- or 13-position into 9(*S*)-, 11(*R*)- or 13(*R*)-HpOTrE. 9(*S*)-HpOTrE is further metabolized into 10(*E*),12(*Z*),14(*E*)-9,16-DiHOTrE (Scheme 29). Finally, 9(*S*)-Mn-LOX can oxidize GLA into 9-HOTrE-γ.^282^

Another original enzyme found in *Fusarium oxysporum* is an iron-containing 13(*S*)-LOX named FoxLOX. This enzyme was determined to act as a regular 13-LOX when LA or GLA were used as substrates; however, further metabolism of ALA-derived hydroperoxy products has been observed. Among the products formed from ALA, an hydroxy-epoxide (9(*Z*),15(*Z*)-11-OH-12(*S*)(13(*S*))-*trans*-EpODE), a ketone (13-oxo-OTrE), and two diols (10(*E*),12(*Z*),14(*E*)-9,16-DiHOTrE and 15,16-DiHOTrE) have been identified.^283^

A fungal catalase (Fg-cat) from the fungus *Fusarium graminearum*, possessing a 13(*S*)-peroxidase activity, has been identified by Teder et al. This new enzyme is responsible for the production of a keto-epoxide from LA (10(*E*)-9-oxo-12(13)-*cis*-EpOME), which is generated as the main product from 13(*S*)-HpODE, with the *trans*-epoxide detected as a minor product. Fg-cat can also oxidize ALA into 13(*S*)-HOTrE, which is further metabolized into a keto-epoxide (10(*E*),15(*Z*)-9-oxo-12(13)-EpODE) as well as a diepoxide (10(*E*)-9-oxo-12(13);15(16)-DiEpOME).^284^

**Scheme 30:**
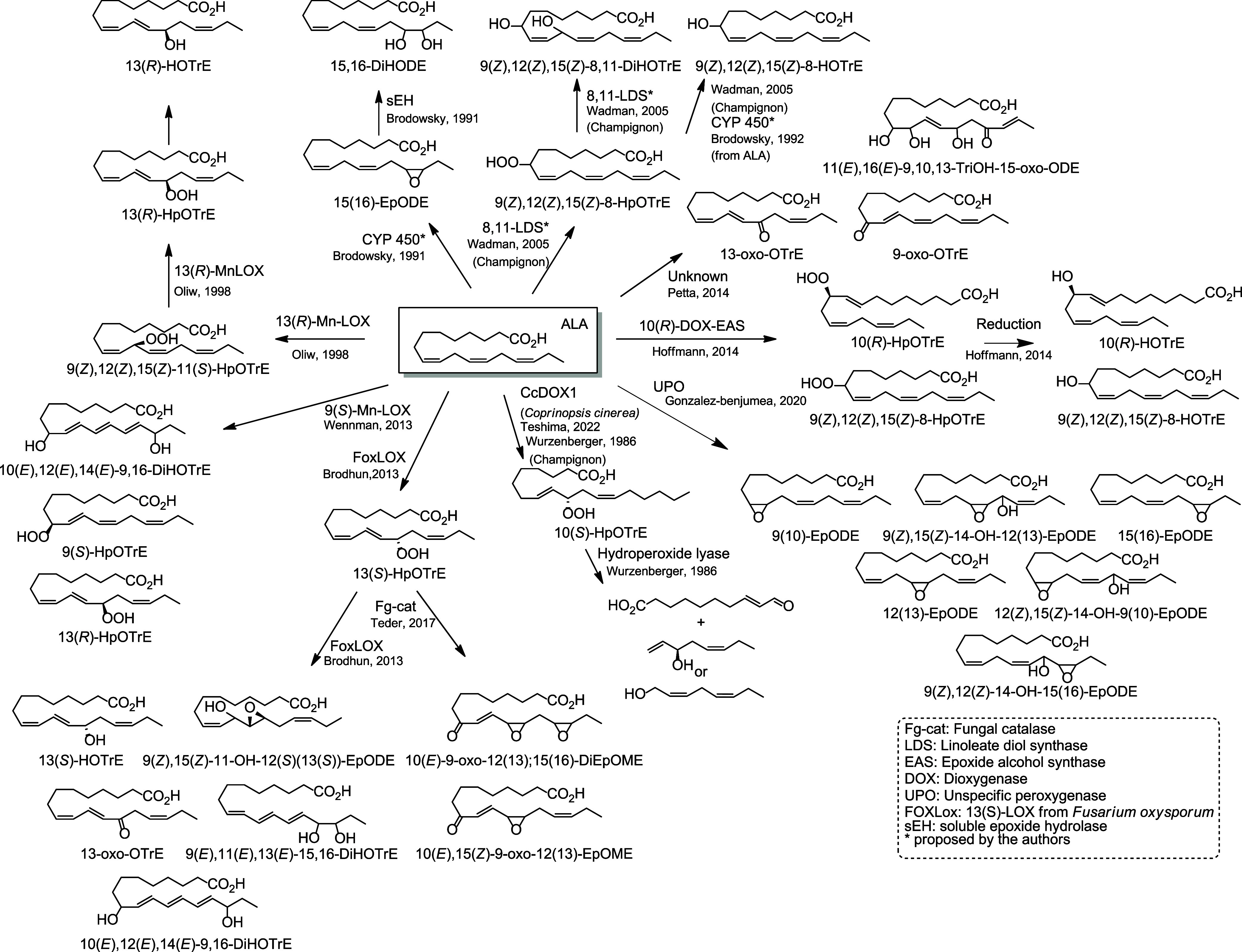
α-Linolenic Acid (ALA)-Derived Octadecanoids Produced by Fungal Enzymes Nomenclature is as described in Scheme 3. Synthetic route is referenced if known.

Finally, fungi possess unspecific peroxygenases that metabolize PUFAs into epoxides. OA is an excellent substrate for these enzymes and is oxidized into 9(10)-EpODA (main product). ALA is metabolized into monoepoxides and 14-OH-EpODEs, by the UPO enzyme of *Collariella virescens*.^285^ LA is a poorer substrate for this enzyme, but can also be oxidized into 9(*Z*)-11-HOME and 12(13)-EpOME (main products) as well as 9(10)-EpOME and 9(*Z*)-11-OH-12(13)-EpOME in *C. virescens*.^285^ Production of 9-oxo-OTrE and 13-oxo-OTrE from ALA by the fungus *Aspergillus niger* has been described by Petta et al. in 2014, as well as the production of a unique metabolite (11(*E*),16(*E*)-9,10,13-TriOH-15-oxo-ODE). However, the enzymes involved in the production of these three metabolites have not been investigated (Scheme 29 and Scheme 30).^82^

**Scheme 31:**
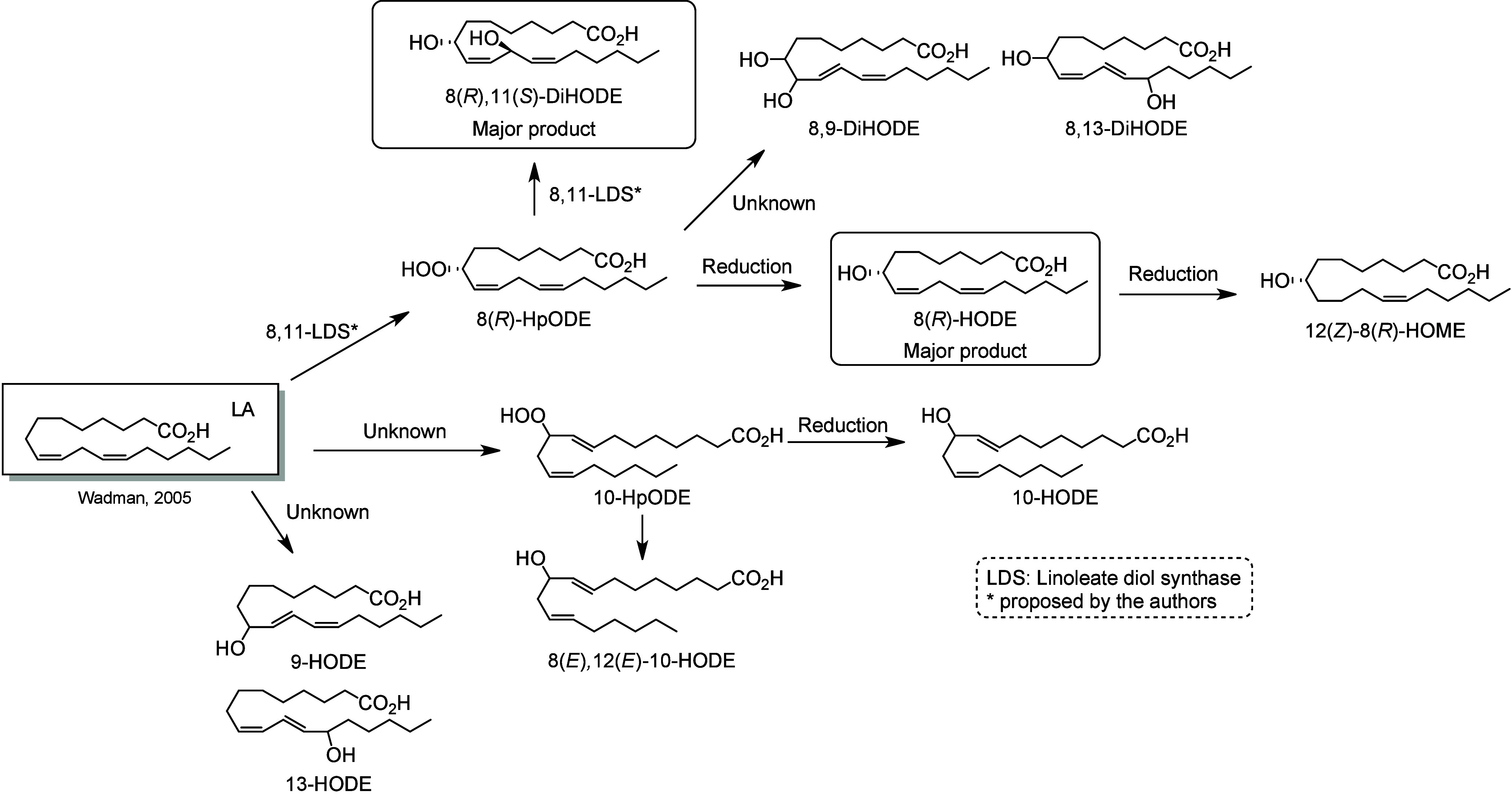
Linoleic Acid (LA)-Derived Octadecanoids Produced by Champignon (*Agaricus bisporus* or *Psalliota bispora*) Nomenclature is as described in Scheme 3. Synthetic route is referenced if known.

In the commonly eaten champignon (*Agaricus bisporus* also called *Psalliota bispora*), several unique metabolites were discovered, but the enzymes responsible for their formation remain unknown. Identified LA metabolites include 9(*Z*),12(*Z*)-8(*R*)-HODE and 9(*Z*),12(*Z*)-8(*R*),11(*S*)-DiHODE as the main products, and 8(*E*),12(*E*)-10-HODE, 10(*E*),12(*Z*)-8,9-DiHODE and 9(*Z*),11(*E*)-8,13-DiHODE, 12(*Z*)-8(*R*)-HOME, 9-HODE and 13-HODE as secondary products. OA, ALA and GLA are also converted into their 8-hydroxy derivatives and, in the case of ALA, into the nonvicinal diol 9(*Z*),12(*Z*),15(*Z*)-8,11-DiHOTrE (Scheme 31).^271^

## 6 Nonenzymatic Biosynthesis of Octadecanoids

In addition to enzymatic biosynthetic pathways described in the previous chapter, a high diversity of octadecanoids can be formed nonenzymatically by the action of free radicals or small reactive molecules such as singlet oxygen.^286^ Endogenous sources of reactive oxygen include the CYPs,^287^ xanthine oxidoreductase,^288^ NADPH oxidases,^289^ flavoproteins, myeloperoxidase, as well as normal mitochondrial function as a consequence of cellular respiration.^290^ The release of ROS by NADPH oxidase is stimulated by saturated^291^ and unsaturated C18-FAs.^292,293^

If dysregulated by external (e.g., exposure to pollution or smoking) or internal (e.g., neurodegenerative or inflammatory) insults, activation of these systems can lead to oxidative stress with diverse ramifications. For instance, within the mitochondria, increased ROS production leads to the oxidation of the LA rich cardiolipin, and subsequent phospholipase activity can release an array of species including oxo-OMEs, HODEs, EpOMEs, HpODEs, DiHODEs, and Hp-oxo-ODEs.^294^ Moreover, radical leakage into tissues can oxidize proteins, lipids, and DNA, leading to their degradation.^295^ Most of these radicals are ROS such as hydroxyl radical (HO^•^), the most reactive radical for peroxidation of PUFAs. ROS comprise the nonradicals singlet oxygen and hydrogen peroxide, and encompass the highly reactive hydroxyl radical (HO^•^), superoxide radical (O^2•–^), and fatty acid peroxyl and alkoxyl radicals that strongly enhance the propagation of lipid peroxidation. Superoxide radical, by itself a weak oxidant, participates in the Fenton reaction,^296^ and in the formation of ^1^O_2_ and of H_2_O_2_ by spontaneous dismutation.^297,298^

The chemical nature of LA oxidation products was studied principally by Gardner’s group in the 1970s and early 1980s. Radical reaction of oxygen with PUFAs has been extensively reviewed by Gardner in 1989,^299^ and the nature and formation mechanisms of nonenzymatic LA metabolites has been reviewed by Spiteller in 1998.^300^ Linear products as well as endoperoxides are formed from LA, whereas cyclic metabolites such as phytoprostanes or phytofurans can be formed from ALA, GLA, and SDA, which possess 2 or more *bis*-allylic carbons.^102^ The first step of lipid peroxidation is the abstraction of a *bis*-allylic hydrogen, generating a stabilized pentadienyl radical. After radical mesomeric rearrangement, a dioxygen molecule is trapped by the conjugated radical to generate a peroxyl radical. Hydrogen abstraction by the peroxyl radical forms a hydroperoxide that can be reduced into a hydroxy group by glutathione peroxidase or a metal complex (Scheme 49, orange path).^301−304^ As opposed to enzymatic oxidation, which generates enantiopure hydroperoxides, the action of free radicals on PUFAs is not stereoselective and generates racemic mixtures.^286^ Nonenzymatic abstraction of a *bis*-allylic hydrogen on LA generates 9-HpODE and 13-HpODE,^305^ and 9-HpOTrE, 12-HpOTrE, 13-HpOTrE, and 16-HpOTrE are formed from ALA.^306^ Even if not yet reported in the literature, the same reactions can occur with other C18-PUFAs including GLA, forming 6-, 9-,10-, and 13-HpOTrE-γ, and SDA, forming 6-,9-,10-,12-, 13-, and 16-HpOTE. Similarly, the abstraction of a hydrogen on carbons 8 and 11 of OA generate 8-HpODA, 9-HpODA, 10-HpODA, and 11-HpODA.^177,307^

**Scheme 32:**
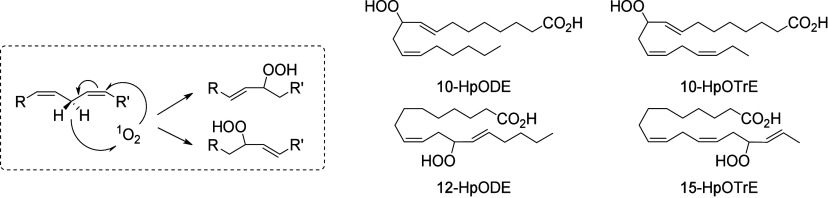
Specific Octadecanoid Products of Linoleic Acid (LA) and α-Linolenic Acid (ALA) Photosensitized Oxidation

Nonenzymatic oxidation can also be initiated by nonradical ROS species such as singlet oxygen. Singlet oxygen (^1^O_2_) is an excited state of dioxygen that can be generated by photoactivation. Singlet oxygen is a highly electrophilic species and can easily react with alkene moieties to produce hydroperoxides via an ene-reaction (Scheme 32).^308,309^ PUFAs can be oxidized by singlet oxygen, resulting in the formation of hydroperoxides in a process called photosensitized oxidation. Reaction of singlet oxygen with LA in mice skin creates four hydroperoxides, (9-HpODE, 10-HpODE, 12-HpODE, and 13-HpODE), among which 10-HpODE and 12-HpODE are specific products of singlet oxygen oxidation.^310^ Photosensitized oxidation of ALA generates 9-HpOTrE, 10-HpOTrE, 12-HpOTrE, 13-HpOTrE, 15-HpOTrE, and 16-HpOTrE, among which terminal hydroperoxides (9-HpOTrE and 16-HpOTrE) are favored. 10-HOTrE and 15-HOTrE are not generated by autoxidation of ALA.^311^ OA, which does not possess any *bis*-allylic hydrogen, is less sensitive to autoxidation but can undergo photosensitized oxidation by singlet oxygen to produce 9-HpOME and 10-HpOME.^310^

Hydroperoxides can be transformed by the same enzymatic systems responsible for the production of octadecanoid oxylipins, as previously described, but can also undergo specific nonenzymatic oxidation. This results in a high diversity of octadecanoids, possessing both linear and cyclic structures. Among the former, mono-, di-, and trihydroxy groups, ketones, hydroxy-ketones, mono-, and diepoxides, keto-epoxides and hydroxy-epoxides have been described.^301−304,306^

Reactive nitrogen species (RNS), such as nitrogen dioxide radical (^•^NO_2_) created from nitric oxide radical (^•^NO) in the presence of oxygen, or peroxynitrite (ONOO^–^) created from ^•^NO in the presence of superoxide radical, are also able to react with mono-FAs and PUFAs to produce nitro-FAs.^312^ While peroxynitrite can initiate lipid peroxidation reactions,^312^^•^NO can terminate such reactions,^313^ and ^•^NO_2_ can react directly with conjugated fatty acids to produce various nitro fatty acids including NODEs and NOTrEs.^314^ Notably, multiple molybdopterin-based nitrate reductases have been identified including xanthine oxidoreductase (XOR), aldehyde oxidase (AO) and sulfite oxidase (SO), which are capable of reducing NO_2_^–^ to ^•^NO in low pH anoxic microenvironments as found in hypoxic inflammatory sites.^315^ In addition, hemoglobin has also been reported to execute the NO_2_^–^ to ^•^NO conversion under hypoxic conditions.^316^ Therefore, it is important to realize that while the classic arginine-dependent production of ^•^NO is dominant when oxygen tensions are high, when oxygen tension is low alternate NO_2_^–^-dependent mechanisms are available and may contribute to nitro-FA production.

### 6.1 Linear Metabolites

#### 6.1.1 Alcohols

**Scheme 33:**
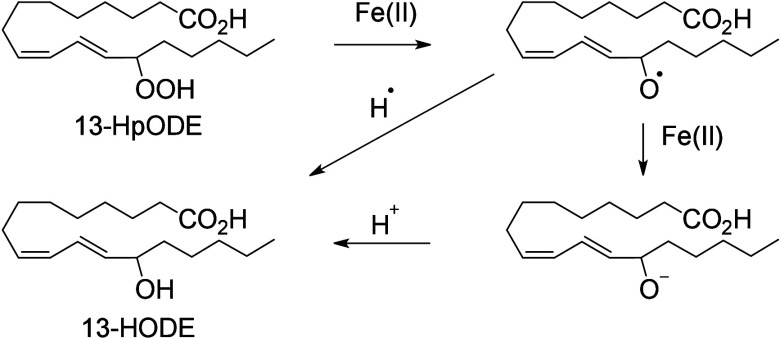
Reduction of Hydroperoxide into Alcohol

Hydroperoxides can be reduced into hydroxy groups in two steps in the presence of a metal. First, an alkoxyl radical is formed by reduction of hydroperoxides by a variety of free metals or metal complexes (e.g., ferrous chloride) and metalloproteins (e.g., hemoglobin and myoglobin).^298,300^ The alkoxyl radical can then abstract a hydrogen to generate a hydroxylated FA.^317^ Alternatively, an electron transfer from Fe(II) can directly form an alkoxyl anion which generates a hydroxy group after protonation (Scheme 33).^300^

The primary autoxidation (and enzymatic) products of LA (9- and 13-HpODE) were originally characterized by Nobelist Sune Bergstrom and Ralph Holman in the 1940s.^318,319^ In detailed extension of this work, the formation of 10(*E*),12(*Z*/*E*)-9-HODE and 9(*Z*/*E*),11(*E*)-13-HODE by autoxidation of LA and formation of 8-HOME, 9-HOME, 10-HOME, and 11-HOME from OA was reported by Gardner et al. in 1974.^177^ Then, in 1977, Frankel described the generation of 9-HOTrE, 12-HOTrE, 13-HOTrE, and 16-HOTrE by autoxidation of ALA followed by NaBH_4_ reduction of the hydroperoxides.^306^

#### 6.1.2 Ketones/Hydroxy-ketones

**Scheme 34:**
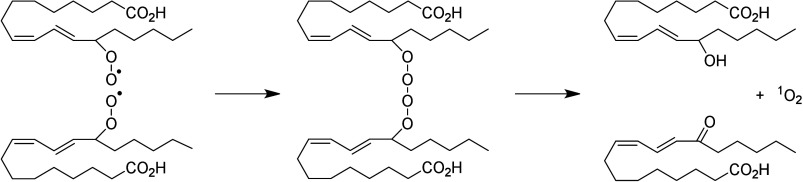
Nonenzymatic Formation of Ketones from a Peroxyl Radical

Ketones are readily available from PUFA hydroperoxides *via* transformations of peroxyl or alkoxyl radicals. The classic “Russell mechanism” involves the combination of two peroxyl radicals followed by the elimination of singlet oxygen and formation of one alcohol and one ketone.^320^Scheme 34 illustrates the reactions of peroxyl radicals from 13-HpODE, combining to form a linear tetroxide that decomposes to give 13-HODE, 13-oxo-ODE, and singlet molecular oxygen. The predictions of singlet oxygen formation have been tested over the years and although early experiments fell short in the quantitative yield of ^1^O_2_,^321^ later experiments have garnered more support for the Russell mechanism.^322,323^

**Scheme 35:**
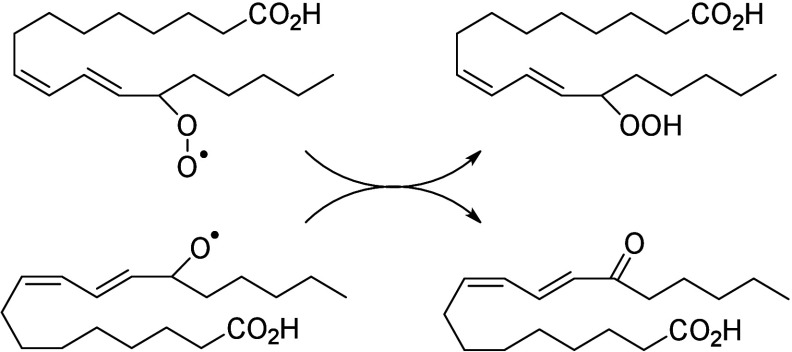
Formation of a Ketone and a Hydroperoxide from a Peroxyl and an Alkoxyl Radical

An interesting twist on the diradical combination is a proposed fusion of peroxyl and alkoxyl radicals to yield a ketone and regenerate a hydroperoxide (Scheme 35).^324^ The reactions occurred in the micro environment of lipid (linoleate) micelles, potentially bringing together these fleeting radical species. Radical–radical dismutation between a (*E*/*Z*)-linoleate alkoxyl radical and (*E*/*E*)-linoleate peroxyl radical explained the production of 9- and 13-(*E*/*Z*)-oxo-ODEs and (*E*/*E*)-HpODEs.^324^

**Scheme 36:**
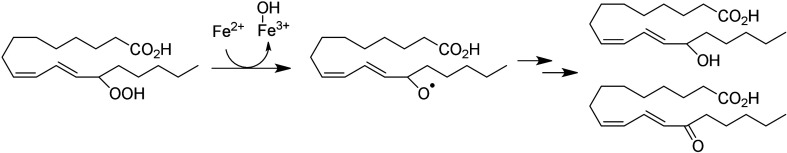
Formation of a Ketone from an Alkoxyl Radical

Alkoxyl radicals are generated by metal-dependent reduction of hydroperoxides and their further transformations lead to complex mixtures prominently featuring ketones and alcohols (Scheme 36). In 1974, Gardner et al. demonstrated the formation of 9(*Z*/*E*),11(*E*)-13-oxo-ODE, 10(*E*),12(*E*/*Z*)-9-oxo-ODE, 10(*E*)-9-OH-13-oxo-OME, and 11(*E*)-13-OH-9-oxo-OME from LA in the presence of cysteine and a catalytic amount of Fe (III), or equimolar amount of Fe(II), in ethanol, at room temperature.^177^ Zhu et al. reported that autoxidation of LA enhanced by Fe(II)/ascorbate under physiological condition (37 °C, pH 7.4) generates three main metabolites: a hydroxy-ketone possessing a *trans*-unsaturation (11(*E*)-10-OH-13-oxo-OME), a dihydroxy-ketone (11(*E*)-9,10-DiOH-13-oxo-OME), and an all-*trans* ketone (9(*E*),11(*E*)-13-oxo-ODE).^325^

**Scheme 37:**
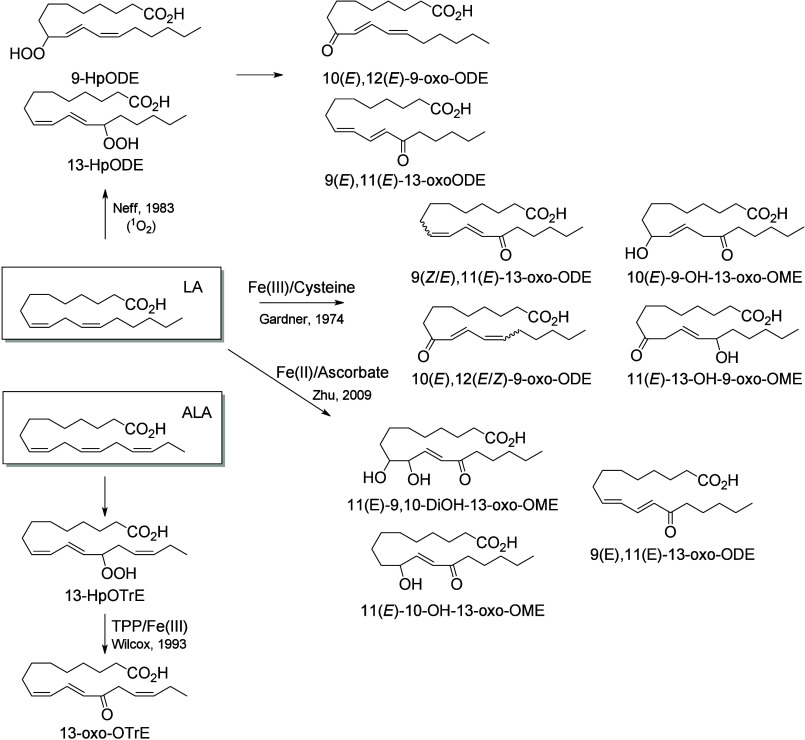
Nonenzymatic Production of Octadecanoid Ketone and Hydroxy-Ketone Metabolites of Linoleic Acid (LA) and α-Linolenic Acid (ALA) Nomenclature is as described in Scheme 3. Synthetic route is referenced if known.

Marnett and co-workers studied these mechanisms and the ensuing products by hematin-catalyzed degradation of oleic, linoleic and linolenic hydroperoxides.^298,326,327^ They noted that “When a double bond is β to the (alkoxyl) radical, the principal fate is loss of the α-H to form a ketone.”^298,326,327^ The transformations of 13-HpODE gave multiple epoxy derivatives plus 13-HODE and 13-oxo-ODE.^327^ Nonenzymatic oxidation of 13-HpOTrE into a small amount (7%) of 13-oxo-OTrE at room temperature in dichloromethane and in the presence of 5,10,15,20-tetraphenyl-21*H*,23*H*-porphyrin iron(III) chloride (TPP/Fe(III)) and 2,4,6-tri-*tert*-butylphenol, which generate an alkoxyl anion from the hydroperoxide, has been reported by Wilcox et al.,^298^ whereas the formation of a small amount (1.2%) of all-*trans* ketones (10(*E*),12(*E*)-9-oxo-ODE and 9(*E*),11(*E*)-13-oxo-ODE) by photosensitized oxidation of LA-derived 9- and 13-HpODEs has been described by Neff in the presence of methylene blue at 0 °C in dichloromethane (Scheme 37).^328^

#### 6.1.3 Diols

**Scheme 38:**
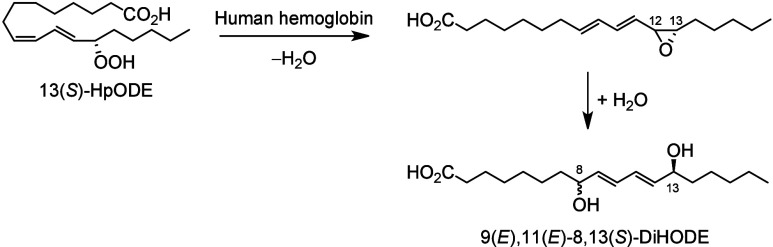
Principal Mechanism for the Nonenzymatic Formation of Nonvicinal Diols Nomenclature is as described in Scheme 3.

In 1983, Hamberg reported on the hemoglobin-catalyzed transformation of 13(*S*)-HpODE to a leukotriene A-type epoxide, 8(*E*),10(*E*)-12(13(*S*))-EpODE, with its rapid nonenzymic hydrolysis on the 8-carbon to two 8,13-dihydroxy diastereomers (Scheme 38).^329^ In analyzing the autoxidation of LA, Hamberg followed up on these findings by identifying four dihydroxy derivatives with conjugated dienes (two 8,13-diols and two 9,14-diols), postulated as the products from 9-HpODE and 13-HpODE.^330^ Subsequently, an equivalent enzymic transformation of 9(*R*)-HpODE to an allylic 9(10)-epoxide and its hydrolysis to 9,14-diols was shown to be catalyzed by an *Anabaena* catalase-LOX fusion protein.^331^

In 1982, Frankel et al. reported the formation of small amount of 9,10-DiHODE, 9,12-DiHODE, 9,13-DiHODE, 10,12-DiHODE, 10,13-DiHODE, and 12,13-DiHODE (0.3–5.3 wt % depending on peroxide value) as secondary products of photosensitized oxidation of LA’s 9- and 13-HpODEs at 0 °C in dichloromethane and in the presence of methylene blue.^332^ The formation of 9,14-DiHpODE from 9-HpODE and 8,13-DiHpODE from 13-HpODE in the presence of singlet oxygen and methylene blue at 0 °C in dichloromethane has also been observed by the same group in 1983, and represent 17.0% of total products.^328^

**Scheme 39:**
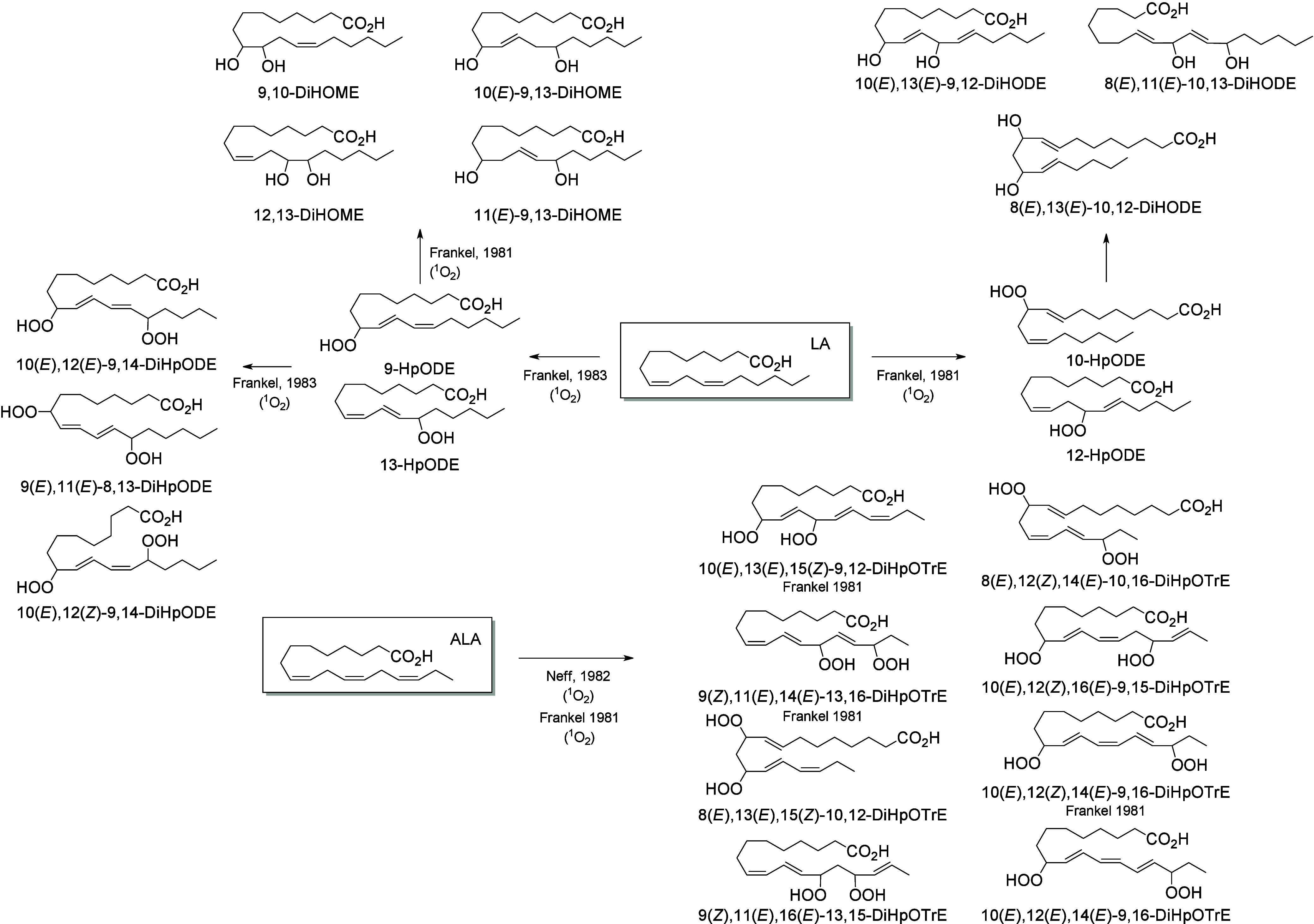
Nonenzymatic Diol Metabolites of Linoleic Acid (LA) and α-Linolenic Acid (ALA) Nomenclature is as described in Scheme 3. Synthetic route is referenced if known.

In 1982, Neff et al. described the photosensitized oxidation of ALA into a mixture of different dihydroperoxides: 10(*E*),13(*E*),15(*Z*)-9,12-DiHpOTrE, 9(*Z*),11(*E*),14(*E*)-13,16-DiHpOTrE, 8(*E*),13(*E*),15(*Z*)-10,12-DiHpOTrE, 9(*Z*),11(*E*),16(*E*)-13,15-DiHpOTrE, 8(*E*),12(*Z*),14(*E*)-10,16-DiHpOTrE, 10(*E*),12(*Z*),16(*E*)-9,15-DiHpOTrE, 10(*E*),12(*Z*),14(*E*)-9,16-DiHpOTrE, and 10(*E*),12(*E*),14(*E*)-9,16-DiHpOTrE. These dihydroperoxides have been identified as major secondary products of ALA oxidation in the presence of singlet oxygen.^333^ The 9,12-, 13,16-, and 9,16-dihydroperoxides may be formed from the 9- and 16-hydroperoxides by the same mechanism suggested for autoxidized methyl linoleate that proceeds through pentadienyl radicals. The other dihydroperoxide products (1,3-disubstituted and 1,7-disubstituted) are created by concerted addition of singlet oxygen on monohydroperoxides. An overview of the described diols formed from LA and ALA is provided in Scheme 39.

#### 6.1.4 Epoxides/Hydroxy-epoxides/Triols

**Scheme 40:**
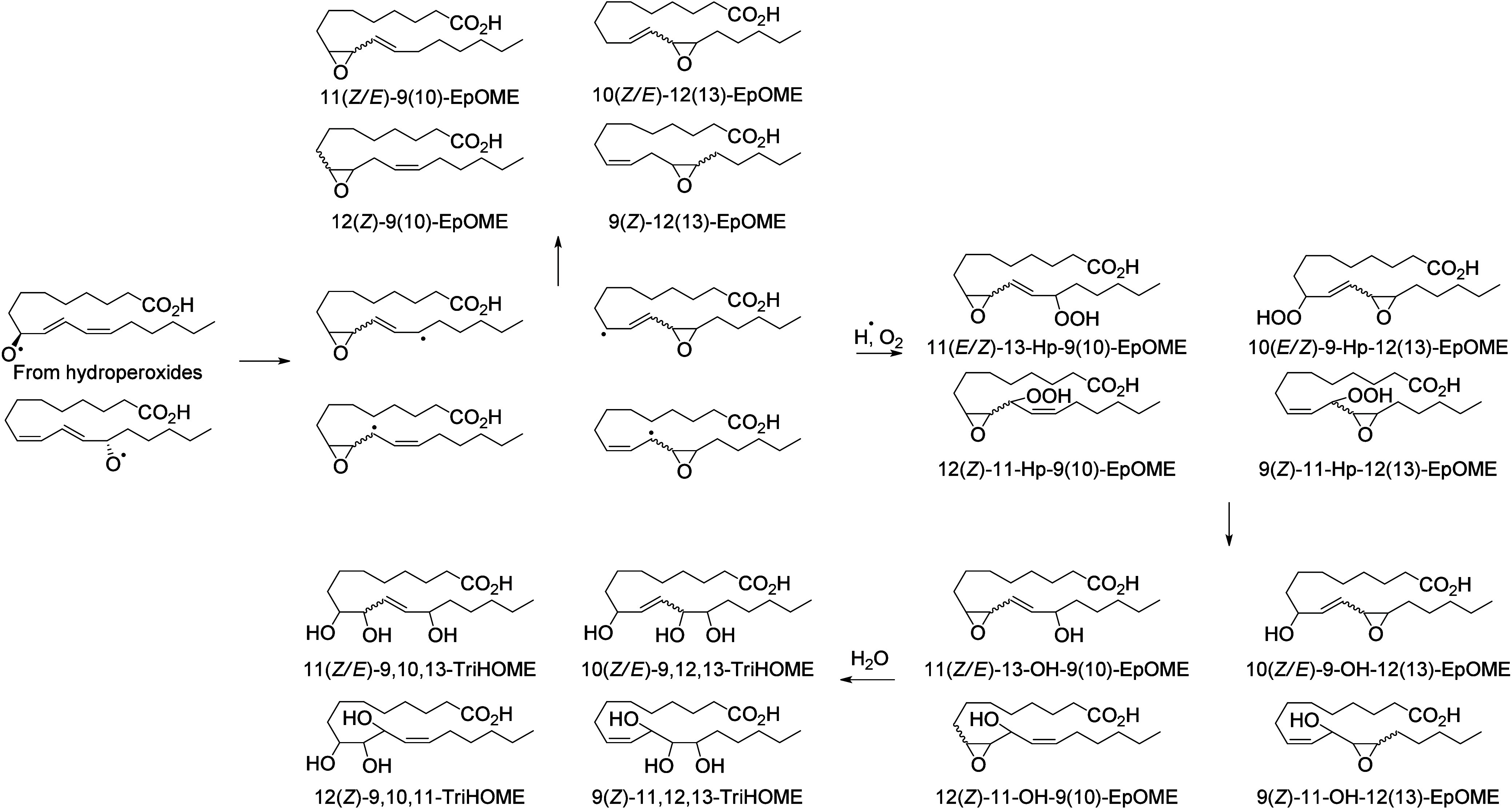
Mechanism of Nonenzymatic Biosynthesis of Octadecanoid Triols from Alkoxyl Radicals Nomenclature is as described in Scheme 3. Synthetic route is referenced if known.

In 1973, Hamberg et al. first reported the autoxidation of 13-HpODE into 9(*Z*)-11-OH-12(13)-*trans*-EpOME by homolysis of the hydroperoxide, formation of an epoxide, and attack by a water molecule.^334^ More recently, these hydroxy-epoxide compounds have been shown to be generated from an alkoxyl radical, which tends to rearrange into more stable mesomeric epoxy-allylic radicals, even in the presence of compounds with a readily abstractable hydrogen.^335,336^ These radicals can perform hydrogen abstraction to form epoxides or, alternatively, they can also be scavenged by oxygen to form hydroperoxy-epoxides after hydrogen abstraction.^337^ In 1981, Gardner et al. described formation of 9(10)-EpOME and 12(13)-EpOME following this mechanism, in the presence of FeCl_3_/Cys to generate the alkoxyl radical. The formation of 10(*E*)-9-Hp-12(13)-*trans*-EpOME from 13(*S*)-HpODE in the presence of Fe(III)/cysteine has also been reported.^338^ Cleavage of the hydroperoxide followed by hydrogen abstraction generates hydroxy-*trans*-epoxide compounds that can be hydrolyzed into triols (Scheme 40).^335^

**Scheme 41:**
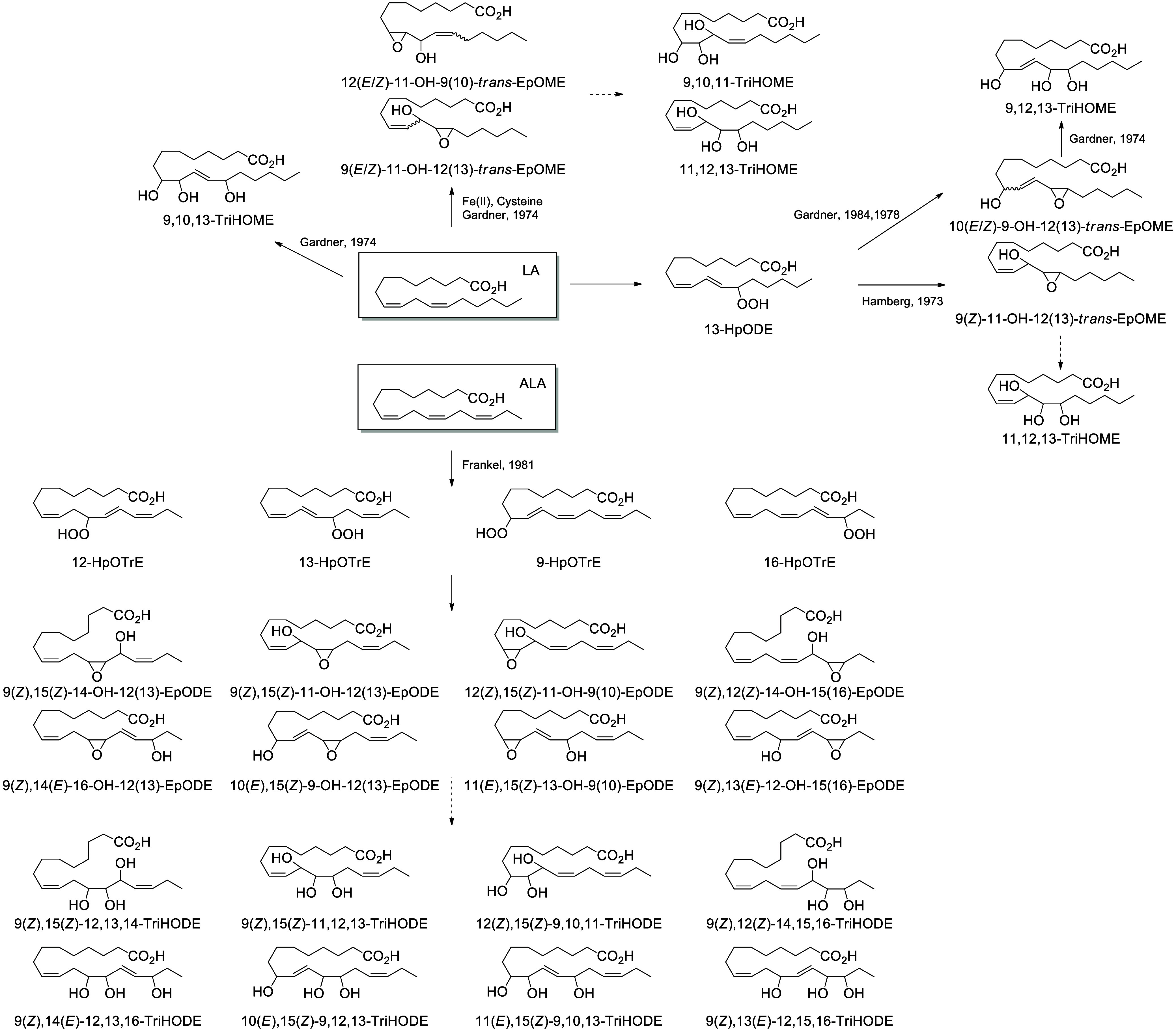
Hydroxy-epoxide and Triol Nonenzymatic Octadecanoids Derived from Linoleic Acid (LA) and α-Linolenic Acid (ALA) Nomenclature is as described in Scheme 3. Synthetic route is referenced if known. The dotted arrows refer to the formation of compounds for which a primary citation could not be identified in the literature.

In the presence of Fe(III) and cysteine, Gardner et al. showed that oxidation of LA results in the formation of 9(*Z*)-11-OH-12(13)-*trans*-EpOME, 12(*Z*)-11-OH-9(10)-*trans*-EpOME, 9(*E*)-11-OH-12(13)-*trans*-EpOME, and 12(*E*)-11-OH-9(10)-*trans*-EpOME as well as the triols 10(*E*)-9,12,13-TriHOME and 11(*E*)-9,10,13-TriHOME.^177^ In 1978, the formation of 10(*E*)-9-Hp-12(13)-*trans*-EpOME from 13-HODE was reported by the same group.^337^ Then, in 1981, they showed that these products are generated from monohydroperoxides. Indeed, 11(*E*),15(*Z*)-9-OH-12(13)-*trans*-EpODE can be generated by decomposition of 13-HpOTrE. Decomposition of 13-HpODE or 9-HpODE in the presence of FeCl_3_/Cys resulted in formation of 9(*Z*)-*erythro*/*threo*-11-OH-12(13)-*trans*-EpOME, and 12(*Z*)-*erythro*/*threo*-11-OH-9(10)-*trans*-EpOME as well as lesser amount (about 15–25%) of the (*E*)-isomer (Scheme 41).^335^

#### 6.1.5 Keto-epoxides

**Scheme 42:**
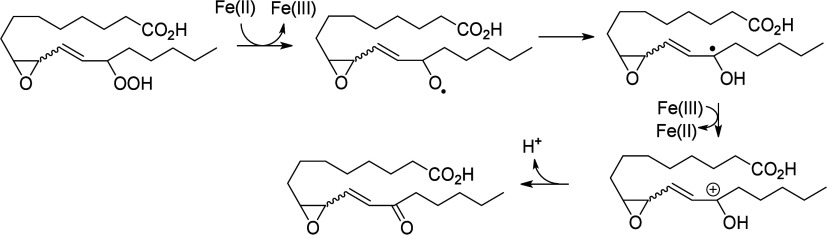
Proposed Mechanism for the Nonenzymatic Formation of Keto-epoxides

Keto-epoxides are mainly formed from hydroperoxy-epoxides via alkoxyl radical formation. The first step of the proposed mechanism is the formation of an alkoxyl radical by a metal-dependent reduction of the hydroperoxide, followed by a rearrangement which gives a hydroxy group possessing a radical in the α-position. This radical electron is trapped by a ferric ion to form a cation which, after deprotonation of the alcohol, forms a ketone. (Scheme 42).^177,300,335−337^

**Scheme 43:**
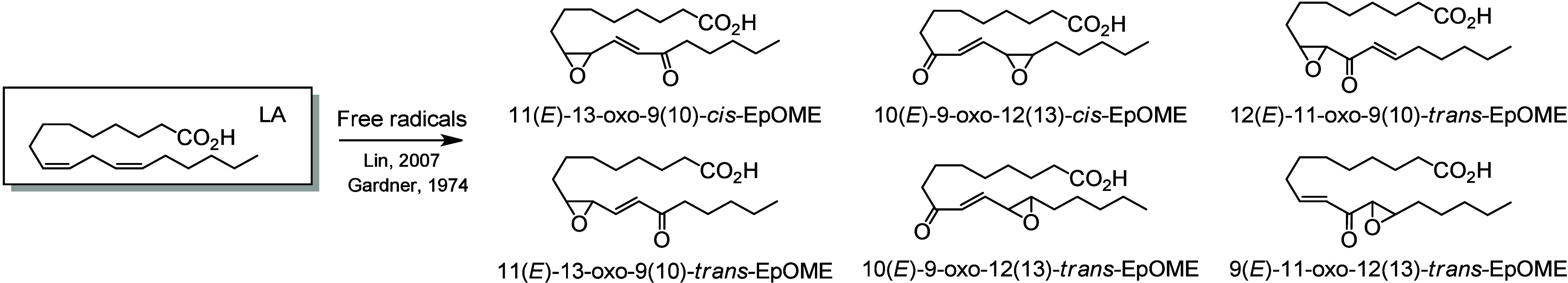
Nonenzymatic Formation of Keto-epoxide Metabolites from Linoleic Acid (LA) Nomenclature is as described in Scheme 3. Synthetic route is referenced if known.

Another reported mechanism describes the formation of the ketone via cleavage of the hydroperoxide into an alkoxyl radical that can degrade into a ketone.^335^ In the presence of FeCl_3_/Cys, Gardner et al. showed the formation of 11(*E*)-13-oxo-9(10)-*trans*-EpOME, 11(*E*)-13-oxo-9(10)-*cis*-EpOME, 10(*E*)-9-oxo-12(13)-*trans*-EpOME, and 10(*E*)-9-oxo-12(13)-*cis*-EpOME by autoxidation of LA.^177^ In 1981, the same group showed that, in the presence of FeCl_3_/Cys, 10(*E*)-9-oxo-12(13)-*trans*-EpOME, or 11(*E*)-13-oxo-9(10)-*trans*-EpOME are formed from 13-HpODE and 9-HpODE, respectively, in ∼18% overall yield. The *cis*-epoxides are produced as well but in lower yield (5% overall).^335^ The formation of these four keto-epoxyoctadecenoic acids possessing a *trans*-double bond was shown by Lin et al., simultaneously with two new keto-epoxy-metabolites by autoxidation of LA promoted by Fe(II)/ascorbic acid (Scheme 43).^339^

#### 6.1.6 Nitro-FAs

When the enzymatic production of superoxide (e.g., NADPH oxidases) and ^•^NO occur in close proximity, these species can combine to form peroxynitrite, which can further react with carbon dioxide to yield ^•^NO_2_.^340^ Under normoxic conditions, nitric oxide synthase is the primary source of ^•^NO; however, under hypoxic conditions, nitrite is reduced to ^•^NO by deoxygenated myoglobin, xanthine oxidoreductase, and other systems.^341^ Nitrated fatty acids (NO_2_-FA) are the best-known products of RNS and are endogenous signaling molecules. They are formed by the attack of nitrogen dioxide radical (^•^NO_2_) or nitronium cation (NO_2_^+^) on unsaturated FAs, leading to the introduction of a nitro group on any position of the double bonds.^342,343^ The mechanism of formation of the NO_2_-FAs is still not known with certainty, because several biosynthetic routes can form these metabolites.

**Scheme 44:**
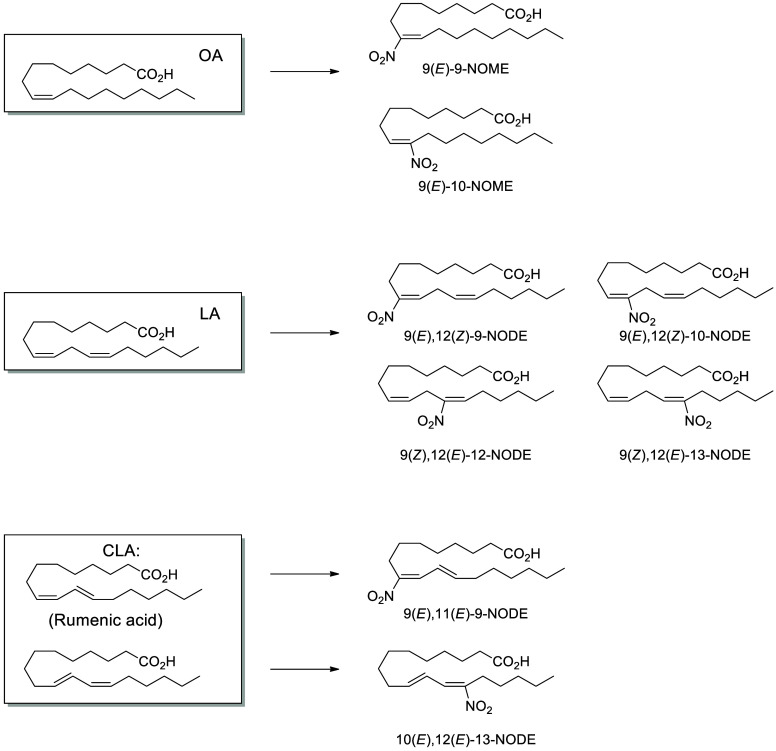
Nitroalkene Metabolites of Oleic Acid (OA), Linoleic Acid (LA), and Conjugated Linoleic Acid (CLA)

The most commonly reported NO_2_-FAs identified in vivo are the nitroalkenes, such as nitro-oleic acids (NOMEs, 2 regioisomers, Scheme 44), nitro-linoleic acids ((*Z*,*Z*)-NODEs, 4 regioisomers, Scheme 44), and nitro-conjugated linoleic acids ((*E*,*Z*)-NODEs, 2 regioisomers, Scheme 44).^344,345^ Notably, conjugated linoleic acid appears to be the preferred substrate for these reactions^160^ and mechanisms associated with ^•^NO_2_ production are increased during inflammation and metabolic stress with both 9(*E*),11(*E*)-9-NODE and 9(*E*),11(*E*)-12-NODE being produced.^346^

Nitroalkenes can also be precursors of other nitrated species because they can undergo additional reactions with ROS and RNS to be further nitrated, and form nitroso, dinitroso, nitronitroso, di- and trinitro species. They can also be oxidized to generate nitrohydroxy, nitrohydroperoxy, nitro-epoxy, or nitro-keto.^160,347^

**Scheme 45:**
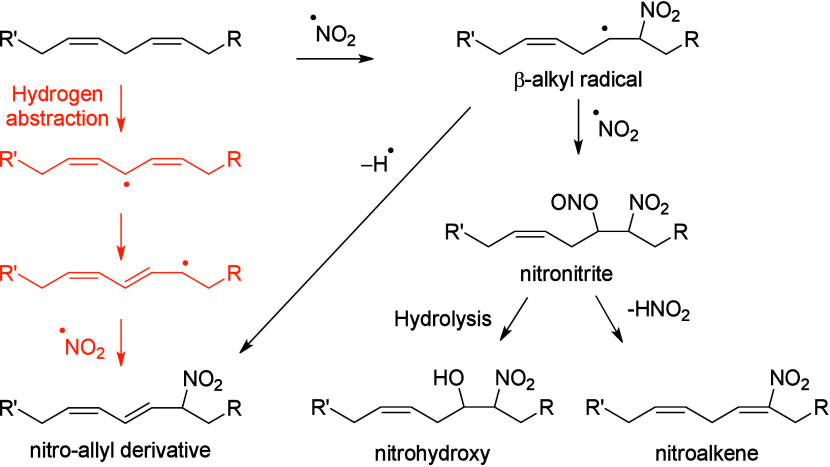
Formation of Nitro-allyl, Nitrohydroxy, and Nitroalkene The orange path indicates formation of nitro allyl derivatives through bis-allylic hydrogen abstraction.

Two mechanisms coexist for the synthesis of the nitroalkene metabolites. One of them is a radical mechanism that begins by a radical-induced nitration that generates a β-nitroalkyl radical. The abstraction of a hydrogen radical gives a nitro-allyl derivative. If the β-nitroalkyl radical undergoes a second addition of a ^•^NO_2_, a nitronitrite compound is obtained, and can be hydrolyzed into a nitrohydroxy fatty acid. It can also lose nitrous acid to give a nitroalkene.^346^ Formation of nitro allyl derivatives can also proceed through *bis*-allylic hydrogen abstraction, and reaction of the resulting stabilized pentadienyl radical with nitrogen dioxide radical (^•^NO_2_) (Scheme 45, orange path).^346^

**Scheme 46:**
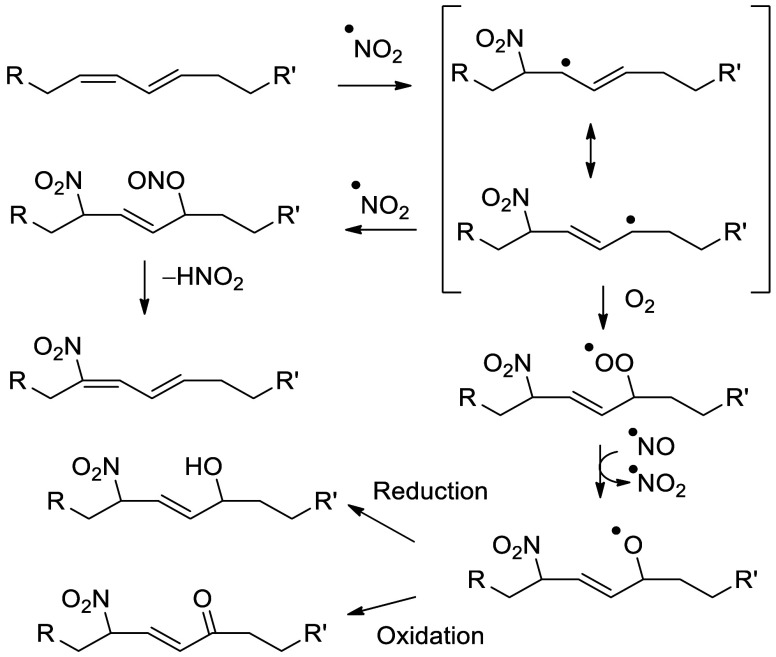
Formation of Nitroalkene, Nitrohydroxy, and Hydroketone from Conjugated Linoleic Acid (CLA)

Similarly, the formation of NO_2_-CLA starts by radical-induced nitration that generates an allylic radical. This radical can isomerize *via* resonance and reacts with a molecule of ^•^NO_2_ or ^•^O_2_ in the gamma position of the nitro group to form a nitronitrite intermediate or a nitro-peroxide intermediate. The elimination of the nitrite gives the nitroalkene, while the reduction of the peroxide into an alkoxyl radical gives a nitro-hydroxy metabolite by reduction of a nitro-oxo metabolite by oxidation (Scheme 46).

**Scheme 47:**
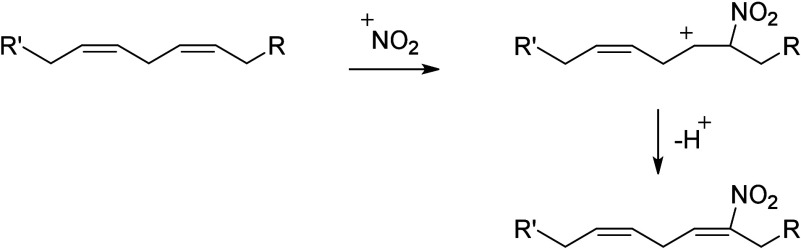
Formation of Nitroalkene via an Electrophilic Substitution Mechanism

The second mechanism for the formation of nitroalkene consists of an electrophilic substitution of a nitronium cation on a double bond. The nitronium cation can be formed by the reaction of a transition metal with peroxynitrite (Scheme 47).

### 6.2 Cyclic Metabolites

In addition to linear metabolites, three families of cyclic metabolites can be generated nonenzymatically from C18-PUFAs: endoperoxides and diendoperoxides, phytoprostanes (PhytoPs), and phytofurans (PhytoFs). These compounds are named in accordance with the eicosanoid classes isoprostanes and isofurans. Cyclic epoxides are discussed in the linear metabolites section. Endoperoxides can be generated from all PUFAs, while PhytoPs and PhytoFs are only generated from C18-PUFAs possessing at least three unsaturations (ALA, GLA, SDA). Differently from C20-PUFAs, for which cyclic metabolites can be also produced enzymatically by COX action, C18-PUFAs only produce cyclic metabolites via nonenzymatic oxidation. The resulting compounds contain 5-membered rings and can be created both via radical and singlet oxygen-dependent processes including a host of biologically mediated processes.^348^ Production of cyclic nonenzymatic metabolites of PUFAs, including octadecanoids, has been extensively studied and reviewed by Durand and colleagues.^102,103,349,350^

#### 6.2.1 Endoperoxides

The ^18^O_2_-labeling evidence by Samuelsson for an endoperoxide intermediate in prostaglandin biosynthesis^351^ and the subsequent isolation of PGG_2_ and PGH_2_^352^ sparked great interest in the occurrence of fatty acid endoperoxides and the mechanisms of their formation. Subsequent mechanistic studies were conducted by the Porter and Pryor laboratories using C18:3 substrates, undoubtedly due to the ready availability of these plant-derived fatty acids in the 1970s. Porter and Funk formed prostaglandin-like bicyclic endoperoxides by free radical chemistry, thus supporting the involvement of this mechanism in prostaglandin biosynthesis.^353,354^ The focus of the Pryor group was on implicating bicyclic endoperoxides as reactants in the widely used thiobarbituric acid method to detect and assay fatty acid oxidants in autoxidation.^355^ Interesting enzymological production of monocyclic endoperoxides was reported by Roza and Francke in their studies on the soybean lipoxygenase-catalyzed transformations of α-linolenate methyl ester.^356^ Using soybean flour and neutral pH conditions, the main products were the hydroxy-endoperoxides 16-hydroxyperoxy-13,15-endoperoxy-linolenate and 9-hydroxyperoxy-10,12-endoperoxy-linolenate. A chemical point of interest is the resistance of these monocyclic endoperoxide moieties to reduction by sodium borohydride; instead, for analytical purposes, the endoperoxides were opened to diols by hydrogenation using palladium on a carbon catalyst.^356^

Octadecanoid endoperoxides can be created via both radical and singlet oxygen-dependent processes. In 1980, the Chan and Mihelich groups reported for the first time the formation of 5-membered ring endoperoxide hydroperoxides by both photosensitized oxidation and autoxidation of LA.^357,358^ In 1981, Neff et al. separated and identified 13(*E*),15(*Z*)-10(12)-epidioxy-9-HpODE, 9(*E*),11(*E*)-13(15)-epidioxy-16-HpODE, and 9(*Z*),11(*E*)-13(15)-epidioxy-16-HpODE as major secondary products of autoxidized ALA.^359^ The same year, the group published a study concerning the photosensitized oxidation of LA and separated and identified the structures of the endoperoxides previously discovered by Mihelich. These products are diastereoisomeric pairs of 8(*E*)-10(12)-epidioxy-13-HpOME and 13(*E*)-10(12)-epidioxy-9-HpOME and are generated from specific singlet oxygen hydroperoxides (10-HpODE and 12-HpODE).^332^

The following year, the Neff and Chan groups showed that 6 endoperoxides already identified among the autoxidation products of ALA^359,360^ could be also produced by photosensitized oxidation and described the formation mechanisms.^333^ These products (13(*E*),15(*Z*)-10(*R*)(12(*S*))-epidioxy-9(*R*/*S*)-HpODE and 13(*E*),15(*Z*)-13(*S*)(15(*R*))-epidioxy-16(*R*/*S*)-HpODE and their enantiomers) are derived from 12-HpOTrE and 13-HpOTrE and represent 97% of the endoperoxides generated by photosensitized oxidation of ALA. The remaining 3% consisted of 8(*E*),15(Z)-10(12)-epidioxy-13-HpODE and 9(*Z*),16(*E*)-13(15)-epidioxy-12-HpODE, generated by cyclization of 10- and 15-HpOTrE. These endoperoxides tend to cyclize again to form the diendoperoxides 16(*E*)-10(*S*)(12(*R*));13(*S*)(15(*R*))-diepidioxy-9(*R*/*S*)-HpOME and 8(*E*)-10(*R*)(12(*S*));13(*R*)(15(*S*))-diepidioxy-16(*R*/*S*)-HpOME as well as their enantiomers, which were reported for the first time by Neff et al. in 1982.^333^ Therefore, 8(*E*),15(*Z*)-10(12)-epidioxy-13-HpODE and 9(*Z*),16(*E*)-13(15)-epidioxy-12-HpODE, as well as diendoperoxide metabolites, are specific products of singlet oxygen-dependent oxidation of ALA and are not formed by radical mediated autoxidation.

**Scheme 48:**
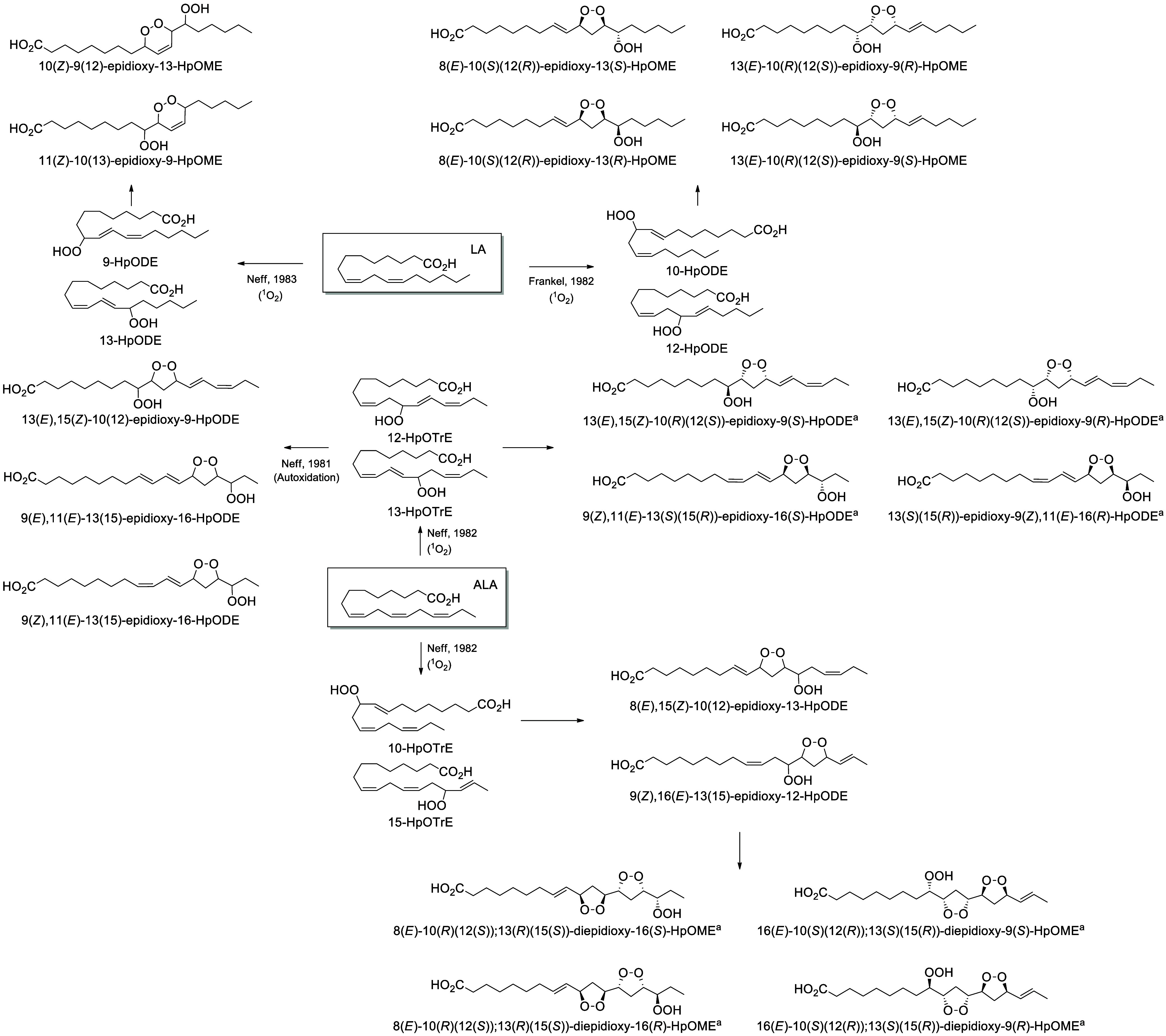
Formation of Endoperoxide Octadecanoids from Linoleic Acid (LA) and α-Linolenic Acid (ALA) Nomenclature is as described in Scheme 3. Synthetic route is referenced if known. ^a^The enantiomer is also generated.

In 1983, Neff et al. identified the 6-membered ring endoperoxides 10(*Z*)-9(12)-epidioxy-13-HpOME and 11(*Z*)-10(13)-epidioxy-9-HpOME as major products (57.9%) of the reaction of 9- and 13-HpODE with singlet oxygen.^328^ Six-membered hydroperoxy-epidioxides are formed by 1,4-addition of singlet oxygen to the 1,3-diene system of linoleate hydroperoxides (Scheme 48).^361^ The formation of 6-membered ring endoperoxides has been hypothesized by Frankel et al. to explain the formation of 9,10,12-TriHODE and 13,15,16-TriHODE from 12-HpOTrE and 13-HpOTrE, respectively.^306^

#### 6.2.2 Phytoprostanes

**Scheme 49:**
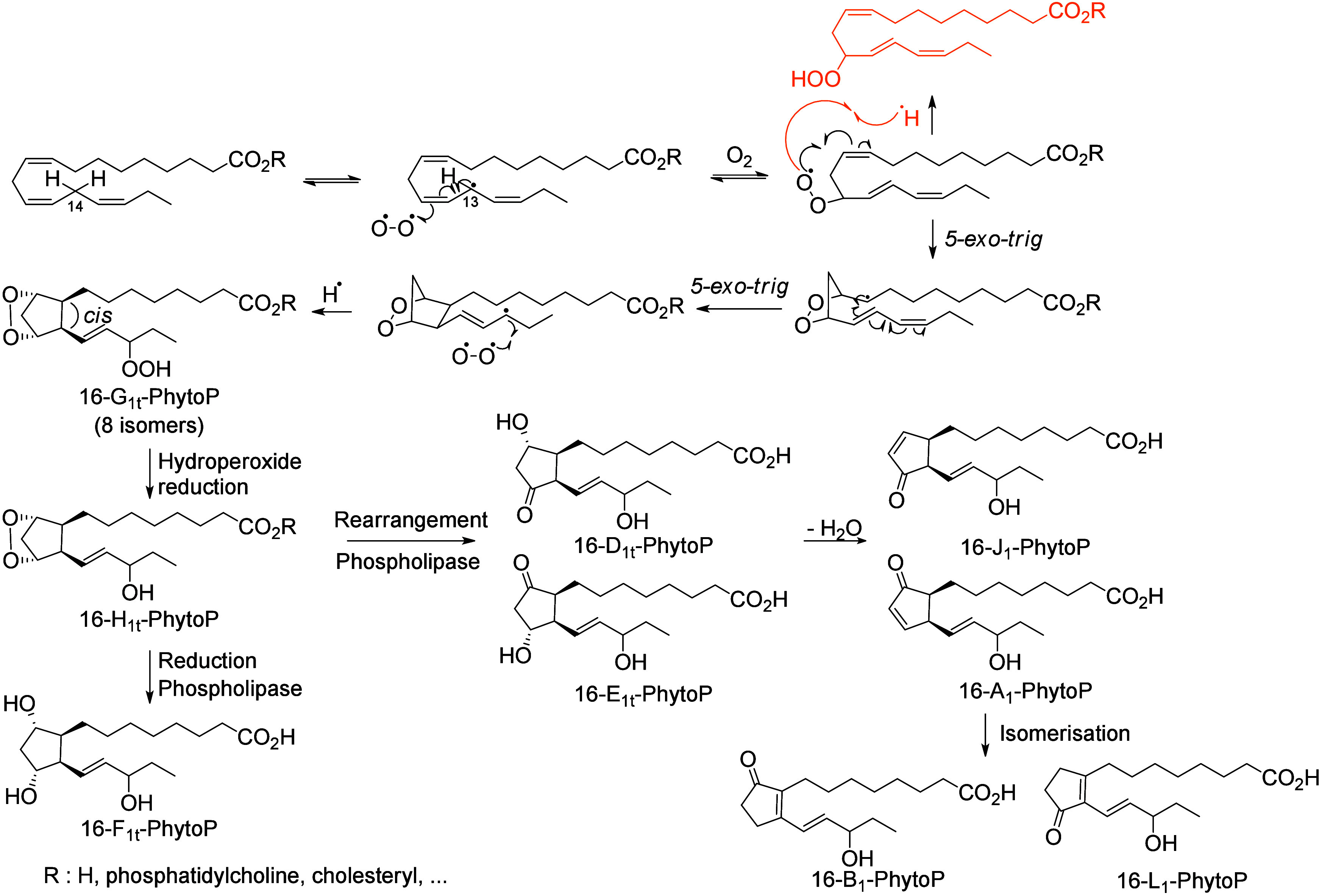
Nonenzymatic Biosynthesis of *trans*-PhytoP and Hydroperoxide Octadecanoids from α-Linolenic Acid (ALA) The orange color indicates the formation of linear hydroperoxide as a side reaction.

PhytoPs can be produced from all C18-PUFAs possessing at least three unsaturations (e.g., ALA, GLA, and SDA). PhytoPs are abundant in plant-based food^144^ but can also be synthesized in humans by direct nonenzymatic oxidation of these PUFAs. In 2009, a study published by Barden et al. showed that supplementation with flaxseed oil, an oil rich in ALA, leads to significant increases in F_1_-PhytoP in human plasma and urine.^362^ The biosynthesis mechanism of PhytoPs has not been determined directly but it has been inferred by analogy with isoprostanes (IsoPs).^102^ It initiates by the formation of a peroxyl by abstraction of a hydrogen radical on a *bis*-allylic position, followed by a radical rearrangement and trapping of a dioxygen molecule. The next step is a 5-*exo-trig* cyclization of the peroxyl that generates an endoperoxide.^363,364^ A second 5-*exo-trig* cyclization forms a bicyclic endoperoxide and creates mostly a *cis*-configuration between the two lateral chains.^365,366^ A second molecule of dioxygen is trapped to form a hydroperoxide that can be subsequently reduced together with the endoperoxide into hydroxy groups via the action of antioxidant species (e.g., glutathione or α-tocopherol), generating mostly type F-PhytoP.^367,368^ The hydroxy moieties on the ring can be in *cis*-configuration with the two lateral chains, creating the *cis*-PhytoPs (e.g., F_1c_-PhytoPs) or in a *trans*-configuration with the lipidic chains, creating *trans*-PhytoP (e.g., F_1t_-PhytoPs). In total, 8 isomers of PhytoPs can be generated from ALA. When the concentration of reducing agent is lower and H-PhytoP is present in an aqueous environment, type D- and E-PhytoPs are mostly formed via the rearrangement of H_1_-PhytoP.^352,369,370^ Dehydration of type D- and E-PhytoPs occurs easily under physiological conditions and creates type J- and A-PhytoPs analogous to the isoprostanes.^371^ Finally, type B- and L-PhytoP are obtained by isomerization of type A- and J- PhytoPs’ intracyclic double bond (Scheme 49).^372^

**Scheme 50:**
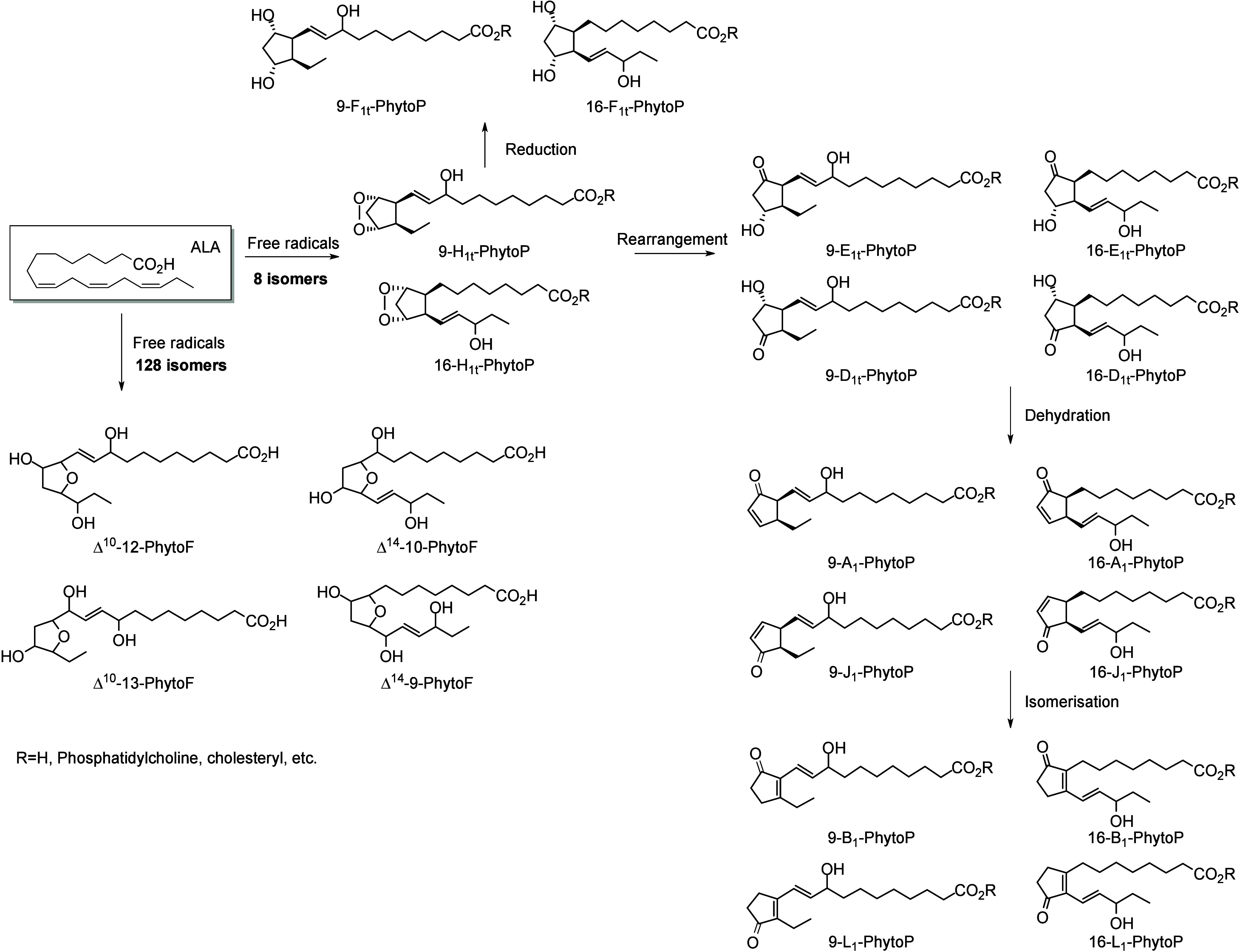
Nonenzymatic Production of α-Linolenic Acid (ALA)-Derived Cyclic Octadecanoids

**Scheme 51:**
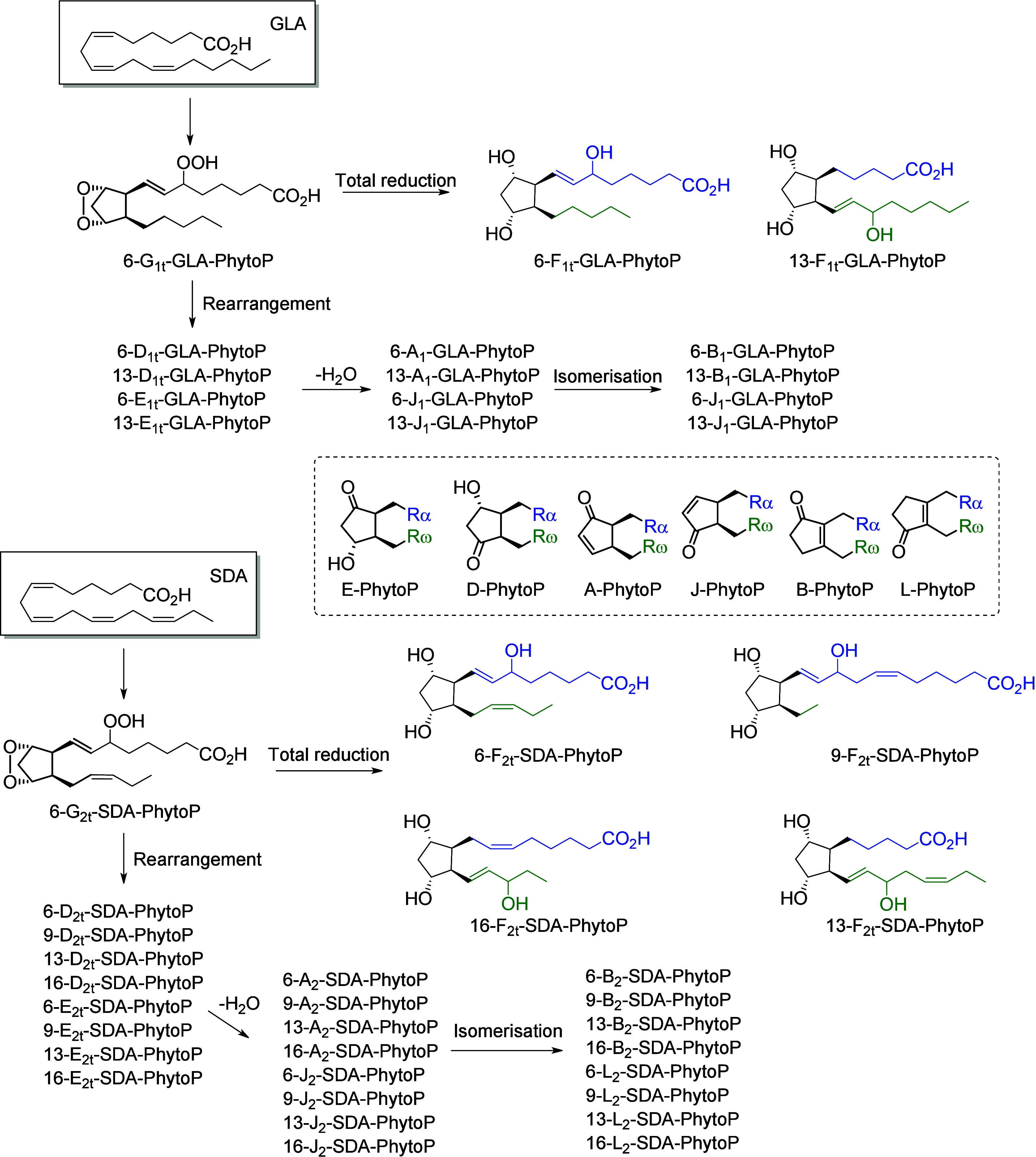
Putative Nonenzymatic Production of γ-Linolenic Acid (GLA) and Stearidonic Acid (SDA)-Derived Cyclic Octadecanoids The α-chain is displayed in blue color and the ω-chain in green color.

As ALA possesses two *bis*-allylic positions, two different families of PhytoPs can be formed, namely 9-PhytoPs (if the abstraction takes place on the 11-position) and 16-PhytoPs (if the abstraction takes place on the 14-position) (Scheme 50). GLA also possesses two *bis*-allylic positions, and the abstraction of a hydrogen radical on the 8-position generates the 6-GLA-PhytoPs, whereas the abstraction of a hydrogen radical on the 11-position forms 13-GLA-PhytoPs (Scheme 51). SDA possesses three *bis*-allylic positions, and four families of SDA-PhytoPs can be created. A *bis*-allylic hydrogen radical can be abstracted on the 8-, 11-, and 14-positions, generating 6-SDA-PhytoPs, 9-SDA-PhytoPs, 13-SDA-PhytoPs, and 16-SDA-PhytoPs respectively (Scheme 51). One study on GLA-derived phytoprostanes was reported by Porter et al. in 1975. In this study, it was shown that 13-F_1_-GLA-PhytoP is generated from LOX-generated hydroperoxide 9-HpODE in the presence of a free radical source in O_2_-saturated benzene.^353^ To the best of our knowledge, the formation of cyclic metabolites from SDA has not yet been reported.

#### 6.2.3 Phytofurans

**Scheme 52:**
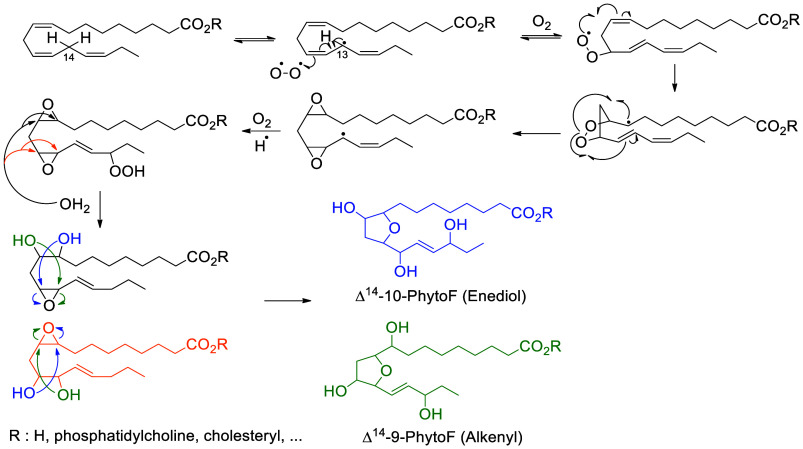
Biosynthesis of PhytoFs Proposed by Jahn et al.^102^ Color indicates the site of attack for a given chemical moiety and the resulting metabolite.

Under increased oxygen tension, such as in highly oxygenated tissues like lung, brain, and kidneys, cyclic nonenzymatic metabolites possessing a substituted tetrahydrofuran ring (PhytoFs) are preferentially formed compared to phytoprostanes.^373,374^ PhytoFs exist in two distinct families, termed alkenyl PhytoFs and enediol PhytoFs. In 2002, Fessel et al. were the first to propose two distinct mechanisms for the formation of the two families of PhytoFs.^373^ A unified route for the formation of both families was then proposed by Jahn et al. in 2008,^102^ based on the known formation of a *bis*-epoxide.^13^ The first step of phytofuran biosynthesis is identical to isoprostane biosynthesis and a cyclic endoperoxide is created by addition of a dioxygen molecule followed by a 5-*exo-trig* cyclization. After generation of the cyclic endoperoxide, a 1,3-S_H_i (intramolecular homolytic substitution) reaction, a 3-exo cyclization generates a radical diepoxide that traps a dioxygen molecule to form a diepoxyhydroperoxide after protonation. After hydrolysis, two regioisomeric epoxy diols are created. An intramolecular nucleophilic ring opening of the epoxide by a hydroxy group generates the phytofurans (Scheme 52). Analogously to PhytoPs, the biosynthesis mechanism of PhytoFs has not been determined directly but it has been inferred by comparison with isofurans (IsoFs).^350^

## 7 Physiological Distribution and Compartmentalization

As with their parent fatty acids, oxygenated octadecanoids are incorporated into various biomolecular complexes through esterification with the free alcohols of glycerol and cholesterol, and their cellular compartmentalization, physiological distribution, and trafficking behavior can be best understood in this context.^375−377^ Similarly, NO_2_-FAs are stable in both esterified and nonesterified forms, are transported in lipoprotein particles^378^ and accumulate in peripheral tissues.^379^ While it has long been recognized that complex lipids can contain functionalized lipid species, the tools for their efficient investigation are still in development, and the importance of the esterified fraction is just beginning to be broadly considered and addressed.^378,380,381^ The majority of oxylipin investigations still focus on the free acid; however, there is an increasing appreciation that the esterified fraction is often the larger oxylipin pool.^380^ While the biosynthesis of esterified oxylipins has been reviewed in plants,^382^ less is known about these pathways in mammals. Free oxylipins can be produced from free fatty acids and then subsequently be esterified into complex lipids. Alternatively, fatty acids can be esterified into complex lipids and then directly transformed into oxylipins.^383,384^ For example, it has been shown that both human 15-LOX-2 and mouse 8-LOX can transform phospholipid esterified AA to 15-HETE.^385^ It has long been known that cholesteryl linoleate esters of LDL are a substrate for 15-LOX, leading to formation of 13(*S*)-HODE by recombinant 15-LOX^46^ or rabbit 15-LOX.^386^ Accordingly, future investigations in the oxylipin field should further characterize the formation and function of esterified octadecanoids.

While the nonesterified octadecanoids are thought to be bioactive fractions, esterified forms can act as lipid mediator reservoirs and may play a role in the activity and function of lipid microdomains. For instance, at the cellular level, the Lands cycle describes the fundamental biochemical process of phospholipid membrane remodeling.^387^ Both epoxides and alcohols of AA participate in this process.^377,388,389^ Notably long-chain acyl-CoA synthetase (ACSL)4 and ASCL1 prefer AA at similar rates, but epoxy and hydroxy arachidonates differentially.^389^ By analogy, the octadecanoids would also be expected to be active participants in this biochemical network, however little direct evidence exists. Interestingly, ASCL1 is broadly distributed and has a distinct preference for oleate and linoleate,^390^ suggesting that it may be a candidate to regulate the intercellular phospholipid distribution of some octadecanoids. We have previously reported that adipose tissue harbors a significant pool of esterified eicosanoids and octadecanoids, whose composition and relative distribution between free and esterified pool are influenced by dietary fat content.^391^ The postprandial decline and late rebound of nonesterified HODEs, EpOMEs, DiHOMEs also suggest that this pool is substantially influenced by release from adipose triglycerides.^392,393^ Similar results were reported with insulin infusions in early and late fasting elephant seals, a unique animal model of insulin resistance.^394^ In unpublished works in the Syrian hamster (J.W. Newman, personal communication), octadecanoids were detected in cholesteryl esters, phospholipids and triglycerides of adipose, liver and muscle tissues. Notably, adipose and muscle tissue triglycerides were particularly enriched in HODEs, HOTrEs, EpOMEs, and EpODEs, and the relative balance of these LA and ALA metabolites were responsive to the dietary lipid balance. In supplemented adipocytes, the NODA, NOME, (*E,Z*)-NOMEs as well as their chain shortened dihydro- and tetranor- metabolites were also observed in mono-, di- and triacylglycerol pools as well as various phospholipid classes.^379^

Considering whole body octadecanoid trafficking, recognition of these bioactive agents as integral parts of lipoprotein particles raises a number of intriguing possibilities. For instance, receptor mediated delivery with lipase-mediated release of bioactive agents may represent an endocrine signaling avenue for such compounds.^376^ Within lipoprotein fractions, octadecanoid contributions have been reported to range from 75 to 90% of the totals, with HODEs, oxo-ODEs > EpOMEs ≫ DiHOMEs and vary by particle density class.^376,395^ Since upward of 90% of oxidized fatty acids in circulation are found in the esterified pools,^375,396^ these lipoprotein bioactive payloads will provide a steady pulsatile signal to the periphery, that will covary with that of other nutrients. Therefore, it will likely be necessary to fully speciate lipoprotein particle classes and subclasses to fully understand the passive and/or active dynamics associated with octadecanoid trafficking in the intact organism.

Another issue for consideration in investigating octadecanoids is the use of serum vs plasma. It has been extensively shown that the levels of oxylipins can vary with the blood preparation examined and that this must be taken into account during experimental preparation. This is primarily due to platelet activation during the blood collection process and is a known issue of concern with some eicosanoids (e.g., TxB_2_, 12-HETE).^397^ For many of the octadecanoids, there are relatively few differences in the choice of blood preparation protocol and there is not a major need to consider this issue (Table 3).

**Table 3:** Comparison of Octadecanoid Levels in Different Human Blood Preparations^a^

parent fatty acid	octadecanoid	serum^b^	serum^c^	serum^d^	plasma EDTA^b^	plasma EDTA^c^	plasma EDTA^d^	plasma heparin^b^	plasma citrate^b^
linoleic acid	9(10)-EpOME	2.7	0.4	0.8	1.6	0.3	0.8	1.8	1.7
	12(13)-EpOME	10	4	2.0	7.0	3	2.0	6.4	6.5
	9,10-DiHOME	14	5	2.9	11.9	4	3.0	10.4	10.7
	12,13-DiHOME	3.7	6	3.1	2.9	5	3.0	2.7	2.6
	9-HODE	15	14	7.0	9.3	12	7.1	9.9	8.4
	13-HODE	37	21	13.6	29.3	17	14.4	27.2	25.2
	9-KODE	2.6	2	2.3	1.9	<1	3.3	1.9	1.9
	13-KODE	0.8	<1	NR	0.6	2	NR	0.6	0.6
	10(*Z*)-9-oxo-12(13)-EpOME	4.0	NR^e^	1.1	2.3	NR	1.7	1.7	5.3
	9,10,13-TriHOME	2.3	NR	NR	1.3	NR	NR	1.2	3.0
	9,12,13-TriHOME	9.2	4	NR	8.3	5	NR	6.7	8.3

alpha linolenic acid	9(10)-EpODE	0.1	0.2	0.1	0.1	0.2	0.1	0.1	0.1
	12(13)-EpODE	0.2	0.2	NR	0.1	<4	NR	0.1	0.1
	15(16)-EpODE	15	3	2.0	10.1	2	2.0	10.8	8.9
	9,10-DiHODE	0.4	0.3	0.3	0.4	0.2	0.3	0.3	0.3
	12,13-DiHODE	0.5	0.3	NR	0.3	0.2	NR	0.4	0.4
	15,16-DiHODE	9	15	10.5	8.5	13	10.3	7.1	7.5
	9-HOTrE	2.3	0.5	0.6	1.6	0.5	0.5	1.6	1.3
	13-HOTrE	1.9	1	1.0	1.5	0.8	1.1	1.4	1.3

a Concentration in nM.

b Data are from a single male in which blood was collected and simultaneously prepared with the different anticoagulants. Oxylipins were measured with the analytical method published by Kolmert et al.^398^

c Data from Pedersen et al.^399^

d Data from Rajan et al.^400^

e NR = not reported. Compounds were not reported in the original publications.^399,400^

## 8 Preparation of Octadecanoids

In most cases, a given octadecanoid can be prepared in more than one way. The reactions and methods listed below have all been tested in our laboratory and can serve as useful starting points for investigators new to the field of octadecanoids and oxylipins. The compounds mentioned are in most cases derived from LA; however, as a rule, the reactions are also applicable to the other C18-PUFAs.

### 8.1 Hydroperoxides

**Scheme 53:**
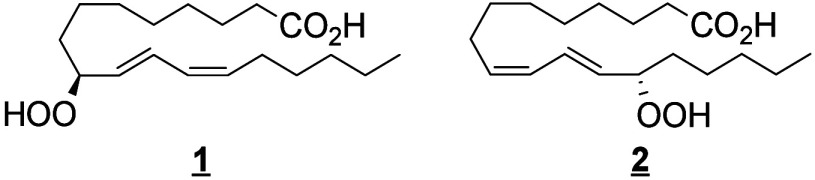
Structure of 9(S)-HpODE **1** and 13(S)-HpODE **2**

The 9(*S*)-hydroperoxide of 9(*S*)-HpODE **1** (Scheme 53) is conveniently prepared using the LOX enzyme present in tomato fruit.^401^ The sodium salt of the fatty acid is stirred at 23 °C under oxygen atmosphere with a crude homogenate of tomato fruit in 0.1 M potassium phosphate buffer pH 6.0.^401^ Purification is performed by silica gel chromatography followed by normal-phase HPLC. The amount of hydroperoxide can be determined gravimetrically or by UV spectroscopy using an ε value of 27 000 at the λ_max_ (around 235 nm). For discussion of the ε value of H(p)ODEs, see the review published by Gardner.^402^ For preparation of 13(*S*)-HpODE **2** (Scheme 53), commercially available soybean LOX-1 is the preferred enzyme. The fatty acid (e.g., LA, ALA) is stirred at 0 °C under oxygen atmosphere with the enzyme in 0.1 M sodium borate buffer pH 10.4; detailed conditions are provided in previous publications.^402,403^ Purification and quantitation are performed as outlined above.

E. J. Corey in an early paper noted that irrespective of the position oxygenated in the fatty acid chain, lipoxygenases produce hydroperoxides of the (*S*) absolute configuration.^404^ Since then, lipoxygenases producing (*R*)-hydroperoxides have been discovered, notably the 12(*R*)-lipoxygenase in human skin,^405^ and for preparation of (*R*)-hydroperoxides such enzymes can be utilized. Alternatively, enzymatic resolution of a racemic hydroperoxide solution can be used. As an example, 13(*R*)-HpODE was prepared by incubation of 13(*R*,*S*)-HpODE with corn allene oxide synthase, an enzyme that specifically converts the (*S*)-enantiomer into product, but does not react with the (*R*)-enantiomer.^11^

Racemic fatty acid hydroperoxides can be obtained by autoxidation or by nonradical oxygenation using singlet oxygen. For the preparation of 9(*R,S*)- and 13(*R,S*)-hydroperoxides derived from LA by the former method, LA is evaporated in a round-bottom flask to produce a film covering the wall. The flask is kept at 37 °C for 15 h under an atmosphere of oxygen gas.^406^ This will produce a mixture of (*E,Z*)-9(*R,S*)-, (*E,E*)-9(*R,S*)-, (*E,Z*)-13(*R,S*)-, and (*E,E*)-13(*R,S*)-HpODEs. If desired, formation of the (*E,E*)-isomers can be suppressed by adding α-tocopherol.^407^ Isolation of products can be performed by normal-phase HPLC, in which case the order of elution is (*E,Z*)-13(*R,S*)-HpODE (first), (*E,E*)-13(*R,S*)-HpODE, (*E,Z*)-9(*R,S*)-HpODE, and (*E,E*)-9(*R,S*)-HpODE. Racemic hydroperoxides of ALA, GLA and SDA can be prepared in the same general way.

**Scheme 54:**
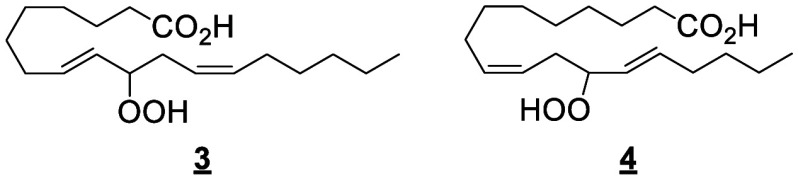
Structure of 10(*R*,*S*)-HpODE **3** and 12(*R*,*S*)-HpODE **4**

Photosensitized oxidation with generation of singlet oxygen provides a general method for producing racemic hydroperoxides. Illumination with visible light of oxygen-bubbled solutions containing methylene blue generates singlet oxygen, which produces two regioisomeric racemic hydroperoxides from each double bond in a PUFA. For example, irradiation (250 W lamp) of an oxygen-purged solution of LA in methanol containing 0.05% methylene blue at 5 °C produces a mixture of 9(*R,S*)- and 13(*R,S*)-HpODEs as well as 10(*R,S*)-HpODE **3** and 12(*R,S*)-HpODE **4** (Scheme 54).^408^

In the same way, photosensitized oxidation of ALA produces 6 regioisomeric hydroperoxides that can be separated by normal-phase HPLC.^409^

### 8.2 Hydroxides

**Scheme 55:**
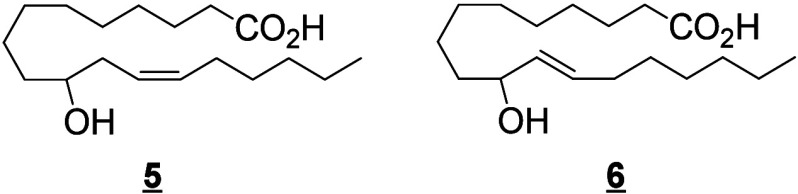
Structure of 12(*Z*)-10-HOME **5** and 11(*E*)-10-HOME **6**

NaBH_4_ in methanol (0 °C, 30 min) is commonly used to prepare a fatty acid hydroxide from its corresponding hydroperoxide.^410^ Milder reduction methods include SnCl_2_ in ethanol and triphenylphosphine in diethyl ether or methanol.^203,402^ The two first-mentioned methods require extractive isolation of the product, whereas the last method requires elimination of the triphenylphosphine oxide formed using e.g., a silica gel column. The fatty acid hydroxides can be separated into regioisomers and (*E,Z*)- and (*E,E*)-isomers using normal-phase HPLC. Various bacteria, notably *Lactobacilli*, produce hydroxy acids by a nonlipoxygenase route involving hydratase, dehydrogenase and isomerase enzymes.^223^ Examples of such compounds are 12(*Z*)-10-HOME **5** and 11(*E*)-10-HOME **6** formed from LA (Scheme 55). The corresponding compounds are produced from ALA. Such octadecanoids can be prepared using *Lactobacillus plantarum* enzymes^223^ or can be synthesized by chemical methods.^411^

The complete series of saturated hydroxystearates has been synthesized as described in the classical paper by Bergström et al.^412^ Most of these compounds were obtained by reduction of the corresponding oxo-stearate (see below).

### 8.3 Ketones

**Scheme 56:**
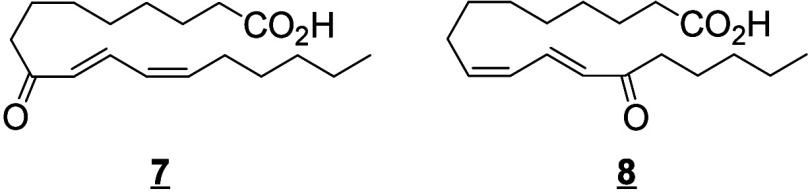
Structure of 9-oxo-ODE **7** and 13-oxo-ODE **8**

Fatty acid ketones (such as 9-oxo-ODE **7** and 13-oxo-ODE **8**, Scheme 56) can be prepared from the corresponding hydroxides using the Dess–Martin periodinane in dichloromethane at 0 °C.^413^ Alternatively, fatty acid hydroperoxides can be directly dehydrated into the corresponding ketones by treatment with acetic anhydride–pyridine (v/v 1:1) at 0 °C.^414^ Amounts of products can be determined gravimetrically or by UV spectroscopy using ε = 24 000 at the λ_max_ (around 278 nm).

**Scheme 57:**
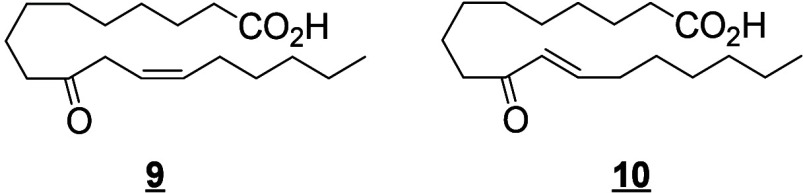
Structure of 12(*Z*)-10-oxo-OME **9** and 11(*E*)-10-oxo-OME **10**

Microbially generated fatty acid ketones include 12(*Z*)-10-oxo-OME **9** and 11(*E*)-10-oxo-OME **10** (Scheme 57) derived from LA as well as the corresponding compounds formed from ALA. *Lactobacillus plantarum* enzymes can be used to prepare these compounds^223^ or they can be synthesized using chemical methods.^411^ In the latter case, the conjugated enone **10** is prepared by treatment of **9** with perchloric acid in tetrahydrofuran, and its reduction with NaBH_4_ affords the hydroxy derivative **6**.

The complete series of 2- to 17-oxo-stearates has been synthesized.^412^ Coupling of dialkyl-cadmium derivatives to acid chlorides was employed in many of these syntheses and in other cases β-ketoesters were reacted with ω-haloesters.

### 8.4 Epoxides

**Scheme 58:**
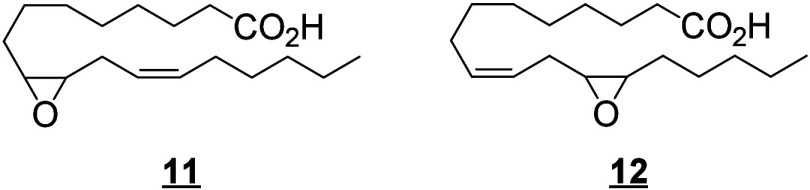
Structure of 9(10)-EpOME **11** and 12(13)-EpOME **12**

The monoepoxides of LA and other fatty acids can be obtained either by nonselective epoxidation of double bonds^415,416^ or be isolated as natural products, in the latter case as defined stereoisomers. As an example of the first-mentioned approach, methyl linoleate in chloroform is treated with 1.1 equiv of peracetic acid and the solution stirred at 23 °C for 2–3 h.^415^ In order to optimize the yield of the two monoepoxides (Scheme 58), the reaction progress has to be followed by TLC or GC-MS. Alternatively, *m*-CPBA is also a broadly used reagent to perform the epoxidation of LA and ALA in a nonselective way.^417−419^ Unreacted methyl linoleate and diepoxide can be removed by silica gel chromatography and the monoepoxide fraction subjected to normal-phase HPLC for separation of the (±)-*cis*-9(10)-epoxy-12(*Z*)- and (±)-*cis*-12(13)-epoxy-9(*Z*)-octadecenoates (9(10)-EpOME **11** and 12(13)-EpOME **12**, respectively, of which the latter is the first eluting). Saponification to the free epoxy acids can be performed by treatment with 0.2 M NaOH in 80% methanol at 23 °C for 15 h. The enantiomers of 9(10)-EpOME **11** are resolved on a Chiralpak AD column in the order 9(*S*),10(*R*)-isomer eluting before 9(*R*),10(*S*)-isomer.^420^

**Scheme 59:**
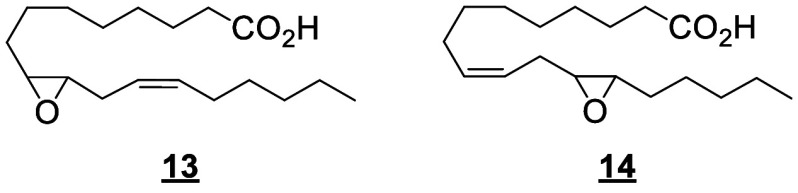
Structure of 9(10)-*trans*-EpOME **13** and 12(13)-*trans*-EpOME **14**

**Scheme 60:**
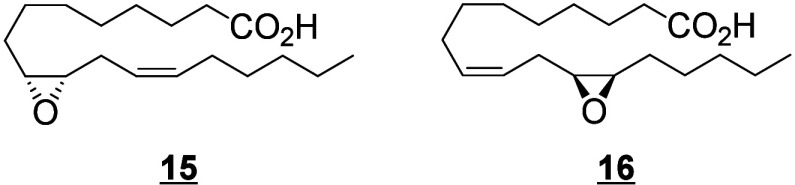
Structure of (+)-Coronaric Acid **15** and (+)-Vernolic Acid **16**

By applying the methodology outlined above on the linoleate *trans*-isomers methyl 9(*E*),12(*Z*)- and 9(*Z*),12(*E*)-octadecadienoates, it is possible to prepare the corresponding *trans*-epoxides, i.e., (±)-*trans*-9(10)-epoxy-12(*Z*)- and (±)-*trans*-12(13)-epoxy-9(*Z*)-octadecenoates, (9(10)-*trans*-EpOME **13**, and 12(13)-*trans*-EpOME **14**, respectively (Scheme 59)).

(+)-Coronaric acid (9(*R*)(10(*S*))-EpOME **15**(421)), (+)-vernolic acid (12(*S*)(13(*R*))-EpOME **16**(422)), and (−)-vernolic acid (12(*R*)(13(*S*))-EpOME^423^) are examples of epoxy acids readily available by extraction of certain seeds, e.g., those from *Chrysanthemum coronarium*, *Euphorbia lagascae*, and *Malope trifida*, respectively (Scheme 60).

### 8.5 Diols

**Scheme 61:**
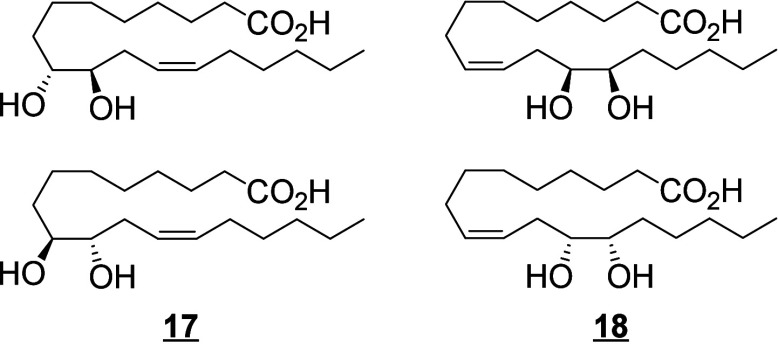
Structure of *threo*- (**17**) and *erythro*- (**18**) 9,10-DiHOME and 12,13-DiHOME

Ring opening of the above-mentioned epoxides by refluxing with acetic acid followed by saponification or methanolysis affords the corresponding diols.^422^ Perchloric acid in tetrahydrofuran is often used to effect opening of epoxides into diols; however, in the case of the fatty acid epoxides **11**–**16** such treatment results in the formation of byproducts due to the presence of the homoallylic double bond. Racemic diols having the *threo* relative configuration are formed from the *cis*-epoxides, e.g., **17** from **11**, whereas *trans*-epoxides produce *erythro* diols, e.g., **18** from **14** (Scheme 61).

Interestingly, attack by acetic acid takes place with preference at the epoxide carbon, which is most distal to the double bond (C-9 in the 9(10)-epoxides and C-13 in the 12(13)-epoxides). This means that diols enriched (about 65:35) with one enantiomer can be prepared if enantiopure epoxides are used.^424^ Another way of producing *erythro* diols is dihydroxylation of double bond(s) in an unsaturated fatty acid using, e.g., the so-called Upjohn method.^425^

### 8.6 Hydroxy-epoxides

**Scheme 62:**
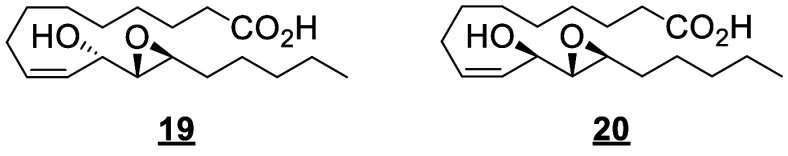
Structure of the Free Acids of 9(*Z*)-11(*S*)-OH-12(*S*)(13(*S*))-EpOME **19** and 9(*Z*)-11(*R*)-OH-12(*S*)(13(*S*))-EpOME **20**

Fatty acid hydroperoxides can produce a variety of hydroxy-epoxide and keto-epoxides, chemically as well as enzymatically. For example, the 2,3-hydroxy-epoxides in which the epoxide and hydroxy groups are vicinally located and additionally possess a double bond vicinal to either the hydroxy group or the epoxide (*cf. e.g.*, structures **19** and **21**). As an example of the preparation of a hydroxy-epoxide of the former type, the methyl ester of 13(*S*)-HpODE is treated in dichloromethane at −78 °C with trifluoroacetic anhydride and lutidine for 30 min followed by K_2_CO_3_ in methanol.^426^ This produces the methyl esters of 9(*Z*)-11(*S*)-OH-12(*S*)(13(*S*))-EpOME and 9(*Z*)-11(*R*)-OH-12(*S*)(13(*S*))-EpOME (**19** and **20**, respectively, Scheme 62) of which the first-mentioned (*erythro*) isomer is the predominant one. These isomers can be easily separated by normal-phase HPLC, the *erythro* form being the first eluting, and then saponified by mild alkali treatment.

**Scheme 63:**
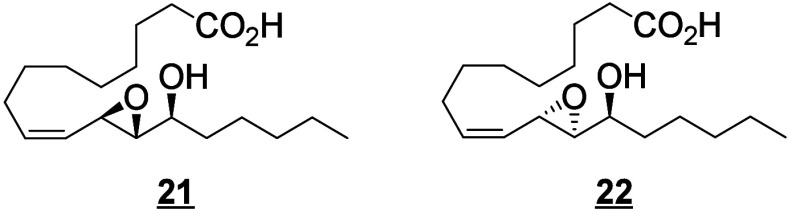
Structure of the Free Acids of 9(*Z*)-13(*S*)-OH-11(*R*)(12(*R*))-EpOME **21** and 9(*Z*)-13(*S*)-OH-11(*S*)(12(*S*))-EpOME **22**

2,3-Hydroxy-epoxides of the latter type can also be prepared from fatty acid hydroperoxide methyl esters, in this case by treatment with a hexane solution saturated with vanadium oxyacetylacetonate.^427^ As an example, from the methyl ester of 13(*S*)-HpODE is produced a 1:1 mixture of the methyl ester of 9(*Z*)-13(*S*)-OH-11(*R*)(12(*R)*)-EpOME and 9(*Z*)-13(*S*)-OH-11(*S*)(12(*S*))-EpOME (**21** and **22**; *threo* and *erythro*, respectively, Scheme 63). Separation of these isomers can easily be performed by normal-phase HPLC, and again the *erythro* form elutes first. The allylic epoxide present in these hydroxy-epoxides renders them quite sensitive to acid (reported half-life times at pH 3, 1–2 min^428^), and special care has to be taken during the workup following their saponification.

**Scheme 64:**
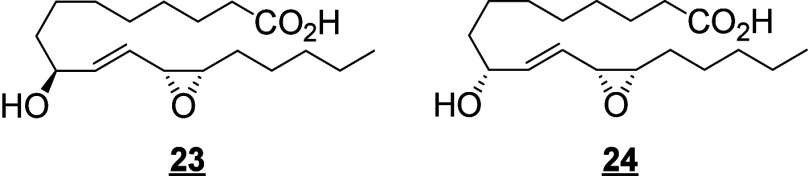
Structure of 10(*E*)-9(*S*)-OH-12(*R*)(13(*S*))-EpOME **23** and 10(*E*)-9(*R*)-OH-12(*R*)(13(*S*))-EpOME **24**

In addition to the chemical methods mentioned above, enzymes such as peroxygenase and other epoxy alcohol synthases/hydroperoxide isomerases can be used to generate hydroxy-epoxides from hydroperoxides. In this case hydroxy-epoxides having the double bond located between the epoxide and hydroxy functionalities are the exclusive or predominant products. For example, upon incubation of 9(*S*)-HpODE with preparations from beetroot, the hydroxy-epoxide 10(*E*)-9(*S*)-OH-12(*R*)(13(*S*))-EpOME **23** is formed,^429^ whereas 9(*R*)-HpODE generates 10(*E*)-9(*R*)-OH-12(*R*)(13(*S*))-EpOME **24** (Scheme 64).^430^

### 8.7 Trihydroxy Acids

**Scheme 65:**
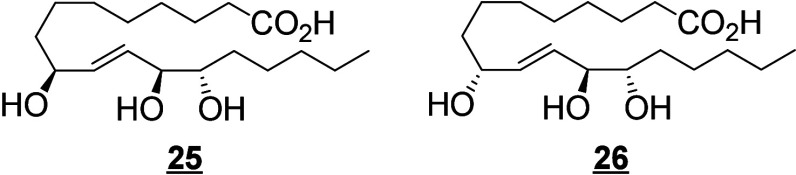
Structure of 9(*S*),12(*S*),13(*S*)-TriHOME **25** and 9(*R*),12(*S*),13(*S*)-TriHOME **26**

Acid-catalyzed solvolysis of allylic epoxy alcohols of types **21** and **22** mainly takes place by solvent attack at the double bond carbon most remote from the epoxide followed by double bond isomerization and epoxide opening.^427,431^ When carried out in aqueous medium, a given hydroxy-epoxide produces a trihydroxy acid formed as a 1:1 mixture of two diastereoisomers. For example, acid treatment of hydroxy-epoxide **21** in water produces the pair 9(*S*),12(*S*),13(*S*)-TriHOME (pinellic acid, **25**) and 9(*R*),12(*S*),13(*S*)-TriHOME **26** (Scheme 65).

**Scheme 66:**
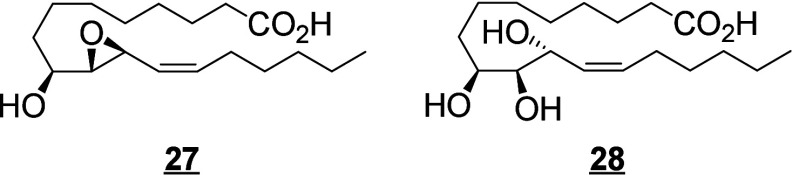
Structure of 12(*Z*)-9(*S*)-OH-10(*S*)(11(*S*))-EpOME **27** and 9(*S*),10(*S*),11(*R*)-TriHOME **28**

Normal-phase HPLC can be used for preparative separation of the methyl esters of trihydroxy acid regio- and diastereoisomers, and the 16 possible 9,10,13- and 9,12,13-trihydroxyoctadecenoic acids derived from LA have been resolved by chiral-phase HPLC.^432^ Stereospecific opening of allylic hydroxy-epoxides catalyzed by epoxide hydrolases is an alternative to the acid-catalyzed reaction shown above. Thus, potato leaves contain an epoxide hydrolase activity which catalyzes stereospecific hydrolysis of the 2,3-hydroxy-epoxide 12(*Z*)-9(*S*)-OH-10(*S*)(11(*S*))-EpOME **27** into trihydroxy acid 9(*S*),10(*S*),11(*R*)-TriHOME **28**(188) (Scheme 66), and a partially purified epoxide hydrolase from oat seeds^433^ catalyzes stereospecific hydrolysis of **23** into trihydroxy acid **25** (pinellic acid).

Additional trihydroxy acids of this and related types have been prepared using chemo-enzymatic methods,^430,434^ and by total chemical synthesis.^435^

### 8.8 Nitro

In 2013, Woodcock et al. reviewed nitro fatty acid synthesis procedures, which were classified into three approaches: (1) nitrogen dioxide radical/nitronium ion reactions, (2) nitroselenation/nitromercuriation reactions, and (3) total syntheses based on Henry nitroaldol reactions for isomeric synthesis. The review also included a general procedure for nitroselenation of OA and total synthesis of 9(*E*)-9-NOME as well as purification and analysis of nitrated fatty acids.^436^

**Scheme 67:**
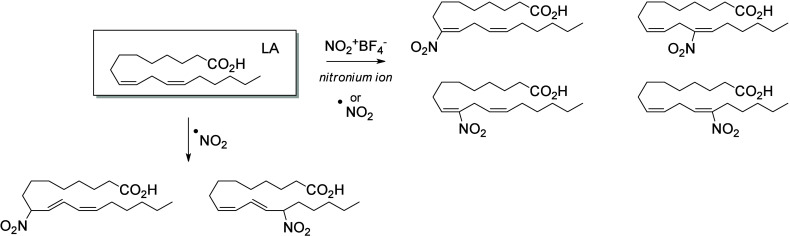
Synthesis of Nitrated Products of Linoleic Acid (LA) by Nitrogen Dioxide Radical and Nitronium Ion

*(1) Nitrogen Dioxide Radical/Nitronium Ion Reactions.* This method, which mimics the biosynthesis of nitro-FAs, is better adapted for the production of endogenous metabolites from a biological matrix in order to study the formation of nitro-derivatives under pathophysiological conditions. It is based on the exposure of rats to nitrogen dioxide (NO_2_) in order to generate *in situ* nitrogen dioxide radical (^•^NO_2_).^437−439^ This approach gives a mixture of products due to the high reactivity and lack of selectivity of the nitrogen dioxide (Scheme 67). Nitronium ions (i.e., NO_2_BF_4_) can also be used to generate a mixture of nitro-alkene products.^440^

**Scheme 68:**
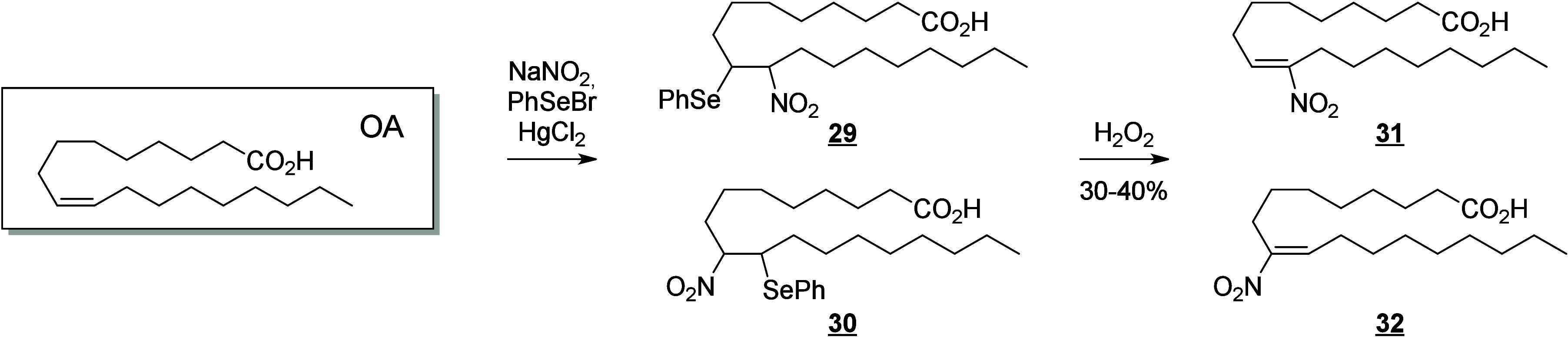
Synthesis of 9(*E*)-9-NOME **32** and 9(*E*)-10-NOME **31** by Nitroselenation of Oleic Acid (OA)

*(2) Nitroselenation/Nitromercuriation Reactions.* Nitro-alkenes can be obtained by nitroselenation of unsaturated FAs via a two-step method. For example, the nitro-selenyl intermediates **29** and **30** are created by treatment of OA by mercury chloride, phenylselenyl bromide and sodium nitrite at 0 °C. Then, a treatment of the crude reaction by H_2_O_2_ at 0 °C gives a mixture of 9(*E*)-9-NOME **32** and 9(*E*)-10-NOME **31**, that can be separated by HPLC (Scheme 68).^345,436^

**Scheme 69:**
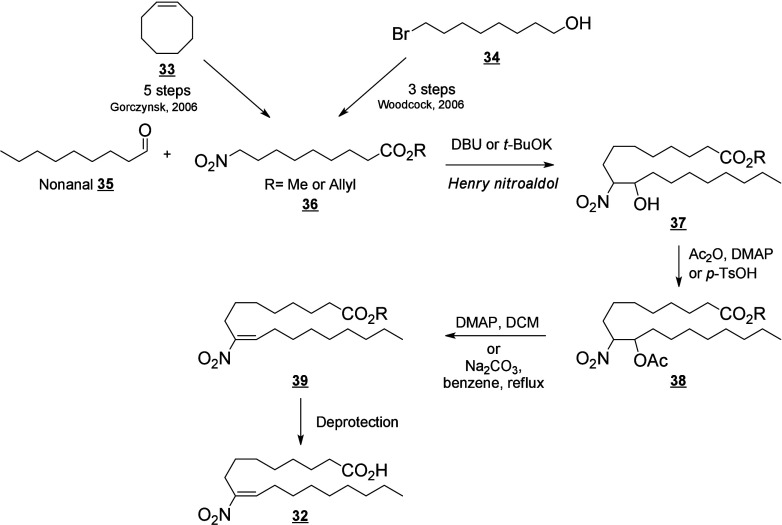
Example of Two Total Syntheses of 9(*E*)-9-NOME **32**

*(3) Total Syntheses Based upon Henry Nitroaldol Reactions for Isomeric Synthesis.* The utility of the total synthesis of nitro-alkenes is the ability to obtain a specific isomer. The synthesis of nitrated metabolites of OA^441,442^ and LA^443,444^ has been published and all methods use a Henry nitroaldol reaction. Typically, the free acid is protected as a methyl^441,443,444^ or allyl^442^ ester, and a Henry nitroaldol reaction is performed using either DBU^442^ or *t*-BuOK^441,443^ as a base. The generated nitro-hydroxy intermediate is acetylated and the acetoxy group is eliminated using DMAP^441,443^ or Na_2_CO_3_^442^ to yield the nitro-alkene (Scheme 69).

**Scheme 70:**
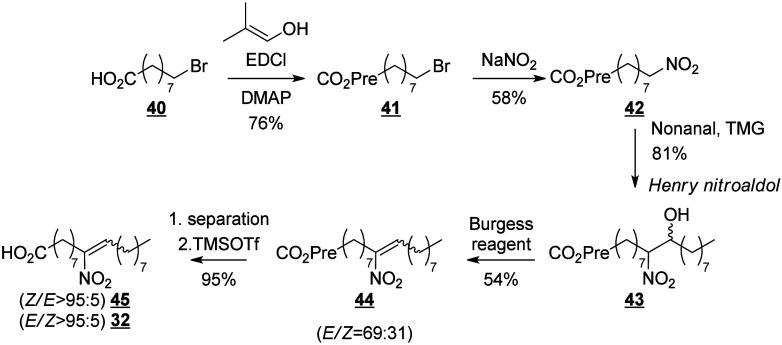
Total Synthesis of 9(*E*)-9-NOME **32** and 9(*Z*)-9-NOME **45**

Recently, an elegant one-pot synthesis has been published by Hassan et al.^445^ In this work, they first describe a synthesis that enables obtaining either (*E*)- or (*Z*)-nitroalkene with good stereoselectivity (>95:5 for both stereoisomers). This is the first multistep synthesis of a (*Z*)-nitro-FA reported. First, the free acid of bromoheptanoic acid **40** is protected by a prenyl group, then prenyl nitroheptanoate **42** is generated and the Henry nitroaldol step was performed with 1,1,3,3 tetramethylguanidine (TMG) and nonanal. The generated hydroxy group of **43** was eliminated with Burgess reagent, a mild dehydrating reagent, and both 9(*E*)-9-NOME **32** and 9(*Z*)-9-NOME **45** were obtained with good selectivity (>95:5) following separation of the isomers and deprotection (Scheme 70).

**Scheme 71:**
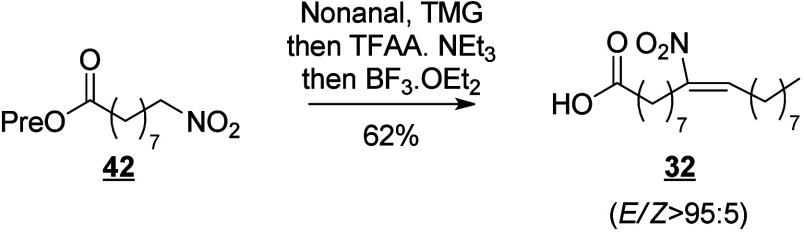
One-Pot Synthesis of 9(*E*)-9-NOME **32**

The (*E*)-isomer can be obtained selectively by a one-pot synthesis. First, the Henry nitroaldol reaction between nonanal and prenyl heptanoate **42** was performed with TMG, then trifluoroacetic anhydride (TFAA) and triethylamine were added *in situ* to eliminate the hydroxy group, and finally boron trifluoride diethyl etherate was added to the mixture to deprotect the acid and obtain 9(*E*)-9-NOME **32** with an efficient one-pot procedure (Scheme 71).

### 8.9 Furan Fatty Acids

**Scheme 72:**
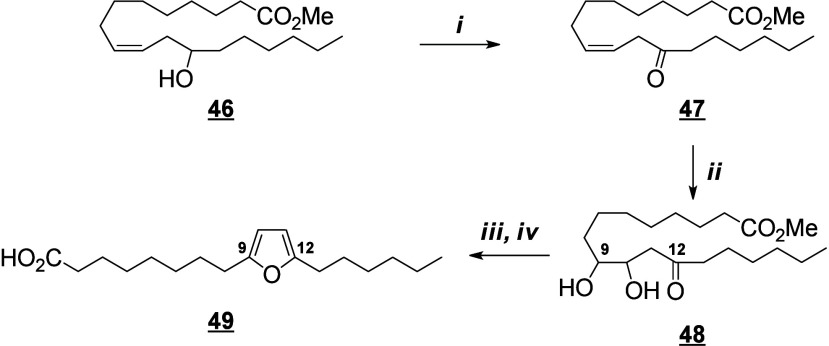
Synthetic Pathway of 9(12)-Epoxyoctadecadienoic Acid **49**

Octadecanoids incorporating a furan ring exist as ring-methylated as well as nonmethylated compounds and several methods have been described for their preparation by total organic synthesis.^446−448^ For easy preparation of the nonmethylated furans 9(12)- and 10(13)-epoxyoctadecadienoic acids, it is possible to use naturally occurring starting materials as exemplified with preparation of the first-mentioned compound **49** from the methyl ester of ricinoleic acid **46**. Oxidation with the Dess–Martin periodinane (i) and dihydroxylation (ii) is followed by warming (iii; 80 °C for 30 min) and saponification (iv). Reaction (iii) involved spontaneous attack by the C-9 hydroxy group at the C-12 carbonyl forming an unstable cyclic hemiketal that loses 2 molecules of H_2_O (Scheme 72).^449^

**Scheme 73:**
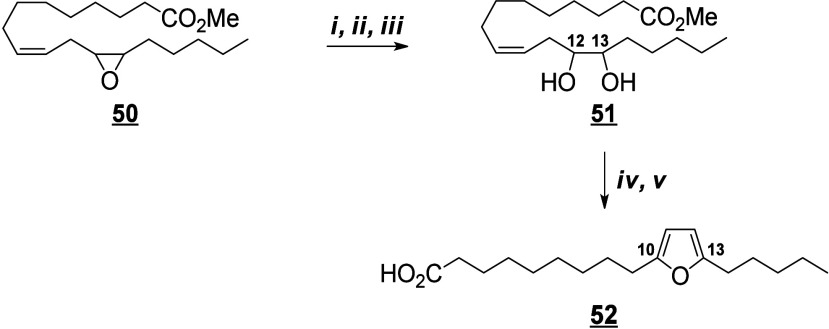
Synthetic Pathway to Form 10(13)-Epoxyoctadecadienoic Acid **52**

The related 10(13)-epoxy-10,12-octadecadienoic acid **52** can be prepared starting from (±)-vernolic acid **50**. Acetolysis followed by saponification and methylation (i, ii, iii) affords the *threo*-12,13-diol **51**, which undergoes aerobic cyclodehydrogenation in the presence of PdCl_2_ and CuCl_2_ (iv; 95 °C for 20 min) to produce the desired product **52** in good yield following saponification (v).^361^ Purifications are performed by RP-HPLC (Scheme 73).

### 8.10 Isoprostanes

#### 8.10.1 F Series

**Scheme 74:**
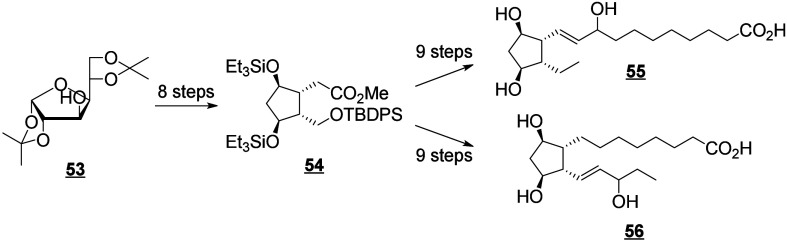
Synthetic Pathway of 9-F_1_t-PhytoP **55** and 16-F_1_t-PhytoP **56** from Diacetone d-Glucose **53** by the Durand Group

F-PhytoPs have been synthesized by Durand and colleagues, and all isomers possessing a *syn–anti–syn* configuration have been obtained, for the two coexisting series (9th and 16th series).^450,451^ The *syn*-*anti*-*syn* ester **54** was first prepared in 8 steps from diacetone l-glucose **53**.^452,453^ The chains of the phytoprostanes were subsequently introduced by Wittig and Horner–Wadsworth–Emmons (HWE) reactions to obtain 9-F_1t_-PhytoP **55** and 16-F_1t_-PhytoP **56** in 9 steps from the ester **54**(Scheme 74).^450^ The enantiomers *ent*-9-F_1t_-PhytoP and *ent*-16-F_1t_-PhytoP were also obtained following the same strategy starting from diacetone d-glucose.^451^

Recently, the first synthesis of a prostaglandin version of a phytoprostane has also been performed by Jahn and colleagues, who developed a strategy based on a radical anion oxidative cyclization and copper(I)-mediated alkyl–alkyl coupling.^454^

#### 8.10.2 D/E Series

**Scheme 75:**
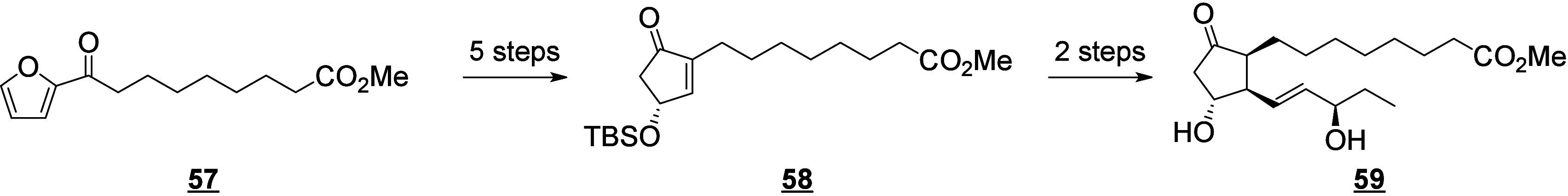
Synthetic Pathway of 16-*epi*-16-*E*_1t_-PhytoP **59** by Spur and Colleagues

**Scheme 76:**
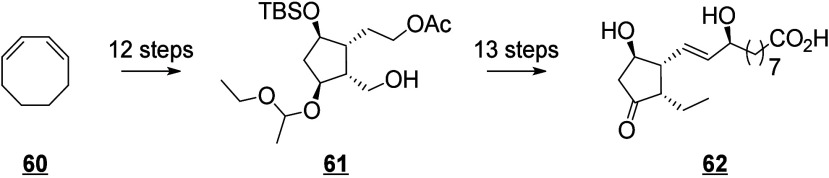
Synthetic Pathway of *ent*-9-D_1t_-PhytoP **62** by Durand and Colleagues

The 16-*epi*-16-E_1t_-PhytoP **59** and *ent*-9-D_1t_-PhytoP **62** have been synthesized in the laboratories of Durand^102^ and Spur.^455^ The synthesis of 16-*epi*-16-E_1t_-PhytoP **59** was initiated with preparation of the furan precursor **57** by Friedel–Crafts acylation of furan using the anhydride of the azelaic acid monoester.^455^ A ZnCl_2_-induced rearrangement followed by treatment with chloral, enzymatic resolution and TBS protection yielded the key intermediate **58**, on which the second chain of 16-*epi*-16-E_1t_-PhytoP **59** was introduced by a conjugated addition (Scheme 75). The *ent*-9-D_1t_-PhytoP was prepared in 2009 by Durand and colleagues following a procedure developed for the synthesis of 15-D_2t_-IsoP and 15-*epi*-15-E_2t_-IsoP.^456^ The *ent*-9-D_1t_-PhyotP **62** was prepared in 25 steps from 1,3-cyclooctadiene^457^ via the functionalized intermediate **61**, which was nicely obtained through a regioselective enzymatic acetylation of the corresponding diol^102,458^ (T. Durand, personal communication) (Scheme 76).

#### 8.10.3 J/A Series

**Scheme 77:**
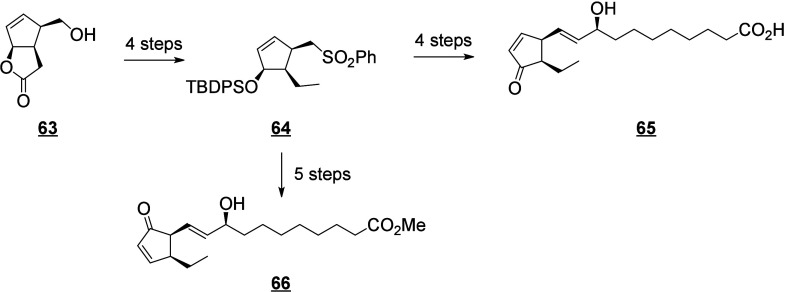
Synthetic Pathway for Formation of 9-J_1_-PhytoP **65** and 9-A_1_-PhytoP **66** by Zanoni and Vidari^459^

9-J_1_-PhytoP **65** and 9-A_1_-PhytoP **66** were prepared from the common sulfone intermediate **64** by the group of Vidari and Zanoni.^459^ This common intermediate was obtained in 4 steps from the enantiopure hydroxymethyl γ-lactone **63**. The preparation of the enantiopure starting material was previously described by the same group.^460,461^ From this sulfone, both 9-J_1_-PhytoP **65** and 9-A_1_-PhytoP **66** were obtained in 4 and 5 steps, respectively (Scheme 77). It is interesting to note that 9-A_1_-PhytoP **66** was obtained as a methyl ester instead of a free acid, which is rather unstable due to the lability of the hydrogen on the 12th position. This is the only description of the synthesis of A- and J-PhytoP to date.

#### 8.10.4 B/L Series

The four existing B- and L-PhytoPs have been synthesized by several groups. 9-L_1_-PhytoPs **69** and 16-B_1_-PhytoP **72** and their enantiomers *ent***-69** and *ent***-72** were first prepared by Boland and colleagues.^462^ A few years later, these phytoprostanes were also made by several research groups including Mikołajczyk,^463,464^ Riera,^465,466^ Durand,^467^ and Vidari.^468^ Finally, the synthesis of 16-L_1_-PhytoP **79** was described by Vidari and colleagues in 2015.^468^

**Scheme 78:**
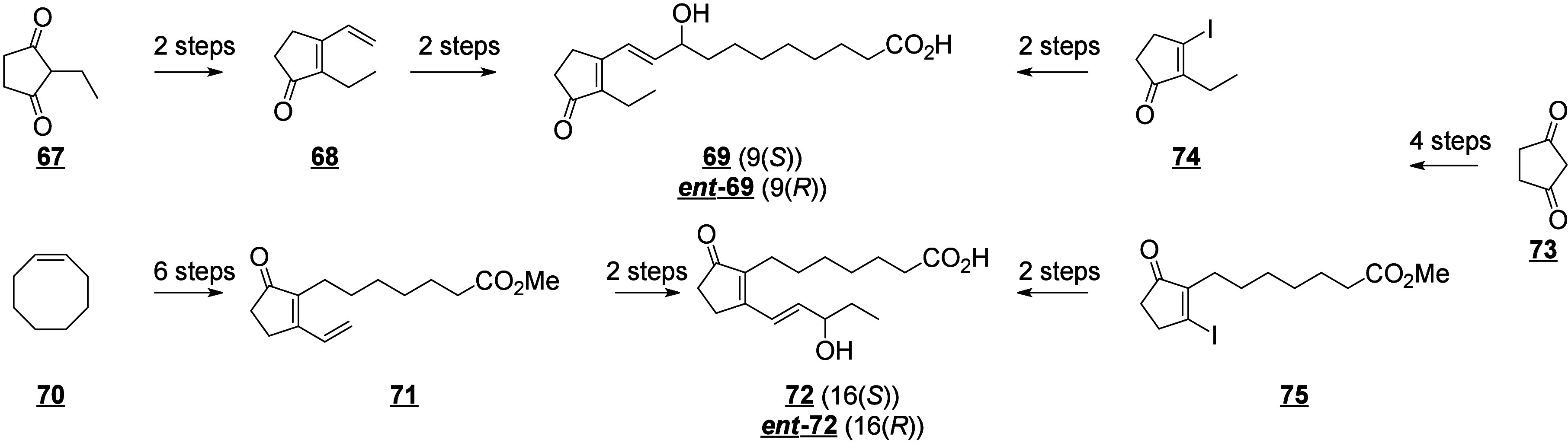
Synthetic Pathways for Formation of 9-L_1_-PhytoP **69** and 16-B_1_-PhytoP **72**, and Their Enantiomers *ent***-69** and *ent***-72** by Boland and Colleagues^462^

9-L_1_-PhytoP **69**, 16-B_1_-PhytoP **72** and their enantiomers *ent***-69** and *ent***-72** were easily obtained from the conjugated dienones **68** and **71**,^467^ or from the vinyl iodides **74** and **75**.^462^ These required intermediates were prepared from the commercially available 1,3-cyclopentandione **67**(467,462) or from the cyclooctene **70**.^467^ The second chains of the phytoprostanes were then introduced either by a metathesis reaction on the conjugated dienones **68** and **71**(467) or by a Heck-type alkylation of the vinylic iodides **74** and **75**(462) (Scheme 78).

**Scheme 79:**
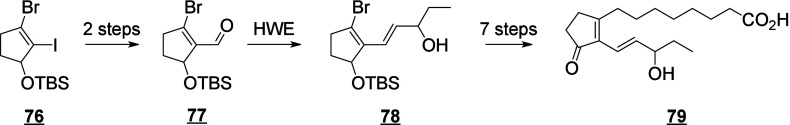
Synthetic Pathway for Formation of 16-L_1_-PhytoP **79** by Zanoni and Vidari^468^

16-L_1_-PhytoP **79** was prepared in 10 steps from O-TBS-protected 2-iodo-3-bromocyclopentenol **76** by the group of Zanoni and Vidari. A I/Li exchange on intermediate **76** followed by formylation with dimethylformamide formed the aldehyde **77**. Then, the first chain of the phytoprostane was introduced by a HWE reaction using diethyl (2-oxobutyl)phosphonate to yield the intermediate **78**. Finally, 7 extra steps were necessary to obtain the 16-L_1_-PhytoP **79** from intermediate **78**(Scheme 79).^468^

### 8.11 Phytofurans

**Scheme 80:**
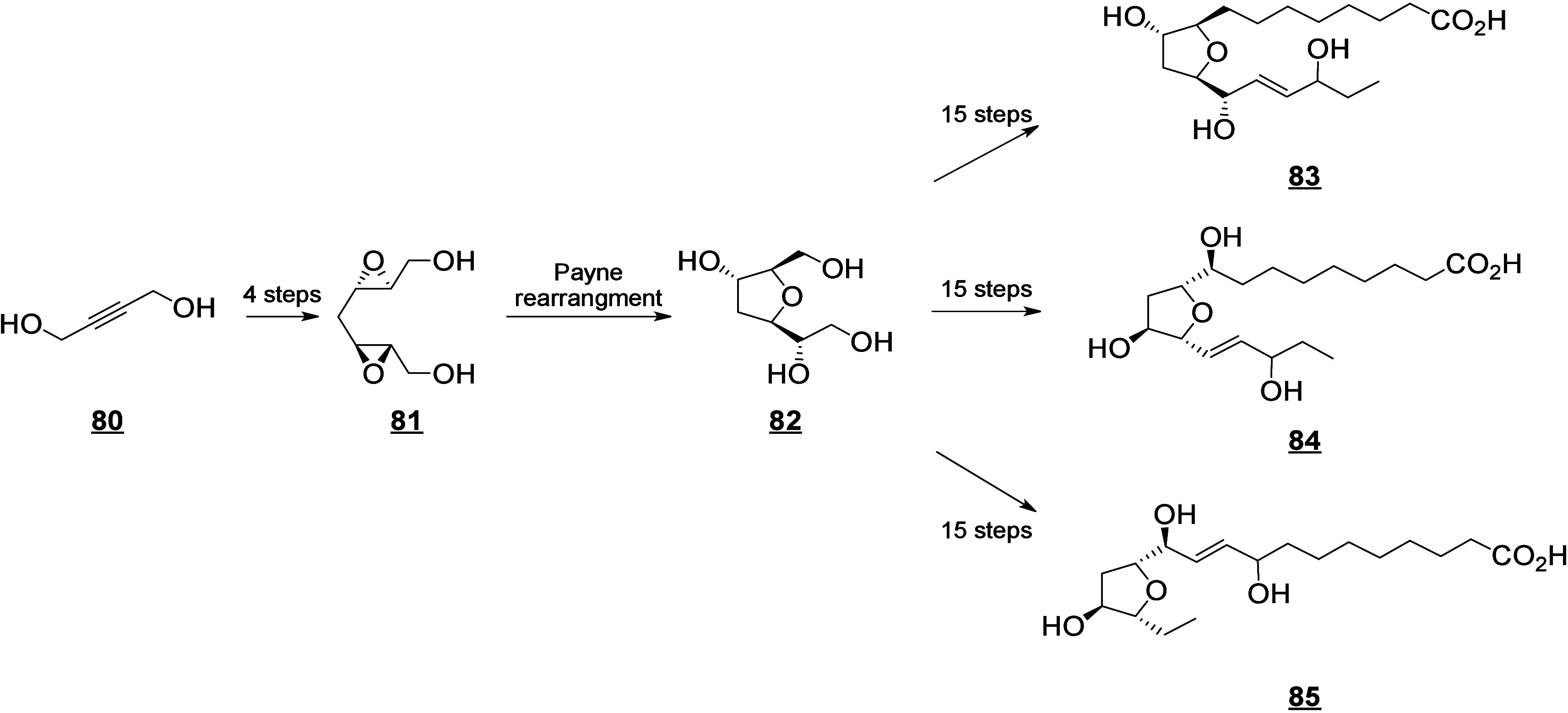
Synthetic Pathway of *ent*-16(*R*,*S*)-13-epi-ST-Δ^14^-9-PhytoF **83**,^469^*ent*-16(*R*,*S*)-9-epi-ST-Δ^14^-10-PhytoF **84**,^470^ and *ent*-9(*R*,*S*)-12-epi-ST-Δ^10^-13-PhytoF **85**(470) by Durand and Colleagues

Only one strategy for the synthesis of phytofuran has been reported in the literature. Durand and colleagues described the synthesis of *ent*-16(*R*,*S*)-13-*epi*-ST-Δ^14^-9-PhytoF **83** in 2015.^469^ Following the same strategy, *ent*-16(*R*,*S*)-9-*epi*-ST-Δ^14^-10-PhytoF **84** and *ent*-9(*R*,*S*)-12-*epi*-ST-Δ^10^-13-PhytoF **85** were also prepared by the same group in 2017.^470^ The *ent*-16(*R*,*S*)-13-*epi*-ST-Δ^14^-9-PhytoF **83**, *ent*-16(*R*,*S*)-9-*epi*-ST-Δ^14^-10-PhytoF **84** and *ent*-9(*R*,*S*)-12-*epi*-ST-Δ^10^-13-PhytoF **85** were made in 20 steps from the commercially available but-2-yne-1,4-diol **80**, *via* a Payne rearrangement of the *bis*-epoxide intermediate **81**, which yielded the tetraol THF intermediate **82**(Scheme 80).

## 9 Measurement of Octadecanoids

A vital component in the study of octadecanoids is the ability to reliably measure these metabolites, either singly for the confirmation of synthesis and purification, or as metabolite suites for biological investigations. There are several decades of literature on the quantification of oxylipins, and an in-depth discussion is beyond the scope of this review. The vast majority of published methods focus on the eicosanoids given their long history of study. For applications in biomedicine, there are few methods designed to specifically target the octadecanoids. Instead, selected octadecanoids have been included in eicosanoid and oxylipin metabolic profiling methods. Accordingly, these general oxylipin methods will be briefly summarized in this section. A consistent challenge experienced in the development of methods for the measurement of oxylipins has been the pM to nM range of endogenous levels. Early efforts employed a range of methods to measure AA metabolites, including radioimmunoassay (RIA), enzyme linked immunoassay (ELISA) and spectrophotometry.^471^ RIA and ELISA methods are sensitive (detection limits of low aM to pM, respectively) and relatively simple to implement; however, they lack specificity and are artifact-prone. Commercial ELISA kits are available for the more common octadecanoids from linoleic acid (e.g., 9-HODE, 13-HODE, EpOMEs, DiHOMEs); however, they are generally not available for other species and there is a paucity of radiolabeled octadecanoids. Accordingly, there are limitations in the application of RIA and ELISA methods for the study of octadecanoids.

UV chromatography has been used extensively for investigations of the eicosanoids and docosanoids.^472−474^ However, it is less useful for many of the octadecanoids, which do not exhibit a selective absorption spectrum due to a lack of conjugated double bonds. The octadecenoids containing a *cis*–*trans* conjugated diene (i.e., HODEs and HOTrEs) possess a λ_max_ = 235–237 nm and a molar extinction coefficient (ε) of 27000 M^–1^ cm^–1^ and the corresponding keto metabolites absorb equally strongly around 275–285 nm (λ_max_ being solvent dependent for oxo-ODEs). Metabolites possessing one or more nonconjugated alkene moieties (e.g., HOMEs) exhibit only “end absorbance” with a weak spectrum near 200 nm and lacking in any diagnostic utility. For products with a characteristic chromophore, UV spectrometry is useful for quantitation of micromolar concentrations of authentic standards (5–100 μM). UV with diode array detection on HPLC is capable of producing high quality UV spectra on low nanomole levels of product and thus has a role in product identification. Beyond these applications, UV-based analyses are of limited utility for measurements or assay of the octadecanoids.

The coupling of a gas chromatograph (GC) to a mass spectrometer was first accomplished by Ragnar Ryhage at the Karolinska Institute in 1962, setting the stage for much of the structural elucidation work for the discoveries of eicosanoids that led to awarding of the Nobel Prize in 1982.^475^ Accordingly, GC-based investigations have been vital for the successful development of the oxylipin field and many of the steps in the metabolic pathway for AA were elucidated using the conventional techniques of thin layer chromatography (TLC) and GC. For GC-based work, initially flame ionization (FID) and electron capture (ECD) detectors were employed, but the development of mass spectrometer detectors increased the specificity of the measurements. The GC-MS methods have been a staple of oxylipin measurements for several decades.^476,477^ This approach requires derivatization of the oxylipins, generally targeting the carboxylic acid to form the methyl ester with diazomethane or the trimethylsilyl (TMS) ester, while pentafluorobenzyl bromide has been used to form the pentafluorobenzyl esters.^478^ In addition, oximes of keto-metabolites can provide additional benefits, and have been used to profile an array of structurally labile plant octadecanoids.^479^ Unfortunately, poor sensitivity due to substantial fragmentation can be a limiting factor in GC-MS detection strategies. The formation of pentafluorobenzyl esters can limit fragmentation and greatly enhance sensitivity. However, the bulk of this derivative can overwhelm the chromatographic resolution of structurally simple regioisomers like epoxides. In this case, regioisomer purification prior to analysis may be required prior to detection if regioisomeric abundance is of interest. This limitation can be overcome by epoxide-directed derivatizations.^480^ For example, the use of 2,3,5,6-tetrafluorobenzenethiol to derivatize arachidonate and linoleate epoxides was demonstrated, with the hydroxysulfanyloctadecanoids having detection limits of 5 pg/μL.^480^ An issue with TMS esters is that they can be readily hydrolyzed and therefore require care in their handling and storage. While still a useful method for oxylipin measurement, the predominant application has shifted away from GC-MS with the advent of LC coupled to electrospray ionization (ESI).

LC-MS has become the preferred method for measurement of oxylipins. While GC-MS and LC-MS have comparable sensitivity in terms of lower limit of detection (LLOD) and lower limit of quantification (LLOQ), the initial primary advantage of LC-MS was its simplicity due to a lack of need for derivatization. For in-depth reading, multiple reviews have been written on the LC-MS quantification of oxylipins.^481−490^ The majority of the LC-MS methods employ solid phase extraction with mixed-mode or reversed phase sorbents as a cleanup and concentration step prior to measurement, limiting matrix specific ion suppression. The extraction protocols are optimized on a matrix- and analyte-specific basis and can significantly affect both absolute and apparent oxylipin recoveries. Generally, the methods employed for octadecanoids are identical to those used for other oxylipins and these efforts are not reviewed here.^491^ The key points include evaluation of recovery, matrix-effects, and ion-suppression. More recently, some simplified analysis schemes have emerged using protein precipitation by organic solvent (e.g., acetonitrile, methanol). While this approach can be associated with considerable matrix and ion-suppression effects, if sufficient dilutions are employed, robust and sensitive methods can result.^399,492^ These methods routinely target over 100 oxylipins along with other metabolites, with quantification in good agreement with methods using more extensive cleanup. For many oxylipins, there is a potential for artifactual formation (or destruction) during the sample collection and cleanup steps. In particular, the activation of platelets during blood collection can result in commensurate increases in LOX-derived compounds (e.g., 12-HETE, 15-HETE, leukotrienes)^493^ as well as thromboxanes. This issue is less relevant for the octadecanoids (Table 3), which are less dependent upon LOX and COX activity (in mammals). In studies of oxylipin levels in tissue samples, it is important to instantaneously stop enzymatic generation of products during handling (i.e., by freezing in liquid nitrogen or addition of an organic solvent such as 2-propanol). Artificial formation by autoxidation can be minimized by adding butylated hydroxytoluene (BHT) or another antioxidant; however, many analytical workflows for oxylipin quantification no longer use antioxidants. Despite these challenges, an interlaboratory analysis using a harmonized LC-MS-based protocol for 133 oxylipins was able to achieve low technical variability.^461^ Some of these factors affecting variability in observed free oxylipin levels in mammalian tissues have been recently reviewed.^494^

The dominant trend in the literature for the past 20 years has been the development of increasingly broad coverage oxylipin profiling methods. Newman and colleagues developed one of the first LC-MS profiling methods for linoleate and arachidonate derived epoxides and vicinal diols in 2002,^417^ measuring 13 oxylipins in 31 min with LOQ ⩽1.5 nM. Further developments in the field due to the availability of commercial compounds, advances in column technology, and the attention of dedicated analytical chemists dramatically increased the number of oxylipins that could be analyzed in a single run. Edward Dennis and co-workers developed some of the first large-scale oxylipin metabolic profiling methods in 2007, reporting the analysis of 60 eicosanoids in 16 min.^495^ In 2009, Yang et al. measured 39 oxylipins in 21 min with LOQ ranging from 20 pM-10 nM,^496^ which included for the first time focus on the HODEs, oxo-ODEs, EpOMEs, DiHOMEs and TriHOMEs. A number of general oxylipin profiling methods were subsequently published; however, the octadecanoid coverage was limited to the primary commercially available standards from LA (EpOMEs, DiHOMEs, 9- and 13-HODE, 9- and 13-oxo-ODE as well as the TriHOMEs). The analogous octadecanoids from ALA became commercially available and were commensurately included in the profiling methods (EpODEs, DiHODEs, 9- and 13-HOTrE, 9- and 13-oxo-OTrE). Ramsden and colleagues expanded these efforts to focus on the octadecanoids, measuring 57 oxylipins of which 28 were octadecanoids including novel hydroxy-epoxy- and keto-epoxy-octadecenoic acids.^497^ Multiple oxylipin profiling methods have since been published,^398,487,498,499^ with Schebb and colleagues publishing a number of comprehensive oxylipin panels that include multiple octadecanoid species.^500,501^ A more recent effort focused on the microbial products of C18-FAs and developed a method for 45 different octadecanoids derived from LA, ALA and GLA.^502^ Advances in octadecanoid-specific methods are proceeding in alignment with the availability of the analytical standards.

There have been a number of specialized LC-MS methods developed to improve oxylipin analysis. The use of micro-UHPLC was shown to be useful for low volume analysis of oxylipins.^503^ Polarity switching is regularly used in profiling methods.^504^ Kampschulte et al. developed a multiple heart-cutting achiral–chiral 2D-LC-MS method that enables simultaneous oxylipin quantification and determination of stereochemistry.^505^ In an attempt to decrease the time required for sample preparation and analysis, online SPE-LC-MS/MS methods for oxylipins have been developed.^491^ Nontargeted methods have also been developed using high resolution mass spectrometry (HRMS); however, these methods generally report oxylipin identification at the MS1 level, which results in significant uncertainty for reporting the different oxylipin isomers.^506^ The development of oxylipin-specific libraries will enhance the utility of these approaches as demonstrated by Galano and colleagues who employed molecular networking with high resolution MS/MS data to expand the strategies for oxylipin annotation.^507^ Attempts have also been made to improve the sensitivity via the use of nitrogen-containing derivatization agents including primary amines, secondary amines, aromatic amines, hydrazines and hydrazides, and hydroxylamines.^508^ For example, Bollinger et al. reported a derivatization reagent N-(4-aminomethylphenyl)pyridinium (AMPP) that can be coupled to eicosanoids via an amide linkage to improve sensitivity by 10–20-fold.^509^ This method has not yet been applied to octadecanoids, but was successfully used for the analysis of fatty acids.^510^

Determining the chirality of stereocenters in oxylipins is important for understanding the route of formation as well as biological function. The different approaches for chiral-based analyses have been previously reviewed.^511^ Initial efforts relied on normal phase chromatography;^495^ however, developments in column technologies have resulted in the availability of reversed phase compatible columns.^398^ While initial particle sizes were large (<3 μm),^512^ newer phases have smaller particle size (<2 μm) with the associated improved separation.^513^ For example, Fuchs et al. employed reversed phase chiral chromatography to separate 16 different isomers of the LA-derived TriHOMEs using an >100 min isocratic gradient.^432^ Supercritical fluid chromatography (SFC) has been used to perform chiral analysis, with a recent octadecanoid-specific method capable of simultaneously screening the HODEs, oxo-ODEs, EpOMEs, DiHOMEs and TriHOMES from LA as well as the corresponding analogues from ALA and GLA in addition to multiple microbial metabolites.^514,515^ This method is the first dedicated method for octadecanoid metabolic profiling, requiring the custom synthesis of many of the analytical standards.

In terms of other methods for oxylipin analysis, nuclear magnetic resonance (NMR) has also been used extensively and is ideal for confirmation of synthesized compound structure and purity. NMR is particularly useful for assigning double bond configurations and stereochemistry of oxylipins, which is challenging to perform via mass spectrometry-based methods. Capillary electrophoresis (CE) has been used for analyzing fatty acids, but there is little information on applications in oxylipins.^516^ Limited efforts have demonstrated the ability to measure eicosanoids,^517−519^ LA-derived epoxy octadecenoic acid isomers^520^ and LA-derived hydroperoxides.^521^ Specialized methods such as immunoaffinity chromatography (IAC) are only commercially available for a few eicosanoids.^522^ Ion mobility spectrometry (IMS) has strong potential for increasing the specificity in oxylipin analysis with a combination of IMS- and LC-based separation. In early efforts, Kyle et al.^523^ successfully employed IMS to separate 9-HODE and 13-HODE as well as 9-oxo-ODE and 13-oxo-ODE. Jónasdóttir et al. then demonstrated that differential mobility spectrometry (DMS) was able to separate leukotrienes that partially coeluted by LC-MS/MS.^524^ Recently, Fedorova and co-workers evaluated the implementation of IMS in LC-HRMS workflows for eicosanoids and concluded that while deprotonated ions of eicosanoids were poorly resolved, adducts evidenced good separation.^525^ While the application of IMS to improve the separation of complex oxylipin mixtures is promising, there is currently a lack of collision cross section (CCS) libraries, which is a significant limitation. Recently, mass spectrometry imaging (MSI) approaches have been developed to detect oxylipins in intact tissue. Lanekoff and colleagues successfully visualized prostaglandins in uterine tissue using desorption electrospray ionization (DESI)-based imaging, reporting that PGD_2_ tissue localization and abundance could not be inferred by COX distribution and that it was necessary to perform *in situ* imaging of the prostanoids.^526^ In addition, they developed targeted methods to discriminate prostanoid isomers (PGD_2_ vs. PGE_2_) using cationization with silver ions.^527,528^ Nano-DESI interfaced with a triple quadrupole has been used to image monohydroxylated isomers of AA (e.g., 11-HETE vs 12-HETE);^529^ however, the selectivity of the annotated isomers depended upon unique transitions, which are challenging to establish for oxylipins. Accordingly, it is recommended to confirm the structural identify with an orthogonal approach (i.e., LC-MS of the same tissue). Octadecanoids have also been successfully imaged using a novel DESI-MRM-based approach, visualizing metabolites from both LA and ALA in lung tissue.^530^ Accordingly, the use of DESI-MRM-based MSI to map the spatial heterogeneity of octadecanoids (and other oxylipins) is promising. This approach should be able to provide useful insight into the *in situ* signaling pathways associated with octadecanoid production.

In order to advance octadecanoid research, there is a need to further develop targeted methods for octadecanoid measurement. The use of chiral chromatography to determine the route of formation (i.e., enzymatic or autoxidative) is an important component of octadecanoid method development. However, the biggest obstacle is the paucity of available analytical standards. Commercial suppliers have begun to recognize the increasing interest in octadecanoids and continue to improve their offerings of standards. The microbial-derived compounds are of particular interest and further standards will be required to develop this research area.

## 10 Bioactivity of Octadecanoids

### 10.1 Cellular Targets of Action

Octadecanoids display an array of biological effects with actions dependent on their structure, their site of action, and their context of formation. For the oxygenated octadecanoids, these effects are propagated from interactions with various nuclear receptors (e.g., peroxisome proliferator activated receptors (PPARs), G-coupled protein receptors (e.g., GPCR132; i.e., G2A), transient receptor potential receptors (TRPs) including the TRP vanilloid type 1 (TRPV1), and processes modulating cell surface receptor translocation (e.g., vitronectin receptor)). Importantly, both regio- and enantioselectivity in receptor activation have been reported in some of these processes as discussed below.

**Figure 1:**
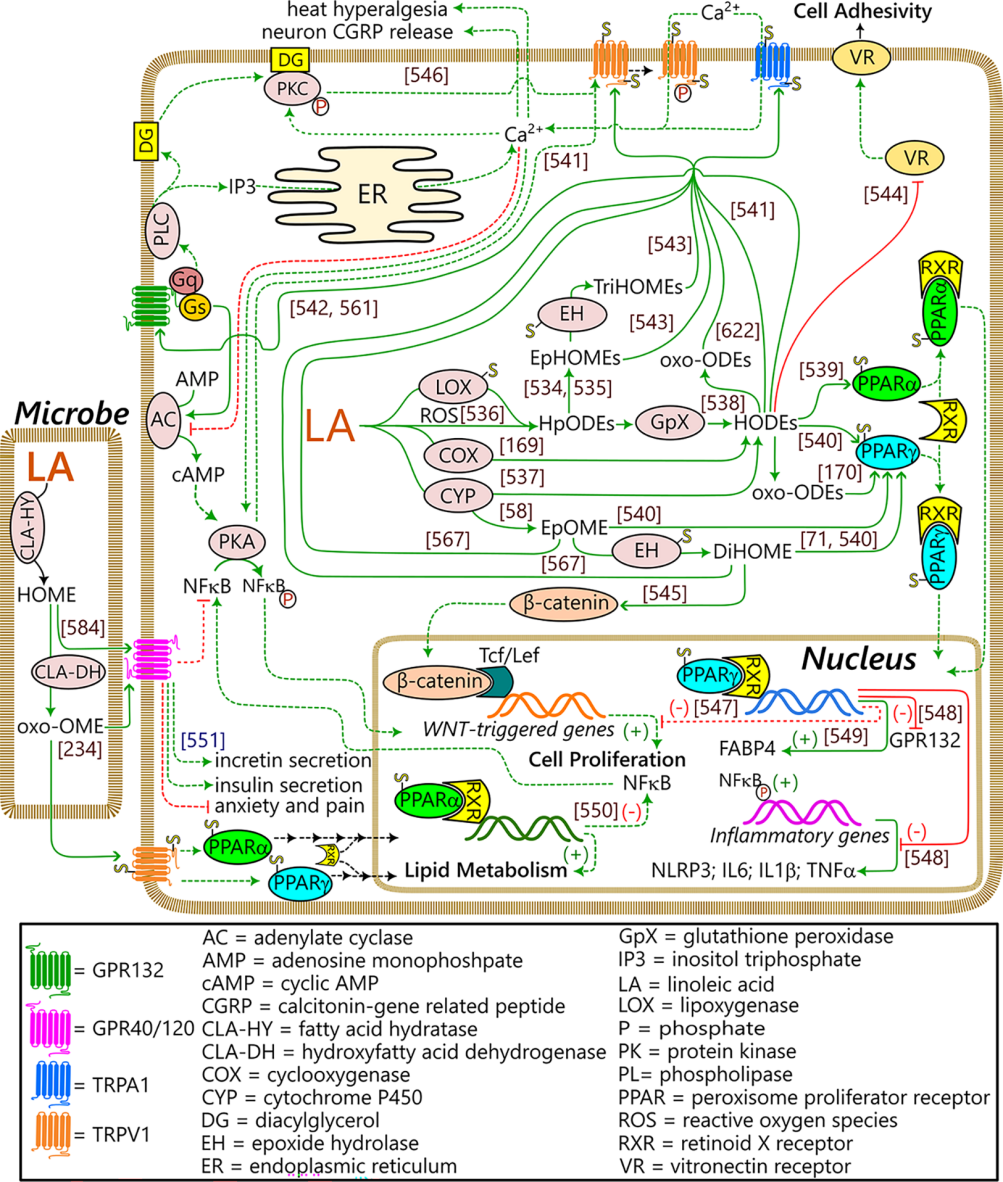
Aggregated octadecanoid mechanisms of action with points of nitro-octadecanoid interactions indicated. Linoleic acid (LA)-derived octadecanoids are shown as an example. Structural analogues are expected to exhibit similar interactions, with differential potency; however, in most cases those data are not yet available. Lines indicate direct (solid) and indirect (dashed) octadecanoid interactions, while line terminations indicate positive (arrow heads) and negative (bars) actions. In mammals, enzymatic and abiotic linoleate oxygenations yield a variety of octadecanoids.^58,169,534−538^ These products reportedly bind PPARs,^170,539,540^ the G-coupled protein receptor GPR132,^534,541^ transient receptor potential (TRP) channels,^542,543^ the vitronectin receptor^544^ and β-catenin.^545^ In addition, the microbial enzyme CLA-HY can produce metabolites in the gut^541,546^ which activate GPR40 (i.e., FFAR1). These ligand binding events influence cell proliferation,^545,547^ inflammation,^71,541,546−550^ lipid metabolism,^549^ cell adhesivity,^541^ and insulin secretion,^551^ along with the central and peripheral perception of pain. Actions within cell types will reflect the availability of biosynthetic machinery, receptors profiles, and downstream response elements, and can be regulated at multiple points by reversible nitroalkylation of redox sensitive thiols, indicated by a yellow S in the displayed system where known. The line colors indicate the directionality of impact (green increase, red increase). Octadecanoid nomenclature is as described in Scheme 3.

For the nitrated octadecanoids, effects are also initiated from multiple interactions including those at nuclear receptors (e.g., Nrf2, PPARs, NFκB, heat shock response, stimulator of interferon genes (STING)), and cellular receptors (e.g., angiotensin II type 1 receptors), as well as by direct modulation of enzyme activity (e.g., soluble epoxide hydrolase (sEH), 5-LOX, xanthine oxidase).^531^ These actions appear to largely stem from their ability to participate in reversible Michael additions with reactive thiols.^532^ Recent efforts identified 184 high confidence intracellular NO_2_-FA targets in macrophages, expanding the reported list to include such important targets as the retinoid X receptor and Toll-like receptors.^533^ The aggregate activity of the effects of oxygenated octadecanoid along with points of known NO_2_-FA interactions are shown in Figure 1.

PPARs are important octadecanoid sites of action. Eighteen carbon alcohols, ketones, epoxides, and nitro-lipids are all ligands, activators and suppressors of PPARs.^170,552−556^ For instance, 13-HODE can activate PPARα and PPARγ, yet suppress PPARδ, while 9(10)-EpOME is a PPARγ activator.^540,557^ In addition, substantial regio- and enantioselective interactions with PPARs are reported. 13-HODE is the most potent PPARγ activator within the linoleate derived alcohols, while the autoxidation product 10(*E*),12(*E*)-9-HODE is the weakest.^558^ Moreover, while 10-HODE, 12-HODE and 9(*E*),11(*E*)-13-HODE increase PPARγ-mediated transcription, the 10(*E*),12(*E*)-9-HODE regioisomer reduces this action.^558^

The stress inducible GPR132 is another important site of action for the HODEs. While the downstream effects of GPR132 vary by cell type and the coactivation of other processes, it was first identified as a factor causing the accumulation of cells in G2/M of the cell cycle with tumor suppressor-like properties.^559^ GPR132 is activated by oxidized nonesterified fatty acids, but not their esterified forms, with 9-HODE being a particularly potent activator.^542,560−562^ Moreover GPR132, is transcriptionally suppressed by PPARγ activation, indicating a point of potential regulatory cross talk by octadecanoids.^548^ For example, in the context of atherosclerosis, the early activation of 15-LOX-1 leads to 13-HODE production and PPARγ-dependent enhanced lipid clearance. Moreover, HODEs may also be involved in atherosclerosis risk reduction. For instance, 13-HODE may increase reverse cholesterol transport early in atherosclerosis through PPARα activation.^563−565^ It has been suggested, however, that with the oxidative stress-dependent generation of racemic 9- and 13-HODEs, 9-HODE-driven GPR132-dependent pro-inflammatory processes take over.^534^

HODEs are also reported activators of the TRPV1, but their importance as endogenous regulators of TRPV1-dependent physiology (e.g., nociception) is still debated.^566^ Regardless, in the context of chemically induced oxidative stress and nociception in the dorsal root ganglion, 9-HODE-dependent activation of GPR132-PKC-TRPV1 coupled signaling appears credible.^546^ In addition, EpOMEs and DiHOMEs are also activators of both TRPV1 and TRPA1.^567^ A comprehensive evaluation of octadecanoids as effectors of the TRP-superfamily would appear to be extremely valuable and may unravel potential mechanisms of action in various cell systems.

The electrophilic NO_2_-FAs impact physiological processes by modulating the function of a variety of proteins through the reversible modification of regulatory thiol and histidine residues. Such processes have been extensively reviewed elsewhere, but are summarized and integrated here.^76,532^ These NO_2_-FA targets include redox sensing mechanisms (e.g., Keap/Nrf2), metabolic and growth regulators (e.g., glyceraldehyde-3-phosphate dehydrogenase, Rad51, PPARs) and inflammatory modulators (e.g., NFκB), along with other targets with broad physiological impacts (e.g., sEH, TRPV1). It is particularly noteworthy that a variety of protein targets of electrophilic lipids have been identified, and while only a subset of these have been investigated for interactions with the NO_2_-FAs, these lipids do share some specific amino acid targets with other electrophilic lipids like 15-deoxy-Δ^12,14^-prostaglandin J_2_ (15-deoxy-Δ^12,14^-PGJ_2_) and hydroxynonenal.^76^ The demonstration of cellular NODE catabolism, with prostaglandin reductase-1 identified as a functional nitroalkene reductase, has also provided a satisfying endogenous mechanism to halt such nitro fatty acid-dependent signaling cascades.^568^ Therefore, this mechanism alone allows the NO_2_-FAs to have broad actions in both plants and animals, many of which likely remain to be described.^105,314,569^

### 10.2 Inflammation and Immunomodulation

Octadecanoids, like other oxygenated fatty acids including eicosanoids and docosanoids, are involved in the regulation of inflammatory processes, with both pro- and anti-inflammatory activity. For instance, μM concentrations of 13-HpODE, but not 13-HODE, stimulate RAS-dependent inflammatory signaling cascades and induce the formation of the transcription factor NFκB in vascular smooth muscle cells.^570^ However, both 9- and 13-HODEs can induce the maturation of monocytes into macrophages through PPARγ-dependent processes.^571^ The 15-LOX-1 dependent formation of 13-HODE has numerous demonstrated anti-inflammatory effects. For instance, Iversen et al. showed that 13-HODE inhibits the human neutrophil production of LTB_4_*in vitro*.^572^ Another LOX product, the nonvicinal diol 9,16-DiHOTrE, has been shown to inhibit recombinant COX and 5-LOX, and decrease the production of pro-inflammatory mediators including LTB_4_ and prostaglandins. The effect was the same for both the 9(*R*),16(*S*)- and 9(*S*),16(*S*)-stereoisomers.^101^ 13-HODE can also inhibit platelet-activating factor (PAF)-induction, but amplifies formyl-methionyl-leucylphenylalanine (fMLP)-induced polymorphonuclear leukocyte degranulation.^573^ Alternatively, GRP132 activation by 9-HODE, but not 13-HODE, stimulates pro-inflammatory cytokine production, and cell cycle arrest in normal human epidermal keratinocytes,^562^ but not GRP132-dependent activities in macrophages.^574,575^ Anti-inflammatory effects were also demonstrated for 13-HOTrE and the corresponding hydroperoxide 13-HpOTrE, with both compounds decreasing pro-inflammatory cytokine/enzyme levels while simultaneously increasing anti-inflammatory cytokines in LPS-challenged RAW 264.7 and mouse peritoneal macrophages. This anti-inflammatory activity is induced by inactivation of the NLRP3 inflammasome complex via activation of PPARγ. Additionally, both metabolites also deactivated autophagy and induced apoptosis in LPS challenged macrophages.^67^ The role of ALA metabolites in the resolution of inflammation and immunomodulation have been extensively reviewed by Cambiaggi et al.^576^

Ketones produced from both LA and ALA have been found to exert anti-inflammatory effects through PPAR-mediated processes. Altmann et al. showed that 13-oxo-ODE can activate PPARγ, inducing transcriptional repression of pro-inflammatory factors and ameliorating colitis and mucosa inflammation in human epithelial colon cells,^170^ whereas the two ALA-derived ketones, 9-oxo-OTrE and 13-oxo-OTrE inhibited inflammatory responses by significantly decreasing nitric oxide (NO) and TNF-α release in a LPS-stimulated RAW 264.7 macrophage cell line.^82^ The anti-inflammatory role of ALA was evaluated in M1-like macrophages from THP-1 cells, resulting in an increase in both ALA- and LA-derived octadecanoids and a reduction in LPS-induced IL-1β, IL-6, and TNF-α production.^577^ The authors concluded that ALA and its associated octadecanoids may act to dampen the inflammatory phenotype of M1-like macrophages.

A role for epoxy and dihydroxy octadecanoids in inflammatory processes is also slowly emerging. For instance, the generation of epoxides from LA during inflammation limited the accumulation of pro-inflammatory Ly6chi monocytes and pro-inflammatory phenotype macrophages.^69^ The 9(10)-EpOME and 9,10-DiHOME can activate inflammation-associated transcription factors NFκB and AP-1,^287^ while 9,10-DiHOME promotes neutrophil chemotaxis^578^ and inhibits the neutrophil respiratory burst.^579^ Interestingly, acute inflammation causes a rapid regiospecific decline in the constitutively high levels of EpOMEs in the mouse peritoneal cavity, with the 9(10)-EpOME being more affected than the 12(13)-regioisomer.^69^ These changes paralleled responses of 9-HODE to this acute inflammatory challenge. Notably, serum levels of 12,13-DiHOME were significantly elevated in severe burn-injured mice, causing immune cell dysfunction through hyperinflammatory neutrophilic and impaired monocytic actions.^68^ A 12,13-DiHOME-induced neutrophil dysfunction has been implicated in intralipid-associated immunosuppression in men (no investigation was performed in women).^580^ Importantly, the 12,13-DiHOME has also been identified as a gut microbiota-derived octadecanoid that can impact the inflammatory state of the host. Two studies from Lynch and colleagues demonstrated increased levels of 12,13-DiHOME associated with overexpression of bacterial sEH in neonates with atopic asthma.^70,71^ Intra-abdominal treatment with 12,13-DiHOME induced pulmonary inflammation and decreased the number of regulatory T (Treg) cells in the lungs of mice.^71^ Similarly, treatment of human dendritic cells with this diol reduced anti-inflammatory cytokine secretion as well as the number of Treg cells *in vitro*, via the alteration of PPARγ-regulated gene expression.^71^ The 12,13-DiHOME also facilitated the maturation and activation of stimulated neutrophils, while impeding monocyte and macrophage functionality and cytokine generation. A recent study demonstrated a pro-inflammatory role for 12,13-DiHOME by enhancing NLRP3 inflammasome activation in human macrophages.^581^

Recent work has further highlighted the potential for microbial octadecanoids to influence mammalian inflammation. Two ALA-derived metabolites produced by lactic acid bacteria, 9(*Z*),15(*Z*)-13-HODE and 9(*Z*),15(*Z*)-13-oxo-ODE, were shown to favor the differentiation of macrophages into the anti-inflammatory M2-type via activation of GPR40 receptor.^582^ The LA-derived 12(*Z*)-10-HOME, produced by gut bacteria, was recently identified as a potent mediator of inflammation. Both the 12(*Z*)- and 11(*E*)-isomers are PPARα agonists, while the 12(*Z*)-isomer is recognized by GPR40.^583^ Administration of 12(*Z*)-10-HOME resulted in reduced intestinal inflammation in DSS-induced colitis and protection from fat-induced obesity in mice.^584^ The same compound was found to down-regulate the pro-inflammatory response while activating the nuclear factor erythroid 2 (NF-E2) p45-related factor-2 (Nrf2)-induced cytoprotective defenses in LPS-matured dendritic cells and MODE-K murine intestine cell lines. Moreover, 12(*Z*)-10-HOME stimulated an anti-inflammatory cytokine pattern and dampened the production of pro-inflammatory mediators in the extracellular space of LPS-matured dendritic cells.^585^ Finally, the production of 12(*Z*)-10-HOME by gut bacteria reduced the production of pro-inflammatory mediators from arachidonic acid by diverting the excess of LA from the eicosanoid cascade.^584^

**Figure 2:**
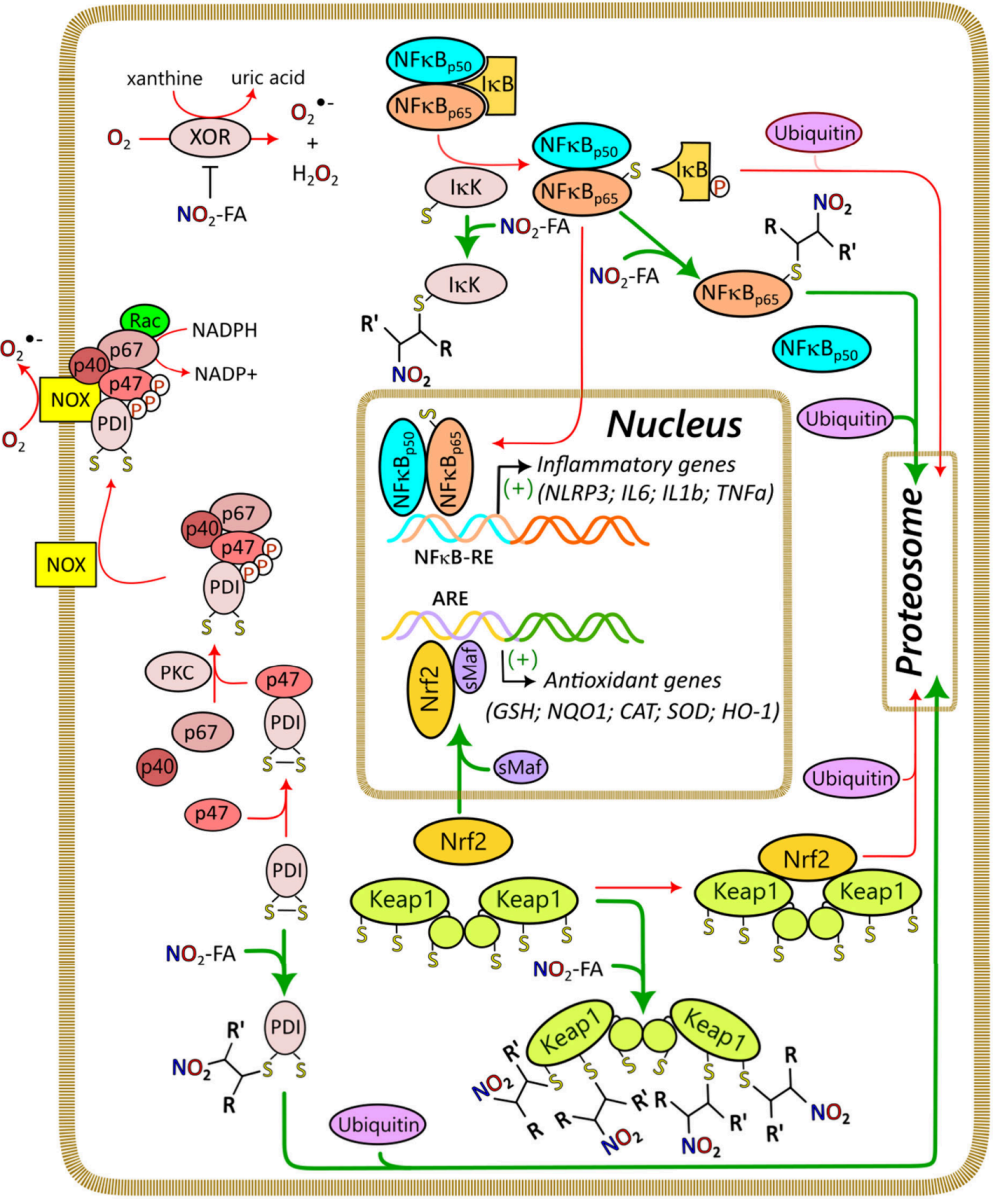
Nitrated fatty acid (NO_2_-FA)-mediated anti-inflammatory and cytoprotective effects. The nitration of fatty acids, particularly conjugated fatty acids, produces reactive electrophiles that can participate in Michael additions with exposed reactive thiols (S) in a multitude of proteins to elicit an array of anti-inflammatory and cytoprotective effects. For example, direct nitro alkylation of xanthine oxidoreductase (XOR) diminishes its activity, reducing the production of reactive oxygen species (ROS). In a more complex interaction, nitro alkylation of the chaperone protein disulfide isomerase (PDI) directs this protein to proteosomal degradation, preventing p47 oxidation, protein kinase C (PKC)-dependent phosphorylation and assembly into the NADPH oxidase (NOX) activating complex, again reducing ROS production. NO_2_-FA modifications to Keap1 (Kelch-like ECH-associated protein) prevent de novo nuclear factor erythroid 2-related factor 2 (Nrf2) capture, ubiquitination, and degradation. The resulting Nrf2 buildup translocates to the nucleus followed by heterodimerization with small musculoaponeurotic fibrosarcoma protein (sMaf). The Nrf2–sMAf heterodimer binds to the antioxidant response element (ARE) transactivating a battery of antioxidant and detoxification genes (e.g., glutathione (GSH), NAD(P)H quinone dehydrogenase 1 (NQO1), catalase (CAT), superoxide dismutase (SOD), and heme oxidase (HO-1). Nitroalkylation of NF-κB p65 at Cys-38 also results in proteasomal degradation of this component reducing the availability of the NF-κB heterodimer of this redox-sensitive transcription factor. NO_2_-FAs can also nitroalkylate IκB kinase (IκK), retarding IκB-activated NF-κB-dependent inflammatory signaling. The line colors indicate the directionality of NO_2_-FA impact (green increase, red decrease).

Nitro fatty acids also exhibit an array of anti-inflammatory properties.^586,587^ For instance, 10-NOME and 9- and 12(*E/Z*)-NODEs can reduce monocyte chemoattractant protein-1 (MCP-1) and interleukin 6 production.^588,589^ In addition, by forming reversible post-translational modifications of Kelch-like ECH-associated protein (Keap) 1, NODEs can lead to nuclear factor (erythroid-derived 2)-like 2 (Nrf2) release from the Keap1/Nrf2 complex, with subsequent Nrf2 translocation to the nucleus and the induction of an array of antioxidant genes.^590−592^ The direct modification of NFκB by NODEs can also inhibit its inflammatory signaling capacity.^591^ The cyclic guanosine monophosphate–adenosine monophosphate (i.e., GMP-AMP) synthase-stimulator of interferon genes (cGAS-STING) signaling pathway is a fundamental system involved in the innate and adaptive immune response to infection and inflammation. Activation of this pathway stimulates both interferon regulatory factor3 (IRF3) and NFκB activation and an array of downstream pro-inflammatory responses (Figure 2).^593,594^ The modification of the transmembrane portion of STING at Cys88 and Cys91 10-NOME blocks the palmitoylation-dependent recruitment activation of this pathway, suppressing interferon release in both human and murine cells.^595^

In addition to disrupting inflammatory signaling cascades at the receptor and transcription factor level, the NOMEs can also modulate the function of a variety of enzymes involved in oxylipin biosynthesis and degradation. For instance, NODE interactions with catalytic cysteines are able to inactivate lipoxygenase as a class.^105,596^ In contrast, NODE-additions to Cys-521 of the murine sEH inhibits fatty acid epoxide hydrolysis and would be expected to have an anti-inflammatory effect by extending the action of endogenous epoxy-fatty acid.^161,597,598^

### 10.3 Octadecanoids in the Mammalian Skin Permeability Barrier

The integrity of the mammalian skin permeability barrier depends on octadecanoids and specifically on LA and its LOX products. The classic paper by Burr and Burr in 1929 first described the existence of dietary EFAs and noted that a feature of EFA deficiency is development of a scaly skin and its resolution by topical application of EFA.^599^ The ability of different fatty acids to correct the symptoms of EFA deficiency was studied in detail during the subsequent 50 years (most commonly by monitoring correction of the trans-epidermal water loss), and the results indicated a structural requirement for at least two double bonds positioned as in linoleate and suitable for transformation by a lipoxygenase.^15^ Other ω6 fatty acids such as arachidonate can cure the scaly skin symptoms of EFA deficiency, although evidence indicates their retro-conversion to linoleate for functionality in the epidermis.^600^

Ichthyosis, named for the fish-like scaly skin, is a human congenital disease that mimics the phenotype of EFA deficiency; it occurs in rare human families with an inactivating mutation in a gene critical for skin barrier formation.^601^ There are over a dozen lipid-related genes whose primary action is epidermal barrier formation,^601^ and these include three working in series on linoleate, starting with 12(*R*)-LOX, then eLOX3,^602^ then the short-chain dehydrogenase-reductase SDR9C7.^603^ Loss of any one of these results in ichthyosis in humans while the gene knockout in mice has a neonatal lethal phenotype; rodent pups cannot survive the trans-epidermal water loss and die of dehydration within hours of birth.^603−606^

**Scheme 81:**
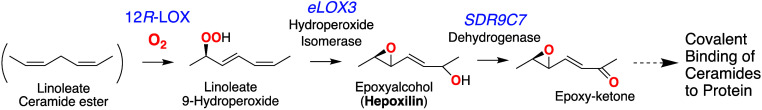
Production of oxo-EpOMEs in the Skin

Linoleate in the outer barrier layer of the epidermis is esterified in the skin-specific Ceramide-EOS (Esterified Omega-hydroxy Sphingosine), and is the substrate for 12(*R*)-LOX, eLOX3, and SDR9C7 (Scheme 81). As shown in Scheme 81, 12(*R*)-LOX forms the 9(*R*)-hydroperoxide on linoleate esters, which is isomerized by eLOX3, and the NAD-dependent dehydrogenase SDR9C7 oxidizes the 13-hydroxy group to the ketone.^180,603^ These transformations are required for the covalent binding of ceramides to the epidermal barrier proteins,^180,603,606,607^ forming a substructure known as the corneocyte lipid envelope, detectable by electron microscopy.^608^ Each of the three mouse knockouts results in loss of covalent binding of ceramides and absence of the corneocyte lipid envelope.

The pathway to the linoleate triols becomes especially prominent after knockout of the *Sdr9c7* gene in mice. The esterified linoleate triol levels increase 100-fold in mouse epidermis after genetic disruption of *Sdr9c7*.^603^

**Scheme 82:**
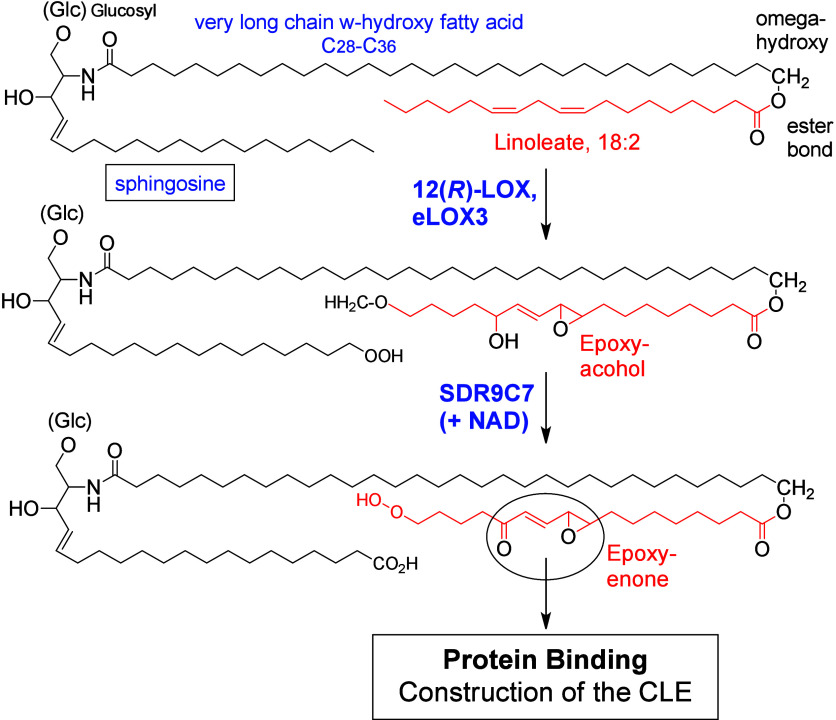
Oxidation of Ceramide-Esterified Linoleate Leading to the Construction of the Corneocyte Lipid Envelope (CLE) The linoleate alkyl chain is shown in red.

**Scheme 83:**
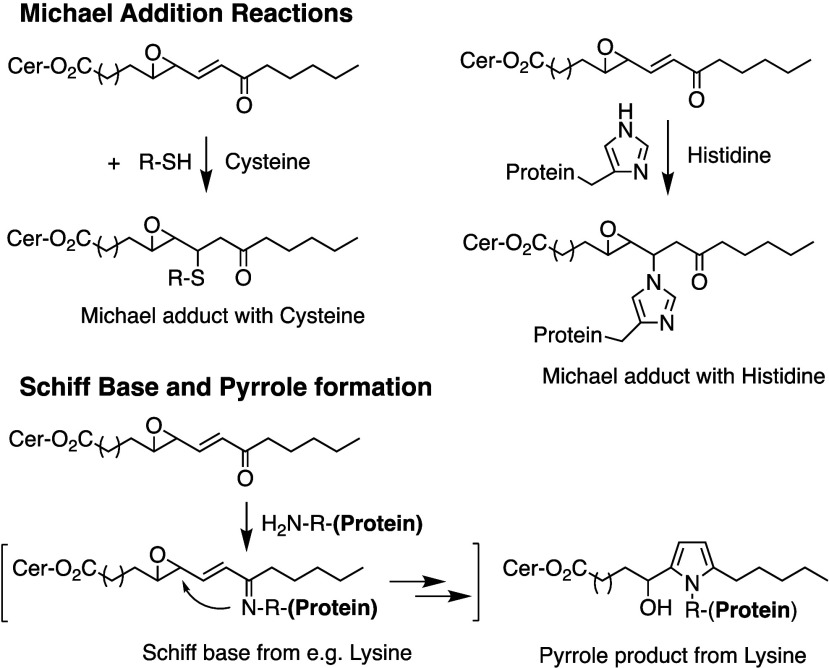
Formation of Pyrrole Adducts on Ceramide-Esterified oxo-EpOMEs

This result points to a rapid turnover of the pathway, with a large buildup of triols as a side product when the route to the epoxy-enone is disrupted. The epoxide hydrolase EH3 (EPHX3) was shown to be responsible for epoxide hydrolysis of the esterified Cer-EOS-epoxyenone in epidermis.^187^ The chemical reactivity of the epoxy-enone moiety is the basis for a postulated mechanistic link between the requirement for the 12(*R*)-LOX pathway of Cer-EOS metabolism and covalent binding of the ceramides (Scheme 82). Simple chemical inspection of this substructure indicates its proclivity for formation of Michael adducts with cysteine and histidine and Schiff base formation with lysine (Scheme 83).

The reactions of linoleate epoxy-enone methyl esters with the free amino acid lysine were reported in 1995 and shown to include the formation of pyrrole adducts.^609,610^ Pyrrole formation would represent a stable adduct formation although reaction with the epoxy-enone methyl esters *in vitro* was extremely sluggish under the conditions reported (CH_3_CN/H_2_O, 7 days at rt). The reactions of analogues of the linoleate epoxy-enone with imidazole nucleophiles were studied by Sayre and co-workers.^339^ Rates of reaction of the epoxy-enones with imidazole and NR-benzoyl-l-histidine were reported and the adducts identified by LC-MS. Nonetheless, in a direct comparison of the reactions of lysine, histidine, serine and cysteine with a synthetic keto-epoxide analogue, only Cys adducts were detectable after 1h reaction at 37 °C in physiological buffer, and Ohno and co-workers went on to identify the adducts as formed by Michael addition to the conjugated enone.^607^ Michael addition is a facile and potentially reversible reaction, and back in 2011, along with the original characterization of LOX involvement in the metabolism and covalent binding of ceramides to protein, reversibly bound Cer-EOS-keto-epoxide was isolated from covalent attachment to mouse epidermal proteins.^180^ Ohno et al. made use of this reversibility to compare the proportions of reversibly and irreversibly bound ceramide covalently bound to mouse epidermal proteins; the results indicated 60% of the total as reversibly bound, with 46% as irreversibly bound^607^ (the latter potentially comprised of ester conjugates with glutamate as reported for human epidermis in 1998^611^). The raison d’être of the LOX metabolism of the epidermal barrier lipids is to promote the covalent binding of ceramides and formation of the CLE,^180,603^ and as the evidence stands, a major component of the Cer-EOS oxidized through the 12(*R*)-LOX pathway is covalently bond via Michael addition to Cys residues in the skin barrier protein.^611^ While not reported to the best of our knowledge, it is interesting to speculate that ^•^NO_2_-dependent nitration of the epoxy-enone could also occur, as elevated levels of (*E*,*Z*)-NODEs can accumulation in inflamed skin.^612^

Other LA regioisomers play important roles in the correct function of human epidermis. Sebaleic acid, the 5(*Z*),8(*Z*)-regioisomer of LA, is secreted by sebaceous glands and is the main component of sebum and of sebaceous cell membrane phospholipids. Increased sebum production results in localized imbalance of fatty acid abundance in skin, with locally decreased levels of LA, which is replaced by sebaleic acid, in follicular epithelium. This was identified as a factor to potentially favor the insurgence of acne in hyperseborrheic individuals.^613^ A more recent study identified a bioactive metabolite of sebaleic acid, 6(*E*),8(*Z*)-5-oxo-ODE, which stimulated calcium mobilization in human neutrophils and induced desensitization to 5-oxo-ETE (5-KETE), but not LTB_4_, indicating that this effect was mediated by the oxo-eicosanoid receptor (GPR170). 6(*E*),8(*Z*)-5-oxo-ODE and its 8-*trans*-isomer were equipotent with 5-oxo-ETE in stimulating actin polymerization and chemotaxis in human neutrophils. Because of these chemoattractant properties, 6(*E*),8(*Z*)-5-oxo-ODE could be involved in neutrophil infiltration and support acne and seborrheic dermatitis.^191^

### 10.4 Nociception

The oxylipins involved in persistent pain states (both eicosanoids and octadecanoids), their biosynthesis and role in inflammation and pain, the corresponding activated receptors, as well as the therapeutic implications of targeting lipid signaling in chronic and neuropathic pain were recently reviewed by Osthues and Sisignano.^614^ Far less has been reported on the potential for NO_2_-FAs in the regulation of pain.

In the past decade, key studies performed by the research groups of Hargreaves, Ramsden, and Sisignano have unveiled that octadecanoids are key mediators of nociceptive processes. In particular, LA-derived octadecanoids play a pivotal role in thermal and mechanical pain modulation, as well as in itch perception, acting both locally and systemically.^61,222,615^ The molecular mechanisms behind nociception in rats were investigated and summarized by Domenichiello et al.^62^ High levels of LA-derived octadecanoids are present in skin due to enrichment of LA, as well as the elevated expression of genes coding for oxylipin production, and have been demonstrated to interact with the main receptors involved in the pain circuit.^62,616^ For example, high levels of 9- and 13-HODEs are produced in mouse and rat skin after exposure to noxious heat,^617^ and were significantly increased in inflamed paw tissue and in the corresponding dorsal root ganglia in the subchronic phase of inflammation.^618^ These HODEs, as well as their oxo derivatives 9- and 13-oxo-ODEs, activate TRPV1 the main receptor for heat perception in the peripheral nervous system,^617^ leading to allodynia and hyperalgesia *in vivo* in rats.^615^ Hargreaves and co-workers determined that the biosynthesis of HODEs and oxo-ODEs in sensory neurons was due to the activity of CYP; treatment with CYP inhibitors completely abolished LA-evoked calcium influx in sensory neurons in inflammatory dental pain, whereas LOX inhibitors had no effect.^619^ As a result, administration of the selective CYP3A inhibitor ketoconazole reduced postburn thermal allodynia in rat hindpaw skin by preventing TRPV1 activation by HODEs and oxo-ODEs,^620^ while local treatment with anti-HODE antibody reversed inflammatory heat hyperalgesia.^619^ Oxidative enzymes (e.g., CYP) are rapidly upregulated in immune cells in human dental pulpitis and in trigeminal nociceptive afferent neurons after orofacial inflammation, resulting in a great capacity to generate lipid mediators able to activate the pain circuits.^621^ Other CYP metabolites of LA, 9(10)-EpOME and 12(13)-EpOME, as well as their hydrolysis products 9,10-DiHOME and 12,13-DiHOME were also found to be increased in spinal cord tissue after burn injury. These four octadecanoids activated both TRPV1 and TRPA1, resulting in postburn mechanical and thermal allodynia (i.e., pain from a typically nonpainful stimulus), which could be reversed by treatment with CYP inhibitors.^567^ Activation of TRPV1 by 9(10)-EpOME^622^ and 12,13-DiHOME^222^ resulted in increased TRPV1-dependent calcitonin gene-related peptide (CGRP)-release from sensory neurons in mice. The subsequent effect on thermal pain sensitivity is opposite for the two compounds, with the diol increasing^622^ and the epoxide decreasing pain hypersensitivity. Reduced thermal hyperalgesia could then be achieved by *in vivo* inhibition of sEH, which results in a decreased concentration of the diol in the nerve tissue.^222^

Activation of GPR132 (i.e., G2A) by 9-HODE in a chemically induced model of neuropathic pain was associated with a protein kinase C-dependent TRPV1-sensitization in peripheral sensory neurons.^546^ The concentration of 9-HODE was strongly increased at the site of nerve injury during neuropathic pain,^623^ whereas increased systemic circulatory levels of both 9- and 13-HODEs correlated with pain intensity in women suffering from chronic neck pain.^624^ Blocking the production of the HODEs led to a significant relief of mechanical and thermal hypersensitivity *in vivo*.^617,618^ Increased levels of HODEs, EpOMEs, and DiHOMEs were detected after UVB irradiation in mouse and rat skin. Given that the biosynthesis of these octadecanoids is COX-independent, this finding may explain why COX-inhibitors (e.g., ibuprofen) only show weak antinociceptive effects in UVB-induced mechanical allodynia in rodents.^625^ The identification of a CYP-dependent enzymatic pathway in pain modulation led the authors to propose CYP inhibitors as an alternative to opioids in postburn pain management, given the lower rate of adverse effects and the extremely decreased potential for generating addiction.^567^ The biological functions of CYP enzymes, with a focus on lipid mediators, and the therapeutic implications of CYP inhibitors were discussed in a recent review that focused on CYP2J2.^626^

The eLOX3-derived hydroxy-epoxide octadecanoids are abundant LA metabolites in skin and play a crucial role in nociception. Ramsden and co-workers identified increased concentrations of hydroxy-epoxide and hydroxy-keto octadecanoids in psoriatic skin lesions and observed that injection of these compounds in rodents enhanced scratching behavior. In particular, 9(*Z*)-11-OH-12(13)-EpOME and 12(*Z*)-11-OH-9(10)-EpOME were involved in C-fiber–mediated pain-related hypersensitivity in rats via the release of CGRP from a sensitization of neurons in the dorsal root ganglion. These two metabolites share a 3-hydroxy-(*Z*)-pentenyl-*cis*-epoxide moiety that was identified as the pharmacophore involved in nociceptor sensitization mediation.^61^ The same group also observed that 9(*Z*)-11-OH-12(13)-EpOME elicited pain-related behavior in rats,^627^ and that both 9(*Z*)-11-OH-12(13)-EpOME and 12(*Z*)-11-OH-9(10)-EpOME (as well as the corresponding keto-epoxides 9(*Z*)-11-oxo-12(13)-EpOME and 12(*Z*)-11-oxo-9(10)-EpOME) stimulated trigeminal neurons by eliciting Ca^2+^ responses, suggesting these metabolites as mediators in chronic headaches and craniofacial pain syndromes.^628^ In addition, systemic levels of 9(*Z*)-11-OH-12(13)-*trans*-EpOME correlated with the frequency of headache events in patients suffering from severe chronic daily headache and diet-induced reduction of plasma levels reduced headache hours per day and headache days per month.^61^ Finally, high concentration levels of 9(*Z*)-11-OH-12(13)-EpOME and 11(*E*)-13-OH-9(10)-EpOME were observed in the brain of chronic pain model rats.^627^ These efforts have focused primarily on the LA derivatives and the affinity of eLOX3 for non-LA peroxides has not been determined and involvement of non-LA octadecanoids in nociception has not been explored.^180,629^ However, it would be of particular interest to investigate if octadecanoids deriving from other PUFAs (e.g., ALA, GLA) interacted with the same pain circuit receptors.

More recently, the role of 9,10,13-TriHOME in nociception in mice was identified. Surprisingly, 9,10,13-TriHOME did not induce nociceptive behavior when tested on rats. As discussed above, this compound is present in its esterified form at high levels in the mammalian epidermis and is released through hydrolysis resulting in high local levels on the skin surface. Application of 9,10,13-TriHOME caused a rapid acute/hyper-acute pain response with short duration, indicating rapid inactivation via acylation and esterification back into lipid membranes and dehydrogenase-induced conversion of the alcohol to ketone moieties. 9,10,13-TriHOME caused hypersensitivity to noxious heat and noxious cold via a TRPA1-dependent mechanism, but also required simultaneous activation of TRPV1.^630^

Dietary intervention was shown to affect nociception in mice. A diet enriched in ω6 PUFAs such as AA and LA, mimicking the Western diet, led to the development of pain hypersensitivity, spontaneously active and hyper-responsive glabrous afferent fibers, and histologic markers of peripheral nerve damage reminiscent of a peripheral neuropathy. Both LA and AA were shown to accumulate in lumbar dorsal root ganglia, where the fatty acids are released by PLA_2_ and subsequently oxidized to a range of octadecanoids (e.g., the nociceptive hydroxy-epoxides, HODEs, EpOMEs, DiHOMEs). The nociceptive hypersensitivity could be attenuated by inhibiting PLA2G7 and was completely reverted by switching to an ω3 enriched diet (rich in EPA and DHA but containing lower levels of ALA).^631^ A similar result was also obtained by investigation of systemic levels of oxylipins in rats subjected to diets enriched in LA or OA. In the first case, the plasma levels of pro-nociceptive LA-derived octadecanoids and AA-derived eicosanoids were significantly increased and subsequent reduction with antinociceptive ω3 oxylipins derived from EPA and DHA. The authors, however, did not characterize the rats’ behavior in relation to the diet, but classified the pro- or antinociception property of the oxylipins based on previous literature reports.^632^ EPA and DHA-derived oxylipins were found to be accumulated in the plasma of rats subjected to the OA-enriched diet, a rather unexpected result given the fact that ALA, the precursor of EPA and DHA, was present at the same level in both diets, and OA is not metabolized to either EPA or DHA. This was consistent with the competition between LA and ALA for elongation-desaturation (as reported by Taha et al.),^633^ further raising the possibility that high LA intake may reduce the benefits of EPA and DHA supplementation on the basis of substrate competition. In humans, tissue concentration from ankle punch biopsies in diabetic patients evidenced a correlation between LA concentration and the necessity for neuropathic pain pharmacotherapy; all patients requiring the therapy had LA > 100 nmol/mg tissue. This result, however, was limited by the low number of investigated subjects (*n* = 16) and requires further investigation.^631^

With respect to nitrated species, NO_2_-FA production in inflamed tissues may be involved in the initial activation, and later suppression of TRPV1 and TRPA1 receptors on afferent nerves.^634^ The presence of cysteine rich ankyrin-like repeats in the N-terminus of TRPV1, TRPV1 and TRPC make these proteins sensitive to modifications and activation by multiple electrophiles, including NO_2_-FAs.^635^ For instance, Cys-414 and Cys-421 within the N-terminal intracellular domain are sensitive to electrophile additions,^636^ as are cysteines in the extracellular domain between transmembrane loops 4 and 5 of TRPV1 (i.e., Cys-616, Cys-621, and Cys-633) and TRPA1 (i.e., Cys-621, Cys-641, and Cys-665).^637,638^ Due to the presence of such redox-sensitive regulatory sites in these pain modulating proteins suggests a role for nitrolipids in the regulation of pain.

### 10.5 Cell Proliferation

The 9- and 13-HODEs are able to influence cell proliferation and apoptosis through PPARδ and PPARγ interactions in multiple cell types.^66^ In 1990, Miller and colleagues demonstrated that 13-HODE could reduce the proliferation of skin in guinea pigs suffering from epidermal hyperproliferation induced by a fat-free diet.^639^ Since then, the effect of HODEs in cell proliferation has been recognized and extensively studied. For example, 9-HODE inhibits proliferation and induces apoptosis in monocyte cell lines.^640^ Similarly, the age-related increases in skeletal lipoxygenase metabolism appears to influence bone loss, where 9- and 13-HODEs are shown to suppress β-catenin mediated Wnt signaling, reducing osteoblast proliferation and promoting apoptosis.^641^ Moreover, 9(*S*)-HODE, but not 13(*S*)-HODE (the equivalent (*R*)-enantiomers were not evaluated), inhibited the proliferation of normal human epidermal keratinocytes cells by suppressing DNA synthesis, while inducing the secretion of inflammatory cytokines IL-6, IL-8, and granulocyte-macrophage colony stimulating factor (GM-CSF).^562^ Conversely, 13(*S*)-HODE increased DNA synthesis in Syrian hamster embryo fibroblasts, while the 13(*R*)-enantiomer showed no activity.^642^ The demonstrated enantioselectivity of these processes argues for an enzymatic role in the regulation of the antiproliferative response mediated by octadecanoids.

The role of the HODEs in the processes of cell adhesion, apoptosis, and mitogenesis in cancer has been extensively reviewed by Vangaveti et al. in 2016.^66^ Honn et al. were the first to show that 13(*S*)-HODE is produced by tumor cells and is able to block the deleterious effect of 12(*S*)-HETE, which increases the metastatic potential of low-metastatic melanoma cells. Moreover, 13(*S*)-HODE reduced lung colonization by high-metastatic melanoma cells.^643^ Since then, several studies supported the finding that 13(*S*)-HODE is able to reduce cancer cell proliferation. 13(*S*)-HODE has been shown to restore apoptosis in a human colorectal cancer cell line^553^ and to inhibit cell growth in a dose-dependent manner in breast cancer cell lines via down-regulation of PPARδ.^644^ In addition, 13(*S*)- and 9(*S*)-HODEs decreased cell growth and DNA synthesis of nondifferentiated Caco-2 cells and showed an apoptotic effect via activation of PPARγ. Conversely, 9(*R*)- and 13(*R*)-HODEs are not ligands for this receptor and resulted in increased cell growth and DNA synthesis through a different mechanism, most likely via activation of the COX pathway. Thus, the two enantiomers used different receptors and exerted contrary effects.^65^ Since vitronectin receptors play an important role in cell growth and differentiation,^645^ the 13-HODE-dependent inhibition of vitronectin receptor translocation to the cell surface may also be involved in this compound’s impact on cell growth.^544,646,647^

LA epoxides and diols have also been implicated in the regulation of cell proliferation. The 12,13-DiHOME appears to promote hematopoietic progenitor cell proliferation and mobilization through modulation of canonical Wnt signaling, a process important for neovascularization.^648^ In addition, the EpOMEs, but not DiHOMEs, promote hair follicle stem cycling and enhance hair growth,^649^ and EpOMEs can induce colon tumorogenesis *in vivo*.^650^ A recent study in early stage breast cancer profiled oxylipins and reported that levels of the 9(10)- and 12(13)-EpOME were reduced in plasma from individuals with breast cancer (*n* = 169) relative to healthy controls (*n* = 152), while 9-HODE levels were increased. The authors concluded that the observed oxylipin changes likely reflect the general status of the individual rather than changes in a specific tumor tissue. Notably, intestinal *Fusobacterium nucleatum* infection has been shown to activate TLR4/AK*T*/Keap1/NRF2 signaling leading to increased 12(13)-EpOME production and oncogenesis.^651^ The Keap1-Nrf2 pathway has been described as “the primary protective response to oxidative and electrophilic stress”^652^ and is often disrupted in oncogenic process.^653^ Interestingly, in metastatic brain melanoma, EpOMEs prevented melanoma cell invasion and macrophage polarization into M1-like macrophages.^654^ The NO_2_-FAs including the (*E*,*Z*)-NODEs are potent activators of the Nrf2-Keap1 pathway, thereby upregulating antioxidant gene expression and inhibiting cell proliferation.^590−592,655^ As the (*E*,*Z*)-NODEs also allosterically inhibit the sEH, this linkage between Keap1-Nrf2 signaling and changes in epoxy-fatty acid metabolism present a unifying linkage between oxylipin and NO_2_-FA actions.

NOMEs also have antiproliferative effects linked to nitroalkylation of RAD51 recombinase, a critical component in the DNA repair machinery.^656^ Another important regulator of cell cycle progression, proliferation and senescence are the Ras-GTPase associated signaling pathway.^657,658^ Like other Ras proteins, H-Ras activation is linked to the canonical Raf/MEK/ERK signaling leading to the induction of proliferation and survival genes. However, H-RAS is also linked to PI3K/AKT signaling, which lead to mTOR activation facilitating protein synthesis, migration, and proliferation.^658^ To the best of our knowledge, interactions between NODEs and the Ras proteins have not been reported. However, Ras modification by reactive nitrogen species has been reported.^19^ Moreover, H-Ras Cys184 binding to the electrophilic lipid mediator 15-deoxy-prostaglandin J_2_ (15d-PGJ_2_) prevents palmitoylation and activates Raf/MEK/ERK-mediated signaling, without AKT pathway activation.^659,660^ Together, these results suggest that modulation of H-Ras regulated cell growth by nitro octadecanoids deserves attention.

### 10.6 Mitochondrial Respiration

A significant body of literature exists indicating that EpOMEs and DiHOMEs are negative effectors of mitochondrial function. The LA-derived 9(10)- and 12(13)-EpOMEs were first found in rice plants and suggested to be derived from rice blast fungus.^661^ In 1986, Ozawa and colleagues reported the structures of two LA-derived epoxides that evidenced a potent uncoupling effect in rat liver mitochondria, simultaneously with relaxation of stomach smooth muscle in a dose-dependent manner.^662,663^ The epoxides (9(10)-EpOME and 12(13)-EpOME) were demonstrated to be formed by incubating LA with leukocytes collected from lung lavages, and were therefore termed leukotoxin and *iso*-leukotoxin, respectively.^662^ However, follow-up work stated that the leukotoxin nomenclature was due to the toxic activity toward mitochondrial respiration.^662,664^ The 9(10)-EpOME was shown to be produced by neutrophils in a calcium ionophore enhanced process, while little 12(13)-EpOME was observed.^662,665^ Importantly, in these early investigations, the other products of LA oxidation (e.g., 9-HODE) were reported to not affect mitochondrial function. The 9(10)-EpOME was detected in rat lung lavages after long exposure to hyperoxia (60 h) and in lung lavages obtained from patients with acute respiratory distress syndrome (ARDS).^666^ Additionally, injection of 9(10)-EpOME (100 μmol/kg) in rats resulted in acute edematous lung injury. However, this dose is approximatively 700 times higher than the physiological level of 9(10)-EpOME in human blood (from 1.5 to 5.5 nM according to the Human Metabolome Database). These collective findings suggested that 9(10)-EpOME played an important role in the development of lung injury observed in patients with ARDS.^667^ However, exposure to low concentrations of 9(10)-EpOME (10 μM) caused lung edema and cellular damage without evidence for mitochondrial dysfunction in rats, while, a higher dose of 30 μM induced a significant decrease of the mitochondrial respiration rate associated with decreased ATP content in the lung tissue.^668^ However, the direct cardiac administration of DiHOMEs, but not EpOMEs, leads to ARDS-like symptoms and rapid death in rodents, implicating an epoxide hydrolase-dependent pathology.^669,670^ The effect of anesthetic agents and sedatives upon circulatory oxylipin profiles was examined in a large RCT of healthy males (*n* = 160).^671^ The authors observed that injection of the anesthetic Propofol (which is used to sedate COVID-19 patients who require mechanical ventilation in the intensive care unit) resulted in elevated circulatory levels of the DiHOMEs in contrast to Dexmedetomidine, which resulted in decreased DiHOME levels. Accordingly, the choice of anesthetic used to sedate COVID-19 patients may have implications for the observed DiHOME levels, which are associated with ARDS-related COVID-19 mortality.

Hammock and colleagues have extensively studied the sEH responsible for converting the EpOMEs to the corresponding DiHOMEs as well as the associated biologies.^58,672^ Their collective efforts have established that the EpOMEs are protoxicants that require activation by sEH to the DiHOMEs. For example, the cytotoxicity of 9(10)-EpOME in ARDS observed in severe burn patients was only observed in the presence of sEH. The study showed that the resulting 9,10-DiHOME was toxic to pulmonary alveolar epithelial cells.^669^ These pathological levels of DiHOMEs have been found to have direct adverse effects on mitochondrial function and engage the mitochondrial permeability transition.^673^ In addition, in the context of LPS-induced cardiac inflammation, 12,13-DiHOME induces severe mitochondrial dysfunction and structural abnormalities, while stimulating inflammatory cytokine production.^674^ The 9,10- and 12,13-DiHOMEs also induced renal proximal tubule cell death associated with mitochondrial dysfunction, while the corresponding epoxide did not induce cell death at concentrations up to 1 mM.^675^ Schuster et al. reported that addition of thermally stressed corn (of which the octadecanoid content was determined by LC-MS) to the diet of mice had an effect in the regulation of mitochondrial function and in the activation of the NLRP3 inflammasome. These effects could be ascribed to the high levels of LA-derived octadecanoids (259.6 ± 21.6 nM) in stressed oil, which mediated cell death in hepatocytes through a mechanism partly dependent on capsase-1 activation.^118^ These findings suggest that dietary octadecanoids are able to directly activate the innate immune response linking lipid metabolism with innate immunity. Interesting, *in vitro* studies in RAW264.7 cells suggested that 9(*S*)- and 13(*S*)-HODE might have different cellular functions, with 9(*S*)-HODE potentially possessing cytotoxic properties,^676^ further evidencing the need to focus on chirality.

While pathological levels of DiHOMEs cause severe mitochondrial dysfunction, EpOMEs can induce cell death *in vitro* by uncoupling oxidative phosphorylation.^198,677^ Notable, 12(13)-EpOME was shown to increase mitochondrial non-ADP-stimulated and oligomycin-insensitive (i.e., state 4) respiration and reduce succinate-dependent and oligomycin-sensitive (i.e., state 3) respiration in isolated rabbit renal cortical mitochondria (at relatively high concentrations of 50 mM). Concomitantly, 12(13)-EpOME decreased the potential of the mitochondrial membrane, while 12,13-DiHOME did not exhibit similar effects at these concentrations.^677^ Nowak et al., then showed that pretreatment of rabbit renal tubular cells with 12(13)-EpOME (but not 12,13-DiHOME) before hypoxia protected the mitochondrial functions and accelerated the recovery of the intracellular ATP levels during reoxygenation.^678^ Moreover, sEH deletion or pharmacological inhibition also prevents the development of mitochondrial abnormalities in models of cardiac ischemia/reperfusion inflammation.^674,679,680^ Likewise, NOME perfusion is cardioprotective in a heart ischemia/reperfusion injury model. These findings provide another plausible physiological linkage between the NO_2_-FA-dependent metabolism and the sEH biology. However, NO_2_-FAs are also able to directly inhibit mitochondrial respiration by nitroalkylation-dependent inhibition of complex-II linked respiration.^681^ Mitochondrial adenine nucleotide translocase 1 (ANT1) Cys-57 has also been identified as a cardioprotective target of NO_2_-FA adduction.^682^ ANT1, which can account for up to 10% of total mitochondrial protein,^683^ is a component of the permeability transition pore^684^ that regulates ADP/ATP exchange across the inner mitochondrial membrane and regulates basal proton leakage.^685^ Knockdown of ANT1 is associated with increased mitochondrial ROS, concomitant with increased TNFα-induced NF-κB reporter gene activity and interleukin-6 and TNFα expression.^686^ Therefore, nitroalkylation of ANT1, sEH, and other cellular and mitochondrial targets likely work in concert to elicit an anti-inflammatory and cardioprotective phenotype.

Because the mitochondrial inner membrane is exposed to an oxidant abundant environment and consists of high levels of cardiolipin, a LA-rich membrane lipid when dietary LA is high,^687^ it is not surprising that linoleate oxidation products including HODEs, DiHOMEs, TriHOMEs, oxo-OMEs, oxo-EpOMEs, HpODEs and Hp-oxo-OMEs are constitutive members of the mitochondrial lipidome.^294,688^ Notably, cytochrome c and iPLA_2_γ are likely involved in cardiolipin oxidation and hydrolysis *in vivo*,^294^ and cardiotoxic chemotherapeutics and ω3 fatty acids can modulate their levels.^688^ Regardless, studies of the impact of these species on mitochondrial function are extremely limited. In the context of airway inflammation, 13(*S*)-HODE has been shown to negatively impact mitochondrial function in airway epithelium.^689^ Together, these findings suggest that future efforts to evaluate the potential for cardiolipin-derived octadecanoids as endogenous mediators of mitochondrial function have great potential.

### 10.7 Metabolism and Hormone Modulation

Octadecanoids exert an important role in fatty acid and glucose metabolism as well as in the modulation of hormone secretion because they can activate a number of receptors linked to metabolism (e.g., PPARα,^690^ TRPV1,^691^ GPR40,^584^ prostaglandin receptor EP3^79^). In 1998, Murthy et al. were the first to show that 13-HODE interferes with the assembly and composition of triacylglycerol-rich lipoproteins secreted by intestinal cells and diminishes the secretion of triacylglycerol in intestinal Caco-2 cells.^692^ Interesting recent evidence in drosophila also suggests that 9-HODE stimulates JNK activation of Forkhead box (i.e., FOXO) proteins, which are important regulators of insulin signaling.^693^ In addition, both 9 and 13-HODEs increase fatty acid binding protein 4 (FABP4) expression in leukemia monocyte cell lines in a PPARγ-dependent manner.

While a similar impact of HODEs on adipose FABP4 expression has not been demonstrated, this protein is strongly associated with the development of insulin resistance and atherosclerosis^694^ and research in this area is warranted.^549^ More recently, the role of batokines during cold-exposure or exercise has been studied by Stanford and colleagues, revealing the importance of 12,13-DiHOME in fatty acid uptake and insulin resistance.^695−697^ Lynes et al. demonstrated that 12,13-DiHOME decreases body-mass and insulin resistance in humans.^64^ Interestingly, 12,13-DiHOME has been reported to reduce glucose uptake and inhibit insulin-dependent signaling in myotubes.^698^ 12,13-DiHOME is released from BAT after 1 h of cold exposure in both rodents (4 °C) and humans (14 °C). Moreover, the levels of epoxide hydrolase were significantly higher in BAT after cold exposure, leading to an increased production of the diol from the corresponding epoxide. The increased levels of the circulating diol enhance fatty acid uptake in BAT, which is involved in heat production in adults, and reduce levels of serum triglycerides.^64^ Acute-intensity bouts of exercise caused the release of 12,13-DiHOME from BAT, increasing the circulating levels and enhancing fatty acid uptake and mitochondrial fatty acid oxidation in skeletal muscles.^63^ Acute treatment with 12,13-DiHOME in mice increased skeletal muscle fatty acid uptake and oxidation, but had no effect on glucose uptake. BAT transplantation resulted in improved cardiac functions in mice through the release of 12,13-DiHOME, which negated the deleterious effects of a high-fat diet. Acute injection with 12,13-DiHOME affected the cardiomyocytes by increasing mitochondrial respiration resulting in increased cardiac hemodynamics. Moreover, the systemic concentration was decreased in individuals with heart disease.^221^ More recent studies report that both the 9,10-DiHOME and 12,13-DiHOME are inversely associated with BMI and activate calcium influx in mouse brown and white adipocytes *in vitro*,^699^ further supporting that activating BAT is a promising target to treat metabolic syndrome.

Ketones derived from LA and ALA interact with PPARα, affecting the intake of triglycerides and fatty acids in cells and tissues. Cellular triglyceride accumulation in the hepatocytes was inhibited by 10(*E*),12(*E*)-9-oxo-ODE, a ketone produced from LA and isolated from tomato juice, via activation of PPARα *in vitro*.^690^ The effects were confirmed by *in vivo* treatment of obese KK-Ay mice fed with a high-fat diet, resulting in decreased levels of triglycerides in both plasma and liver.^700^ Activation of PPARα was also reported for 9-oxo-OTrE, resulting in enhanced fatty acid uptake in murine hepatocytes.^701^

Exercise-induced changes in octadecanoids have also been reported, suggesting they may report on metabolic changes associated with metabolic exertion. Elevations in HODEs and DiHOMEs induced by a 75-km bout of cycling were partially resolved in 1.5h, and fully resolved within 1d, further suggesting these compounds as markers of physiologically relevant oxidative stress.^702^ Notably, exercise-induced changes in HODEs were reportedly unrelated to isoprostane formation, muscle damage, or soreness, but negatively correlated with granulocyte colony stimulating factor and interleukin 6 (IL-6), suggesting associations with neutrophil chemotaxis and inflammation in this context.^703^ Given the impact of 12,13-DiHOME on glucose uptake and insulin-dependent signaling in myotubes,^698^ some of these changes may report on physiological changes in muscle energy metabolism.

Interesting metabolic effects were demonstrated for octadecanoids produced by gut microbiota. Alcohols and ketones oxidized at the 10-position can activate the PPARα receptor, while the two regioisomers of 10-oxo-OME (12(*Z*)- and 11(*E*)-10-oxo-OME) can also activate PPARγ, with the former possessing the highest potency. The activation of PPARα by 12(*Z*)-10-oxo-OME caused an increase of adiponectin production and insulin-stimulated glucose uptake.^583^ This ketone was also able to activate the TRPV1 receptor and improve noradrenalin turnover in adipose tissues. The intake of 12(*Z*)-10-oxo-OME by obese and diabetic KK-Ay mice improved obesity-associated metabolic disorders, such as glucose intolerance, insulin resistance, and increased adiposity.^691^ Recently, Miyamoto et al. showed that the corresponding alcohol, 12(*Z*)-10-HOME attenuates HFD-induced obesity in mice. Acute 12(*Z*)-10-HOME administration promoted the secretion of GLP-1, a peptide hormone associated with appetite suppression and improvement of glucose homeostasis via activation of GPR40 and GPR120.^79^ Finally, 12(*Z*)-10-HOME activated the EP3 receptor in the gut, promoting intestinal peristalsis.^79^ Dietary-derived octadecanoids therefore appear to participate in a feedback system in which the gut microbiome converts dietary fatty acids into octadecanoid lipid mediators that attenuate obesity.

Hormone modulating effects were reported for three octadecanoids: a keto-epoxide metabolite of LA, 10(*E*)-9-oxo-12(13)-EpOME, and two gut microbial metabolites, 11(*E*)-10-oxo-OME, 9(*Z*),15(*Z*)-13-oxo-ODE, derived from LA and ALA, respectively. In particular, 10(*E*)-9-oxo-12(13)-EpOME promoted the production of aldosterone, a hormone regulating blood pressure, at low doses (from 0.5 to 5 μmol/L *in vitro*), and inhibited its production at higher doses,^185^ while both 11(*E*)-10-oxo-OME and 9(*Z*),15(*Z*)-13-oxo-ODE stimulated production of a cholecystokinin, a gut hormone that helps digestion and reduces appetite, via activation of GPR40 (further demonstrating the role of octadecanoids in attenuating diet).^234^

There are limited studies investigating the utility of octadecanoids as indicators of endocrine disruption. Markaverich and colleagues showed that THF-diols, but not the DiHOMEs, have been reported to block male sexual behavior.^704,705^ While the mechanism of action is unknown, Okamura’s and Hull’s groups hypothesized that it occurs via modulation of nitric oxide-dependent pathways that control gonadotrophin-releasing hormone release.^706−708^ THF-diols and the DiHOMEs are thought to act additively to disrupt endocrine function in both male and female rats at low concentrations (0.5–1 ppm), which is ∼200-fold lower than those of classical phytoestrogen endocrine disruptors (e.g., isoflavones).^709^ Both DiHOMEs stimulated MCF-7 breast cancer cell proliferation equivalently, but did not compete for [3H]estradiol binding to the estrogen receptor or nuclear type II sites. Oral administration of the DiHOMEs at low doses (>0.8 mg/kg body weight/day) disrupted estrous cyclicity in female rats, but did not disrupt male sexual behavior, suggesting sex-specific differences in endocrine response.^705^

### 10.8 Cardiovascular Effects

Octadecanoids possess diverse effects in the cardiovascular system, influencing cardiac muscle contraction, arterial relaxation, ischemia, and platelet adherence. Injection of very high doses of 9(10)-EpOME in dogs (15 mg/kg) caused an immediate decrease in aortic flow and cardiac failures and death in <1 h.^55^ This depressed cardiac function in dogs in a dose dependent manner. Similarly in cats, injection of 2.5–25 ng of 9(10)- and 12(13)-EpOMEs into isolated papillary muscles reduced myocardial contractility, while administration to carotid arteries resulted in vasoconstriction.^710^ While injection of OA, LA, and SA at 10 mg/kg had no significant hemodynamic changes, injection of 50 mg/kg LA exhibited cardiotoxic effects (although less than those observed with 9(10)-EpOME).^711^ The same effect was observed in guinea pig papillary muscles after acute administration of the gut bacteria metabolite 12(*Z*)-10-HOME at concentrations 30–300 μM.^78^ At a concentration of 10 nM, 13-HODE and its precursor 13-HpODE relaxed canine coronary artery segments with endothelium after PGF_2α_-induced contraction.^712^ This vasodilatation activity is due to stimulation of prostacyclin (PGI_2_) biosynthesis and activation of the thromboxane receptor.^713^ This effect was also observed with 13-HpODE (10 μM) in human pulmonary arteries after PGF_2α_-induced contraction.^712^ Treatment with 9,10-DiHOME (250 μM) increased the coronary resistance in mouse heart after ischemia-reperfusion injury, significantly impairing the heart functional recovery.^714^

Impacts on platelet function and cellular adhesivity are another critical site of octadecanoid action. 13-HODE reduces thrombin-induced platelet aggregation,^715,716^ and platelet adherence to monolayers of cultured pulmonary artery endothelial cells.^715^ Moreover, high density lipoproteins enriched in 13-HODE containing phosphatidylcholine dose-dependently inhibited platelet aggregation.^717^ Notably, in endothelial cells, the intracellular association of 13-HODE with the vitronectin receptor stabilizes it in the intracellular space. Dissociation of this complex appears to allow vitronectin to translocate to the cell surface where it promotes adhesivity.^544,646,647^ The 9(*R*),16(*S*)- and 9(*S*),16(*S*)-diastereoisomers of 9,16-DiHOTrE have also been shown to inhibit collagen-stimulated platelet aggregation through COX-2 inhibition.^101^

Nitro lipids are also potent vasodilators with antihypertensive effects resulting from either the direct modulation of enzyme and/or receptor function, or by influencing the expression of key gene products. As mentioned in the immune modulation and mitochondrial metabolism sections, the sEH is inhibited by nitroalkylation of a redox-sensitive cysteine residue. In mice fed CLA and sodium nitrate, the antihypertensive effects and associated increase in plasma concentrations were ablated by deletion of this redox-sensitive cysteine.^161^ In addition, nitroalkylation of the angiotensin 1 receptor reduces its responsiveness to angiotensin II stimulation,^718^ and the ability of NOMEs to enhance endothelial nitric oxide synthase and hemeoxygenase-1 expression further contribute to the antihypertensive influences of the nitro-octadecanoids.^719^ Therefore, as in other systems, the impact of NO_2_-FAs in the vascular system are pleotropic and dependent on their soft electrophilic characteristics.

## 11 Octadecanoids as Biomarkers

Alteration in octadecanoid levels has been observed in multiple pathologies including neurodegenerative, cardiometabolic, hepatic, and respiratory diseases as well as with chronic pain. Given that octadecanoid fatty acid precursors are generally present in high concentration, and that octadecanoid formation can proceed as a consequence of both oxidative stress and enzymatic activity, monitoring these compounds in the context of disease risk and progression may yield useful biomarkers; however, the specificity of these compounds as independent markers of any given malady is unlikely. The following section provides an overview of the literature to date reporting the potential uses of octadecanoids as biomarkers of diseases. Much work remains to establish the specificity and sensitivity of octadecanoids as biomarkers, either alone or in combination with other metabolites.

### 11.1 Neurological Diseases

The study of octadecanoids in neurological diseases has to date focused primarily on Alzheimer’s disease (AD), with a few studies investigating cerebral ischemia (e.g., stroke) and multiple sclerosis. Yoshida et al. reported a higher level of total-HODEs (defined as 9-(*E*,*Z*)-HODE, 13-(*Z*,*E*)-HODE), 9-(*E*,*E*)-HODE, and 13-(*E*,*E*)-HODE) in plasma and erythrocytes of individuals with AD compared to those with vascular dementia and healthy controls.^720^ Furthermore, the levels of total-HODEs increased with the clinical severity of dementia. The diagnosis of AD was based upon probable AD (according to Diagnostic and Statistical Manual of Mental Disorders IV (DSM-IV) criteria), whereas work by Kurano et al. reported that 13-HpODE levels in brain tissue from autopsies that included histopathological examination correlated with AD clinical phenotype.^721^ Unfortunately, the absolute levels of 13-HpODE, as well as other octadecanoids potentially measured, were not reported. In a larger study of 150 AD patients and 139 healthy controls, Borkowski et al. reported that CSF levels of 9(10)- and 12(13)-EpOMEs were strong predictors of AD.^722^ Morris et al. compared oxylipin profiles in individuals with AD, both with and without T2D, and reported that plasma levels of 9,10-DiHODE and 15,16-DiHODE contributed significantly to the PLS model of AD patients with T2D; however, the associations were weak and not univariately significant.^723^ Shen et al. recently reported a decrease in 13-HODE in male 10-month old AD TgF344 transgenic rats in brain neutral lipids compared to wild-type controls. In AD transgenic females, 13-HODE slightly increased in brain neutral lipids and the anti-inflammatory metabolite 13-HOTrE decreased in phospholipids. Neutral lipid-bound 13-HODE was reduced in AD males at 10 months, but not at 15 months. Compared to wildtype controls, the AD transgenic group of 15 months rats had significantly lower concentrations of esterified 13-oxo-ODE, 9-oxo-ODE and 9(10)-EpOME (decreased by 31%–40%) in brain phospholipids. The reported increases in HODEs further support the involvement of oxidative stress in AD pathology.^724^ The role of oxylipins in AD has been reviewed,^725,726^ with suggestions that ALA-derived octadecanoids may mediate the observed beneficial effects of ALA in AD.^726^

In 2017, Hennebelle et al.^727^ showed the accumulation of LA-oxygenated metabolites in several rat brain regions during CO_2_-induced ischemia. In particular 13-HODE was the most abundant metabolite in the hippocampus and its concentration increased 1.7-fold in cortex and brainstem following ischemia. 9-HODE and 13-oxo-ODE were also increased in cortex by 1.8-fold and 5.6-fold, respectively, in the ischemic CO_2_-group compared to controls, whereas 13-oxo-ODE and 12,13-DiHOME were increased 3.2-fold and 1.4-fold, respectively in brainstem. Additionally 12(13)-EpOME and 9(10)-EpOME increased in both hippocampus (5.7-fold and 2.8-fold, respectively) and cerebellum (2.7-fold and 2.8-fold, respectively) compared to controls.^727^ Follow-up work in 2019 further reported that hypercapnia/ischemia increases brain oxylipin concentrations with observed increases in multiple LA-and ALA-derived octadecanoids.^728^ Interestingly Szczuko et al. in 2020 reported a significant decrease in the levels of 9- and 13-HODEs in the plasma of patients after ischemic stroke.^729^

Alteration in the levels of octadecanoids in brain has been used to predict the insurgence and severity of neurological disorders and age-related cognitive impairment. Swardfager and co-workers were able to correlate the ratios between 9,10-DiHOME and 9(10)-EpOME as well as between 12,13-DiHOME and 12(13)-EpOME (an index of sEH activity) with white matter hyperintensities (WMH) and proposed this value as a biomarker for vascular cognitive impairment in patients with WMH.^730^ The increased diols and reduced epoxides were related to increased sEH activity, which was reflected in increased levels of sEH-derived oxylipins in serum, implicating CYP/sEH-dependent metabolism in the etiology of WMH by injury to the periventricular subcortical white matter. A growing body of literature has supported this observation, and both plasma and cerebrospinal fluid levels of EpOMEs and DiHOMEs have proven to be valuable components of prediction models of neurological disorders. Increased CYP/sEH metabolites (as well as ethanolamides) were found to be strong predictors of AD with ROC curves ranging from 0.82 to 0.92 in CSF and plasma, respectively.^722^ In 2020, Shinto et al. also showed that a higher ratio of 9,10-DiHOME/9(10)-EpOME is associated with increased WMH and poorer performance on the cognitive test Trails-B, and found a positive association between 9-HODE and WMH, and a negative association between 9-HODE and gray matter volume in nondemented people with controlled hypertension (mean age 65 ± 7.1 years), supporting the potential vasoconstrictive effects of 9-HODE.^731^ Moreover, investigations into age related cognitive decline found a negative association between perceptual speed and the 12,13-DiHOME/12(13)-EpOME ratio after adjusting for the omega-3 diol and bile acid components.^732^ More recently, Anita et al. reported that the 12(13)-EpOME was associated with poorer executive function and verbal memory scores in individuals with T2DM, while the 12,13-DiHOME was associated with lower executive function scores.^733^ Of interest, the authors reported an interaction between obesity and the ratio of 12,13-DiHOME/12(13)-EpOME, suggesting that BMI may play a role in the putative effects of these octadecanoids upon cognitive performance.

Multiple sclerosis is a severe inflammatory disorder resulting from an immunological attack on nerve fiber myelination throughout the body.^734^ Villoslada and colleagues identified 13-HODE as a biomarker of multiple sclerosis severity, its level being higher in serum samples of patient with active disease (associated with relapses and increase in EDSS, expanded disability status scale) compared to patient with a stable disease. (The paper refers to 13(S)-HODE although chirality was not measured in the analyses).^735^ Håkansson et al. found significantly higher levels of 9-HODE (average = 380 nM) and 13-HODE (average = 930 nM) in CSF of patients suffering from clinically isolated syndrome or relapsing remitting multiple sclerosis compared to healthy controls (average = 290 nM and 690 nM, respectively). However, the levels of the 9- and 13-HODE did not differ between patients with signs of disease activity during one, two and four years of follow-up and patients without, suggesting that increased HODE levels in patients may be an unspecific sign of neuroinflammation.^736^ Recently, it has been shown that the concentration of esterified 9(10)-EpOME was 206% higher in the neutral lipid pool of multiple sclerosis patients compared to controls. Because 9(10)-EpOME is known to be pro-inflammatory, increased esterification of this octadecanoid may reduce its availability as the free-form, resulting in decreased chronic inflammation associated with multiple sclerosis pathogenesis.^737^

Collectively, these findings suggest that there may be utility in monitoring octadecanoid levels in individuals with neurological disorders, but it is likely that the 9- and 13-HODE associations observed are due to nonspecific inflammation. The use of chiral-based methods will be important to delineate enzymatic vs. nonenzymatic formation of octadecanoids. Further research into other octadecanoid pathways including ALA would be of interest as well as investigations into other neurological diseases including Lewy body dementia and Parkinson’s. For example, the sEH pathway via epoxy-fatty acids has been proposed as a therapeutic target for neuropsychiatric disorders including Parkinson’s.^738^ While the literature suggests that alterations in these oxylipin pathways associate with neurological disorders, a comprehensive analysis of longitudinal changes in octadecanoid levels in relation to cognition is warranted, with particular focus on the putative role of ω6- vs. ω3-derived mediators.

### 11.2 Atherosclerosis

The analysis of oxidized linoleate in arterial atheromatous plaques dates from the early 1970s. C.J.W. Brooks and colleagues first noted the appearance of two classes of polar sterol esters in advanced aortic atheroma, one they identified as polar sterols esterified with normal fatty acids, and the second as cholesterol esterified with 9-HODE, 10(*E*),12(*E*)-9-HODE, or 13-HODE.^739^ They went on to show that the levels of oxidized linoleates were undetectable in “early” lesions (<1 μg/g of lipid), with an increase from 89 μg/g in fibrous plaques and atheromas, to 335 μg/g in more advanced lesions such as thrombotic or ulcerated plaques.^740^ Brooks further extended the analyses in two important ways: the sterol-HODE esters were shown to originate from cholesterol-linoleate hydroperoxides, and stereochemical analysis of the 13-HODE revealed it to be racemic.^741^ The ratio 13(*S*)/13(*R*)-HODEs has been further investigated by Kühn et al., who determined that 15-LOX oxidation contributes to lipid peroxidation mainly in young human atherosclerotic lesions, where the ratio of 13(*S*)/13(*R*)-HODEs is (54 ± 3.2)/(45 ± 3.2). However, in more advanced human lesions, the ratio (50.7 ± 3.5)/(49.3 ± 3.5) is not significantly different to the one obtained by copper treated LDL, suggesting that most of the oxidized lipids of these lesions come from nonenzymatic oxidation.^742^ The results pointed to lipid peroxidation as a factor in the development of atherosclerosis, a finding that has been followed up extensively in the subsequent decades.

By the 1990s, the oxidation of LDL became a major focus of research regarding its involvement in the initiation of atherosclerosis.^743,744^ LA makes up approximately 40–45% of the PUFAs of low-density lipoproteins, which are the main constituent of atherosclerotic plaques. While further studies confirmed the findings of Brooks on the structures, relative abundance, and racemic stereochemistry of the oxidized linoleates in arterial plaques,^745,746^ a key question was the possible involvement of a 15-LOX in initiating the oxidation of LDL and promoting the development of atherosclerosis.^747^ 15-LOX is capable of oxygenating LDL *in vitro*,^748^ and is implicated in the cellular oxidation of LDL.^749,750^ This was examined using an *in vivo* model in which rabbits were fed a cholesterol-rich diet. The esterified 13-HODE in the initial arterial plaques exhibited 74% 13(*S*) chirality, which is evidence of oxygenation by 15-LOX. However, as the atheroma progressed, the 13-HODE became close to racemic, suggesting lipid peroxidation was predominant in the later stages of plaque development.^747^ Mouse knockout of the 12/15-LOX gene tends to support involvement of the enzyme in the early stages of atherogenesis, at least in animal models.^751^ Further work in a rabbit hypercholesterolemia model reported that the 9- and 13-HODE were the most abundant quantified oxylipins in both plaques and plasma,^752^ suggesting that these molecules would be translatable biomarkers of atherosclerosis. While the vast majority of studies have focused on the LA-derived HODEs, a recent review summarized the putative role of ALA-derived octadecanoids in cardiovascular diseases, suggesting potential immunomodulating effects.^576^ However, the reviewed studies were based upon mouse- and cell-based models and did not report utility as biomarkers of disease. In addition, Zhu et al. recently reported that the substitution of plant for animal protein in the diet was a promising strategy to modulate atherogenic lipids in a (apoE^–/–^) mouse model.^753^ A 12-week high plant protein diet increased the abundance of microbiota from the Lachnospiraece family and resulted in a commensurate increase in circulatory levels of the 12,13-DiHOME, which was shown to inhibit lipid accumulation *in vivo*. The authors concluded that a high plant protein diet can alleviate hyperlipidemia via increased microbial production of the 12,13-DiHOME.

While there has been interest in the application of octadecanoids as indicators of atherosclerosis, the field has made few advances beyond the early identification of the 9- and 13-HODEs. This may be partially due to a paucity of studies investigating the octadecanoid content of the plaque in combination with screening for concomitant circulatory signatures. It would be of value to see studies performed in which the octadecanoid content of plaques was determined in conjunction with coronary angiography or other imaging techniques (e.g., CT angiography, ultrasound Doppler) to determine plaque composition and heterogeneity (e.g., calcified, necrotic core, fibroatheroma). This would enable determination of whether specific octadecanoid signatures, particularly as deposited cholesteryl esters, were associated with plaque pathology and/or stability, which could then be potentially linked with circulatory profiles to identify more accessible biomarkers.

### 11.3 Respiratory Diseases

There have been several investigations into the application of octadecanoids as an indicator of respiratory diseases. Most of these studies have concentrated on obstructive lung diseases (e.g., asthma, COPD) with a focus on the EpOMEs and DiHOMEs. The role of these compounds in the lung has been previously reviewed,^754^ with emphasis on the molecular and cellular mechanisms of EpOME-induced acute lung injury. An octadecanoid signature has been suggested to be a marker of the transition from healthy smokers with normal lung function to COPD, specifically driven by increases in EpOMEs, DiHOMEs, and TriHOMEs.^755^ Follow-up work on the TriHOMEs identified these compounds to be strongly increased in COPD;^172^ however, production was due to autoxidation likely in association with the oxidative stress in COPD patients. Levels of the 12,13-DiHOME were reported to increase in bronchoalveolar lavage fluid (BALF) of rats exposed to nitronaphthalene and ozone.^756^ Plasma levels of the 9,10-DiHOME and 9-HODE increased in healthy individuals following 1h exposure to biodiesel,^757^ while 12,13-DiHOME and 13-HODE were increased in BALF of healthy individuals exposed to biodiesel.^758^

The HODEs and oxo-ODEs have been reported to increase in several studies as primarily markers of oxidative stress. In a small pilot study, multiple octadecanoids including DiHOMEs, 13-HODE, 13-oxo-ODE, and 13-HOTrE were detected in the nasal epithelium of asthmatics and healthy controls. Only the 13-HOTrE evidenced a dysregulation, with decreases observed in asthmatics.^759^ The 9,10-DiHOME has been shown to correlate with lung function,^755^ and was reported to be at higher concentrations in circulation among a subgroup of asthmatics with the lowest lung function.^760^ Metabolomics analysis of sputum of asthmatics identified LA metabolism as the most significant pathway to discriminate between neutrophilic, eosinophilic and paucigranulocytic asthma, but did not identify any octadecanoid products.^761^ Panda et al. reported that the 13-HODE partially leads to steroid-resistant asthma features through nuclear factor (NF)-κb.^762^ Henricks et al. showed that 13-HODE (0.14 nmol) enhanced the increases in pulmonary resistance observed after administration of the contractile agents histamine or methacholine, in anesthetized, spontaneously breathing guinea pigs. These results indicate that 13-HODE may play an important role in the induction of airway hyperresponsiveness, a characteristic features of asthma, *in vivo*.^763^ It has been speculated that LA metabolism may have implications for the individualized treatment of neutrophilic asthma.

More recently, the DiHOMEs have been suggested to be biomarkers of COVID, with circulatory levels reported to associate with severe disease. A pilot study from Hammock and colleagues reported significant increases in the EpOMEs and DiHOMEs in plasma of 6 patients with laboratory-confirmed severe SARS-CoV-2 infection.^764^ Of the lipids analyzed, 18 had a > 4 fold-change and false discovery rate (*p* < 0.01), making a case for the “potential biomarker” claim in the title of the paper. The authors also noted that “incorporating diols in plasma multi-omics of patients could illuminate the COVID-19 pathological signature along with other lipid mediators and blood chemistry”. In a study of patients with varying COVID severity, 5 octadecanoids were elevated in plasma from intensive care unit patients, including 2–5-fold increases in both DiHOMEs as well as 12,13-DiHODE, while 13-HODE and 9-HOTrE increased 1–2-fold and there was surprisingly no change in 9-HODE levels.^765^ A study of individuals who had recovered from COVID reported decreased plasma levels of 13-oxo-ODE relative to individuals diagnosed with long COVID.^766^ There was no difference between healthy controls and individuals diagnosed with long COVID, indicating that 13-oxo-ODE would not be a useful biomarker of long COVID. This study explored potential mechanisms using a macrophage cell line to test the hypothesis that individuals with long COVID had alternatively polarized macrophages. They reported that the 12,13-DiHOME and a peak characterized as HpODEs were elevated in M2-like macrophages relative to M1 and concluded that system-wide alternative macrophage polarization is a key cell mechanism accounting for long COVID symptoms. A clinical trial treated hospitalized COVID patients with EPA for 3 days and found that the 9,10-DiHOME levels decreased relative to the placebo group.^767^ The uncontrolled inflammatory response in COVID-19 is characterized by a high neutrophil to lymphocyte ratio, which was decreased by EPA supplementation. Given that the 9,10-DiHOME is preferentially formed by neutrophils, there is potential for use of this compound as a biomarker for severe COVID. While promising, this clinical trial included a small number of patients (*n* = 22), who were of advanced age (81 ± 6.1 years). There is a need to perform additional studies in a broader cohort with inclusion of additional control groups to account for nonrespiratory viral infection. A recent study by Edin et al. examined oxylipins in a mouse model of COVID with human angiotensin-converting enzyme 2 (ACE2) expression.^768^ The study investigated the effect of sEH inhibition upon the host response to infection, focusing on the ensuring eicosanoid and cytokine storms. While circulatory levels of the DiHOMEs increased with infection and decreased with sEH inhibitor treatment, there was no overall effect on morbidity or mortality. A comprehensive multiomics study reported increases in the plasma levels of multiple LA-derived octadecanoids in adults with COVID-19 and in children with multisystem inflammatory syndrome in children (MIS-C) including the 12,13-DiHOME as well as 9-HpODE, 9-HODE and 13-KODE.^769^ Aside from COVID-19, there is less known about the role of octadecanoids in respiratory infections. The ratio of 13- to 9-HODE in bronchoalveolar lavage (BAL) was identified as a potential biomarker for immune status during an active influenza infection in a mouse model.^770^ The findings were replicated in nasopharyngeal lavage fluid of individuals with an active influenza infection, reporting an increased ratio in individuals with a high disease burden. The authors hypothesized that the ratio of 13-/9-HODE ratio reflected the balance of “anti-inflammatory” 13-HODE to “pro-inflammatory” 9-HODE, which is potentially an oversimplification of the inflammatory functions of these compounds.

The utility of octadecanoid profiles as biomarkers of respiratory diseases is promising. While early studies have demonstrated promise in circulatory profiles being associated with asthma severity and the transition from healthy smoker to a COPD smoker, additional investigations are needed to examine if these associations can be replicated in larger and more diverse cohorts. In particular, given the increasing focus on the role of the gut-lung axis in the etiology of respiratory diseases, emphasis should be placed on examining the utility of gut microbiome-derived octadecanoids as biomarkers of obstructive lung diseases. The association between COVID and circulatory DiHOME levels is of interest, and mechanistically plausible given the role of the DiHOMEs in ARDS and pulmonary damage. Further work should investigate the specificity of the DiHOME signature for COVID severity and particularly to determine if the levels decrease with the resolution of COVID symptoms.

### 11.4 Liver Diseases

There have been several investigations into the relationship between octadecanoid levels in liver diseases, with a focus on nonalcoholic fatty liver disease (NAFLD) and nonalcoholic steatohepatitis (NASH). Maciejewska et al. studied the role of 9-HODE in NAFLD in 24 patients (12 in the first stage and 12 in the second stage of NAFLD). Levels of HODEs were compared in plasma between the first and the second stage of hepatic steatosis. 9-HODE was present in higher concentrations in patients with grade II steatosis due to the greater exposure of liver cells to oxidative stress during the progression of the disease. After hepatic steatosis resolution by a six-month dietary intervention, a significant decrease in the concentrations of both 9- and 13-HODEs was observed.^771^ In 2021, Mazi et al. reported elevations in a wide array of LA- and ALA-derived octadecanoids in NAFLD. Histology-matched Hispanic individuals had higher levels of TriHOMEs and EpOMEs than those of European ancestry, and DiHOME/EpOME ratios were important discriminators of ethnicity in those with full nonalcoholic steatohepatitis (i.e., NASH).^772^ Kirpich and colleagues have published several studies on the interaction between alcohol-associated liver diseases and bioactive lipid mediators.^773^ Using a mouse model of alcoholic steatohepatitis, Warner et al. showed that the levels of 13-HODE, 9,10-DiHOME, and 12,13-DiHOME increased in plasma.^774^ A follow-up study examined the combined effects of ethanol and a diet high in LA (based upon corn oil) in the same mouse model, and reported that plasma and liver levels of 9- and 13-HODE increased in response to the combined feeding.^676^ The authors concluded that LA-derived octadecanoid induction of a pro-inflammatory response in macrophages is a potential mechanism driving the progression from alcohol-induced steatosis to alcoholic steatohepatitis. Of interest though, Liang et al. published that administering a high fat (corn oil) diet did not increase the liver concentration of octadecanoids (or other oxylipins), suggesting that these compounds do not accumulate in the liver.^117^ Further work in 2021 reported that the levels of 13-HODE and 13-oxo-ODE as well as 9,10- and 12,13-DiHOMEs were elevated in heavy drinkers suffering from moderate alcohol-associated hepatitis (mAH) compared to patients with mild alcohol-associated liver disease. However, 9(10)-EpOME and 12(13)-EpOME were decreased in heavy drinkers regardless of the presence or absence of liver injury.^775^

There have been several studies investigating associations between circulatory octadecanoid levels and different forms of liver diseases. In 2010, Thum et al. determined the levels of 9(10)-*cis*-EpODA in plasma of patients suffering from chronic liver diseases, a condition that often displays impaired liver CYP enzyme activities. 9(10)-*cis*-EpODA plasma concentrations were significantly repressed in patients with hepatic disease compared with healthy subjects. Thus, 9(10)-*cis*-EpODA was proposed as a biomarker to assess liver function.^205^ The profile of circulating lipid mediators has been characterized in individuals with acute decompensation of cirrhosis; 59 lipid mediators (out of 100 screened) were detected in plasma from cirrhotic patients and were significantly associated with disease severity. Among them, 11(*E*)-13-oxo-9(10)-EpOME was associated with short-term mortality and was a marker of coagulation and liver failure.^776^ Investigations in patients with hepatitis infection observed increases in circulatory octadecanoids, with Yoshida et al. showing in 2008 that the levels of total-HODEs (including 13-HODE, 9(*E*),11(*E*)-13-HODE, 9-HODE, 10(*E*),12(*E*)-9-HODE, 10-HODE, and 12-HODE) significantly increased in plasma and liver of patients infected with hepatitis B and C viruses.^777^ Later, in 2018, a study showed that 13-HODE, 9,10- and 12,13-DiHOME increased in patients with liver cirrhosis and hepatocellular carcinoma and the diols correlated with the levels of α-fetoprotein (AFP), a marker of hepatocellular carcinoma.^778^

There is a clear relationship between circulatory octadecanoid profiles and liver diseases, but liver levels do not appear to correlate with dietary input. Elevated octadecanoids have been suggested to have a causal role in NALFD and reported to be a biomarker, with circulatory levels being predictive of acute liver failure. Interestingly, some of these effects may occur via TRPV1, with deficiency protecting against experimental alcohol-associated liver disease. The sEH has been proposed as a therapeutic target in liver diseases, with therapeutic efficacy reported in NAFLD, liver fibrosis, and portal hypertension,^779^ potentially in combination with farnesoid X receptor (FXR) modulation.^780^ These findings collectively suggest that dietary octadecanoids are involved with liver diseases and merit further investigation to their utility as markers of both chronic and acute diseases, and represent a potential treatment route via reduction in dietary LA.

### 11.5 Inflammatory Pain

In 2018, Jensen et al. investigated the effect of chronic inflammatory pain on oxylipin concentrations in a mouse model of amygdala and periaqueductal gray (PAG) chronic inflammatory pain. Twelve LA-derived octadecanoids were detected in both the PAG and amygdala. Interestingly, two hydroxy-epoxide compounds known to be present in the skin of rodents and humans, 9(*Z*)-11-OH-12(13)-EpOME, and 11(*E*)-13-OH-9(10)-EpOME, were detected for the first time in brain tissue.^627^ When chronic inflammatory pain was induced by Complete Freund’s Adjuvant treatment, the concentration of the most detected compounds decreased in the amygdala, while the concentrations of 9(10)-EpOME, 12(13)-EpOME, and 9,10,11-TriHOME were reduced in PAG compared to the placebo controls.^627^ This octadecanoid reduction in mice suffering from chronic inflammatory pain is surprising, because an increase of concentrations of most octadecanoids (HODEs, oxo-ODEs, diols, keto-epoxides and hydroxy-epoxides) was previously observed in peripheral tissues, DRG, trigeminal ganglia (TG) and the dorsal horn of the spinal cord in animal models of inflammatory pain by lipidomic analyses.^61,222,617^ Moreover, the levels of 9- and 13-HODEs as well as the related ketones increased in rodents suffering from heat and burn related pain of peripheral tissues.^195^ The implications of these findings are as of yet unclear, but warrant further investigation into the specificity of octadecanoids as markers of inflammatory pain.

### 11.6 Sepsis

Lipid metabolism is closely associated with sepsis, with reported correlations with disease severity and systemic inflammation.^781−783^ The role of oxylipins, particularly eicosanoids,^784,785^ has been examined in sepsis with a number of reviews.^786−788^ While the eicosanoids and SPMs have been investigated, there is significantly less known about octadecanoid involvement in this condition. Dalli et al. proposed that lipid mediator profiles from sepsis patients (*n* = 22) could serve as biomarkers of survival as well as development of ARDS.^789^ Sepsis nonsurvivors had greater plasma levels of inflammatory lipid mediators relative to sepsis survivors. However, no octadecanoids were included in these analyses. Bergmann et al. performed a study focusing on the DiHOMEs and EpOMEs in a burn-injured mouse model. They reported that DiHOME serum concentrations were significantly elevated, these increases could be ablated by administration of a sEH inhibitor, and that DiHOMEs rather than EpOMEs were the key driver of immune cell dysfunction through hyperinflammatory neutrophilic and impaired monocytic actions.^68^ These findings are consistent with Hamaguchi et al., who reported that 9,10- and 12,13-DiHOME, as well as 9- and 13-HODE, were elevated in plasma from a single sepsis patient with a fatal Sequential Organ Failure Assessment (SOFA) score of 12.^790^ In a dog model, circulatory levels of the 9-HODE and 12,13-DiHOME were suggested as biomarkers of sepsis, with circulatory levels increasing 13.2- and 15.0-fold, respectively in sepsis.^791^ Conversely, Sulaimin et al. reported in a study of sepsis patients (*n* = 274) that while AA-derived eicosanoid levels in plasma were elevated in poor outcome patients with sepsis compared to those with a more sustained and rapid recovery, there were no statistical differences in either 9- or 13-HODE levels.^792^ While there is general consistency in the literature that octadecanoids increase in sepsis patients, further investigation is necessary, particularly in terms of establishing the mechanism. For example, it has been shown that phospholipase activity affects the observed eicosanoid signature in sepsis, with increased secretory phospholipase A2 group IIA (sPLA2-IIA) levels associating with eicosanoid metabolism in patients with bacterial sepsis syndrome.^793,794^ Regardless, in the context of sepsis, monitoring changes in oxylipin profiles over time may provide an indication of the developing prognosis of a patient allowing adaptive therapeutic interventions.

### 11.7 Atopy

There have been multiple reports about linkages between dietary PUFA consumption and atopy, with reviews concluding that there is no relationship between atopy and exposure to ω6 PUFAs.^795^ Rucci et al. reported that in a population-based prospective cohort study of 4976 mothers in the second trimester, fatty acid levels in glycerophospholipids were associated with an increased risk of childhood eczema (1.21-fold) at age 6-years.^796^ The associations were primarily driven by LA, leading to the conclusion that higher maternal ω6 PUFA levels during pregnancy influence the risk of atopic diseases in childhood. While Rucci et al. did not observe a relationship with LA levels in the children and onset of atopy, the Ryukyus Child Health Study of 23 888 children in Japan (ages 6–15 years) identified a positive relationship between dietary LA and eczema (OR = 1.27).^797^ However, few studies have investigated the relationship between octadecanoids and allergic sensitization or atopy. Lundström et al. examined the effect of birch allergen provocation in asthmatics and found that multiple LA- and ALA-derived compounds were increased in the BALF relative to healthy individuals.^798^ While the majority of these compounds were increased in asthmatic controls relative to healthy controls, they further increased following provocation. In particular, the TriHOMEs, EpOMEs and DiHOMEs increased 1.7–2.6-fold. Nontargeted metabolomics showed that the LA-derived metabolites (13-HODE, 13-oxo-ODE) were significantly associated with moderate-to-severe atopic dermatitis in fecal samples from 6-month-old infants.^799^ The levels of 13-oxo-ODE were significantly reduced in the moderate-to-severe atopic dermatitis group compared to those in the healthy control and mild atopic dermatitis groups. In addition, 13-oxo-ODE negatively correlated with the SCORAD index (*r* = −0.595, *p* < 0.01), and 13-HODE negatively correlated with egg white–specific IgE at 12 months of age (*r* = −0.411, *p* = 0.016). Treatment of individuals with allergic rhinitis with either single- or double-species mite subcutaneous immunotherapy (SM-SCIT and DM-SCIT) for 36 weeks showed that the downstream products of LA metabolism (e.g., 9-HpODE, 13-HODE) decreased in serum; however, there was no significant difference between the SM-SCIT and DM-SCIT groups.^800^ These metabolites, as well as a number of eicosanoids (e.g., 11-HETE) were suggested as potential biological indicators for monitoring the desensitization effect on house dust mite (HDM) SCIT.

An elevated ratio in serum of LA to total fatty acids in 12-month-old infants was reported to be associated with onset of allergy in infants; however, the study employed an NMR method that did not measure the octadecanoids. The study included data from 438 infants, of which 48 had reported food allergy.^801^ An earlier study by Yen and colleagues examined atopic dermatitis in children ages 2–17 years, with results reporting that atopic children had higher serum levels of LA, but lower levels of GLA and DGLA.^802^ The authors concluded that the pathogenesis of atopic dermatitis is related to deficiency in ω6 essential fatty acids, which are required for normal skin barrier function and protection against inflammatory changes in the skin.^802^ However, an earlier clinical trial in 102 subjects reported no effect of essential fatty acid supplementation upon atopic dermatitis.^803^ A pilot study in 20 atopic dermatitis patients reported that levels of 9,10,13-TriHOME in stratum corneum were elevated relative to healthy controls.^804^ This study used a tape-stripping method to sample the skin. Following correction with corneum total protein, the mean concentrations of total TriHOMEs in the atopic dermatitis group were 2.5- and 9.3-fold greater in the forehead skin and forearm skin, respectively, relative to normal controls. The study concluded that the noninvasive tape-stripping sampling may be useful for using TriHOME levels to monitor barrier function in atopic dermatitis. The antiallergic and anti-inflammatory effects of 12(*Z*)-10-HOME were investigated in a murine model of human atopic dermatitis. Addition of 12(*Z*)-10-HOME in the diet (0.01%, w/w) for 6 weeks decreased plasma IgE levels and skin infiltration of mast cells with a concomitant decrease in dermatitis score.^805^ While interesting, this study was relatively small in scope with 5 mice in each group, future work should expand the study and consider the role of parent LA in the mouse chow. Interestingly, an earlier study using a hairless mouse model of atopic dermatitis found that the development of atopic dermatitis-like symptoms was prevented by dietary supplementation with LA, but not with ALA.^806^ The study concluded that dietary-induced atopic dermatitis is mainly caused by deficiency of ω6 PUFAs, suggesting a potential therapeutic role of LA in atopic dermatitis.

Beyond the diet, environmental exposure is a significant octadecanoid source. While a number of groups have examined these effects, a limitation is that all of the reported environmental investigations presented here are cell based-studies. For example, pollen is rich in octadecanoids that have been demonstrated to exert a functional role in T2-mediated allergy. Plötz et al. examined the octadecanoid content of aqueous extracts of *Phleum pratense* L. (Timothy grass) and *Betula alba* L. (birch) pollen grains and measured the eosinophilic chemotaxic activity.^807^ Multiple LA-derived HODEs and ALA-derived HOTrEs were measured in the pollen, with chiral analyses demonstrating that the compounds were formed by autoxidation (racemic mixtures). The authors suggested that exposure to these lipid mediators may contribute to the elicitation and aggravation of eosinophil-associated inflammatory reactions by generating a T2-promoting local environment. Similar work from the group examined the biological activity of the same pollen fractions on polymorphonuclear granulocytes (PMNs), reporting that both LA- and ALA-derived monohydroxylated octadecanoids induced migratory responses as well as PMN activation.^808^ Traidl-Hoffmann and colleagues reported that the E_1_-PhytoPs from birch pollen grains modulate human dendritic cell function in a fashion that favors T2 cell polarization in a fashion similar to PGE_2_.^809^ Work by Mariani and colleagues identified the E_1_-PhytoPs to increase the capacity of LPS-stimulated dendritic cells to attract T2 cells, whereas the capacity to recruit T1 cells was reduced.^810^ Follow-up work concluded that the pollen-derived E_1_-PhytoPs modulate dendritic cell function via PPARα dependent pathways that lead to T2 polarization.^811^ The ability of pollen-associated E_1_-PhytoPs to impact cytokine secretion and maturation of dendritic cells was examined, with inhibition of IL-6 observed, but no effect upon LPS-induced surface expression of the maturation markers.^812^ More recently, the octadecanoid content of birch pollen was examined in more detail and reported multiple phytoprostanes as well as phytofurans.^813^ Bet v 1 is the main allergen in birch pollen, and E_1_-PhytoPs have been identified as novel ligands for this protein. These pollen-derived ligands enhance the proteolytic resistance of Bet v 1 and exhibit a dual role by stabilizing Bet v 1 and inhibiting cathepsin protease activity.^814^ The presence of phytoprostanes and phytofurans has also been investigated in date palm, *Phoenix dactylifera*, edible parts and byproducts. The highest concentrations of phytoPs (11375 ± 2201 ng/100 g dry weight (dw)) have been reported in pollen, and PhytoFs concentration is elevated in skin (329.15 ± 70.19 ng/100 g dw) and pollen (188.41 ± 28.53 ng/100 g dw).^815^ In addition, no HODEs were detected contrary to earlier reports.^808^

Given the hypothesis that dietary PUFAs are associated with atopy, there is a need to further explore the relationship between octadecanoids and onset of allergic sensitization. The role of octadecanoid lipid mediators in pollen in allergy has been previously reviewed.^816,817^ The data to date are varied, rendering it difficult to make a definitive assessment of the relationship. Further studies will need to address the timing of the dietary exposure (maternal vs. child exposure), the route of exposure (inhalation, dietary, contact), and the dose/response nature of the exposure as well as the putative biological activity of the parent PUFA relative to the octadecanoid metabolite.

### 11.8 Diuresis/Antidiuresis

Urinary levels of DiHOMEs and TriHOMEs are influenced by salt loading and depletion. Dreisbach et al. showed that intravenous salt loading increased, and salt depletion with oral furosemide decreased, the urinary excretion of these linoleate metabolites measured after glucuronidase treatment.^818^ Arachidonic acid epoxides are known to reduce the residence time of the furosemide sensitive Na^+^/K^+^/Cl^–^ cotransporter (NKCC) in the renal proximal tubule epithelial sodium channel to promote sodium clearance.^819,820^ In addition, arginine vasopressin system activation increases soluble epoxide hydrolase activity, shifting tissue DiHOME/EpOME ratios.^821^ Therefore, urinary levels of DiHOMEs, and possibly TriHOMEs, appear to be reporters of diuresis/antidiuresis processes in the kidney. This is of particular interest since such urinary octadecanoid profiles could complement classic measures of renal tubular function like electrolyte levels, osmolarity, or a furosemide stress test^822,823^ by providing measures of the kidney’s adaptive ability to respond to a physiological demand, rather than simply measuring the outcome of the response.

### 11.9 Type 2 Diabetes Mellitus and Metabolic Syndrome

In 2012, Grapov et al. demonstrated that diabetes was associated with increases in plasma EpOMEs and EpODEs in weight-matched obese women, with the 9(10)-EpODE/12(13)-EpODE ratio being an important indicator of diabetes status.^824^ In 2013, Umeno et al. reported that the singlet oxygen products 10- and 12-HODEs are potential biomarkers of early Type 2 diabetes melitus.^825^ Specifically, they reported that after saponification, fasting plasma levels of the 10- and 12-HODEs/LA ratio increased with other clinical markers of diabetes and could enable the identification of borderline diabetes without the use of an oral glucose tolerance test. More recently, esterified oxylipin profiling was found to provide a powerful discriminate model of metabolic syndrome, with shifts in a host of HODEs, oxo-OMEs, DiHOMEs, EpOMEs, and EpODEs detected.^826^ In 2022, Fedorova and colleagues published an elegant workflow to analyze the levels of esterified oxidized lipids in plasma in lean, obese, and Type 2 diabetic people. The results evidenced significant differences between groups of samples for 20 oxidized phosphatidylcholines, 26 oxidized triglycerides and 26 oxidized cholesteryl esters. Of these 72 lipids, 56 contained octadecanoids. Interestingly, 25 species were significantly increased in lean individuals. All of the species contained octadecanoids in their side chains.^827^ Therefore, octadecanoid profiling in the context of diabetes and cardiometabolic syndrome may provide insights into disease progression. A particularly relevant point from this work is the focus on the esterified species, demonstrating the importance of not only focusing on the nonesterified oxylipin pool.^380^ Hateley et al. investigated oxylipins in the omental WAT, liver biopsies and plasma of patients undergoing bariatric surgery. They reported that increases in the sEH activity index of 12(13)-EpOME:12,13-DiHOME in WAT and liver were a marker of worsening metabolic syndrome in patients with obesity.^828^

## 12 Conclusion

The formation of octadecanoids in mammalian systems has been known for decades, but often overlooked relative to the eicosanoids and more recently the docosanoids. The longer chain PUFAs have understandably taken the lead due to their potency, exquisite regulation, and demonstrated actions in diverse systems. On the other hand, the octadecanoids have often been dismissed as simply oxidized fat and a marker of lipid oxidation. However, as our understanding of regulatory lipid metabolism has been enriched from decades of research, interest in this class of molecules has begun to increase as we recognize that many octadecanoids are in fact potent contextual lipid mediators. While there has been a long-standing question as to whether the octadecanoids are bioactive, it is now clear that numerous members of this class are indeed potent lipid mediators. In fact, some octadecanoids are considered to be lipokines or serve as epigenetic modifiers. The expanding interest in these compounds necessitates a concomitant response from the scientific community to develop the necessary tools to investigate octadecanoid biology. There is to date a paucity of analytical methods and chemical standards from the octadecanoid field, but this is changing and should result in a concomitant increase in studies investigating octadecanoids. We also see a need to standardize the nomenclature and reporting of these compounds and to work with repositories such as LIPID MAPS and HMDB to develop our understanding of their endogenous concentrations and tissue distributions. It is of particular importance to ascertain which aspects of octadecanoid biology are well established (i.e., reproduced) vs. proposed, especially in the context of describing the biology, biochemistry and signaling actions of these compounds. Ultimately, many important challenges remain in the octadecanoid field.

As is often the case with lipid mediators, there is little knowledge with regards to specific octadecanoid receptors. There is a need to perform much of the basic biochemical characterization of these compounds that has been done for other fatty acid cascades. We need to determine which cell types produce which octadecanoids under which conditions. There is a necessity to determine the range of their production, specifically in terms of triggers of biosynthesis (e.g., calcium ionophore, anti-IgE, and LPS) as well as the effects of cytokines and growth factors that affect the fidelity of the system (e.g., alarmins, IL-25, IL-33). As a component of understanding the mechanisms of formation, the effects of pharmacological interventions with enzyme (e.g., COX, LOX, CYP, sEH) inhibitors on lipid mediator formation should be examined. These efforts need to be augmented with investigations of the putative biological activity, which remains unknown for the vast majority of octadecanoids. In particular, identifying specific receptors will be key in developing an understanding of their biology. A comprehensive evaluation of octadecanoids as effectors of the entire TRP superfamily would be extremely valuable and may unravel potential mechanisms of action in various cell systems.

A limitation in the field is that the majority of studies to date have focused on the canonical LA-derived octadecanoids (e.g., HODEs, EpOMEs, DiHOMEs). In particular, there is a need to investigate the bacterial and fungal derived octadecanoids which constitute a rich source of unique structures whose biological activity has not been investigated. In addition, the low abundant and lesser-known octadecanoids should be characterized. For example, SDA is likely absent or present at very low concentrations in the general population; however, this lipid is present in the food supply (as well as in dietary supplements). The oxidative metabolism of this fatty acid has not been investigated, likely due to a lack of analytical standards and the general emphasis on more abundant precursors. As awareness increases of the richness of the full octadecanoid cascade from multiple C18-FAs, there is a need to cast a broader net and investigate these other octadecanoids, with plant species rich in the parent oils being an appropriate target to begin such an exploration. There is the potential that even if some compounds are not present endogenously, they may possess pharmacologic activity that could become relevant for individuals taking supplements and may even add value to some commodity crops. This work needs to include analytical characterization to establish endogenous levels under varying conditions as well as to characterize potential pharmacological activity. The application of chiral analyses will be useful to determine formation mechanisms. This is particularly relevant given that the majority of proposed octadecanoid biomarkers to date are HODEs, which readily form from oxidation of LA. While we have summarized multiple studies reporting octadecanoids as putative biomarkers of various diseases and pathologies, assigning mechanisms associated with the reported associations will be challenging. The most common associations reported are with the LA-derived 9- and 13-HODE, and may simply reflect shifts in systemic oxidation stress associated with general disease pathology rather than specific disease mechanisms. Demonstrating the enantiomeric ratios of formation will add specificity to any proposed biomarker and immediately enhance our ability to interpret the implications of the associated changes. With the advent of new technologies including gas-phase chiral separations by ion mobility mass spectrometry, we believe that research in this area over the next decade could revolutionize our understanding of the role of octadecanoids in complex biological systems.

While only addressed briefly in this review, it is relevant to highlight that the vast majority of fatty acids, including the C18 octadecanoid precursors, are esterified and that the octadecanoids themselves primarily exist as esterified products. The free forms are presumably the biologically active oxylipins, but the functions of those found esterified within phospholipids are not known and should not be ignored. It has been demonstrated that HODE incorporation into inositol phosphates proceeds the formation of HODE-diacyl glycerols and that they have participated in intercellular signaling cascades.^829^ It is also a strong possibility that they could alter membrane properties, thereby influencing the function of membrane proteins, and/or act as a storage reservoir participating in the Lands cycle of lipolytic release and re-esterification as demonstrated for eicosanoids.

There is also a need to characterize the octadecanoid component of different food oils and high fat food stuffs particularly under different storage and preparation conditions. It is likely that these greatly affect dietary octadecanoids levels. In addition, when examining the biological activity of octadecanoids, it is important to place their dietary and subsequent endogenous level within context. Eicosanoid lipid mediators have been demonstrated to exert potent biological activity at low concentrations. However, levels of AA are estimated to be 0.051% of total energy intake vs. 0.72% for ALA and 7.2% for LA in the United States.^51^ Octadecanoids are subsequently likely to be present at endogenous concentrations that are orders of magnitude greater than eicosanoids.

The field of octadecanoid research is promising; however, there is much work required to adequately characterize this class of lipids in systems that directly impact human biology, including both host and microbiota biochemistry. Only through the concerted efforts of the lipid community will we accumulate sufficient knowledge regarding these compounds to establish their rightful place alongside their more well-known cousins, and establish the importance of octadecanoids to animals, as has been so well established in plants.^830−833^

## Dedication

This work is dedicated to Dr. Bruce Hammock, whose pioneering work in leukotoxins set the stage for this report of the octadecanoids.

## Abbreviations

AA: arachidonic acid
ACSL: acyl-CoA synthase
AD: Alzheimer’s disease
AdA: adrenic acid
ADP: adenosine diphosphate
AFP: α-fetoprotein
AKT: A serine/threonine protein kinase
ALA: α-linolenic acid
AMPP: *N*-(4-aminomethylphenyl)pyrimidium
ANT1: adenine nucleotide translocase 1
AOS: allene oxide synthase
ARDS: acute respiratory distress syndrome
ATP: adenosine 5′-triphosphate
BALF: bronchoalveolar lavage fluid
BAT: brown adipose tissue
CCS: collision cross section
CFA: complete Freund’s adjuvant
CGRP: calcitonin gene-related peptide
CLA: conjugated linoleic acid
CLA-DC: CLA-decarboxylase
CLA-DH: CLA-dehydrogenase
CLA-ER: CLA-enoyl reductase
CLA-HY: CLA-hydrolase
CLnA: conjugated linolenic acid
COPD: chronic obstructive pulmonary disease
COX: cyclooxygenase
CSF: cerebrospinal fluid
CYP: cytochrome P
Cys: cysteine
DESI: desorption electrospray ionization
DGLA: dihomo γ-linolenic acid
DHA: docosahexaenoic acid
DiHODA: dihydroxy-octadecanoic acid
DiHODE: dihydroxy-octadecadienoic acid
DiHOME: dihydroxy-octadecenoic acid
DiHOTE: dihydroxy-octadecatetraenoic acid
DiHOTrE: dihydroxy-octadecatrienoic acid
DMS: differencial mobility spectrometry
DM-SCIT: double-species mite subcutaneous immunotherapy
DNA: deoxyribonucleic acid
DOX: dioxygenase
DPA: docosapentaenoic acid
DRG: dorsal root ganglia
DSM-IV: Diagnostic and Statistical Manual of Mental Disorders IV
DSS: dextran sulfate sodium
dw: dry weight
EAS: epoxy alcohol synthase
ECD: electron capture detector
EDSS: expanded disability status scale
EET: epoxy-eicosatrienoic acid
EFA: essential fatty acid
EKODE: epoxy-keto-octadecadienoic acid
EKOME: epoxy-keto-octadecenoic acid
ELISA: enzyme linked immunoassay
ELOVL: elongation of very long chain fatty acids
eLOX: epidermal lipoxygenase
EOS: esterified omega-hydroxy sphingosine
Ep: epoxide
EPA: eicosapentaenoic acid
EPHX3: epoxide hydrolase EH3
EpODA: epoxy-octadecanoic acid
EpODE: epoxy-octadecadienoic acid
EpOME: epoxy-octadecenoic acid
EpOTrE: epoxy-octadecatrienoic acid
ESI: electron spray ionization
ETA: 8,11,14,17-eicosatetraenoic acid
FA: fatty acid
FABP4: fatty acid binding protein 4
FAD: flavin adenine dinucleotide
FADS: fatty acid desaturases
FA-HY: fatty acid hydratase
Fg-cat: fungal catalase
FID: flame ionization detector
fMLP: formyl-methionyl-leucylphenylalanine
FMN: flavin mononucleotide
FXR: farnesoid X receptor
GC: gas chromatography
GLA: γ-linolenic acid
GLP-1: glucagon-like peptide-1
GM-CSF: granulocyte-macrophage colony-stimulating factor
GPCR: G protein-coupled receptor
HDM: house dust mite
HETE: hydroxy-eicosatetraenoic acid
HFD: high fat diet
HODA: hydroxy-octadecanoic acid
HODE: hydroxy-octadecadienoic acid
HOME: hydroxy-octadecenoic acid
HOTE: hydroxy-octadecatetraenoic acid
HOTrE: hydroxy-octadecatrienoic acid
Hp: hydroperoxide
HpETE: hydroperoxy-eicosatetraenoic acid
HPLC: high-performance liquid chromatography
HpODA: hydroperoxy-octadecanoic acid
HpODE: hydroperoxy-octadecadienoic acid
HpOME: hydroperoxy-octadecenoic acid
HpOTE: hydroperoxy-octadecatetraenoic acid
HpOTrE: hydroperoxy-octadecatrienoic acid
HRMS: high resolution mass spectrometry
HWE: Horner–Wadsworth–Emmons reaction
IAC: immunoaffinity chromatography
IL: interleukin
IMS: ion mobility spectrometry
IsoF: isofuran
IsoP: isoprostane
JNK: c-Jun N-terminal kinase
Keap: Kelch-like ECH-associated protein
KETE: keto-eicosatetraenoic acid
LA: linoleic acid
LC-MS/MS: liquid chromatography tandem-mass spectrometry
LC-PUFA: long chain PUFA
LDL: low-density lipoprotein
LDS: linoleate diol synthase
LLOD: low limit of detection
LLOQ: low limit of quantification
LOX: lipoxygenase
LPS: lipopolysaccharide
LTB_4_: leukotriene B4
MA: Mead acid
mAH: moderate alcohol-associated hepatitis
MCP-1: monocyte chemoattractant protein-1
mDC: matured dendritic cell
MRM: multiple reaction monitoring
MSI: mass spectrometry imaging
MUFA: monounsaturated fatty acid
MW: microwave
NAD: nicotinamide adenine dinucleotide
NADPH: nicotinamide adenine dinucleotide phosphate
NAFLD: nonalcoholic fatty liver disease
NASH: nonalcoholic steatohepatitis
NF-E2: nuclear factor erythroid 2
NKCC: Na^+^/K^+^/Cl^–^ cotransporter
NLRP3: NLR family pyrin domain containing 3
NMR: nuclear magnetic resonance
NO_2_-FA: nitrated fatty acids
NODE: nitro-octadecadienoic acid
NOME: nitro-octadecenoic acid
NOTrE: nitro-octadecatrienoic acid
NRF2: nuclear factor erythroid 2-related factor-2
OA: oleic acid
ODA: octadecanoic acid
ODE: octadecadienoic acid
OME: octadecenoic acid
OPDA: 12-oxo-phytodienoic acid
OTE: octadecatetraenoic acid
OTrE: octadecatrienoic acid
PAG: periaqueductal gray
PCET: proton-coupled electron transfer
PG: prostaglandin
PhytoF: phytofuran
PhytoP: phytoprostane
PLA_2_: phospholipase A2
PLS: partial least-squares
PMN: polymorphonuclear granulocyte
PPAR: peroxisome proliferator-activated receptor
PUFA: polyunsaturated fatty acid
RIA: radio immunoassay
RNS: reactive nitrogen species
ROS: reactive oxygen species
SDA: stearidonic acid
sEH: soluble epoxide hydrolase
SM-SCIT: single-species mite subcutaneous immunotherapy
SPM: specialized pro-resolving mediator
T2D: type-2 diabetes
TG: trigeminal ganglia
THFA: tetrahydrofuranyl fatty acids
TLC: thin layer chromatography
TLR4: toll-like receptor 4
TNF-α: tumor necrosis factor alpha
TPP: 5,10,15,20-tetraphenyl-21*H*,23*H*-porphyrin
TPPU: 1-(1-propanoylpiperidin-4-yl)-3-[4-(trifluoromethoxy)phenyl]urea
Treg: regulatory T cell
TriHODA: trihydroxy-octadecanoic acid
TriHODE: trihydroxy-octadecadienoic acid
TriHOME: trihydroxy-octadecenoic acid
TriHOTrE: trihydroxy-octadecatrienoic acid
TRPV: transient receptor potential vanilloid
Tyr: tyrosine
UPLC: uultraperformance liquid chromatography
UV: ultraviolet
WMH: white matter hyperintensities
